# JAX-CNV: A Whole-genome Sequencing-based Algorithm for Copy Number Detection at Clinical Grade Level

**DOI:** 10.1016/j.gpb.2021.06.003

**Published:** 2022-01-25

**Authors:** Wan-Ping Lee, Qihui Zhu, Xiaofei Yang, Silvia Liu, Eliza Cerveira, Mallory Ryan, Adam Mil-Homens, Lauren Bellfy, Kai Ye, Charles Lee, Chengsheng Zhang

**Affiliations:** 1 Precision Medicine Center, The First Affiliated Hospital of Xi’an Jiaotong University, Xi’an 710061, China; 2 The Jackson Laboratory for Genomic Medicine, Farmington, CT 06032, USA; 3 School of Cyber Science and Engineering, Xi’an Jiaotong University, Xi’an 710049, China; 4 Department of Pathology and Laboratory Medicine, Perelman School of Medicine, University of Pennsylvania, Philadelphia, PA 19104, USA; 5 School of Computer Science and Technology, Faculty of Electronic and Information Engineering, Xi’an Jiaotong University, Xi’an 710049, China; 6 MOE Key Lab for Intelligent Networks & Networks Security, Faculty of Electronic and Information Engineering, Xi’an Jiaotong University, Xi’an 710049, China; 7 Department of Life Sciences, Ewha Womans University, Seoul 03760, South Korea

**Keywords:** Copy number variant, Chromosomal microarray assay, Whole-genome sequencing, JAX-CNV, Genetic testing

## Abstract

We aimed to develop a **whole-genome sequencing** (WGS)-based **copy number variant** (CNV) calling algorithm with the potential of replacing **chromosomal microarray assay** (CMA) for clinical diagnosis. **JAX-CNV** is thus developed for CNV detection from WGS data. The performance of this CNV calling algorithm was evaluated in a blinded manner on 31 samples and compared to the 112 CNVs reported by clinically validated CMAs for these 31 samples. The result showed that JAX-CNV recalled 100% of these CNVs. Besides, JAX-CNV identified an average of 30 CNVs per individual, respresenting an approximately seven-fold increase compared to calls of clinically validated CMAs. Experimental validation of 24 randomly selected CNVs showed one false positive, *i.e.*, a false discovery rate (FDR) of 4.17%. A robustness test on lower-coverage data revealed a 100% sensitivity for CNVs larger than 300 kb (the current threshold for College of American Pathologists) down to 10× coverage. For CNVs larger than 50 kb, sensitivities were 100% for coverages deeper than 20×, 97% for 15×, and 95% for 10×. We developed a WGS-based CNV pipeline, including this newly developed CNV caller JAX-CNV, and found it capable of detecting CMA-reported CNVs at a sensitivity of 100% with about a FDR of 4%. We propose that JAX-CNV could be further examined in a multi-institutional study to justify the transition of first-tier **genetic testing** from CMAs to WGS. JAX-CNV is available at https://github.com/TheJacksonLaboratory/JAX-CNV.

## Introduction

Copy number variants (CNVs) are known to play key roles in human evolution, genomic diversity, and disease susceptibility [1], [2], [3], [4], [5]. In addition, copy number changes have been reported to cause microdeletion and microduplication syndromes, such as Williams syndrome, Prader-Willi syndrome, Angelman syndrome, and DiGeorge syndrome [5], [6], [7], [8], [9]. Various technologies, including fluorescence *in situ* hybridization (FISH), PCR-based assays, chromosomal microarray assays (CMAs), and next-generation sequencing (NGS), have been developed in research and clinical laboratories for CNV detection. Since 2010, CMAs have been considered the first-tier test for patients with unexplained developmental delay or intellectual disability, autism spectrum disorders, and congenital anomalies [10], [11].

Over the past decade, advances in NGS technologies have brought unprecedented improvements in the throughput, speed, and cost of DNA sequencing. These improvements make whole-genome sequencing (WGS) feasible for broad use in research, with its ability to detect many types of genetic variations, and promise to offer the potential of a single test that captures nearly all genomic variations in an unbiased manner [12], [13], [14]. Although several WGS-based CNV calling algorithms were developed [15], [16], [17], [18], [19], [20], [21], none of them has been widely accepted for clinical applications, because callsets of those algorithms are not highly concordant with the current running assay (*i.e.*, CMA).

In addition to developing new CNV calling algorithms, integrative pipelines that combine multiple CNV calling algorithms to improve accuracy and overcome limitations of individual performance are commonly used. For example, Zhou et al. [22] developed a method to integrate callsets of CNVnator [19] and Lumpy [16]. Noll et al. proposed SKALD [23], which is based on consensus, filtered calls from BreakDancer [24] and GenomeSTRiP [25]. Trost et al. [26] developed a pipeline that employs CNVnator and ERDS [27] for CNV identification. However, most of those pipelines are not open source, and apply filters that are specifically developed for individual projects but not standardized. Thus, it is unknown whether the sensitivity and specificity of those pipelines are comparable to the standards in clinical diagnosis. Given current limitations, an algorithmic pipeline with sufficient sensitivity and specificity for clinical application is demanded.

Here, we present JAX-CNV, a newly developed WGS-based CNV calling algorithm. Its performance was evaluated on WGS data from 31 patient samples and compared to the callsets of the clinically validated CMAs at the Jackson Laboratory for Genomic Medicine (JAX-GM CMA). The result suggests that JAX-CNV has a high sensitivity (100%) necessary for diagnostic decisions and a low false discovery rate (FDR; 4%). This algorithm could serve as a basis for the use of WGS, as a replacement for array-based clinical genetic testing.

## Method

### WGS analysis workflow

#### Pre-processing

**Figure 1:**
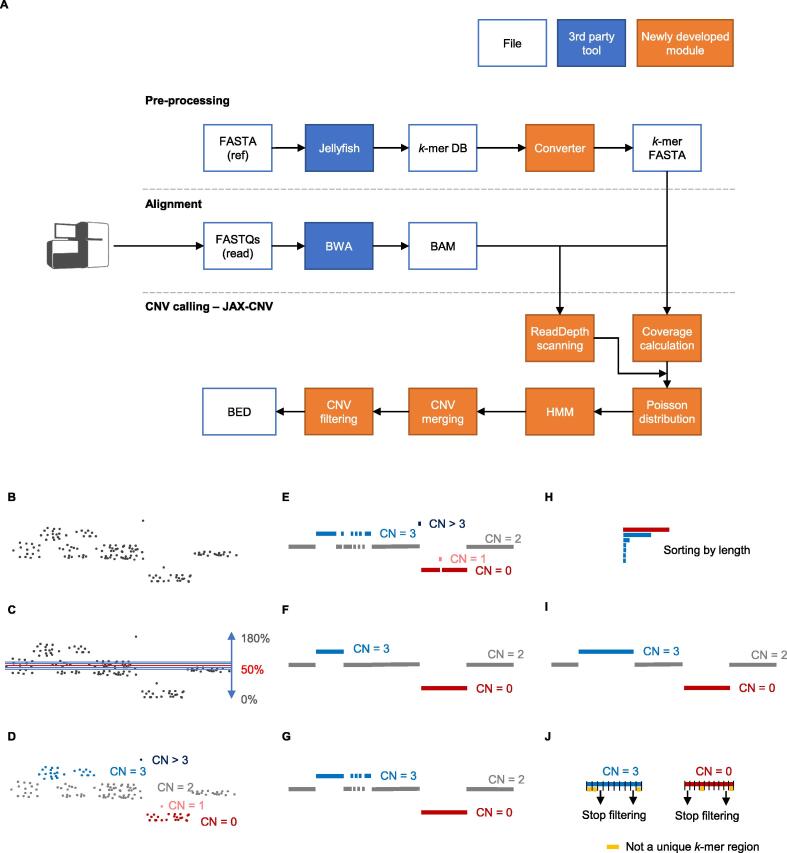
**WGS analysis workflow** **A.** An overview of the CNV calling pipeline consisting of three major steps: pre-processing, alignment, and CNV calling. **B.–J.** Details of CNV calling approach. WGS, whole-genome sequencing; CNV, copy number variant; DB, database; HMM, hidden Markov model.

The pre-processing step for a given reference genome (such as GRCh37, GRCh38, or other versions of human reference genome) includes BWA index (v0.7.15) and Jellyfish [28] count (v2.2.6). BWA index creates the required files for BWA alignment, while Jellyfish calculates the counts of each 25-mer of the reference genome and generates *k*-mer database (*k*-mer DB in Figure 1A) to indicate mappabilities of regions. This is similar to the concept proposed in CLImAT-HET [29], using *k*-mer for low mappability region identification. Theoretically, a larger *k* will increase the uniqueness of a *k*-mer against the given reference genome. However, that also requires larger computational resources to process larger *k*-mers. An experiment of different *k*-mers (*k* from 5 to 40) shows that a 25-mer achieves 76% unique *k*-mer (File S1; *k*-mer selection) and maintains the final sensitivity of 100% that is the result from the pipeline of multiple steps. The final sensitivity does not depend on *k*-mer selection solely. To efficiently compress the file size, each number in Jellyfish *k*-mer DB is converted to a character by calculating its log_2_ value added by 33 because ASCII code 33 in decimal is the first printable and visual character. For example, if a 25-mer has only one position in the genome, the log_2_ (1) value is zero. For converting the zero to the first printable ASCII code, 33 is added to the original log_2_ value (*k*-mer FASTA in Figure 1A). BWA index, Jellyfish count, and *k*-mer DB conversion may take 190 min, 105 min, and 403 min, respectively.

#### Alignment

The analysis could start as raw FASTQ files or BWA aligned BAM/CRAM files. If FASTQ is given, FastQC (v0.11.5) would be applied additionally in the pipeline for quality control. Then, BWA mem (v0.7.15) is employed for mapping reads against a given human reference genome. Once alignments are obtained (from our pipeline or given by users), alignments are sorted by SAMtools and used as the input file of JAX-CNV. Figure 1B illustrates read depths after alignment.

#### CNV calling

##### Coverage calculation

JAX-CNV uses the log_2_ (25-mer) FASTA-format file from the pre-processing step to scan the high-confident mapping (unique) regions for an accurate coverage calculation. A region is considered as a high-confident mapping region when each 25-mer count in the region is one, and the size of the region is larger than 20 kb. For each chromosome, we calculate an average coverage based on those 20 high-confident regions on a given BAM/CRAM of a sample. Once average coverages of all chromosomes are obtained, the interquartile range is applied to filter outliers. Outliers could indicate trisomy, monosomy, and other gross chromosome number anomalies of a sample. Then, an overall coverage of the sequenced sample is calculated based on average coverages of all chromosomes excluding the outliers. Using the interquartile range method, we are able to detect aneuploidies. For those aneuploidies, we will not detect any smaller CNVs on the respective chromosomes in the further steps.

##### Hidden Markov model

The overall coverage of the sequenced sample is used as the baseline and set to the 50th percentile (Figure 1C). Next, we scan the BAM/CRAM by shifting bins (the default bin size is set to 50 bp, and it is user-adjustable by “--bin”) and assign a percentile for each bin according to the difference between the overall coverage and the read depth of the bin. The percentiles normally range from 0% to 180%, and 180%–200% are reserved for copy numbers larger than two. For example, if the overall coverage is 50× and a read depth of a bin is 100×, the percentile of the bin will be 100% [(100/50) × 50%; Figure 1C]. Then, a hidden Markov model (HMM) with a Poisson distribution [30] is applied to convert the percentile of each bin to one of the five HMM CNV statuses: CN = 0 (loss), CN = 1 (loss), CN = 2 (normal), CN = 3 (gain), and CN > 3 (gain), (Figure 1D). Afterward, if the CNV statuses of the two adjacent bins are the same, we merge them into a segment (Figure 1E).

##### CNV merging

Since the default bin size is set to 50 bp, which is smaller than the CNVs that we expect to detect, oscillations of CNV statuses could happen frequently as illustrated in Figure 1E. There is a small CN = 1 status inside a large CN = 0 region. Using larger bins may resolve oscillations, but it decreases sensitivity. To maintain high sensitivity, we set the default bin size to be 50 bp, which is user-adjustable by “--bin”, followed by a merging step. The merging step is necessary to mitigate oscillations caused by the uneven read depth problem which is common in WGS alignments. To show oscillations of CNV statuses and uneven read depth problems, Figure S1 shows coverage, log_2_ (25-mer), and the ratio of low-quality alignments for the 112 truth CNVs.

If the length of a CNV status is shorter than 5 kb, then it will be absorbed by the previous status (Figure 1F) to form a cluster regardless of the CNV type (loss or gain). This status consolidation may be too aggressive, namely merging too many other CNV types together into a cluster, leading to incorrect results. Thus, for each status consolidation, if the original CNV type of a cluster covers less than 80% of the total length of the current cluster, the merging stops and the CNV calling status reinstates the original status (Figure 1G). This approach also prevents small statuses from merging into large statuses.

After the completion of merging, all CNV regions are sorted by their respective lengths (Figure 1H). From the largest to the smallest, each CNV region checks other CNVs of the same type (loss or gain) on their downstream and upstream coordinates from the nearest to the farthest for further clustering (Figure 1I). The aforementioned procedure stops when encountering a CNV with a different type (loss or gain). This step allows larger CNVs to cross normal status (CN = 2) and to merge smaller CNVs nearby. Starting from the largest CNV enables larger CNVs to have more opportunities to cross normal statuses and merge with other CNVs of the same type. Candidate CNVs are then generated.

#### CNV filtering

For each candidate CNV obtained from the previous step, CNV calling: CNV merging, we divide it into ten bins of equal length. Each bin is assigned a unique value corresponding to the count of unique *k*-mers (*i.e.*, the number of 25-mers that have a unique position in the reference genome). Starting from the bin with the smallest coordinate (the most left) to the bin with the largest coordinate, we filter a bin if the uniqueness value is low (percentage of unique *k*-mers is lower than 60% by default; user-adjustable by “--unique_kmer”). The procedure stops after encountering a low uniqueness bin (Figure 1J). The aforementioned procedure is repeated from the bin with the largest coordinate (the most right) to the bin with the smallest coordinate. This approach helps us trim the tails of CNVs in low-confident mapping regions.

Then, the density-based spatial clustering of applications with noise (DBSCAN) algorithm [31] is employed to cluster the candidate CNV fragments as the final step to making a CNV call. For this final step, using DBSCAN helps us to have a global view of CNV regions and consolidate them better. Specifically, we first sort the candidate CNV fragments based on their coordinates. Then, we separate the fragments into different raw clusters by two conditions: 1) the distance of any two continuous fragments is <3 Mb, and 2) the types (loss or gain) of all fragments are located in the raw cluster region. Next, for each raw cluster, we calculate the distance of every continuous fragment pair. The mean distance of the raw cluster is also calculated. The DBSCAN function (in DBSCAN R package) is applied to the distance matrix of each raw cluster. The procedure ends when the cluster results are not changed (File S1; DBSCAN). We report CNVs for a given individual in a BED-format file.

### Assessment of pathogenicity of a CNV

The criteria and guidelines used for the classification and interpretation of CNVs were published previously [10], [11], [32]. In general, a CNV is classified as pathogenic if 1) it overlaps genomic coordinates for a well-known deletion or duplication syndrome; or 2) it contains disease genes reported in GeneReviews (https://www.ncbi.nlm.nih.gov/books/NBK1116/), OMIM (https://www.omim.org/), or Decipher (https://decipher.sanger.ac.uk/disorders#syndromes/overview).

### Droplet digital PCR validation

Droplet digital PCR (ddPCR) assays were performed to examine the accuracy of the genomic aberrations detected by the JAX-GM CMA platform (File S1; ddPCR validation) and the JAX-CNV algorithm. The customized assays utilized primers designed by Primer3Plus [33], based on the GRCh38 assembly. All primer pairs were tested for their uniqueness across the human genome using *in silico* PCR from UCSC Genome Browser. The BLAST-Like Alignment Tool (BLAT) search was also performed at the same time to make sure all primer candidates only hit one site of the human genome. Lastly, the NCBI 1000 Genomes Browser was used to check if there were any single nucleotide variations (SNVs) in the primer or probe-binding region. All primers and probes used in this study are listed in Table S1.

## Results

### Dataset

Currently, the microarray proficiency test offered by the College of American Pathologists (CAP) requests participating clinical laboratories to report CNVs larger than 300 kb [32]. We selected 31 samples associated with various constitutional disorders (*i.e.*, DiGeorge syndrome, Williams syndrome, Cri-du-chat syndrome, Smith-Magenis syndrome, Wolf-Hirschhorn syndrome, Miller-Dieker Lissencephaly syndrome, Tetralogy of Fallot syndrome, 1p deletion syndrome, 18p deletion syndrome, and Angelman syndrome) from the Corriell Institute. For 22 of these 31 samples, the Corriell Institute reports a total of 45 CNVs (25 deletions and 20 duplications, ranging from 101 kb to 94 Mb in size) (Table 1, Table S2).

**Table 1:** **Detection of the 45 Corriell-registered CNV calls by JAX-CNV pipeline**

**Corriell ID**	**Corriell description**	**CNV region (length)**	**CNV type**	**Pathogenic annotation**	**JAX-CNV**				
					**Original_coverage (30**×**–48**×**)**	**30×**	**20×**	**15×**	**10×**
GM02820	Chromosome aberration	9p24.3p13.3 (34.5 Mb)	DUP	G/M	+	+	+	+	+
		12q24.32 q24.33 (7.3 Mb)	DEL	G/M	+	+	+	+	+
GM03997	Derivative chromosome	5q35.1 (130 kb)	DUP	M	+	+	+	+	+
		12p13.33p12.2 (20.8 Mb)	DUP	G/D/M	+	+	+	+	+
		12q24.33 (623 kb)	DEL	G/M	+	+	+	+	+
GM05876	DiGeorge syndrome	22q11.21 (1.4 Mb)	DEL	G/D/M	+	+	+	+	+
GM09025	Ring chromosome	16q24.2 (383 kb)	DUP	G/M	+	+	+	+	+
		22q13.31q13.33 (2.9 Mb)	DEL	G/D/M	+	+	+	+	+
GM09209	Miller-Dieker Lissencephaly syndrome	17p13.3 (5.9 Mb)	DEL	G/D/M	+	+	+	+	+
GM09687	Recombinant chromosome	16p13.3 (1.1 Mb)	DEL	G/D/M	+	+	+	+	+
		16q22.1q24.3 (20 Mb)	DUP	G/M	+	+	+	+	+
GM09711	Dicentric chromosome	2q13 (140 kb)	DUP	G/M	+	+	+	+	*
		13q11q34 (94 Mb)	DUP	G/M	+	+	+	+	+
		13q34 (1.7 Mb)	DEL	M	+	+	+	+	+
GM10946	Recombinant chromosome	6p21.2p21.1 (964 kb)	DUP	G/M	+	+	+	+	+
		6p12.3 (780 kb)	DUP		+	+	+	+	+
		6q14.1q16.3 (25 Mb)	DEL	G/M	+	+	+	+	+
GM11428	Duplicated chromosome	3p26.3p26.2 (5.3 Mb)	DEL	G/M	+	+	+	+	+
		3q22.1q26.1 (29.8 Mb)	DUP	G/D/M	+	+	+	+	+
		3q26.1 (112.8 kb)	DEL		+	+	+	+	+
		3q26.1q29 (35.2 Mb)	DUP	G/M	+	+	+	+	+
GM11516	Angelman syndrome	15q11.2q13.1 (7 Mb)	DEL	G/D/M	+	+	+	+	+
GM13480	Williams syndrome	7q11.23 (1.6 Mb)	DEL	G/D/M	+	+	+	+	+
		9p24.1 (107.6 kb)	DUP		+	+	+	+	+
GM13590	Duplicated chromosome	2q11.2q21.1 (33.6 Mb)	DUP	G/M	+	+	+	+	+
		2q37.3 (119 kb)	DEL		+	+	+	+	+
		4q31.22 (101.5 kb)	DEL	M	+	+	+	+	+
		9p13.3 (120.3 kb)	DUP	G/M	+	+	+	*	*
		17q11.1 (101 kb)	DUP	M	+	+	+	+	+
GM13946	Williams syndrome	7q11.23q11.23 (1.6 Mb)	DEL	G/D/M	+	+	+	+	+
GM14164	Tetralogy of fallot	13q14.2 (47.9 Mb)	DEL	G/M	+	+	+	+	+
		22q11.21 (148.8 kb)	DUP	M	+	+	+	+	No
GM16580	18p deletion syndrome	18p11.32 (1.6 Mb)	DEL	M	+	+	+	+	+
		18q21.33q23 (13.5 Mb)	DUP	M	+	+	+	+	+
		18q23 (4.0 Mb)	DEL	G/M	+	+	+	+	+
GM16593	Cri-du-chat syndrome	5p15.3 (14.7 Mb)	DEL	G/M	+	+	+	+	+
		14q24.3 (2.7 Mb)	DEL	M	+	+	+	+	+
GM18828	Chromosome aberration	1q31.3 (118 kb)	DUP	G/M	+	+	+	+	No
		4p16.1 (140 kb)	DUP	M	+	+	+	+	+
GM20200	Isodicentric chromosome	1q31.3 (103 kb)	DEL	G/M	+	+	+	+	+
		15q11.1q13.1 (8.5 Mb)	DUP	G/D/M	+	+	+	+	+
GM20375	Angelman syndrome	15q11.2q13.1 (4.9 Mb)	DEL	G/D/M	+	+	+	+	+
GM20743	Smith-Magenis syndrome	17p11.2 (2.1 Mb)	DEL	G/D/M	+	+	+	+	+
GM22569	1p deletion syndrome	1p36.33 (5.5 Mb)	DEL	G/M	+	+	+	+	+
GM22601	Wolf-Hirschhorn syndrome	4p16.3 (25.0 Mb)	DEL	G/D/M	+	+	+	+	+

*Note*: In the “pathogenic annotation” column, ‘G’, ‘D’, and ‘M’ mean annotations from GeneReviews, Decipher, and OMIM databases, respectively. In the “JAX-CNV” column, ‘+’ denotes CNVs captured by the methods/coverages; ‘*’ denotes that CNVs are not 50% reciprocal overlapping but recovered in manual review; and ‘No’ means no call. DEL, deletion; DUP, duplication.

**Figure 2:**
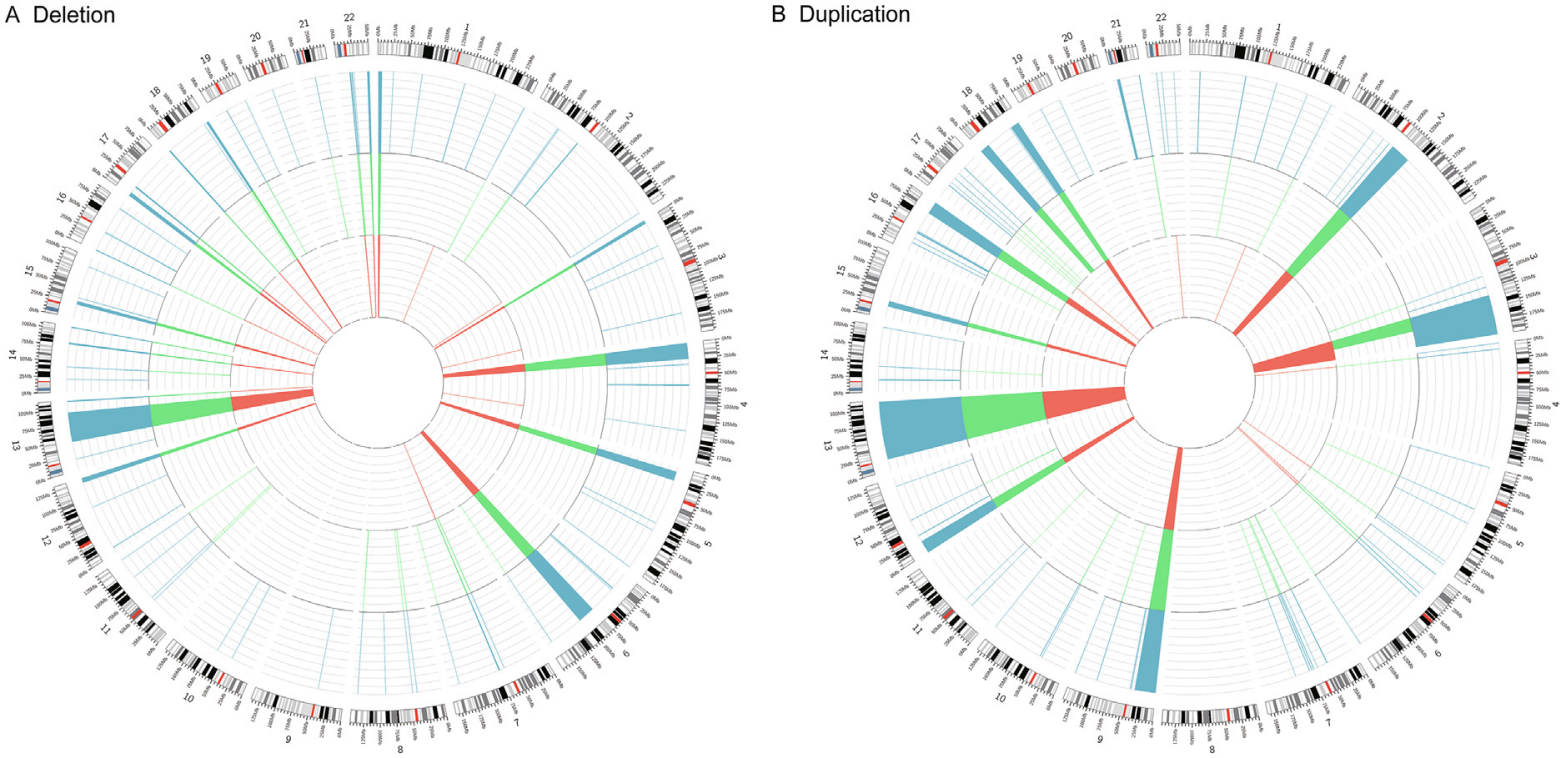
**JAX-CNV accurately detected all CNVs in the 31 testing samples** **A.** Deletion. **B.** Duplication. The 31 testing samples were examined by three different methods, including the Corriell Institute CMA (red inner circle), the JAX-GM CMA (green middle circle), and the JAX-CNV algorithm (blue outer circle).

These 31 samples were also examined with a clinically validated Affymetrix CytoScan HD platform (Affymetrix, Santa Clara, CA) for the detection of chromosomal imbalances following the standard operating procedures of the CLIA-certified laboratory at JAX-GM. The CMA data analysis was performed using the software supplied by the vendor (ChAS v3.3; methods: Affymetrix CytoScan HD analysis flow). The JAX-GM CMA platform reported an additional 67 CNVs among these 31 individuals, using a size cutoff of 50 kb (Figure 2; Table S3). In total, these 112 CNVs (65 deletions and 47 duplications, ranging from 51 kb to 94 Mb in size) were used as the truth set and set an initial baseline for sensitivity analysis. Of note, 70 of the 112 CNVs were considered to be pathogenic based on the previously published criteria and guidelines [32], [34], including 41Corriell-registered CNVs and 29 CNVs detected by the JAX-GM CMA.

### JAX-CNV showed a 100% recall rate of CNVs detected by arrays

We performed WGS on these 31 samples by Illumina paired-end sequencing at 30×–48× coverage with the read length of 2 × 150 bp (Table S4) in a blinded study manner. BWA was applied for short read alignment against the GRCh38 human reference genome (Chr1–22, X, Y, and mitochondria), followed by CNV identification using the JAX-CNV algorithm. JAX-CNV accurately detected all 112 CNVs of the truth set from WGS data (Figure 2; Table 1). Figure 2 shows the aggregated CNVs of all samples, and the locations of CNVs of each sample are given in Figure S2 and Table S5. Of note, due to the different resolutions of CMAs and WGS, there were three deletions ranging from 104 kb to 291 kb in size (one from GM20375 and the other two from GM20743) and three duplications ranging from 105 kb to 292 kb in size (GM09687, GM13480, and GM20743) that did not meet the benchmark of 50% reciprocal overlap with the JAX-CNV calls, but they were still located in the same regions with either smaller or larger size ones (Figure S3).

**Figure 3:**
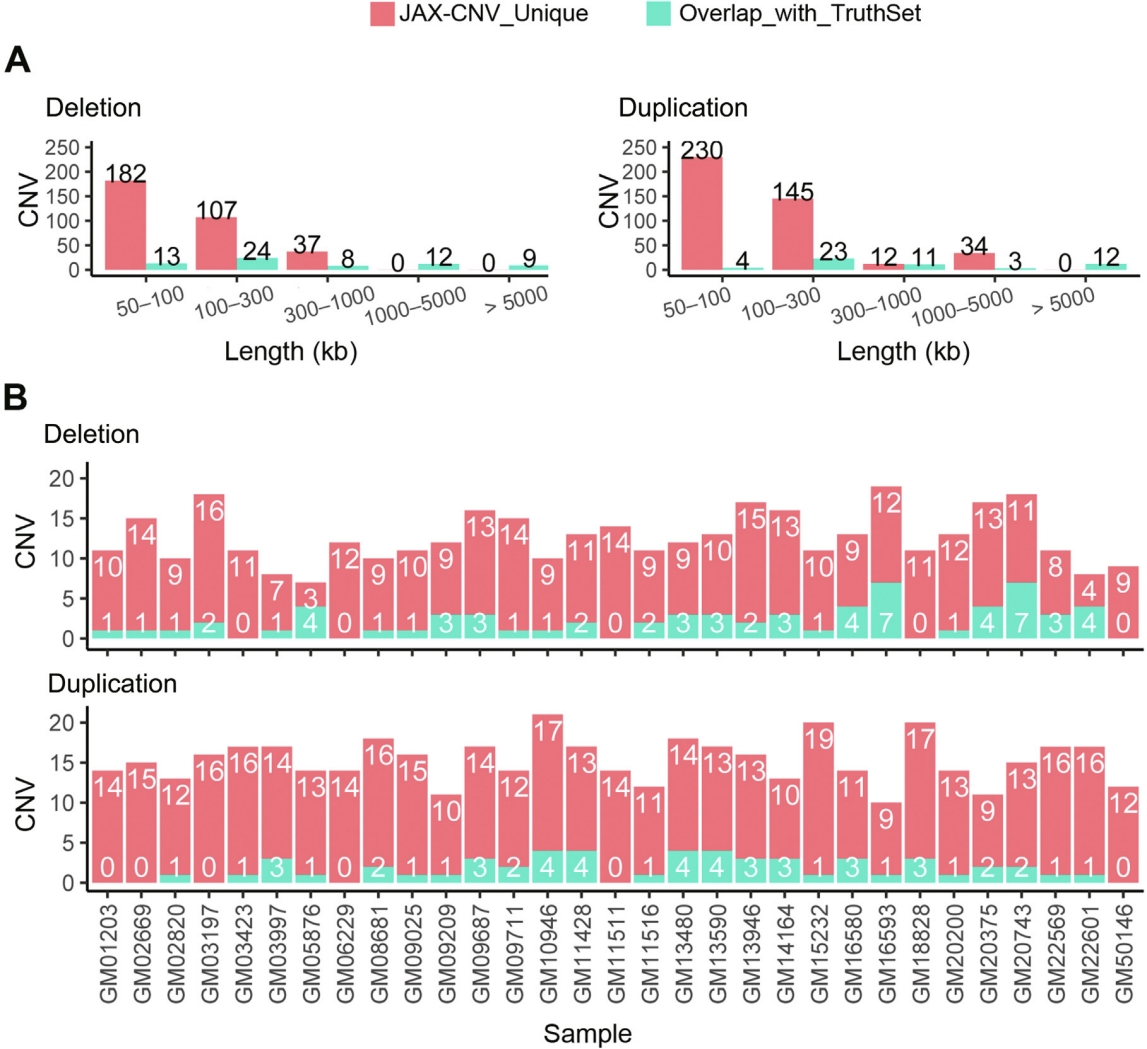
**JAX-CNV detected a number of unique CNV calls compared to the array-based method** **A.** Bar chart summarizing the unique JAX-CNV calls (in red) and CNV calls overlapping with the truth set (in green) as a function of CNV sizes. **B.** Concordance of CNV calls per sample.

Next, the CNV calls from JAX-CNV compared with the truth set. As shown in Figure 3A, JAX-CNV detected an additional 747 more CNVs than the array-based technology, and 89% of these calls were less than 300 kb in size, and 50% were less than 100 kb in size. Figure 3B further summarizes CNV calls of each sample. JAX-CNV used 119 calls to identify 112 CNVs in the truth set. For example, for a 20.8-Mb duplication at the chromosome region 12p13–12p12 in GM03997, JAX-CNV made two CNV calls (12.3 Mb and 8.5 Mb) across the 20.8-Mb duplicated region (Figure S4).

### JAX-CNV presented a 100% sensitivity and a 4.17% FDR for CNV calls larger than 50 kb

As described above, JAX-CNV detected 747 more CNVs (across 31 individuals), which resulted in an approximately seven-fold increase of CNV calls compared to the truth set (Table S5). To assess the accuracy of those additional calls, we randomly selected two samples, GM05876 and GM09209, for experimental validation. Compared to the truth set, there were 8 concordant CNV calls and 16 novel CNV calls in the two samples. Among the 16 novel CNV calls, 14 were validated by ddPCR (Table S6). For the two invalidated CNV calls, one (1450.3 kb loss at 21p11) was inconclusive due to an unclear separation of positive and negative droplet clusters in the ddPCR assay; the other one (duplication at 16p11; encompassing a segmental duplication and a simple repeat-rich region) was a false positive from JAX-CNV, which was detected in 28 of the 31 samples by JAX-CNV due to signal noise in this region. However, we did not simply filter this region because JAX-GM CMA identified a duplication in this region for GM16580. As a result, there were 15 CNVs (out of 24, larger than 50 kb in size) that were missed by the clinically validated CMAs and one false alspositive CNV call among the 24 randomly chosen JAX-CNV-based CNV calls. Together, these results represent a 100% sensitivity for CNV calls larger than 50 kb with a FDR of 4.17% (1/24) for JAX-CNV calls.

### JAX-CNV maintained a high sensitivity at 10× WGS coverage

Although the cost for NGS has dropped rapidly, its price still remains a big concern when WGS is considered as a first-tier assay in clinical diagnostics. To address this issue and assess the ability of JAX-CNV to accurately detect CNVs on low-coverage WGS datasets, we down-sampled the WGS data and assessed CNV calling sensitivity of JAX-CNV. These 31 testing samples were originally sequenced at coverages ranging from 30× to 48× (Table S4). The simulation of different coverages was performed by SAMBAMBA [35] on the aligned BAM files. The test series consisted of down-sampling to 30×, 20×, 15×, and 10× from the original 30×-to-48× coverage. JAX-CNV was then applied on WGS data at different coverages. The assessments on WGS data at 9× and 8× coverages were also performed. At 9× coverage, JAX-CNV failed to identify one deletion and 20 duplications (loss of sensitivity, 42%), and at 8× coverage, JAX-CNV failed to identify one deletion and 24 duplications (loss of sensitivity, 50%). Thus, 10× WGS coverage is the lowest coverage that JAX-CNV could maintain the sensitivity.

**Figure 4:**
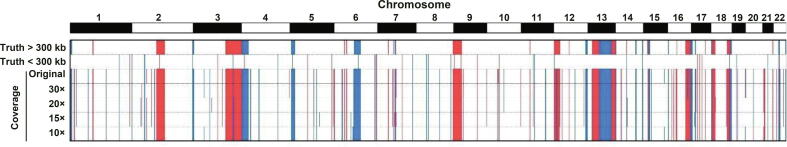
**JAX-CNV maintained a high sensitivity for CNV detection at 10× WGS coverage** The top two panels are the truth set using 300 kb as the cutoff. Red-colored bars indicate deletions, while blue-colored bars indicate duplications.

Among the 112 CNVs of the truth set, 50 were larger than 300 kb (CAP standard cutoff size). Even when the coverage was reduced to 10×, JAX-CNV remained 100% sensitive for detecting CNVs larger than 300 kb (Figure 4). At 15× coverage, JAX-CNV failed to identify a 79.9-kb duplication at the chromosome region 17q21.31 of GM05876 (Figure S5). At 10× coverage, JAX-CNV additionally failed to identify a 55-kb deletion at the chromosome region 11p11.12 of GM09687 and five duplications ranging from 52 kb to 204 kb in size (Figure 4, Figure S6). In summary, for deletions larger than 50 kb, the sensitivities were 100% at 30×, 20×, and 15× coverages and 98.5% at 10× coverage, while for duplications larger than 50 kb, the sensitivities are 100% at 30× and 20× coverages, 97.9% at 15× coverage, and 89.4% at 10× coverage.

### JAX-CNV matches clinical needs better than other CNV calling algorithms

We compared JAX-CNV to other CNV calling algorithms, Manta [15], Lumpy [16], Delly [17], CNVnator [19], and cn.MOPS [18], using the same WGS data. The scripts of performing those algorithms can be found in File S1. We also tested combined methods (pipelines) for detecting CNVs, such as FusorSV [36] and MetaSV [37]; however, the sensitivity assessments of these two pipelines failed, since both of them do not fully support GRCh38.

For the 112 CNVs of the truth set, Manta, Lumpy, Delly, CNVnator, and cn.MOPS identified 66, 61, 77, 100, and 20 of these CNVs, yielding sensitivities of 59%, 54%, 69%, 89%, and 18%, respectively (Figure S7). Some of the algorithms also incorrectly identified the CNV types (*e.g.*, a deletion instead of a duplication). In general, read-depth-based algorithms, such as JAX-CNV and CNVnator, have greater sensitivities in detecting genomic imbalances. Other algorithms that primarily use paired-end, split-read, or combinations of these strategies clearly show lower sensitivities in identifying large chromosomal imbalances. Moreover, Manta, Lumpy, and Delly identified tens of thousands of CNVs larger than 50 kb in size (39781, 35886, and 350183, respectivley) (Figure S7). Such big amount of identified CNVs poses a huge challenge for efficiently conducting clinical diagnosis and decision-making with these CNV algorithms.

With respect to speed, JAX-CNV took less than 1 h to finish CNV detection for a given sample (ranging from 29 min to 47 min for the 31 testing samples) by using a single thread. Comparatively, Manta, Lumpy, Delly, CNVnator, and cn.MOPS took 6 h, 6.1 h, 8.2 h, 1.1 h, and 1 h, respectively, to finish CNV calling for a single sample. Moreover, analyses with JAX-CNV were completed using 4.5 GB of memory.

## Discussion

Since WGS-based assays are able to detect all types of genetic variations [*e.g.*, SNVs, insertions and deletions (InDels), and structural variants (SVs)], it has the potential to eventually supplant karyotyping, CMAs, and exome sequencing for disease diagnosis. Despite the growing applications of WGS-based assays for detecting SNVs and InDels in clinical settings [38], [39], [40], [41], it still remains challenging to reliably detect CNVs for clinical diagnostics. While a number of CNV detection pipelines have been developed for research studies and further employed for clinical applications [26], [34], [42], none of them has been widely used for clinical diagnosis.

For research purpose pipelines, sacrificing specificity for sensitivity is beneficial to avoid missing potential CNVs. However, reporting hundreds or even thousands of chromosomal aberrations is impractical for clinical diagnosis both due to time constraints and the complexity of evaluating a large number of CNVs with unknown significance. Without careful clinical considerations, it is impossible for those tools to meet the sensitivity, specificity, and turnaround time requirements for disease diagnosis. Thus, there is still a need to develop novel or optimize existing bioinformatics tools and/or pipelines to improve the accuracy and turnaround time of the WGS-based assays for clinical applications. Hence, we develop a WGS-based CNV caller, JAX-CNV, showing a 100% sensitivity for detecting CNVs larger than 50 kb.

Currently, the HMM in JAX-CNV has five statuses of CNVs: CN = 0 (loss), CN = 1 (loss), CN = 2 (normal), CN = 3 (gain), and CN > 3 (gain). Thus, JAX-CNV can only indicate that a CNV status is >3 if it is. To indicate the exact copy number that is >3, JAX-CNV needs to check BAMs/CRAMs for read depth of the detected CNV region and detect the exact copy number in this region.

Since pathogenic chromosomal abnormalities are not limited to CNVs. Other SVs, including translocations and inversions, can also cause diseases [32]. We are currently developing new modules based on the established pipeline to accurately identify translocations and inversions. Detecting translocations and inversions is more difficult than detecting CNVs, and a major reliance on the read depth signal as employed in the current CNV caller will not be effective. Thus, we are considering the inclusion of paired-end alignment distance and orientation as inputs for identifying translocations and inversions in the next version of this pipeline. Breakpoints of inversions may be associated with deletions and thus further increase the detection difficulty. Nevertheless, previous studies have successfully detected translocations and inversions in WGS data [43]. Thus, we believe that with a careful design that reflects advanced knowledge of SVs, our future pipeline will become a comprehensive SV caller for clinical applications.

JAX-CNV is a newly developed WGS-based CNV algorithm for detecting deletions and duplications that are larger than 50 kb. The results obtained from the 31 Corriell samples demonstrated a 100% concordance between JAX-CNV calls and the calls registered by Corriell and detected by JAX-GM CMA. In addition to the high sensitivity and specificity, JAX-CNV is easy-to-use, stable, robust, and fast for detecting CNVs in WGS data. JAX-CNV requires 4.5 GB of memory and finishes CNV detection for a single sample in less than 1 h. JAX-CNV meets the sensitivity, specificity, reproducibility, and speed requirements necessary for clinical applications, and demonstrates the potential to supplant CMA-based methods as the first-tier diagnostic assay.

## Code availability

JAX-CNV is available at https://github.com/TheJacksonLaboratory/JAX-CNV.

## CRediT author statement

**Wan-Ping Lee:** Conceptualization, Methodology, Software, Formal analysis, Investigation, Data curation, Writing - original draft, Writing - review & editing, Visualization. **Qihui Zhu:** Validation, Data curation, Writing - original draft, Writing - review & editing. **Xiaofei Yang:** Methodology, Software. **Silvia Liu:** Methodology, Visualization. **Eliza Cerveira:** Validation, Data curation, Writing - original draft. **Mallory Ryan:** Validation, Data curation. **Adam Mil-Homens:** Validation, Data curation. **Lauren Bellfy:** Validation, Data curation. **Kai Ye:** Resources, Supervision. **Charles Lee:** Conceptualization, Methodology, Resources, Writing - original draft, Writing - review & editing, Supervision, Project administration, Funding acquisition. **Chengsheng Zhang:** Conceptualization, Methodology, Validation, Resources, Writing - original draft, Writing - review & editing, Supervision, Project administration. All authors have read and approved the final manuscript.

## Competing interests

The authors declare that they have no competing interests.

## Supplementary material

Supplementary File S1 **Supplementary methods**

Supplementary Figure S1 **Profiles of 112 CNVs in the truth set presented by Read Depth, log2(k-mer count) and Ratio of low-quality alignment, respectively** Each CNV consists of three panels that are, from the top to bottom, (1) Read Depth colored gray, (2) log2(k-mer_count) colored red, and (3) Ratio of low-quality alignment colored blue.

Supplementary Figure S2 **Common and unique CNVs in the 31 testing samples detected by three different methods** Each sub figure shows reported CNVs, from the inner circle to the outer circle, are from (1) Corriell Institute, (2) JAX-GM CMA, and (3) JAX-CNV on WGS. Deletions and duplications are colored red and blue, respectively.

Supplementary Figure S3 **JAX-CNV detected CNVs that were less than 50% reciprocal overlap with the ones detected by aCGH** Three deletions and three duplications that did not meet the benchmark of 50% reciprocal overlap with the JAX-CNV calls, but they were still located in the same regions with either smaller and larger size ones.

Supplementary Figure S4 **JAX-CNV detected a huge duplication with an arm-level size (20.8 Mb)** A 20.8 Mb duplication at the p arm of the chromosome 12 in the sample GM03997 was detected by JAX-CNV with two calls to cover it entirely.

Supplementary Figure S5 **A false negative result was observed with JAX-CNV at 15x WGS coverage** One duplication of 79.919 kb located in q21.31 region was detected by aCGH and JAX-CNV at 30x, 20x and 10x WGS coverages, but was missed by JAX-CNVat 15x WGS coverage data, suggesting a false negative result.

Supplementary Figure S6 **Decreased sensitivity of JAX-CNV at 10x WGS coverage** JAX-CNV was unable to detect one deletion and six duplications at 10x WGS coverage data.

Supplementary Figure S7 **The sensitivity and specificity of JAX-CNV are better than other calling algorithms** A. The recalled CNVs in the truth set of each algorithm. B. Numbers of total calls reported by each algorithm.

Supplementary Table S1 **Primers used for the ddPCR validation**

Supplementary Table S2 **CNV calls reported from the Corriell Institute**

Supplementary Table S3 **CNV calls reported by JAX-GM CMA**

Supplementary Table S4 **Sequence coverage**

Supplementary Table S5 **CNV calls reported by JAX-CNV**

Supplementary Table S6 **ddPCR validation result**
