# Supplementary figures and images for: JAX-CNV: A Whole-genome Sequencing-based Algorithm for Copy Number Detection at Clinical Grade Level
Source: Genomics Proteomics Bioinformatics. 2022 Jan 25;20(6):1197–206. doi: 10.1016/j.gpb.2021.06.003 (PMC10225484; doi:10.1016/j.gpb.2021.06.003)

**Figure S1.**

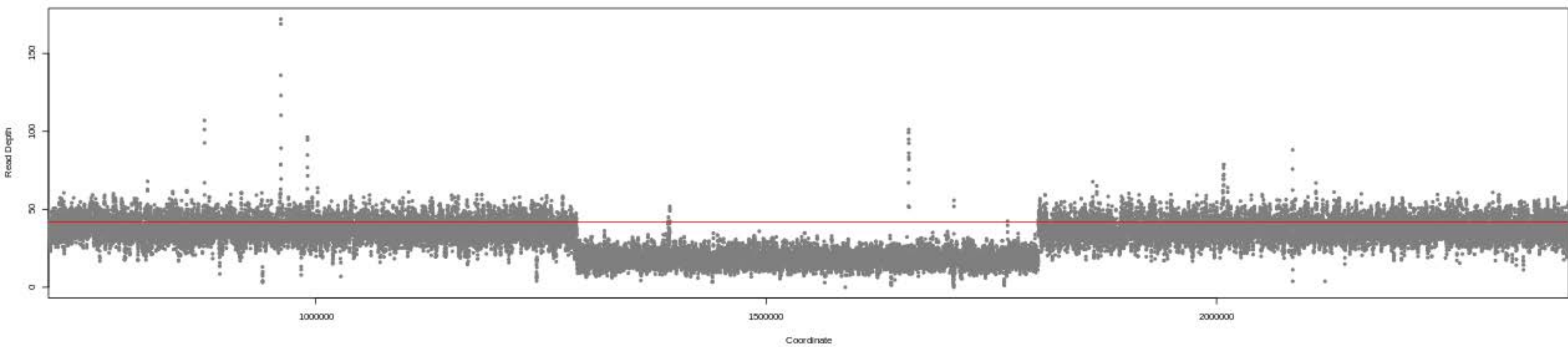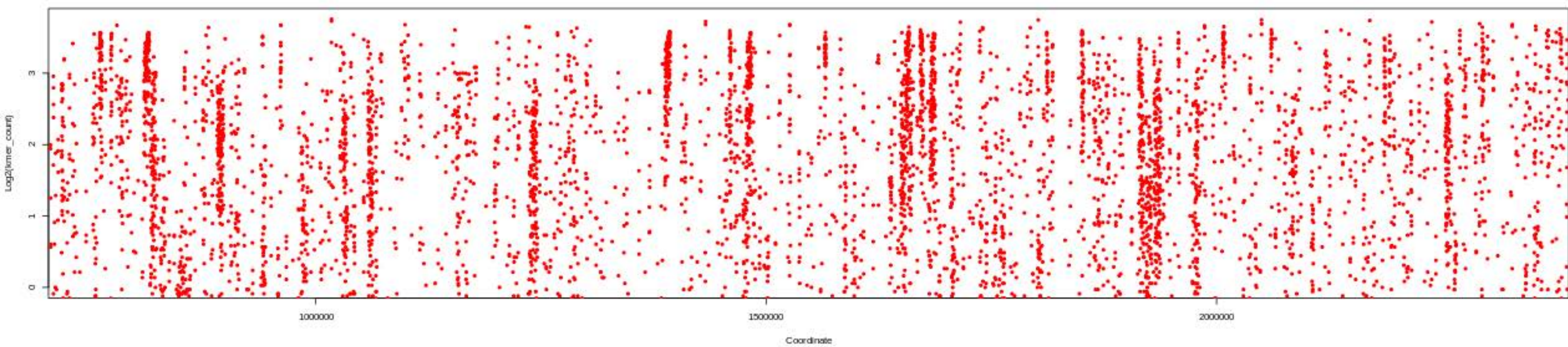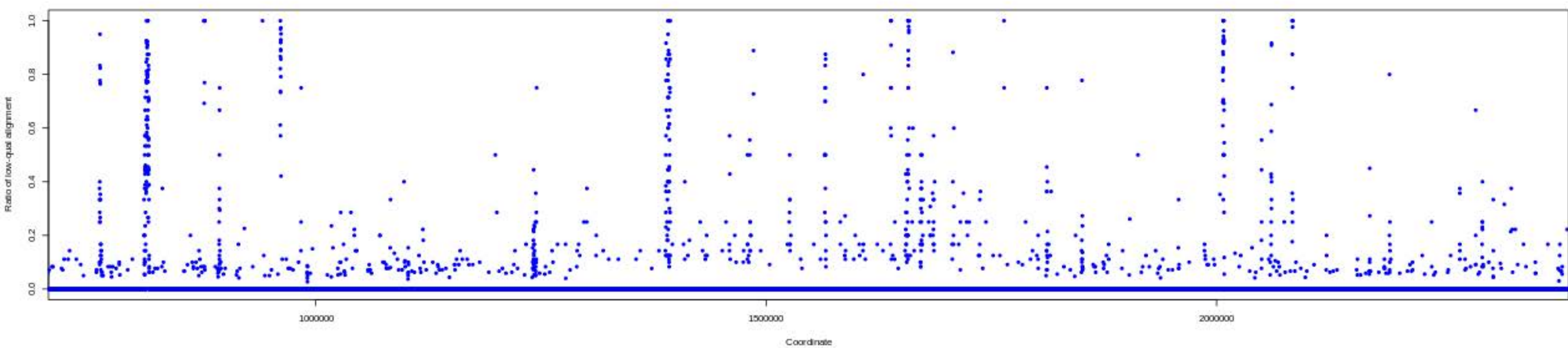

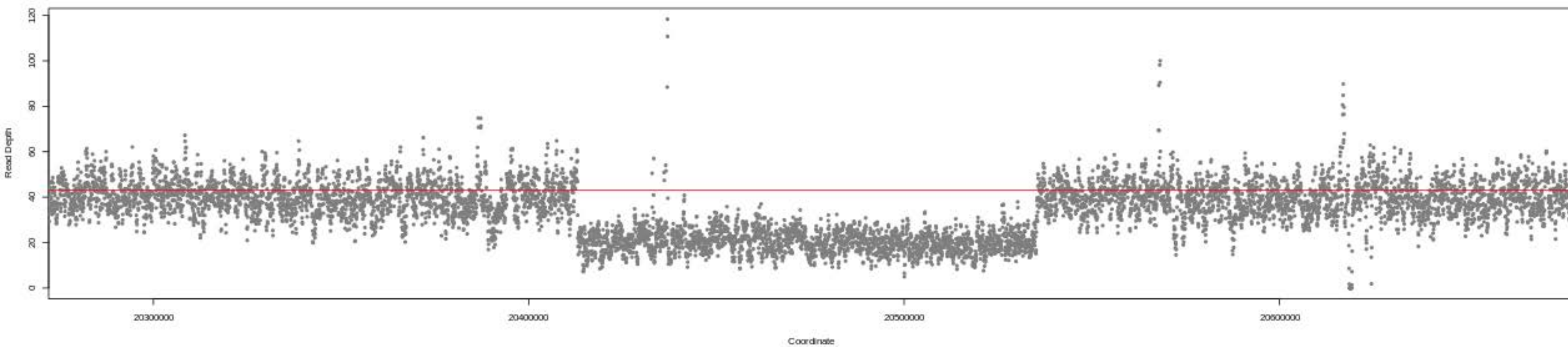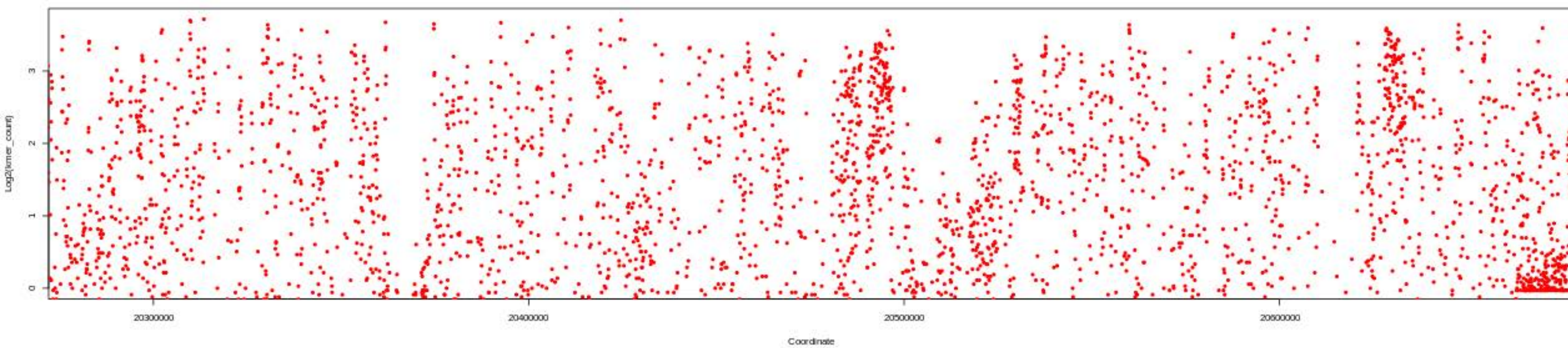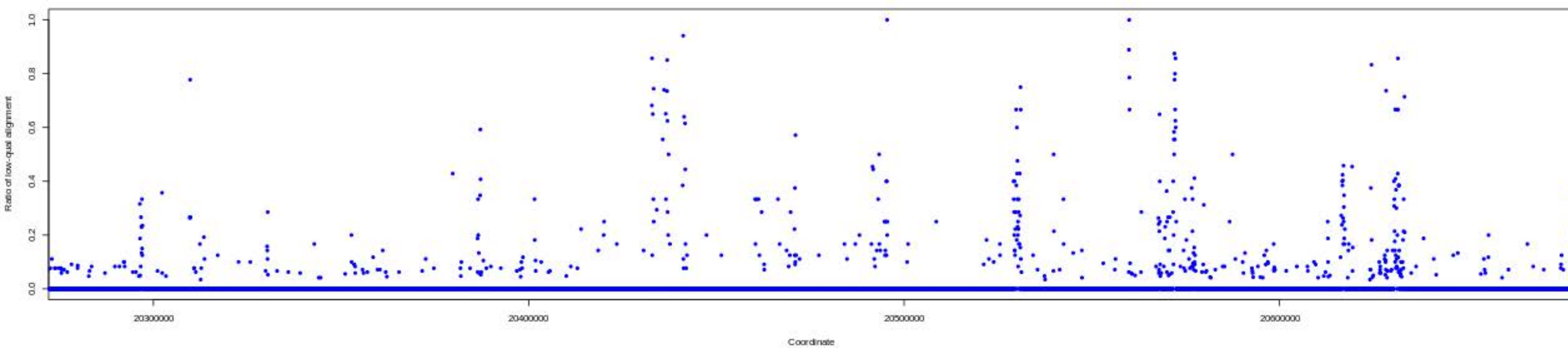

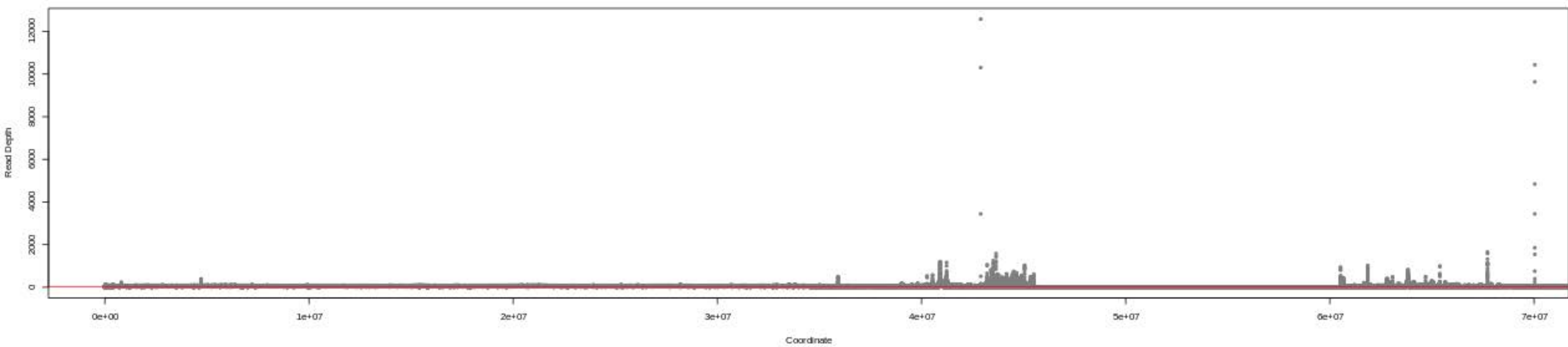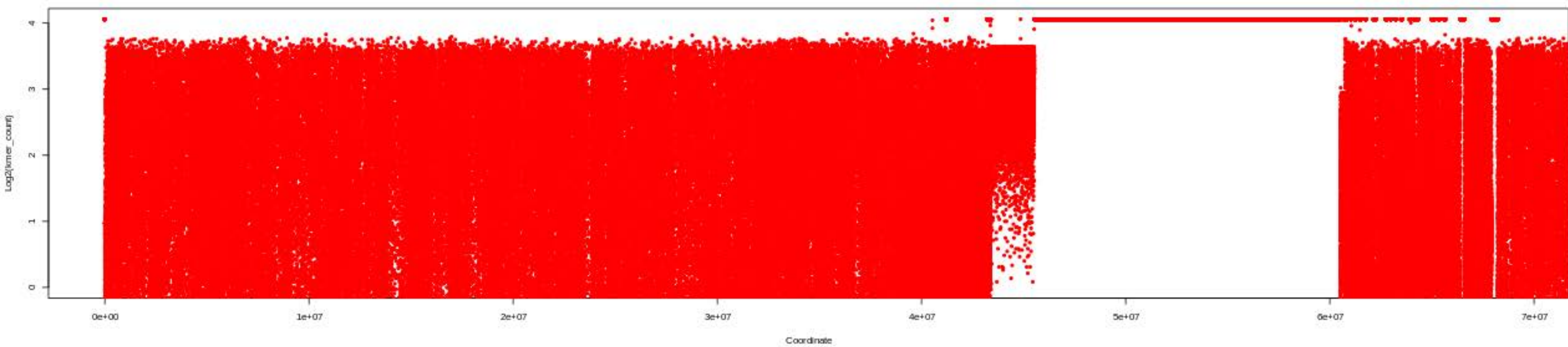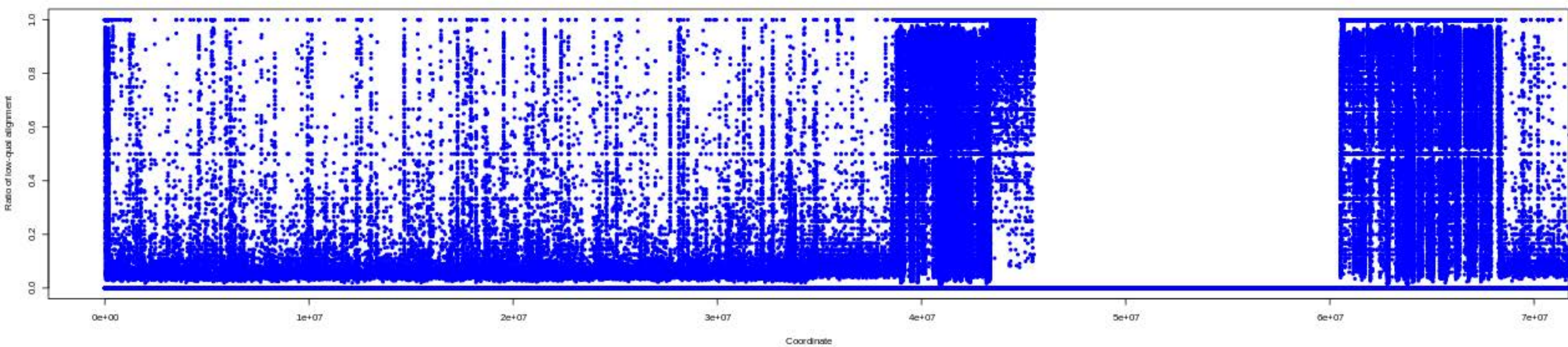

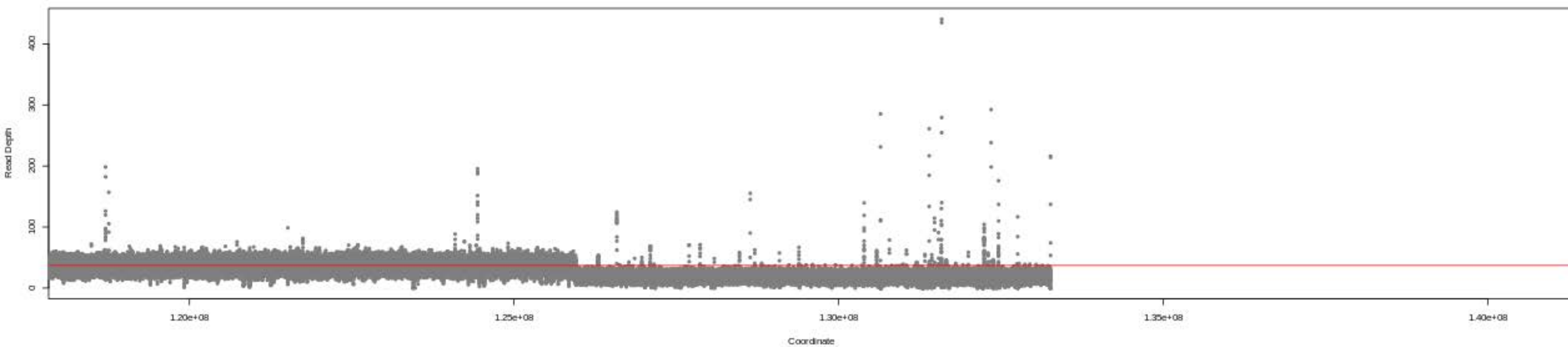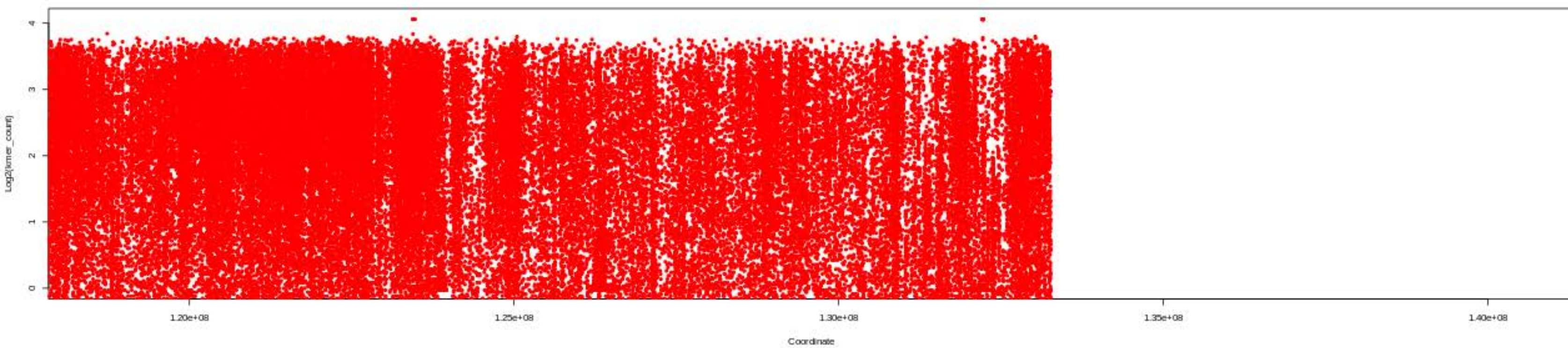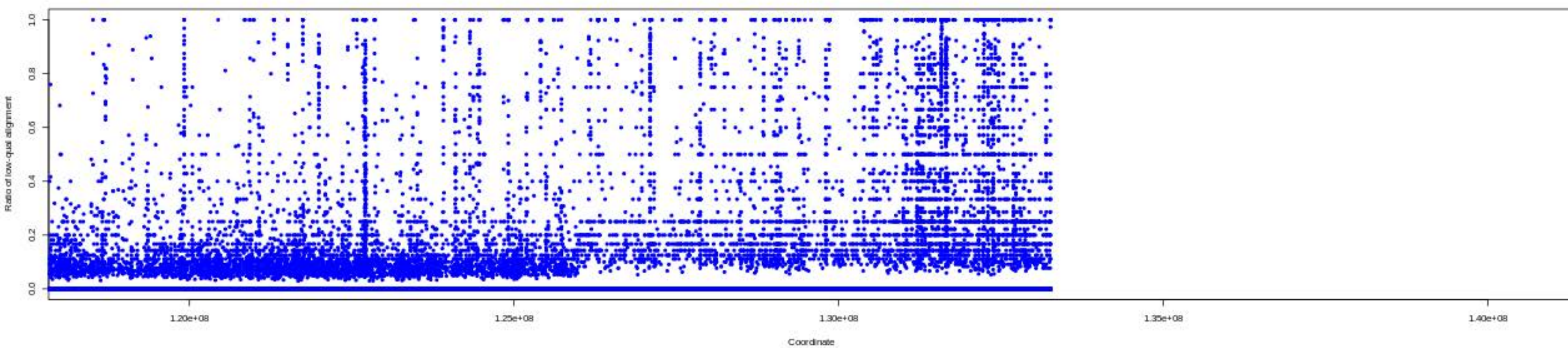

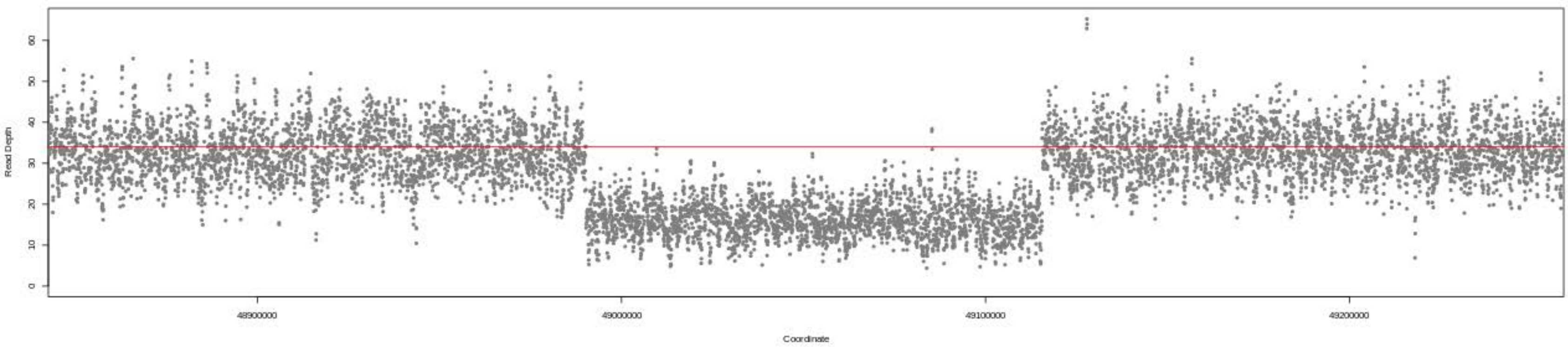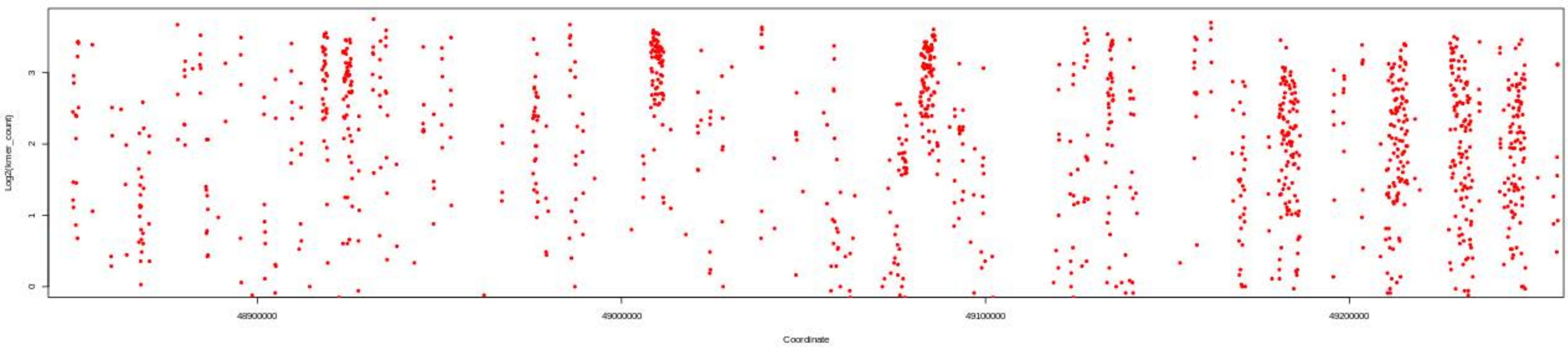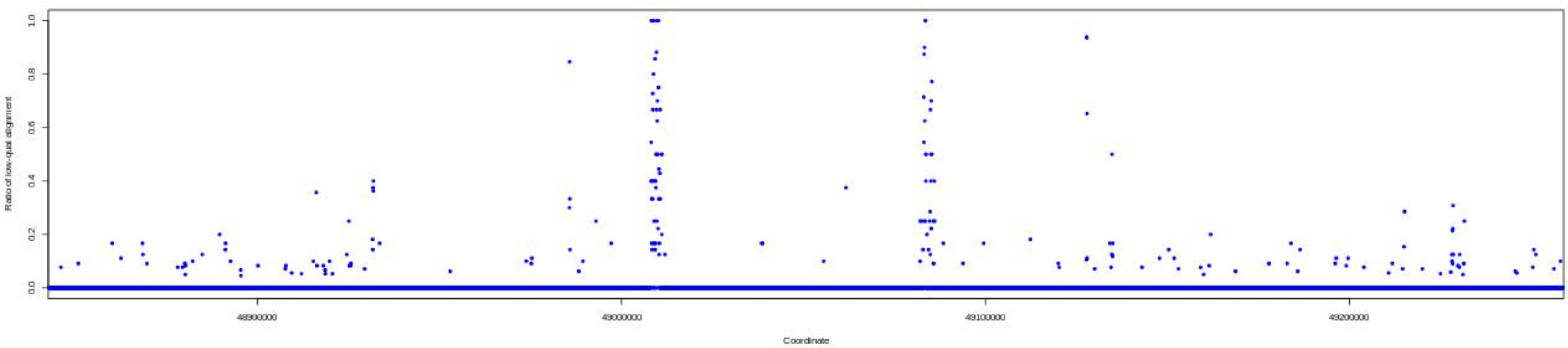

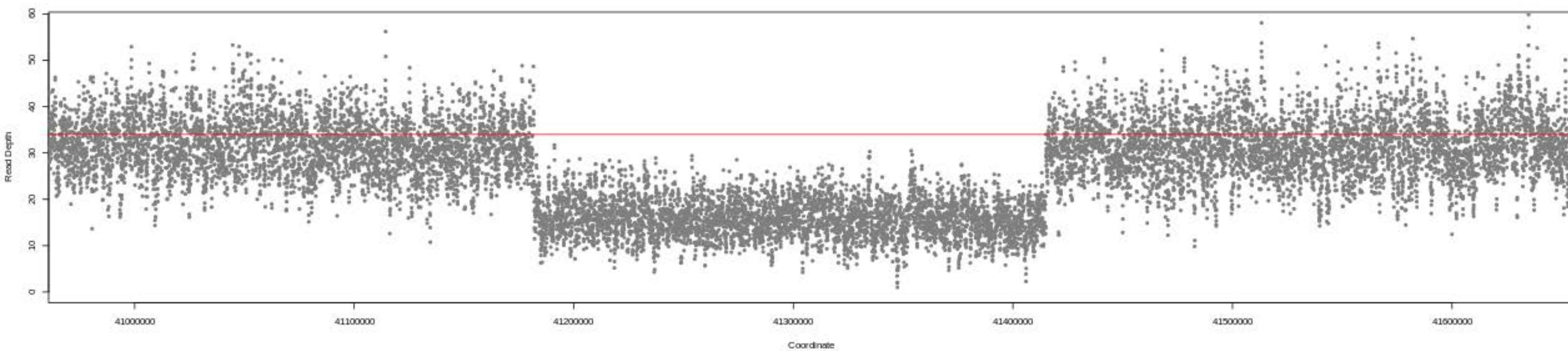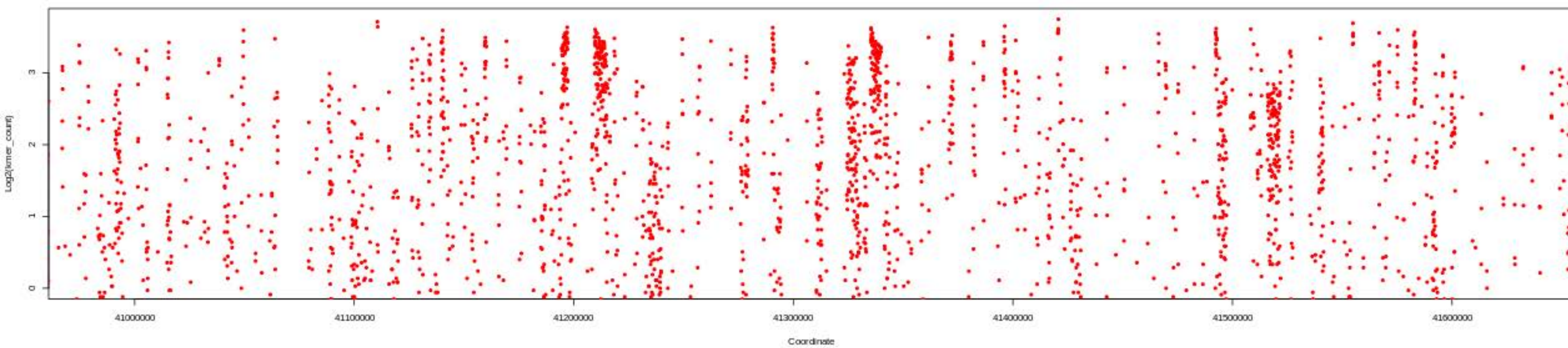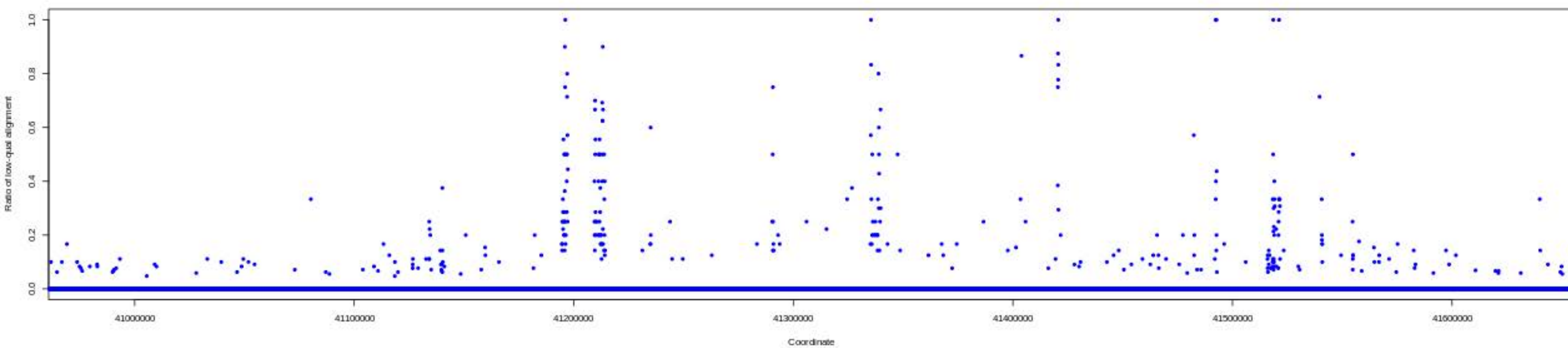

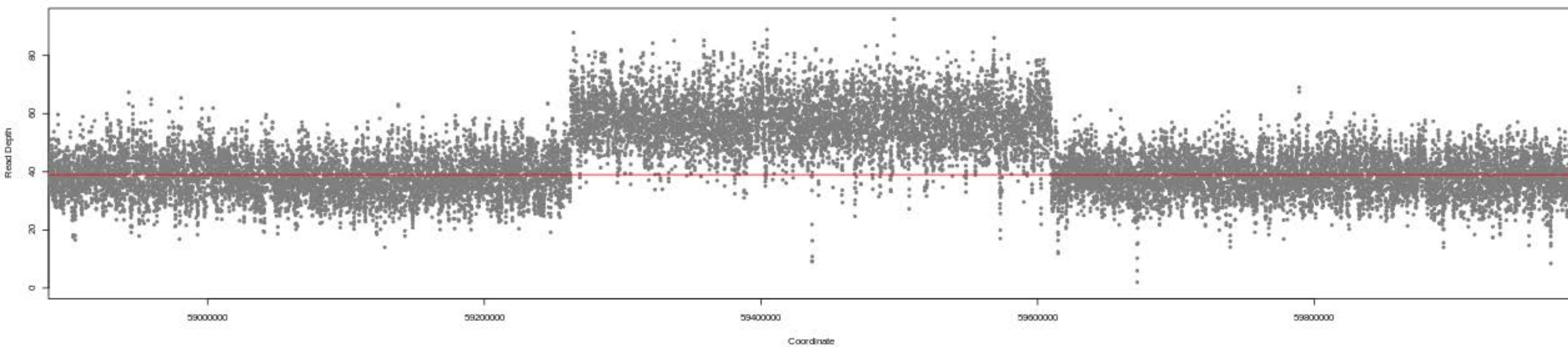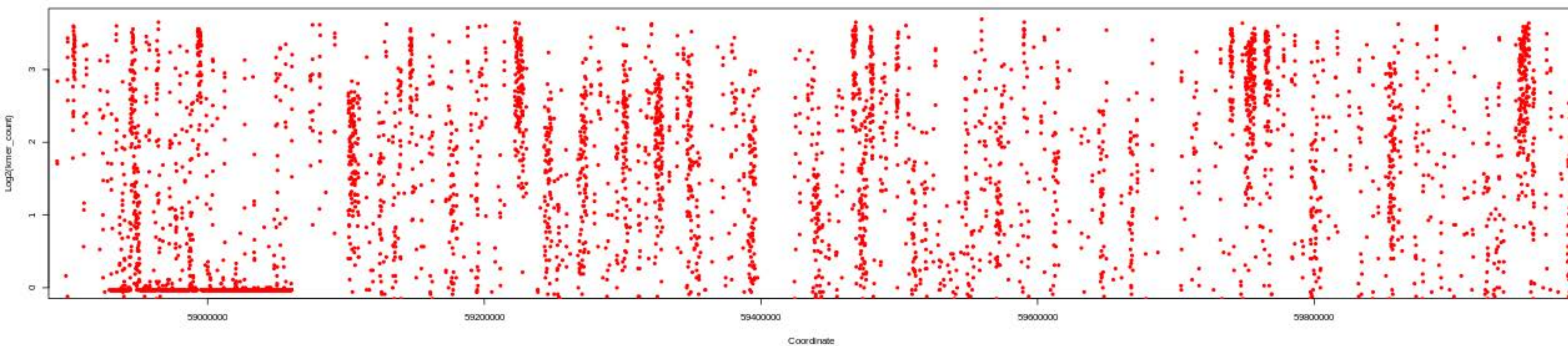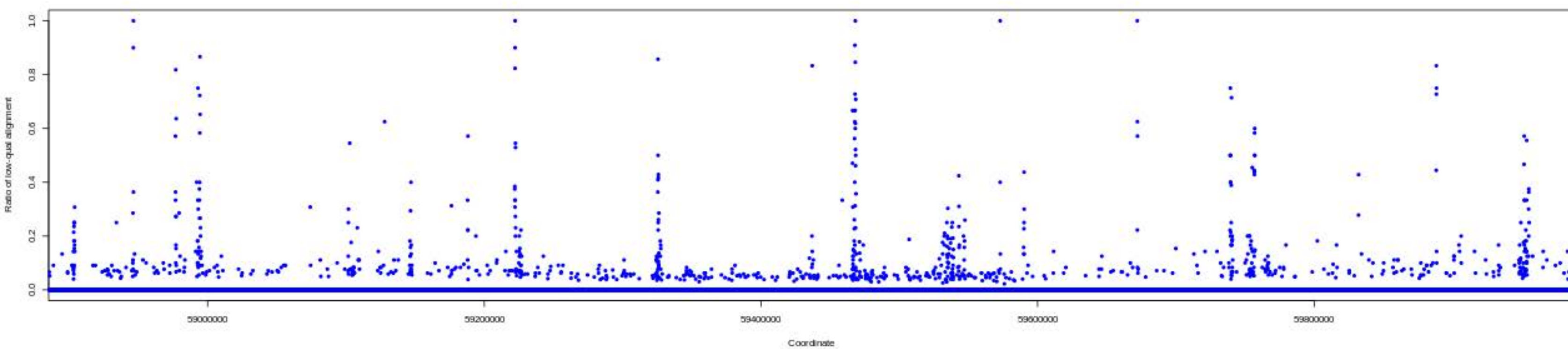

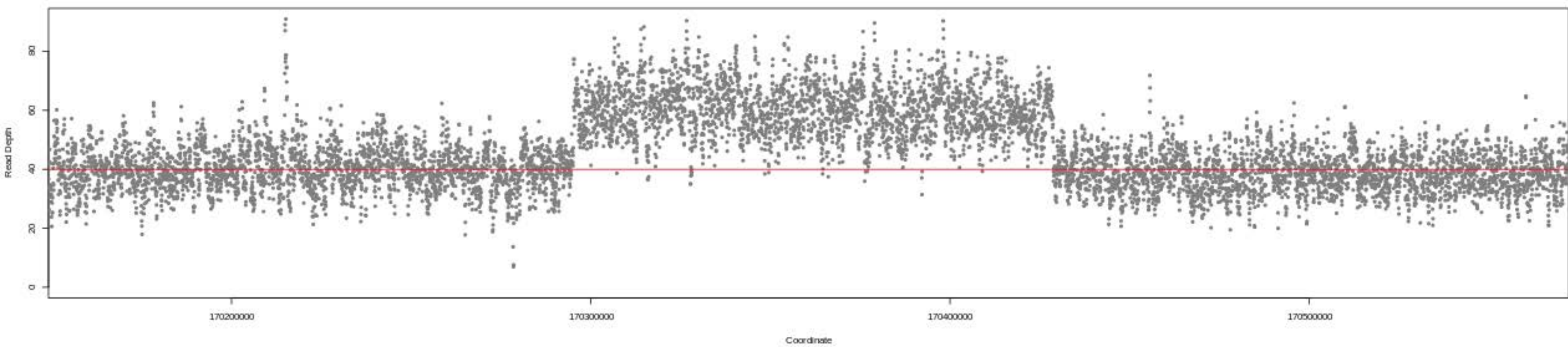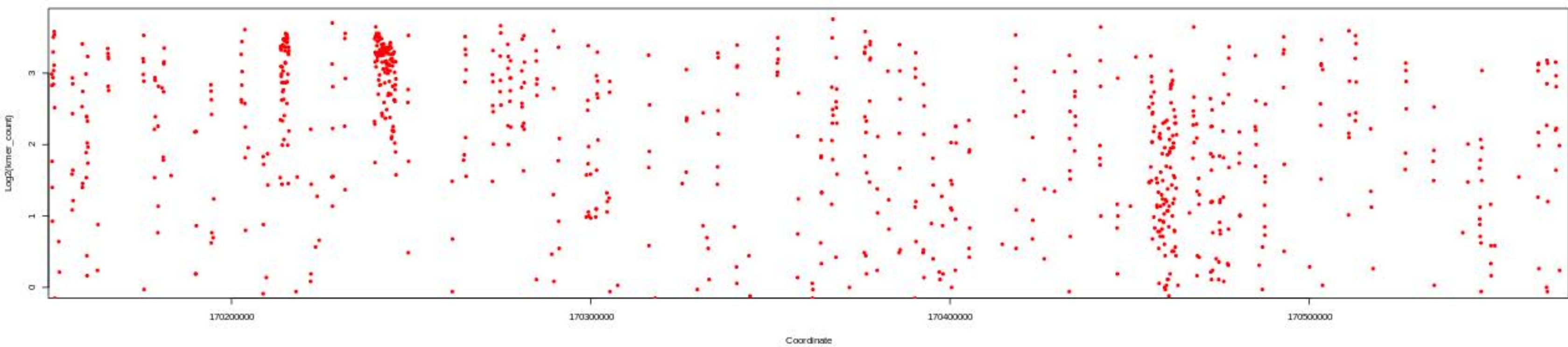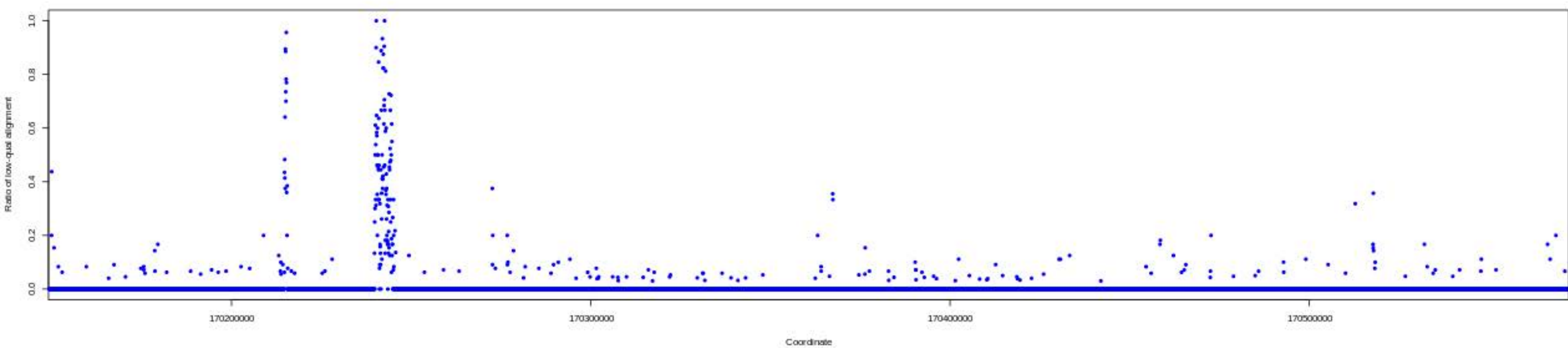

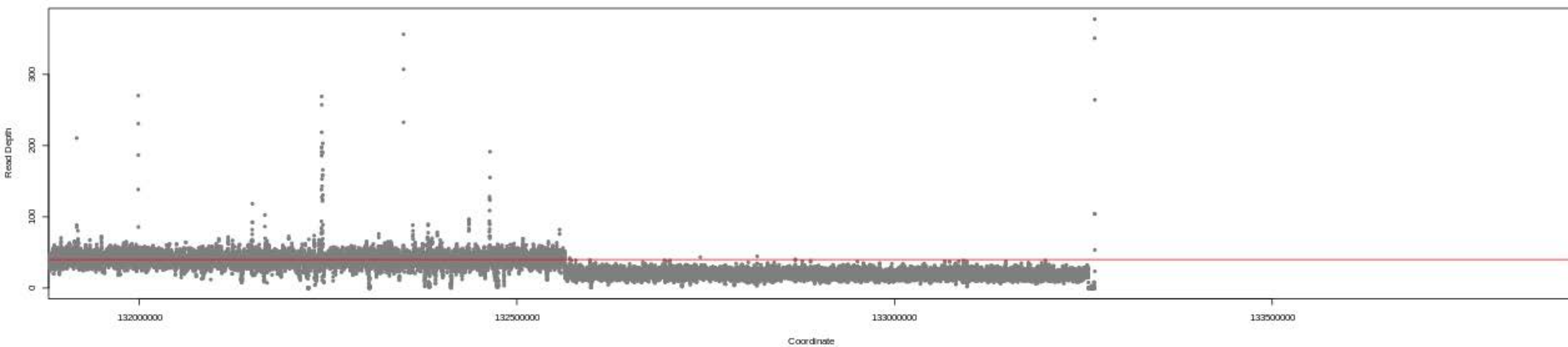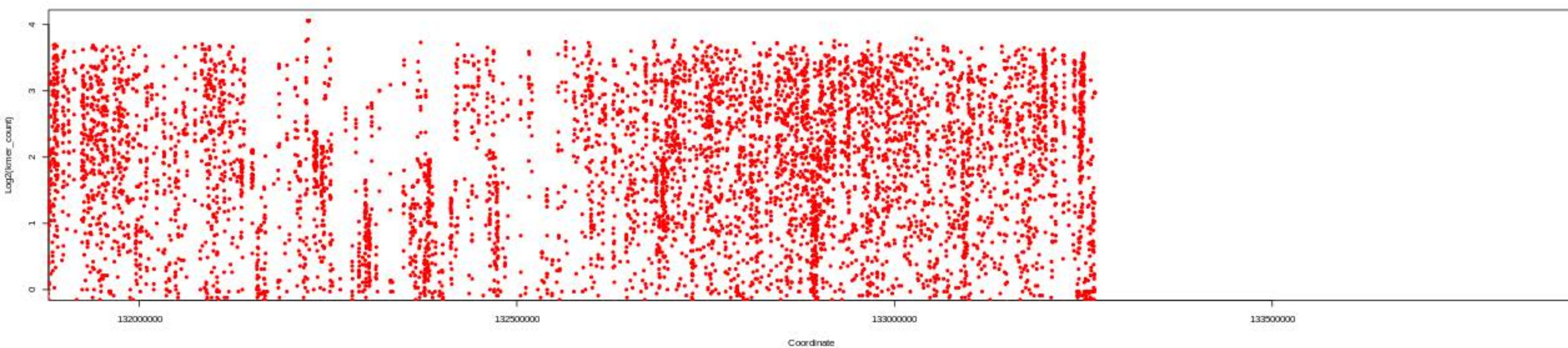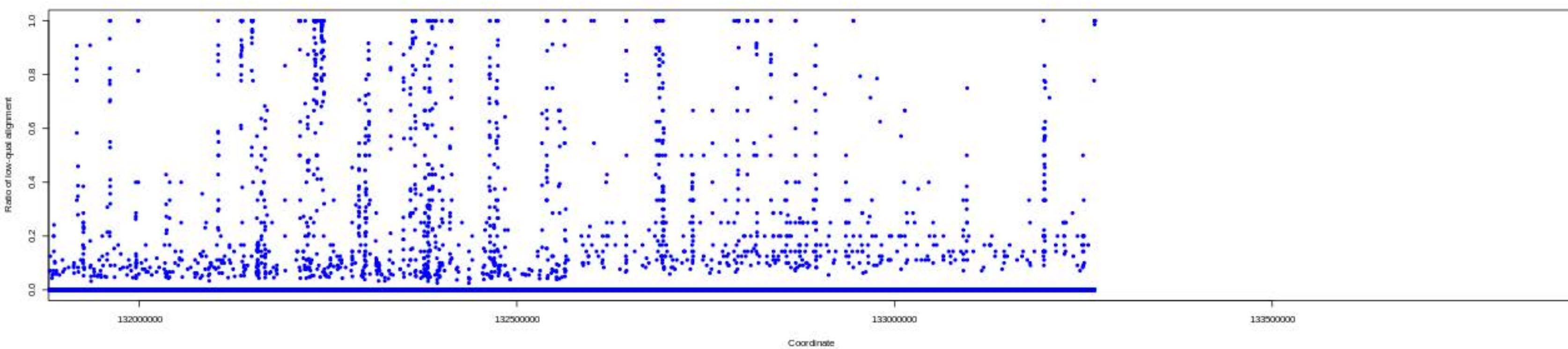

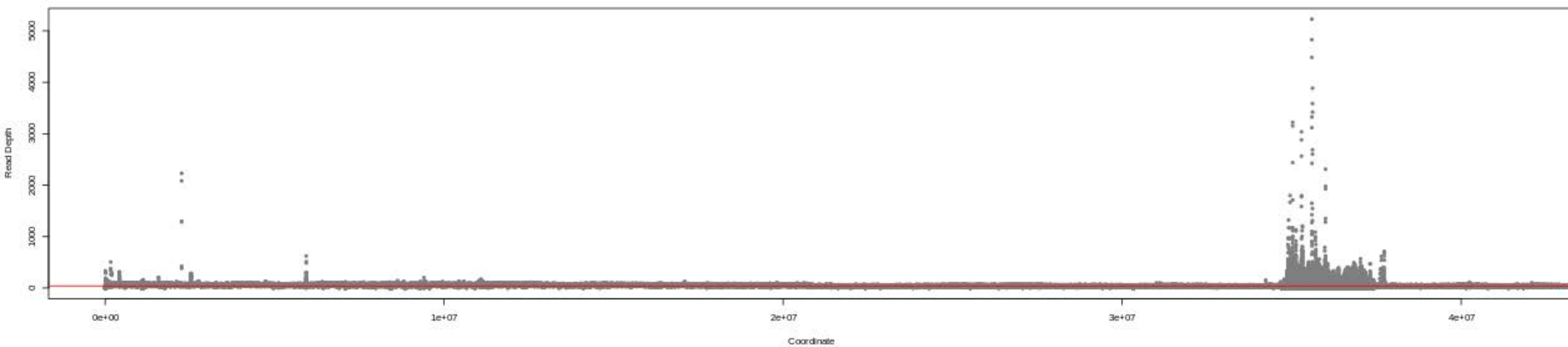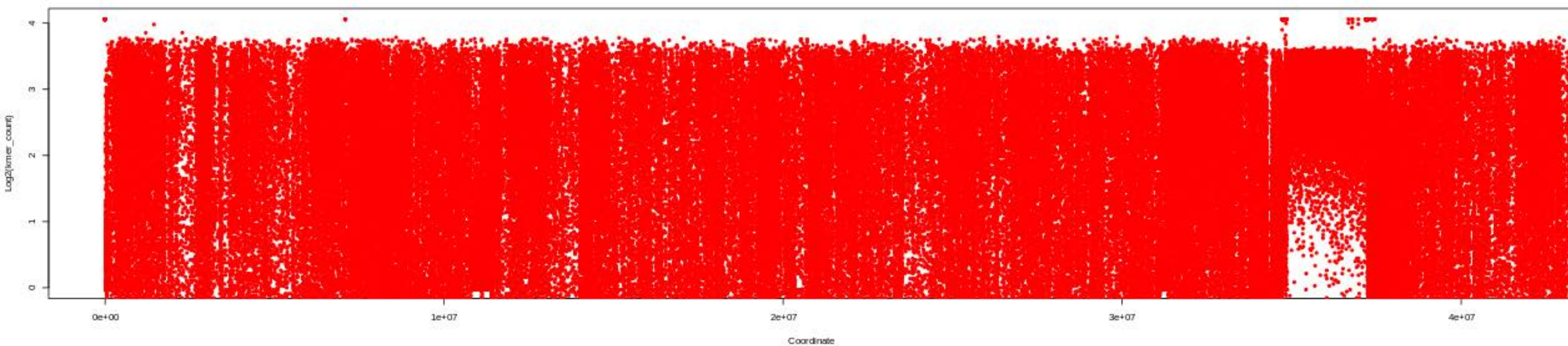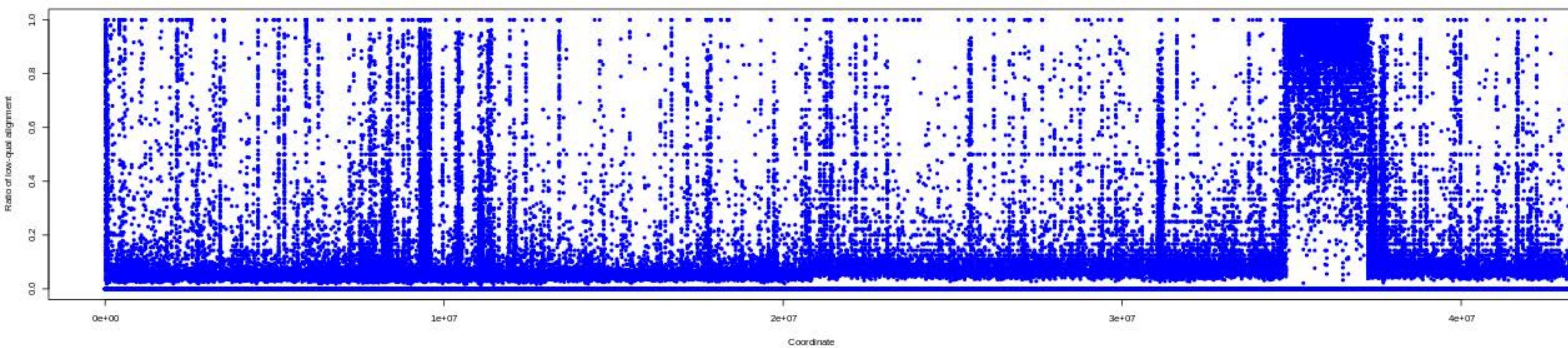

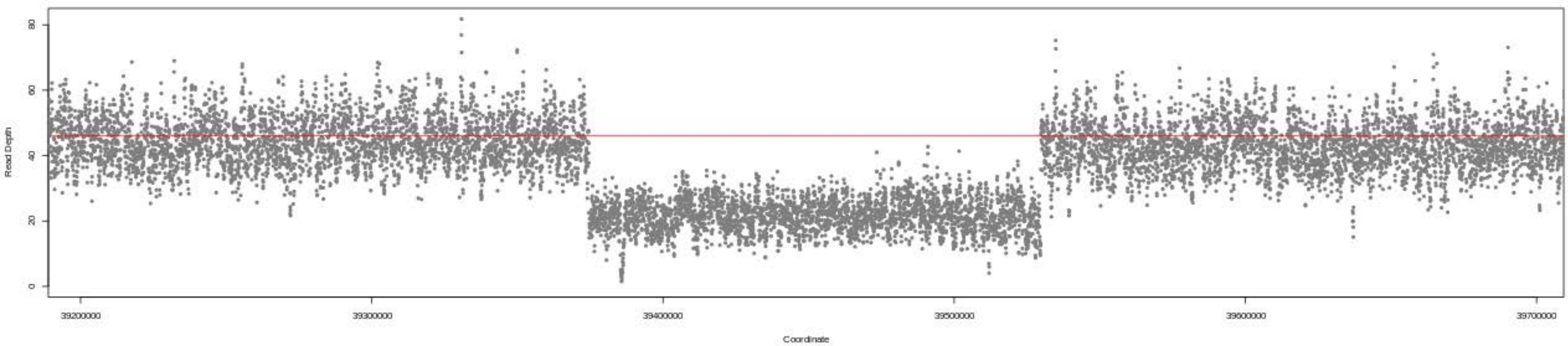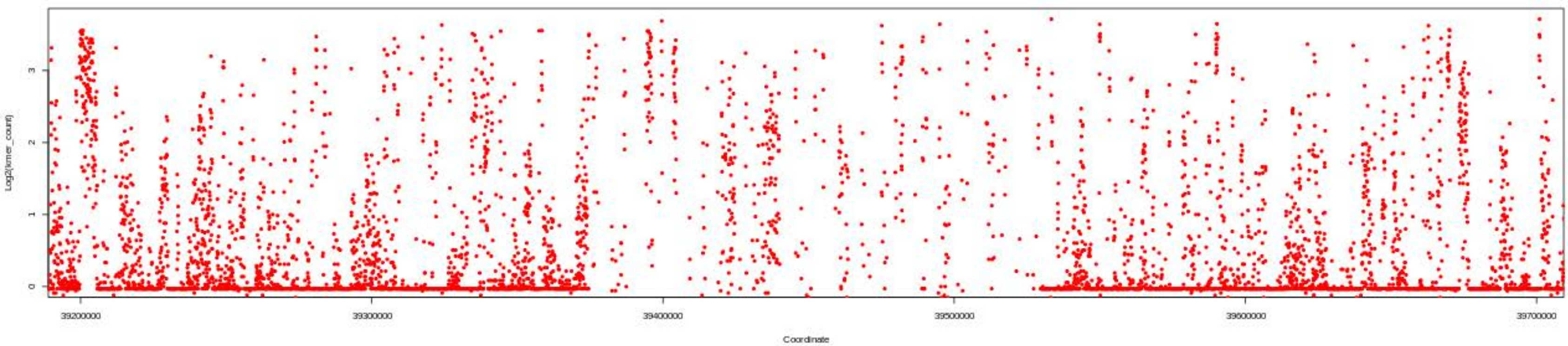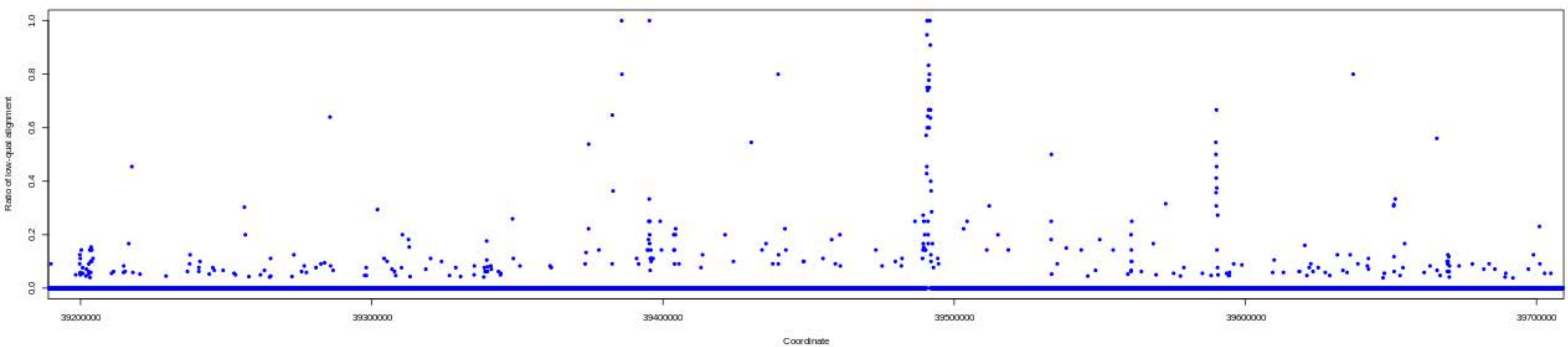

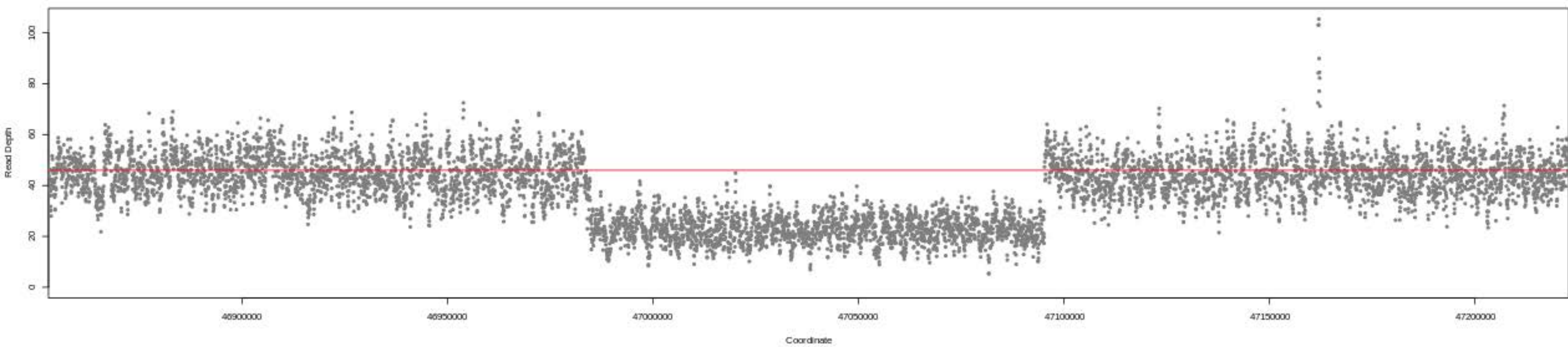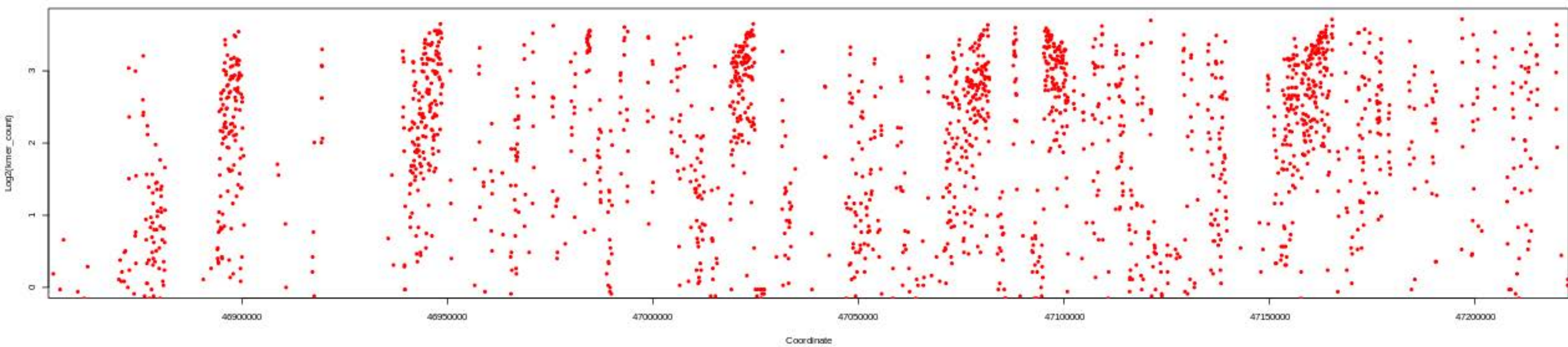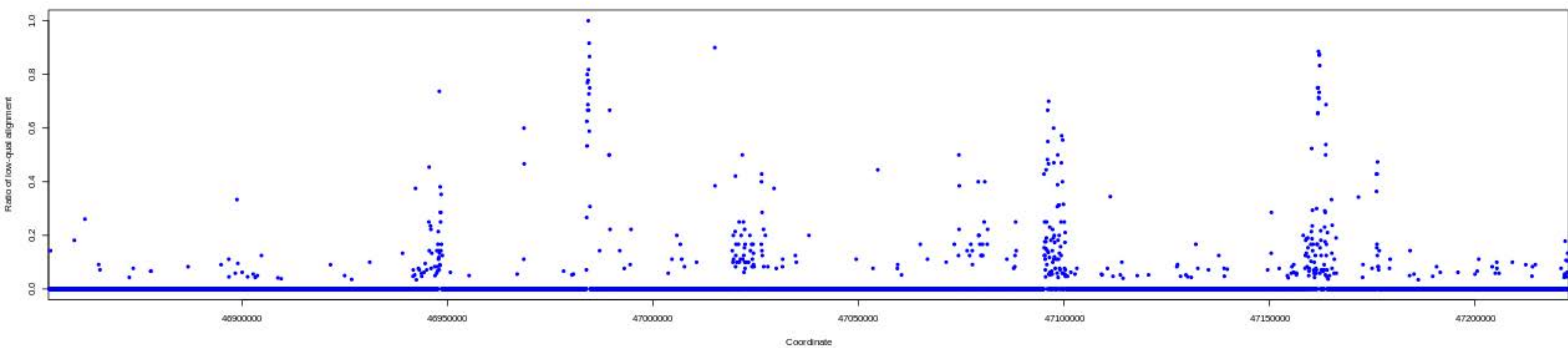

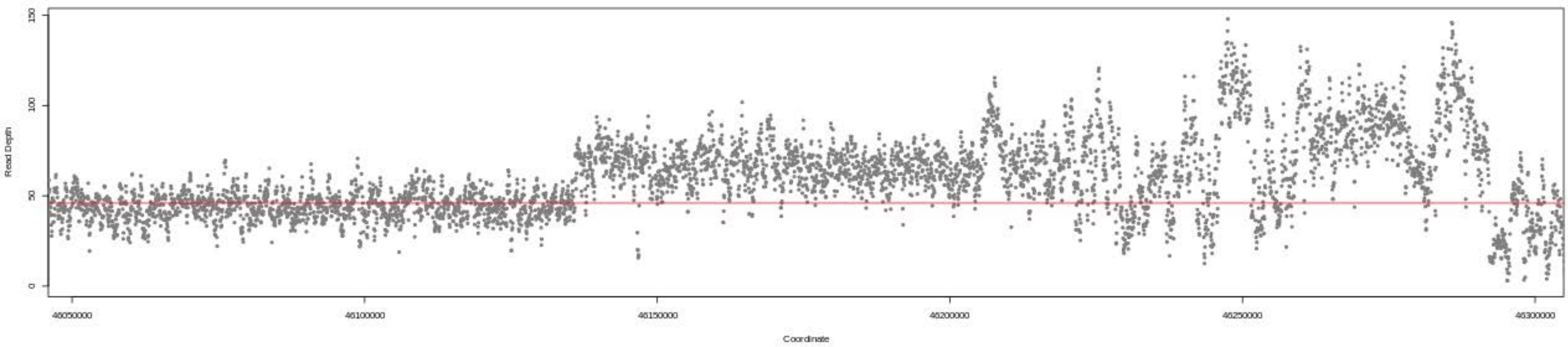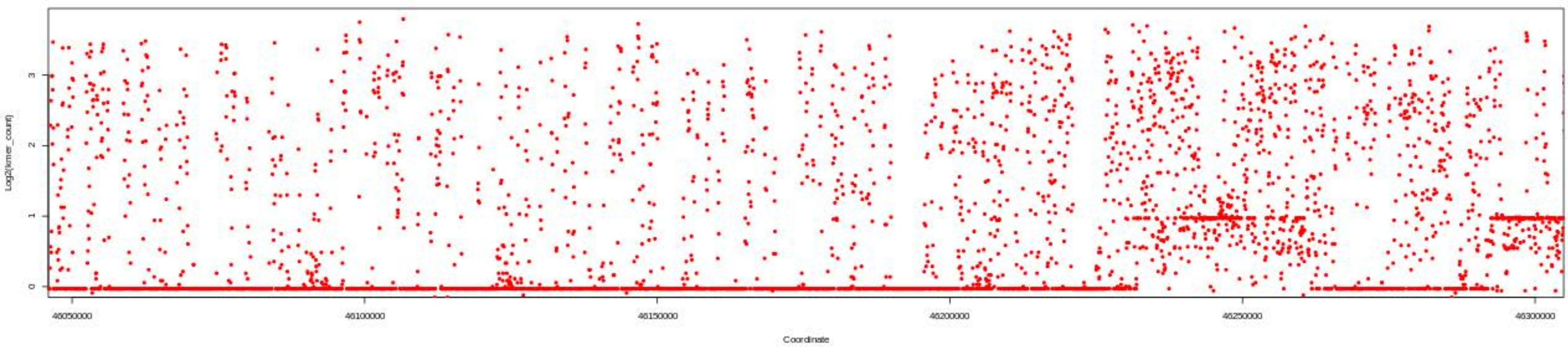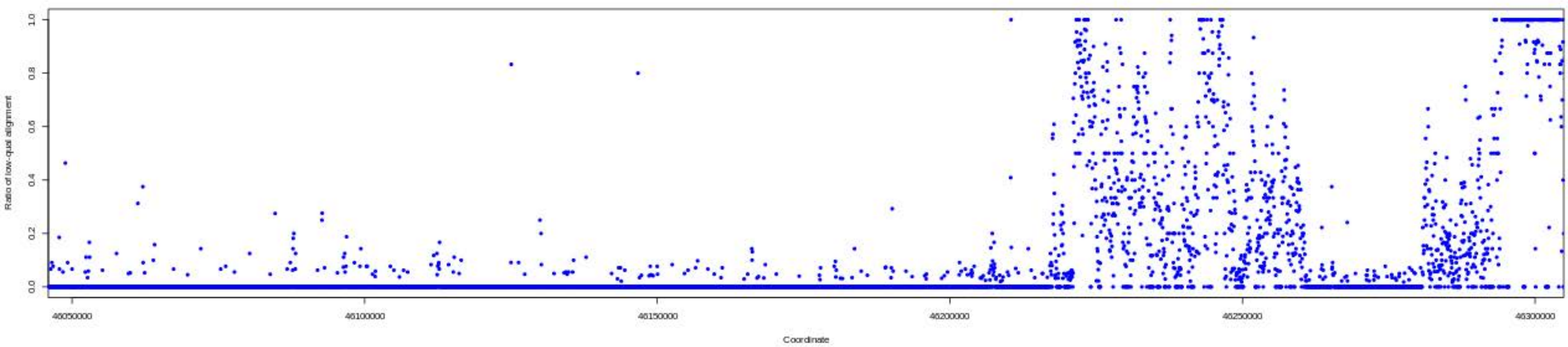

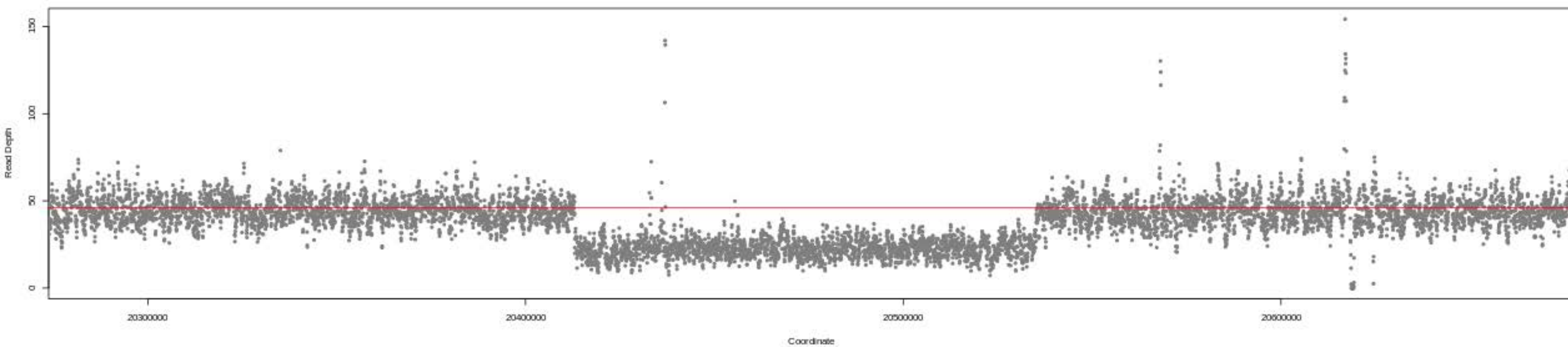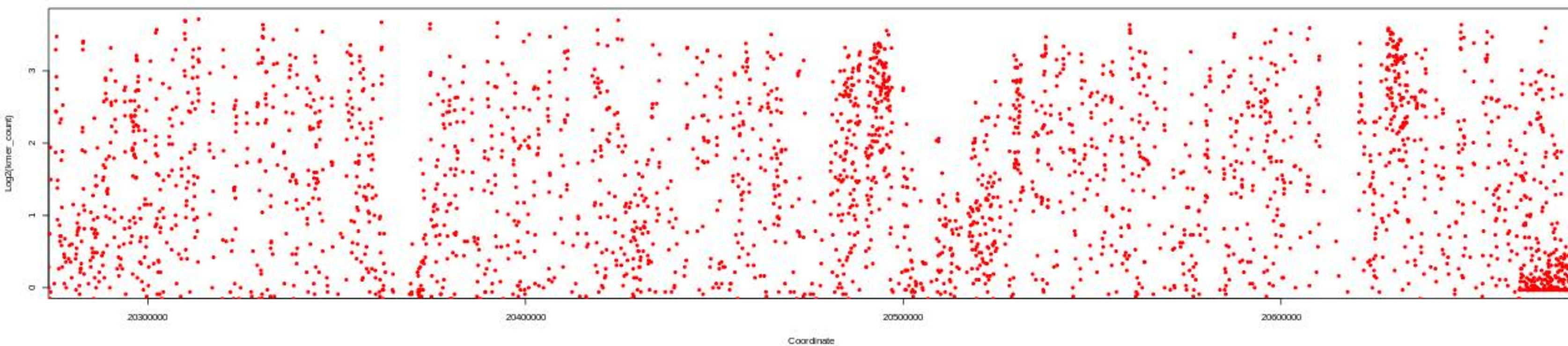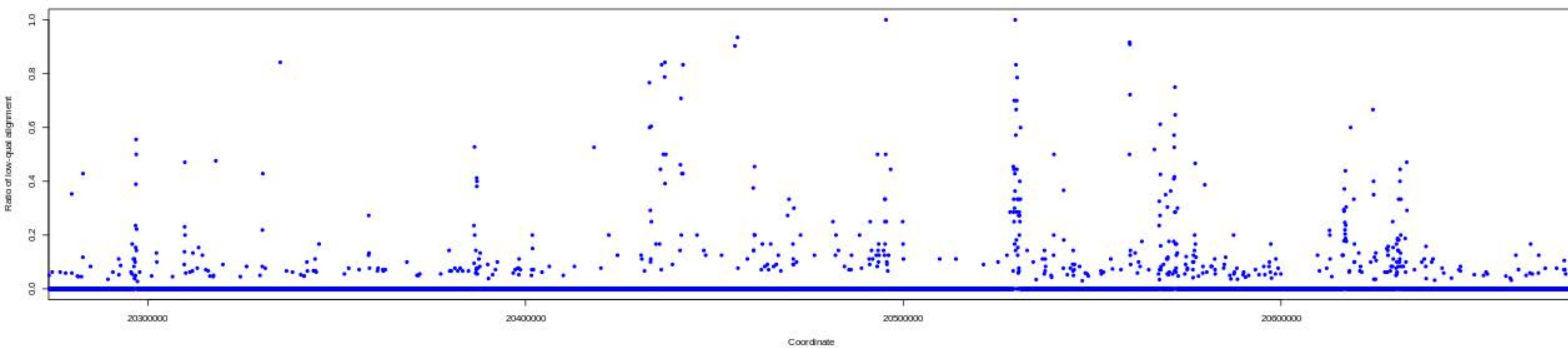

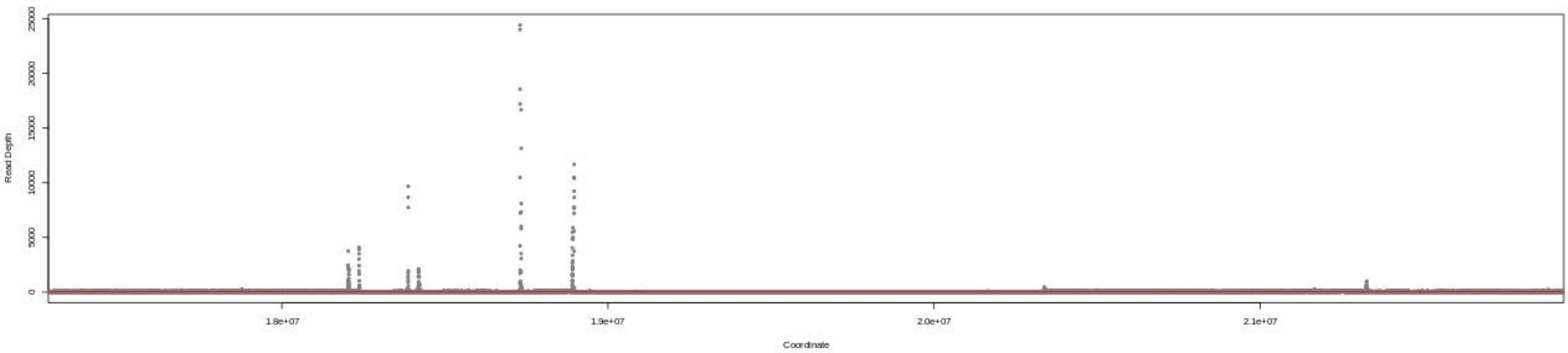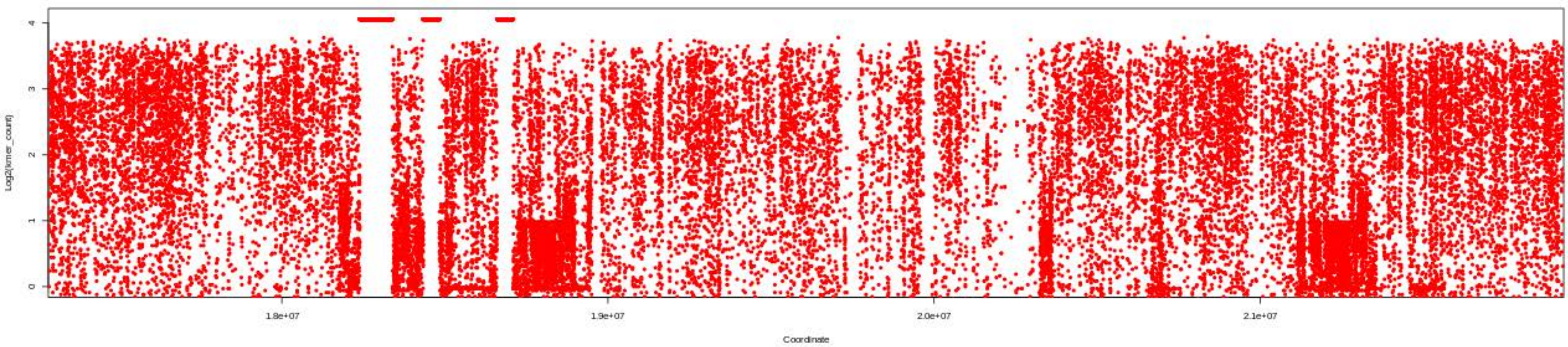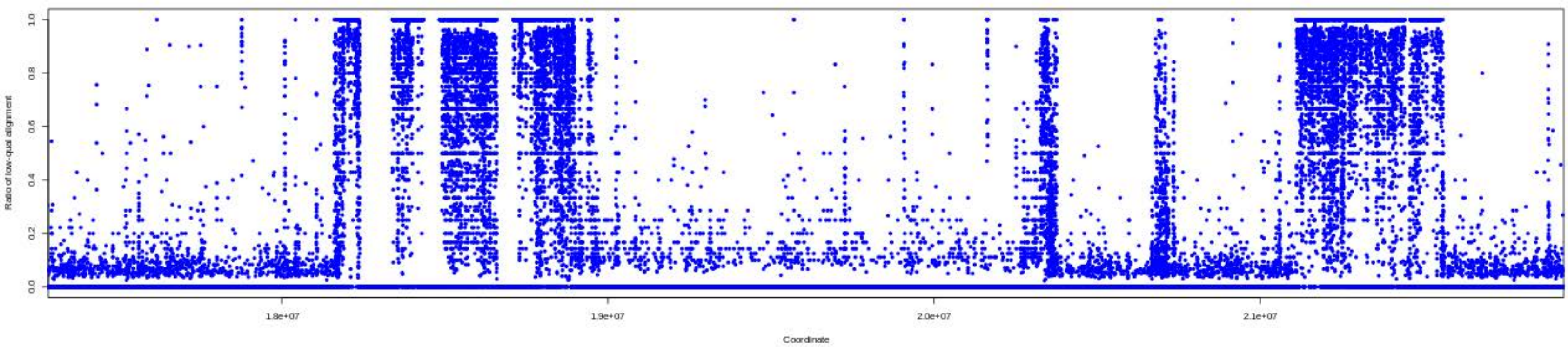

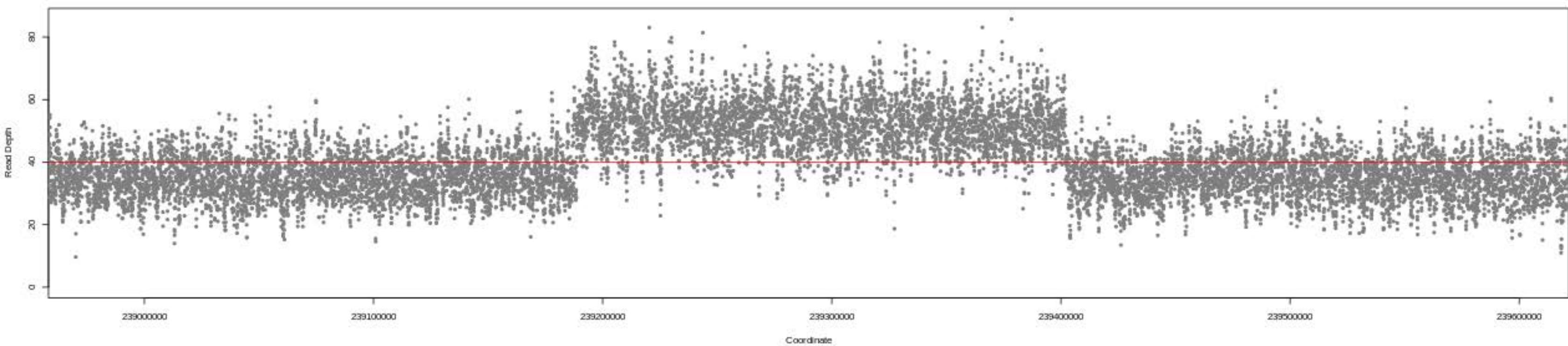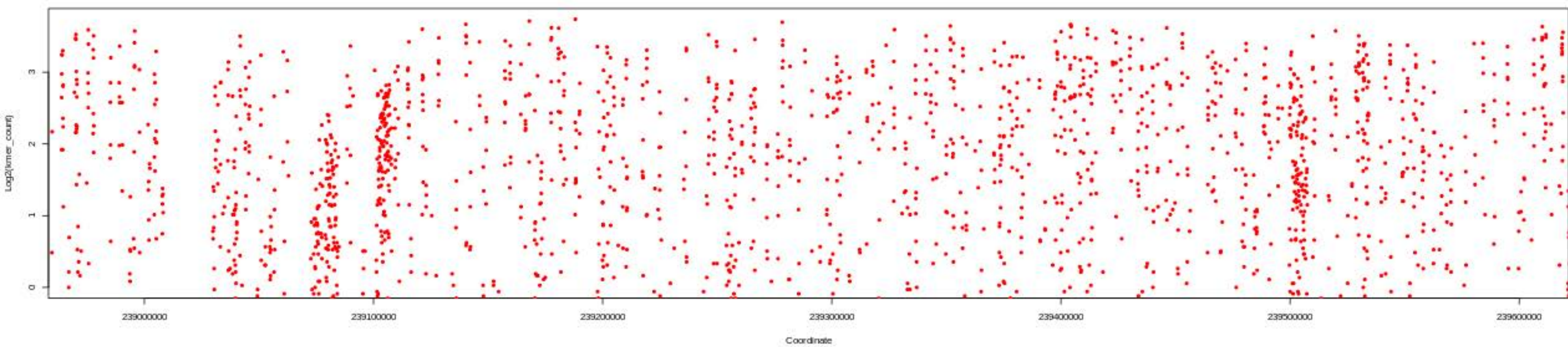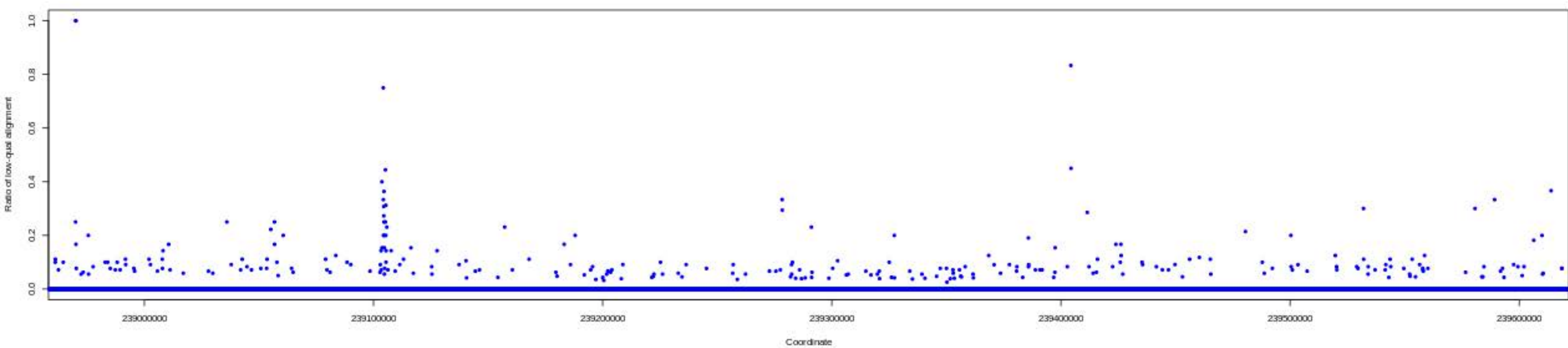

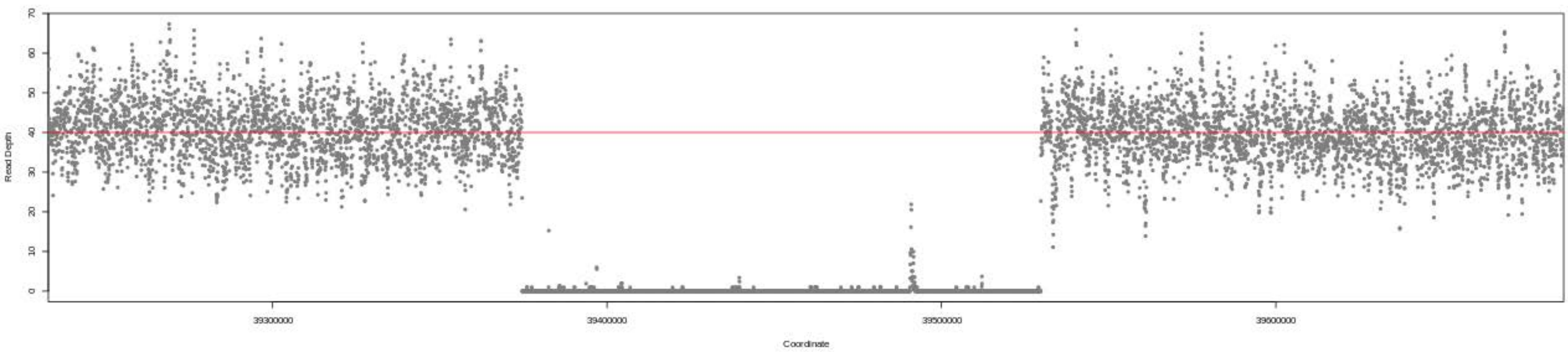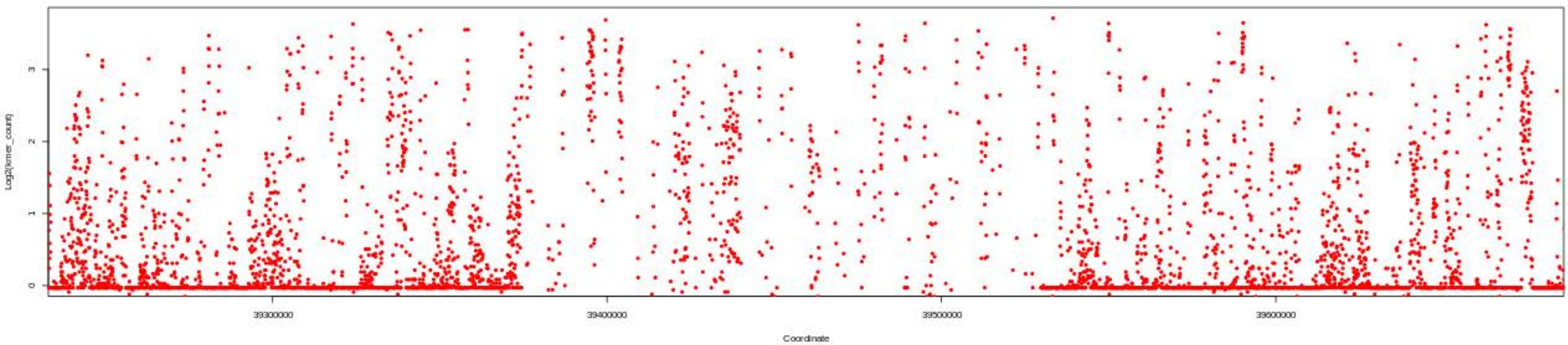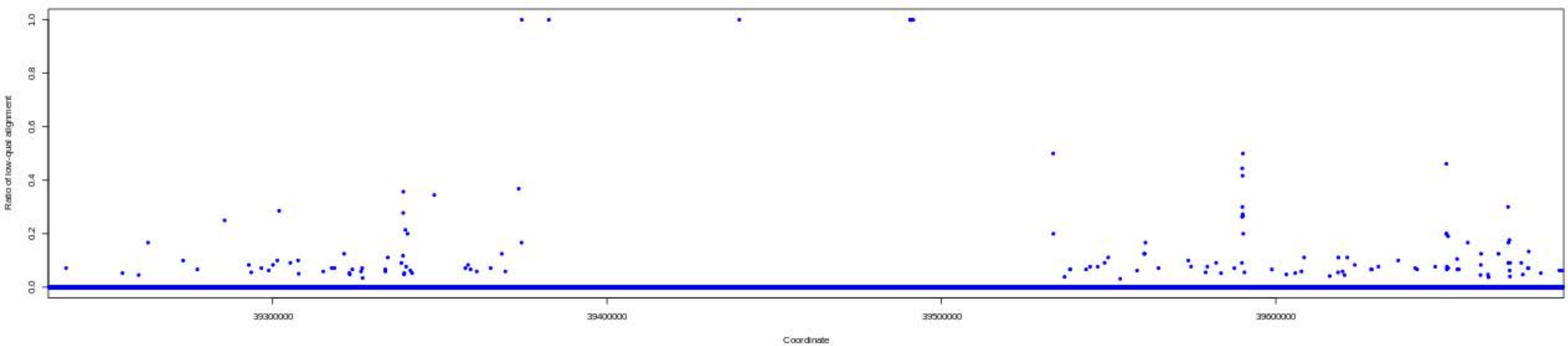

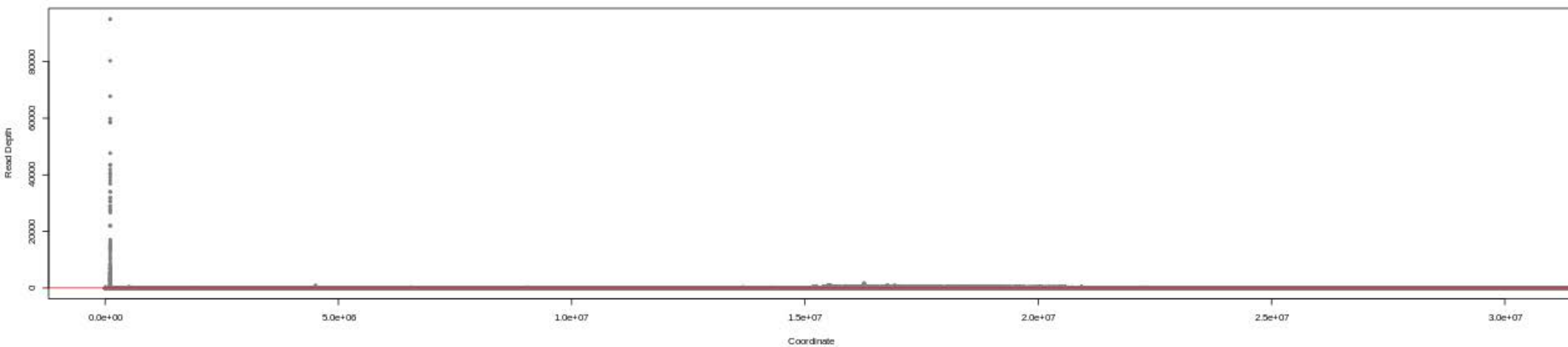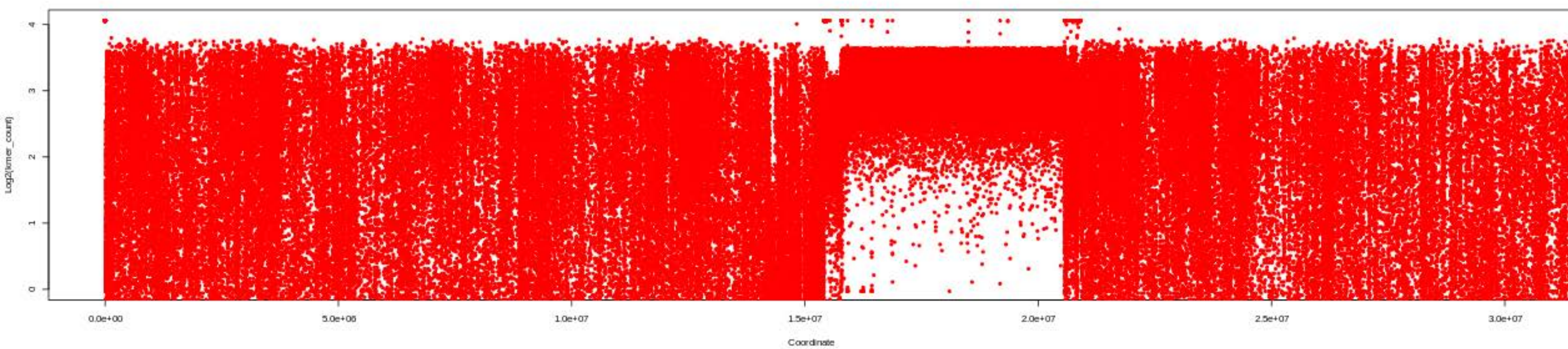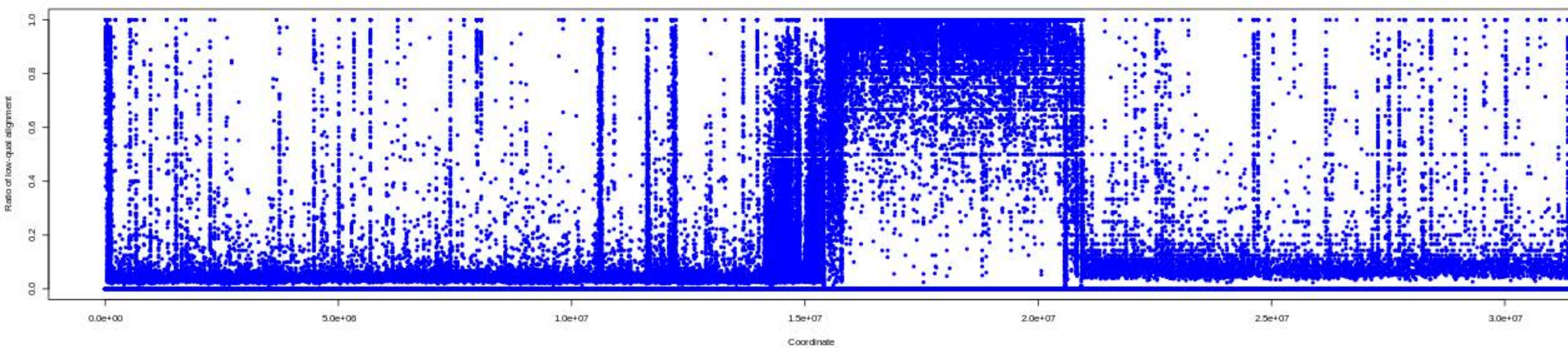

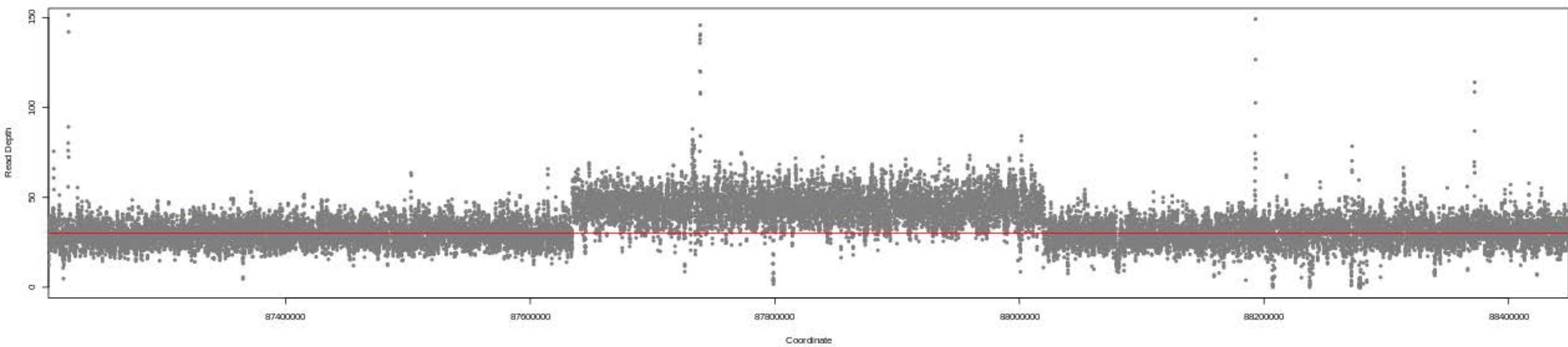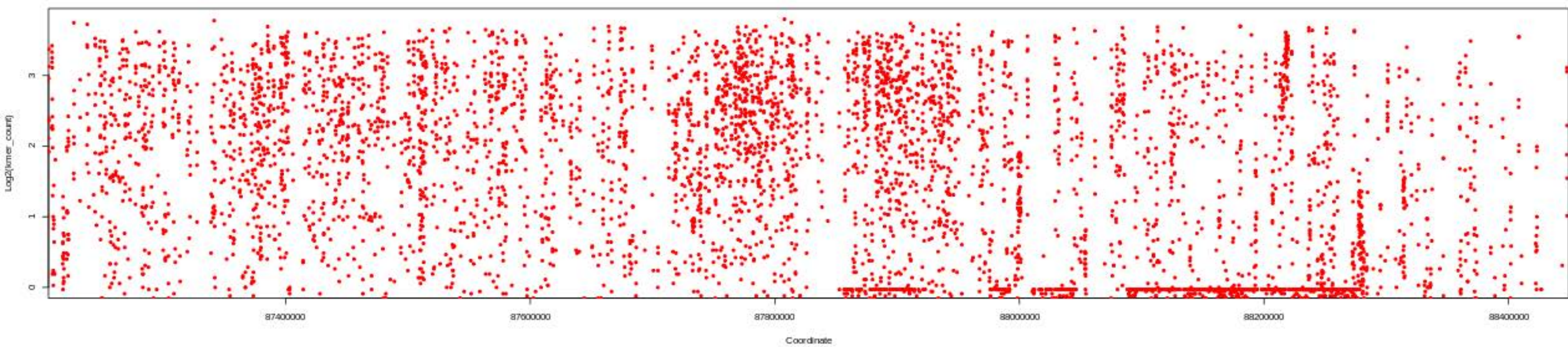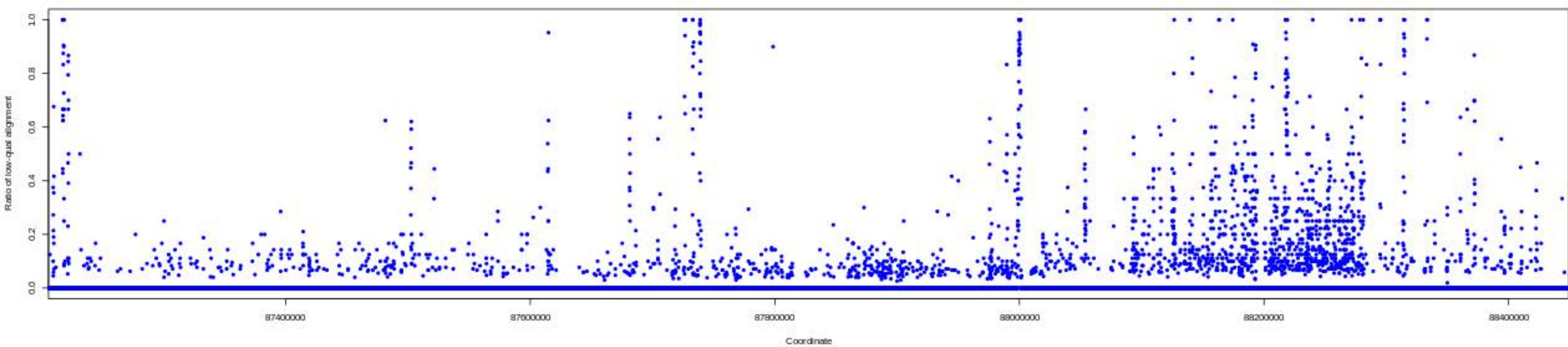

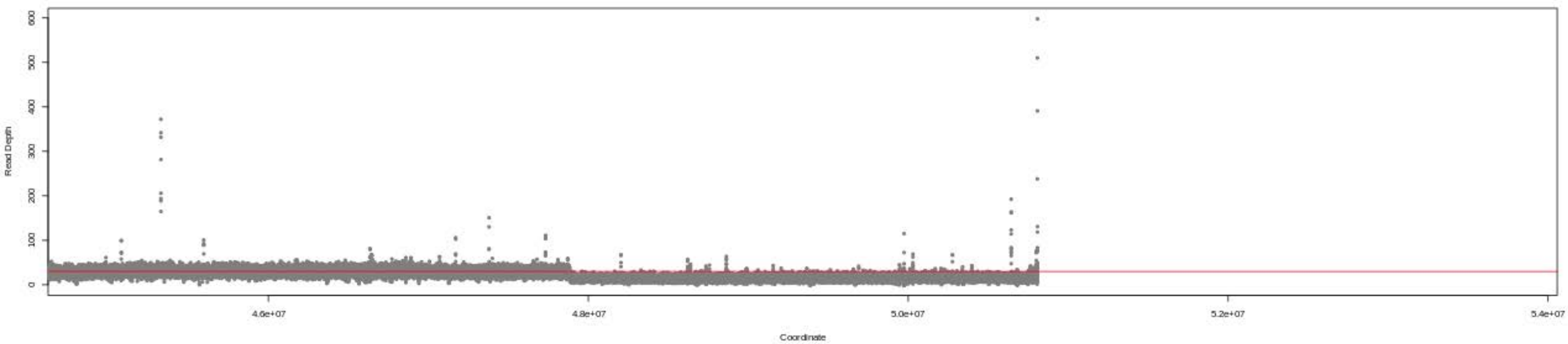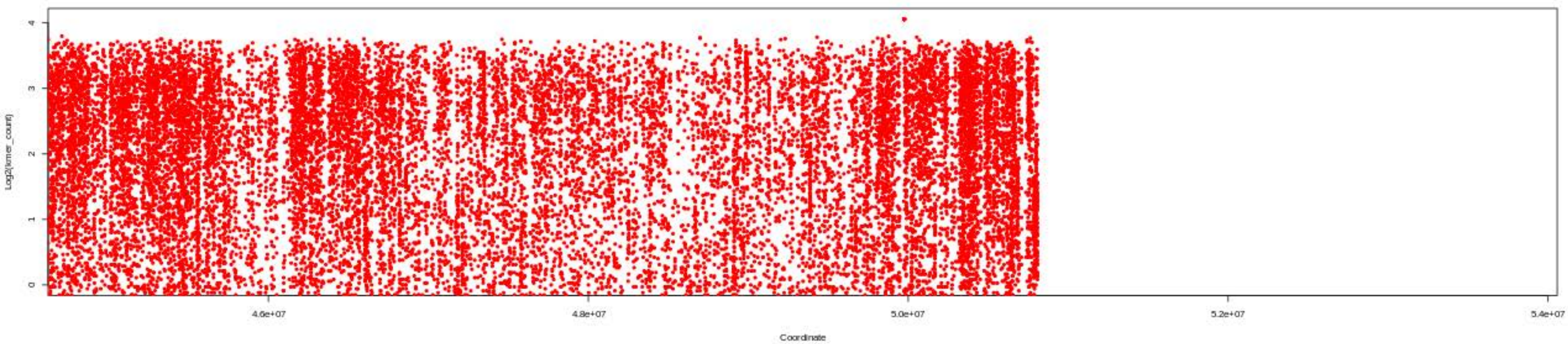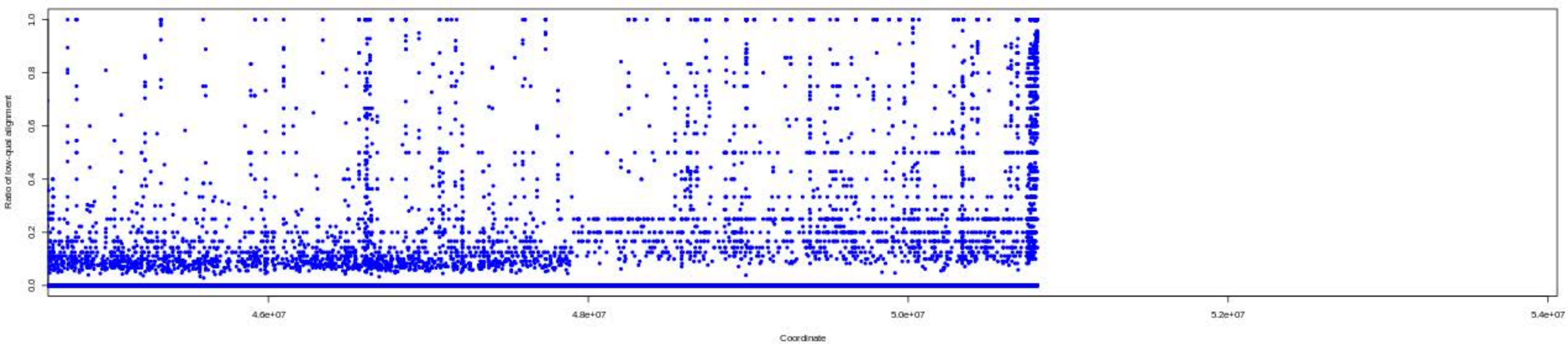

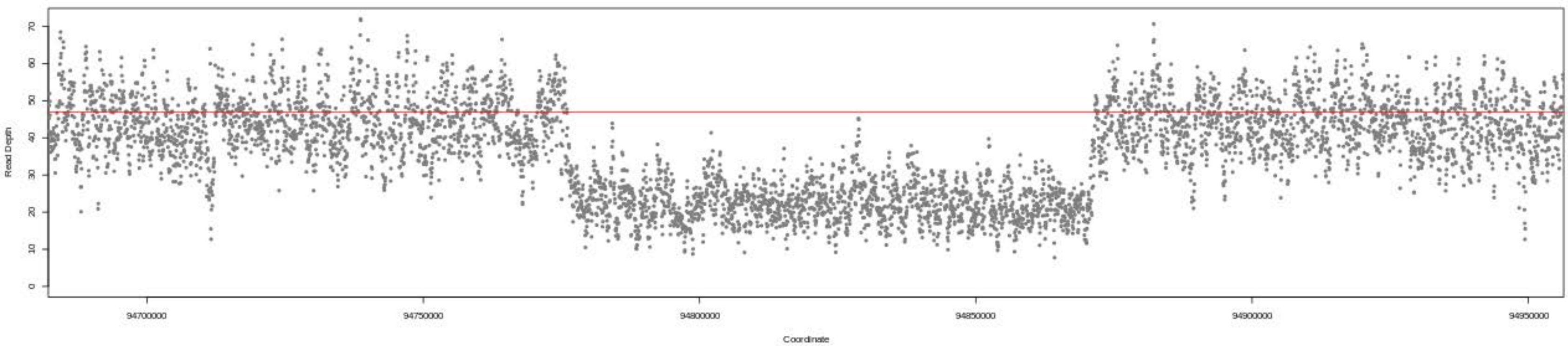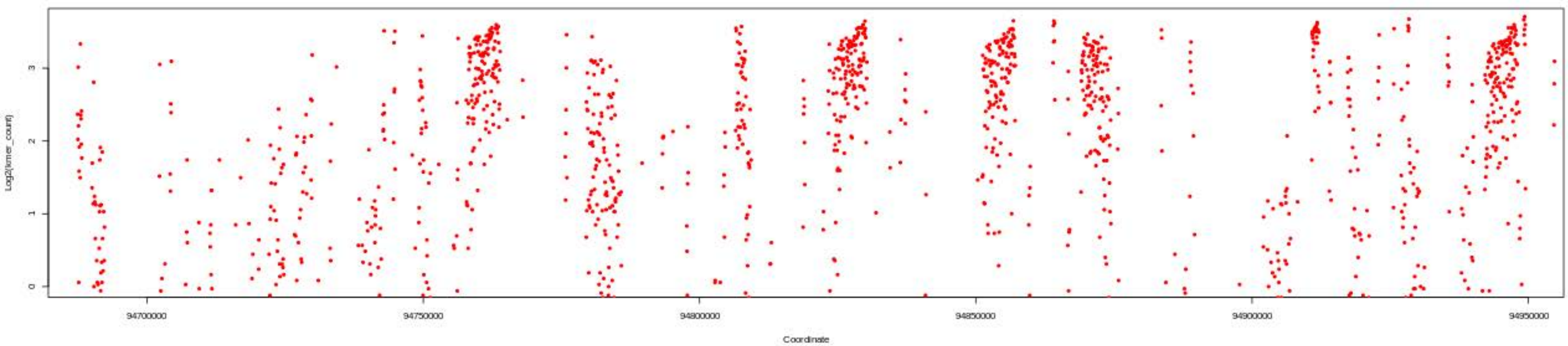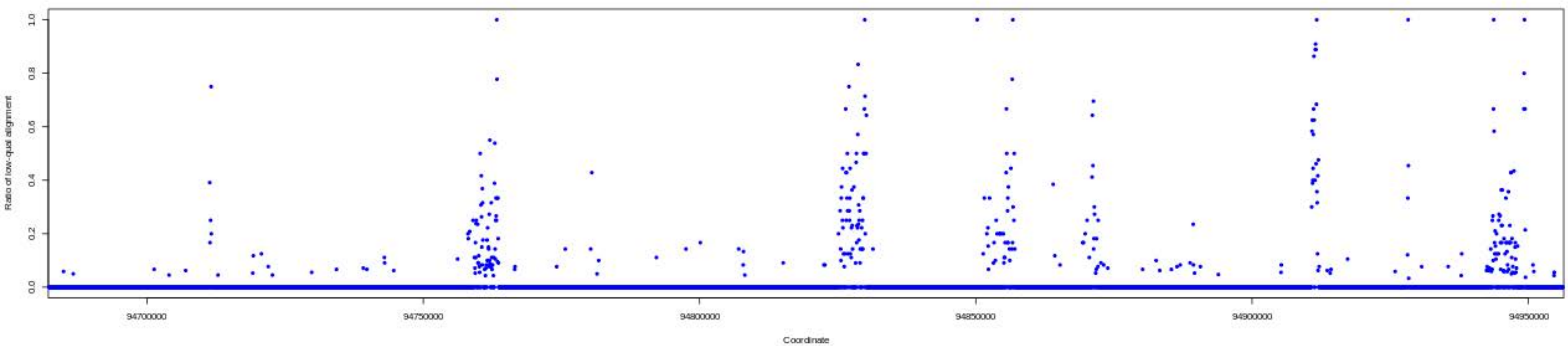

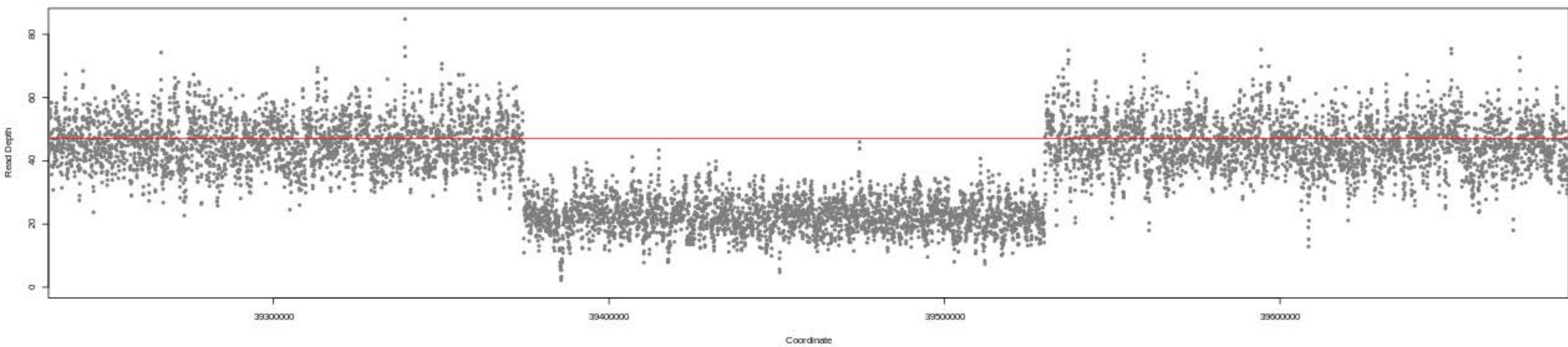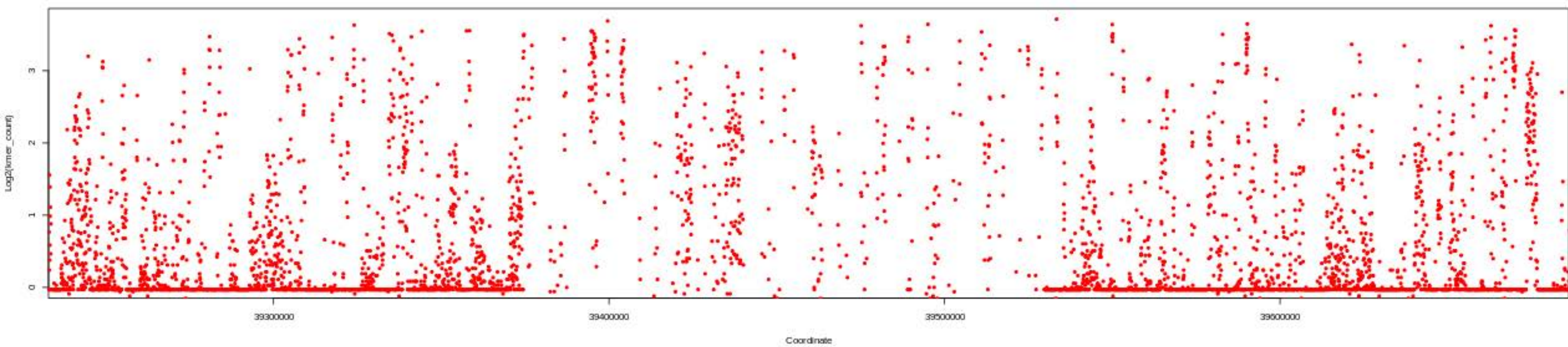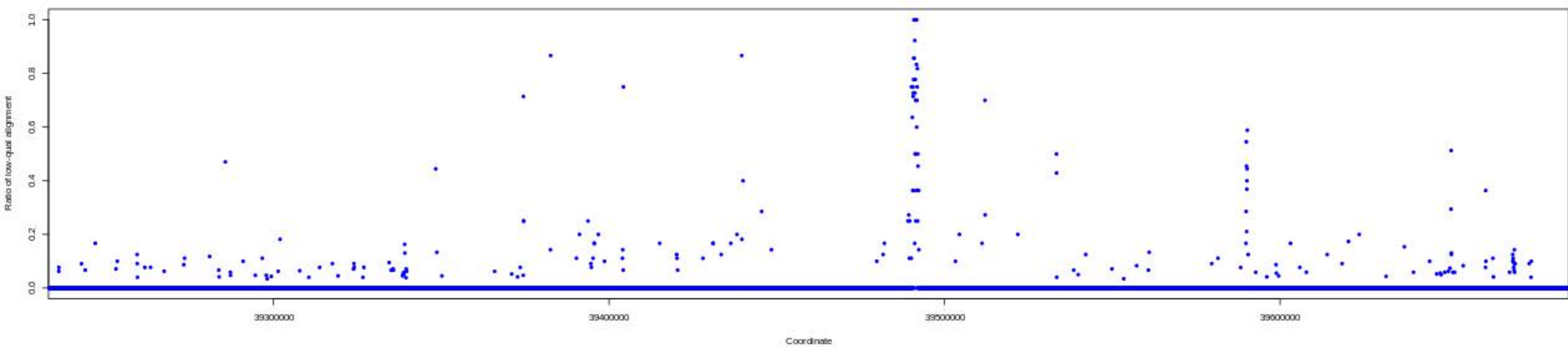

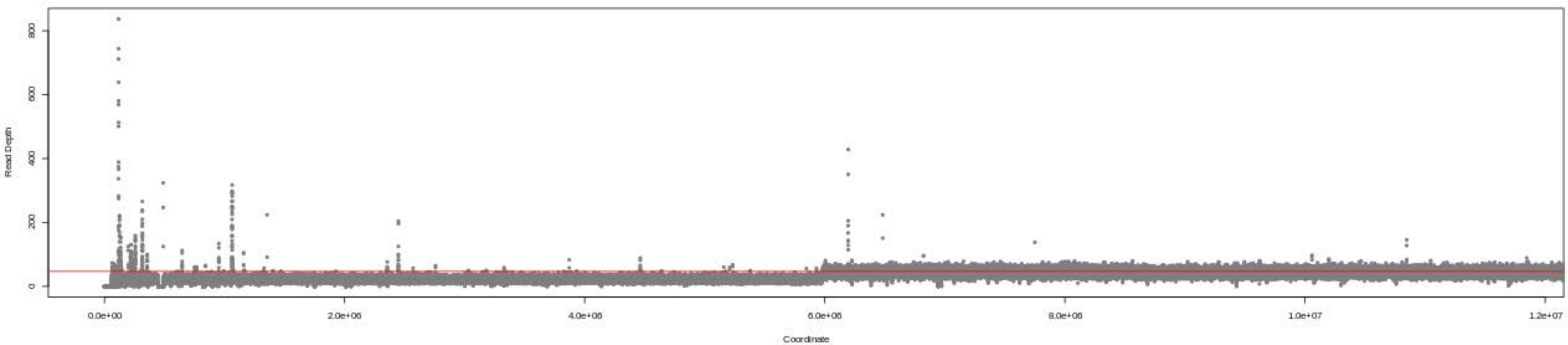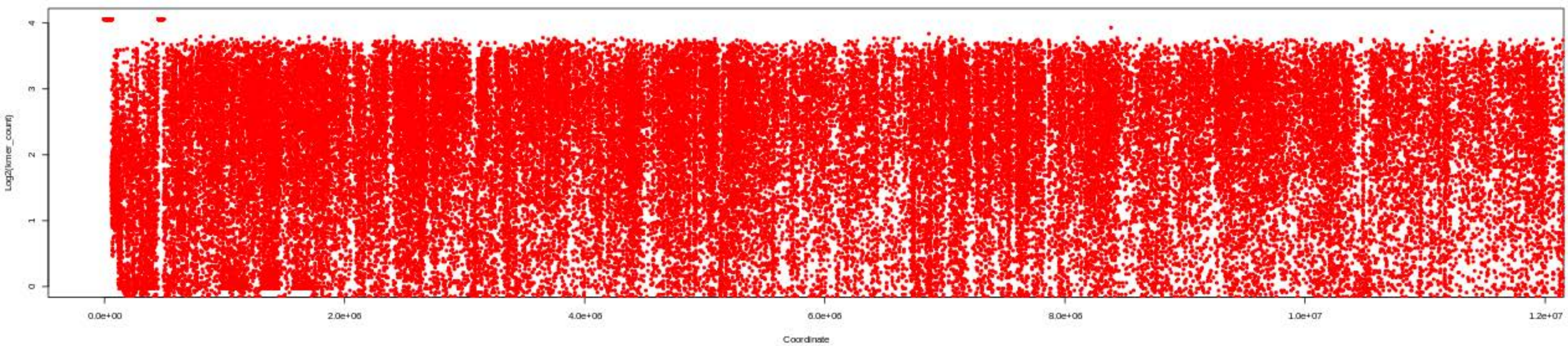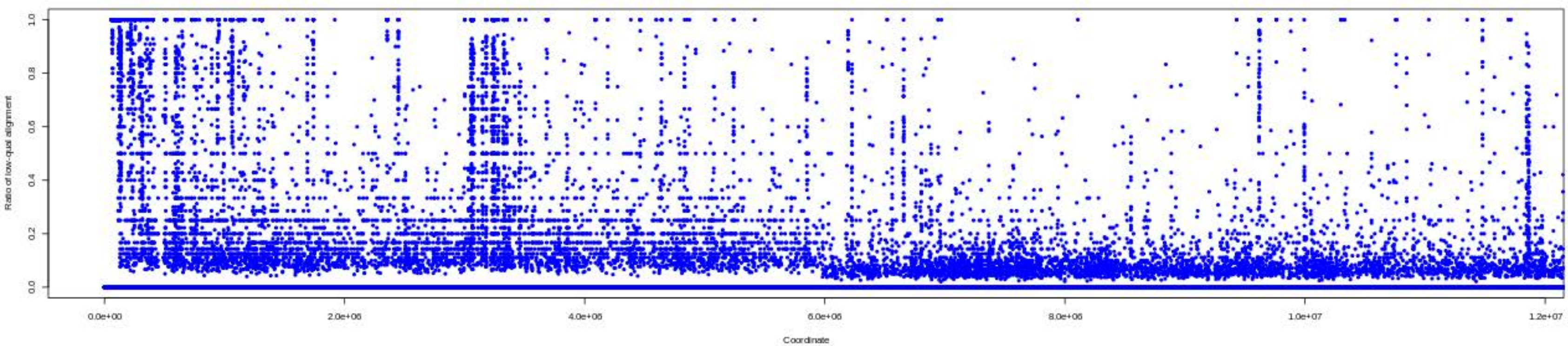

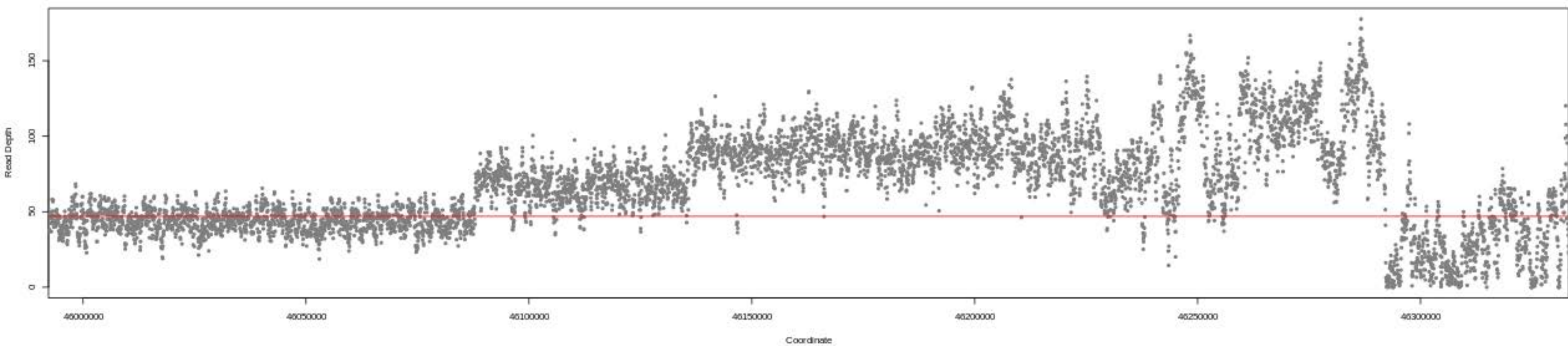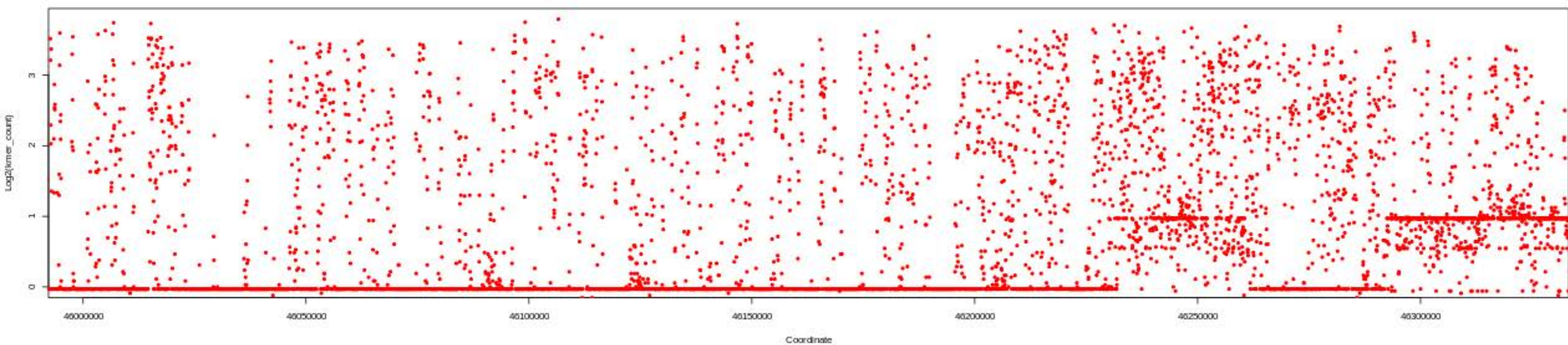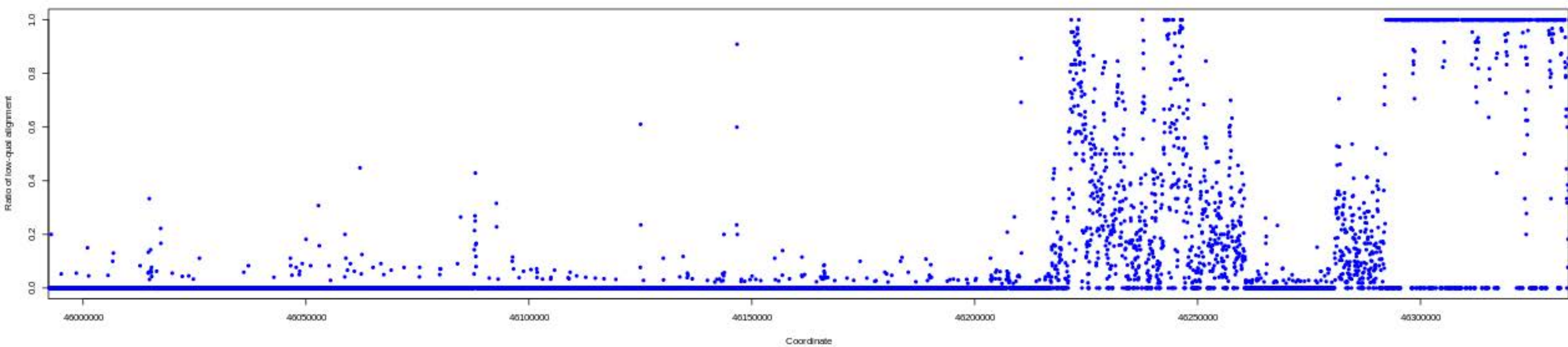

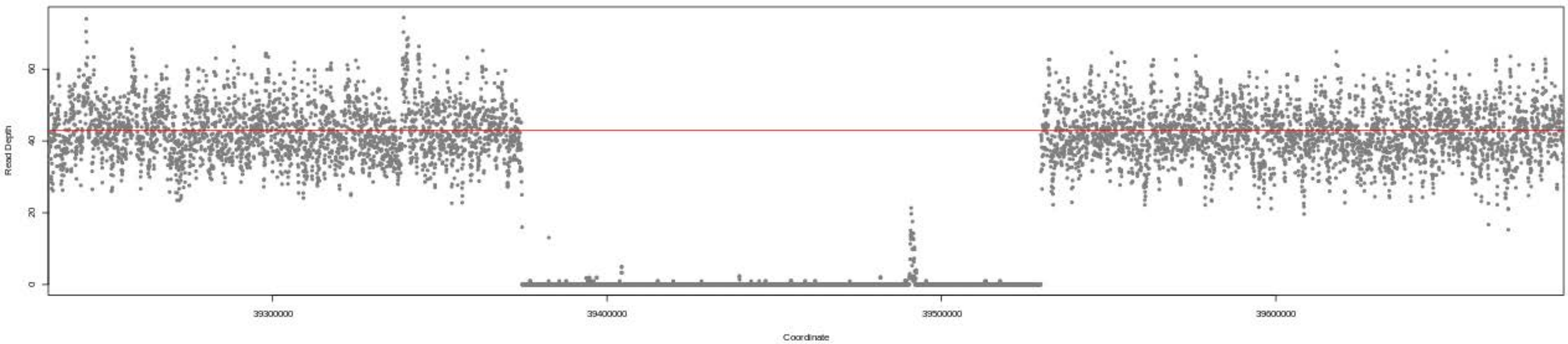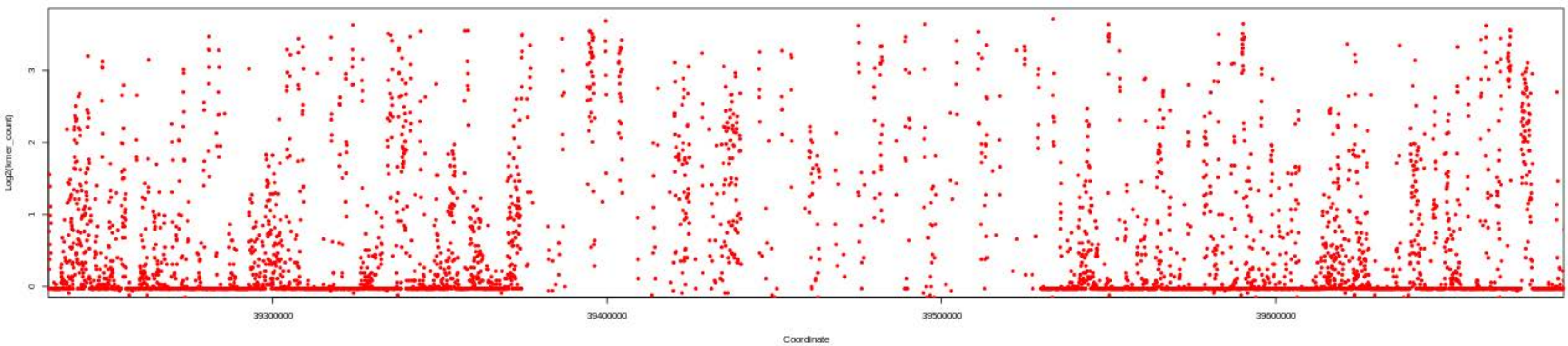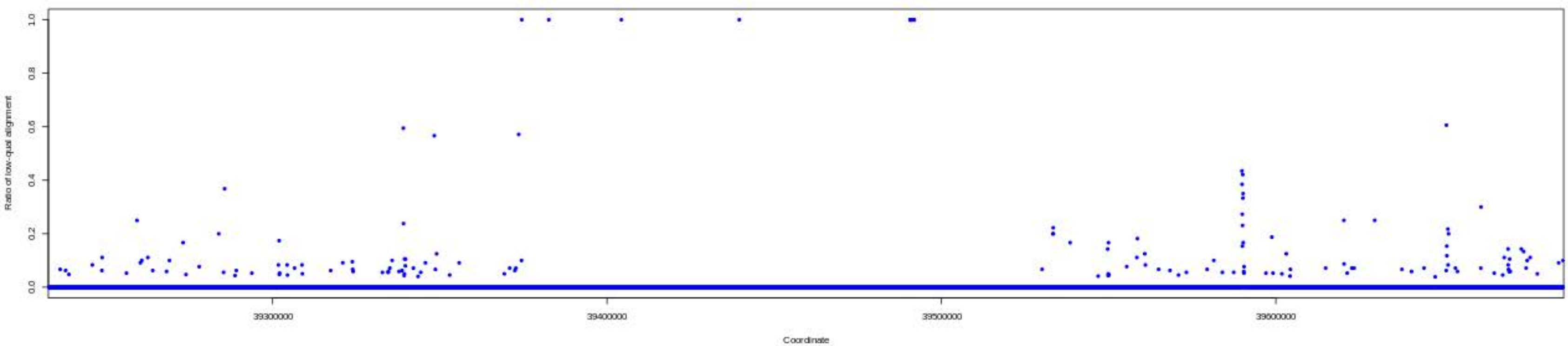

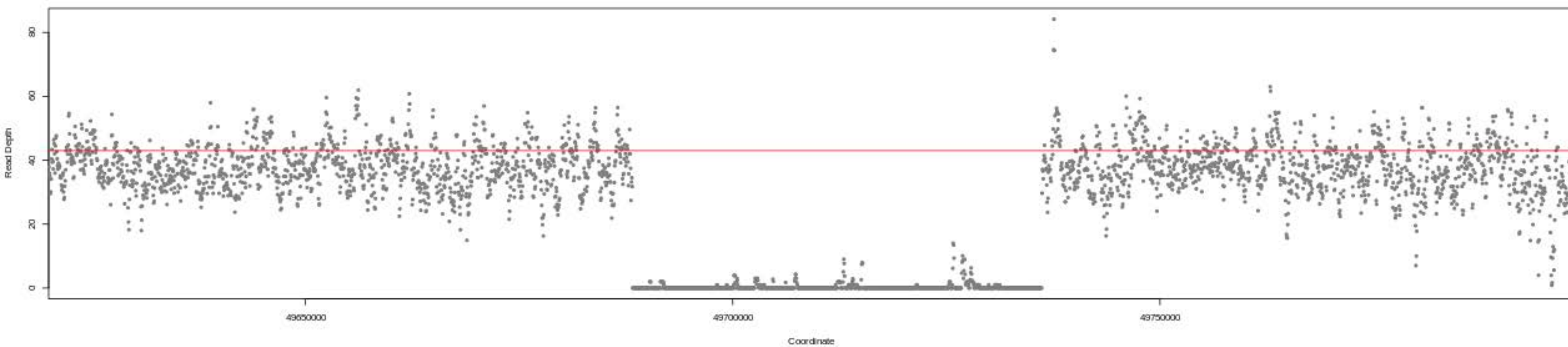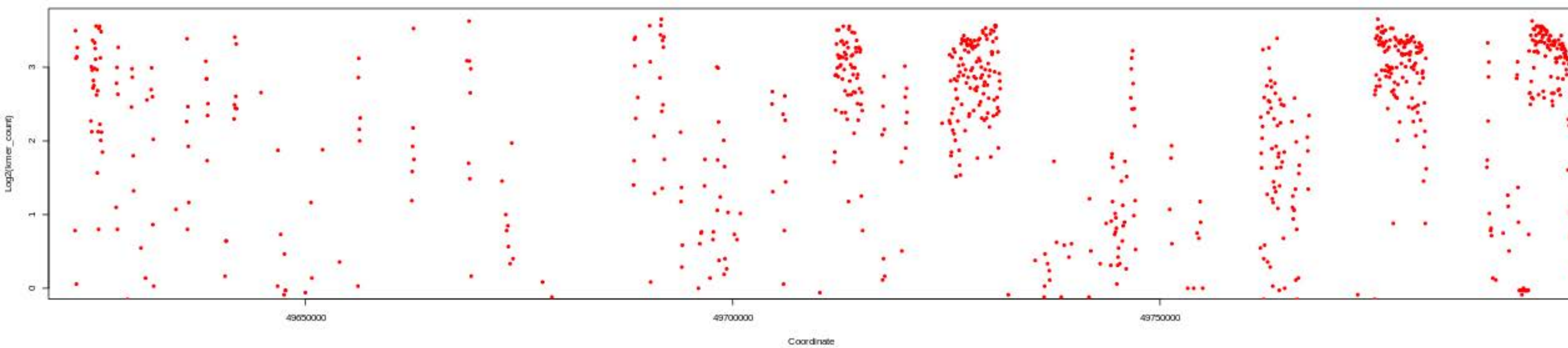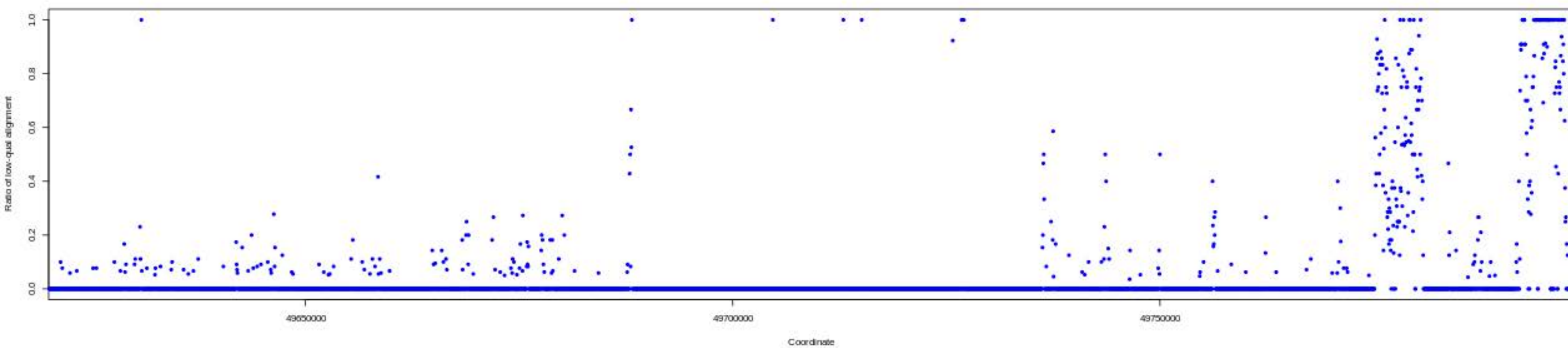

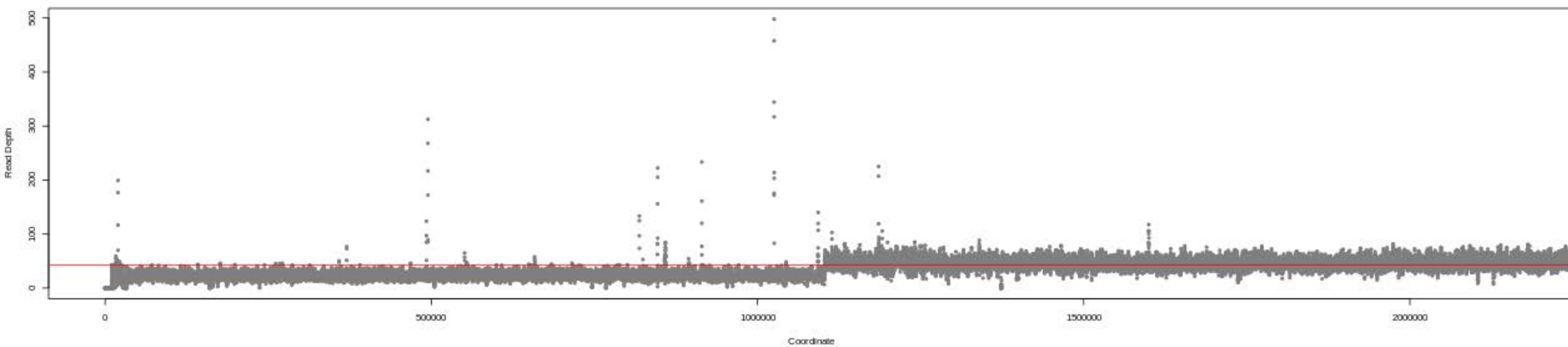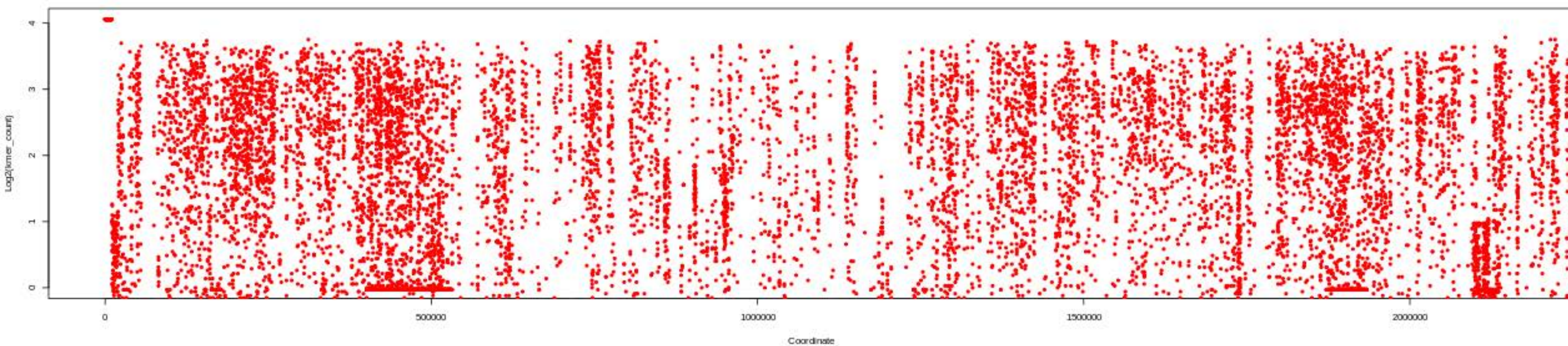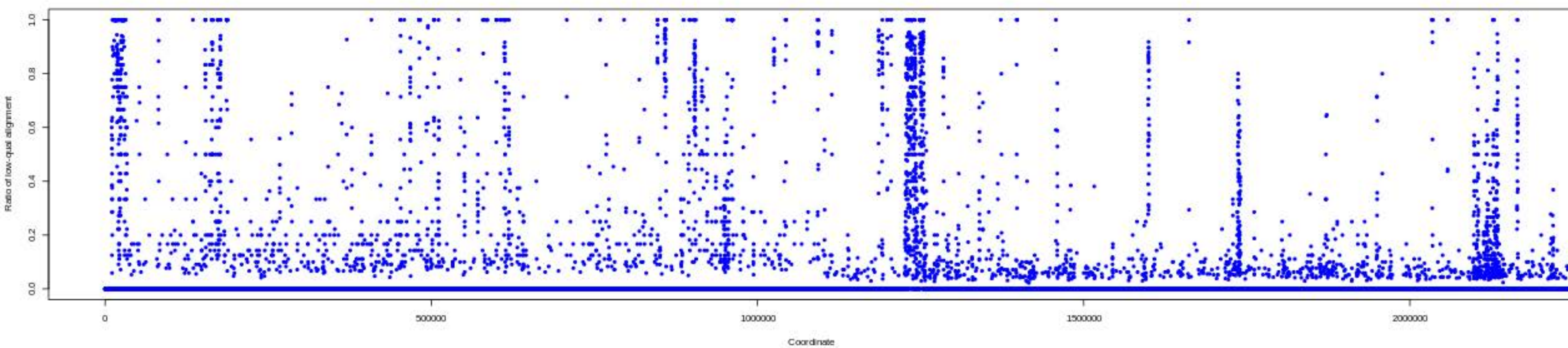

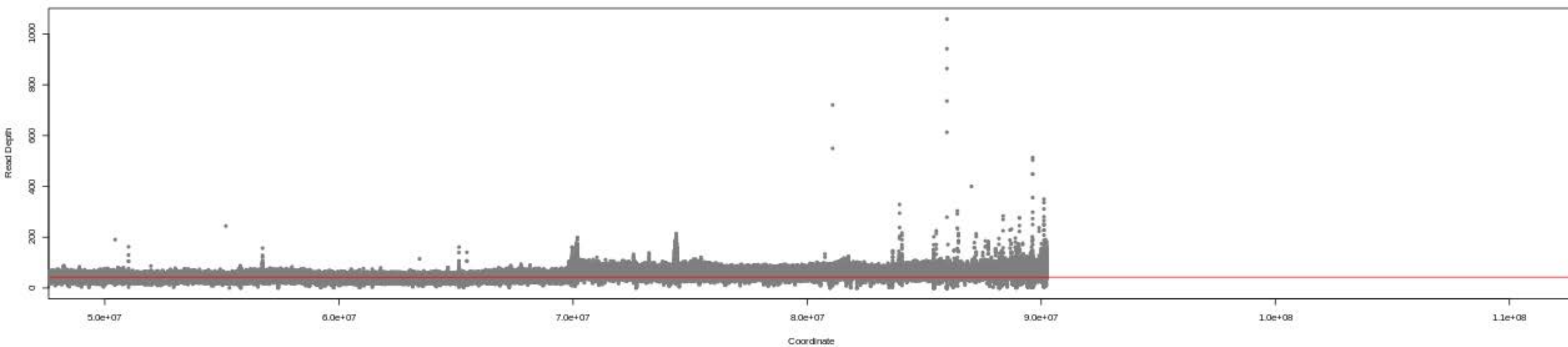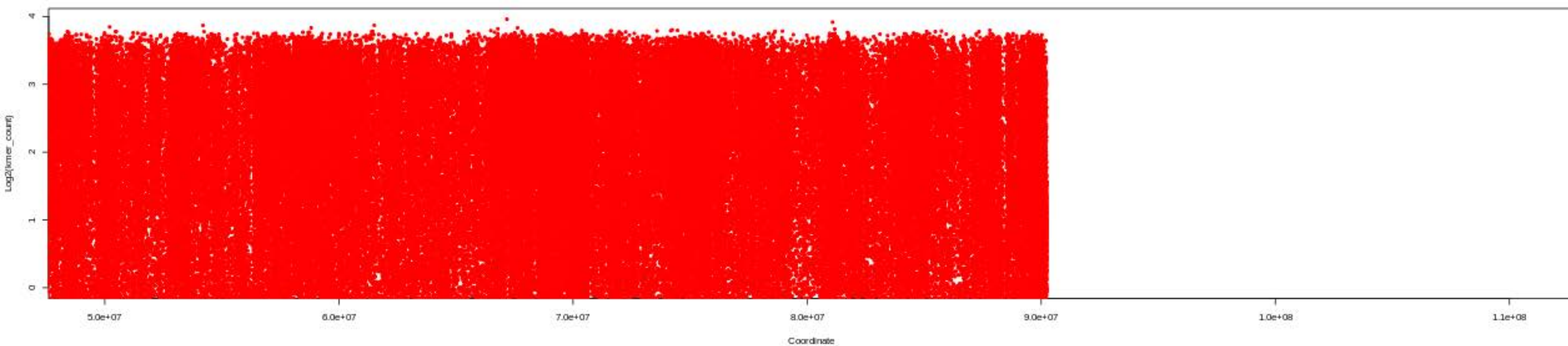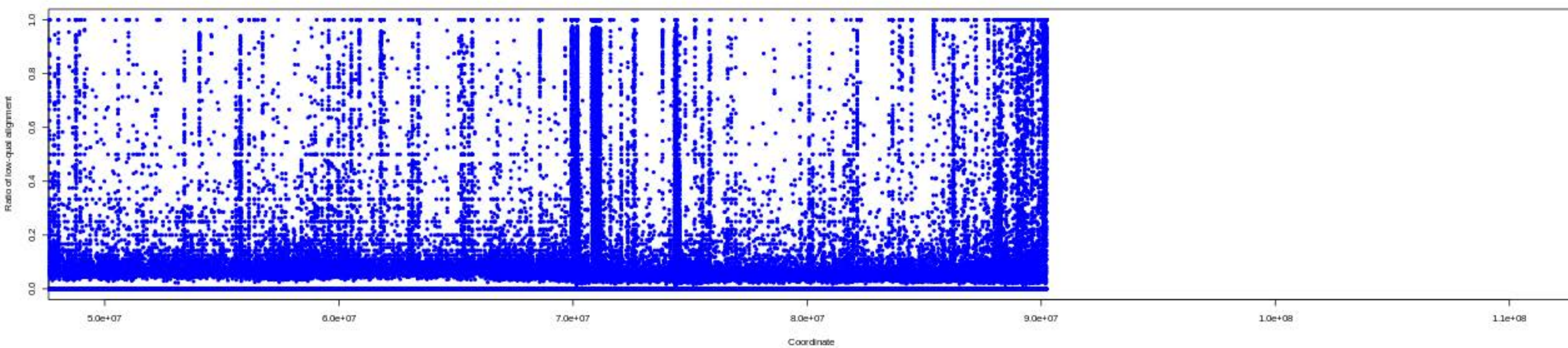

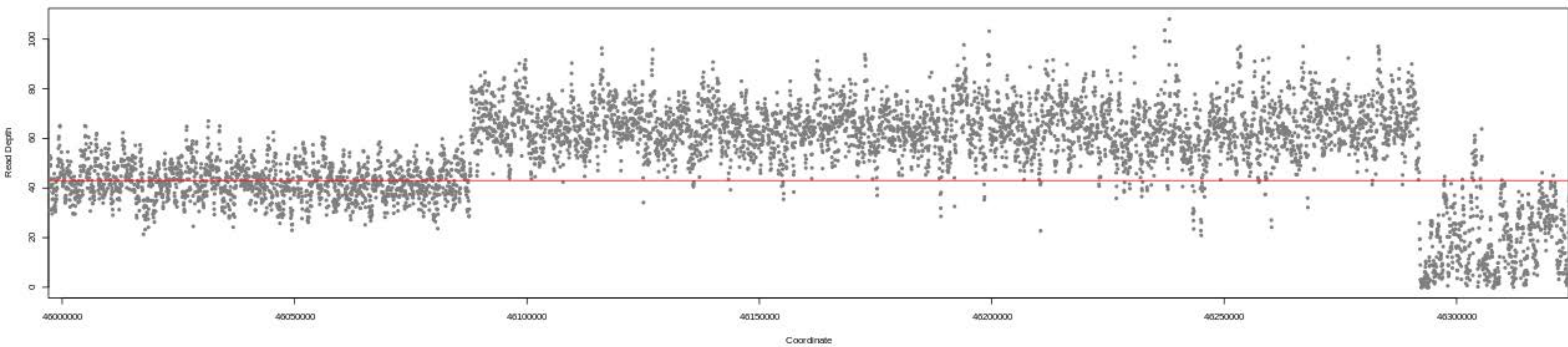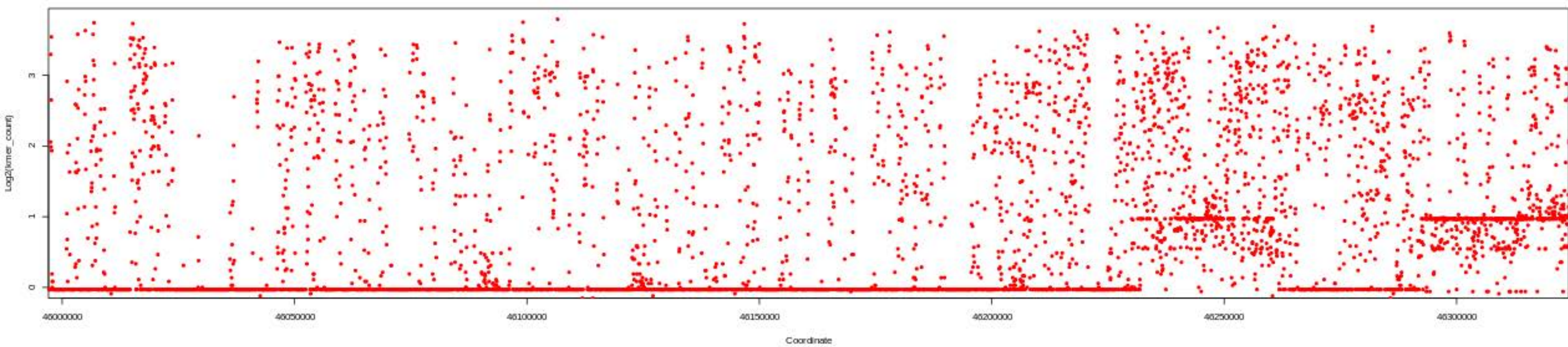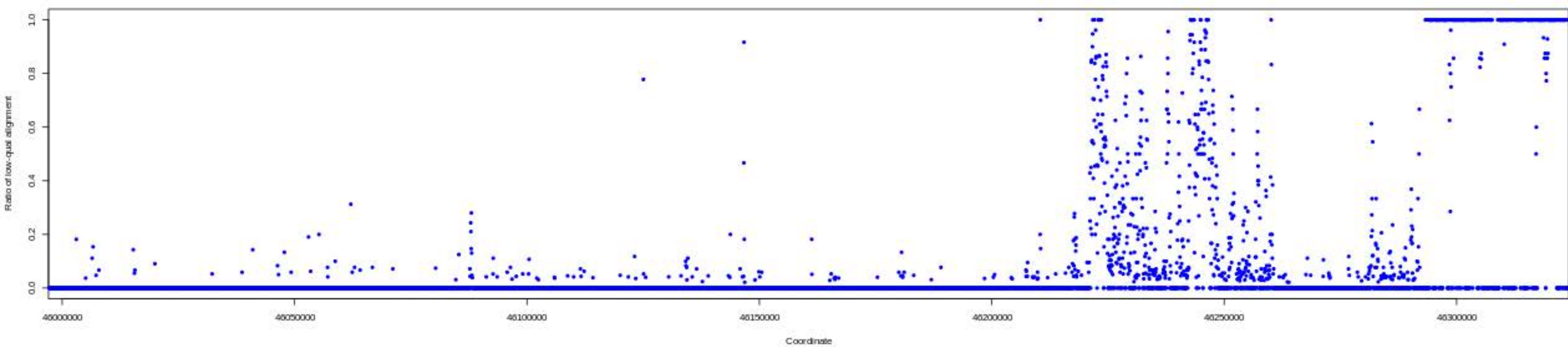

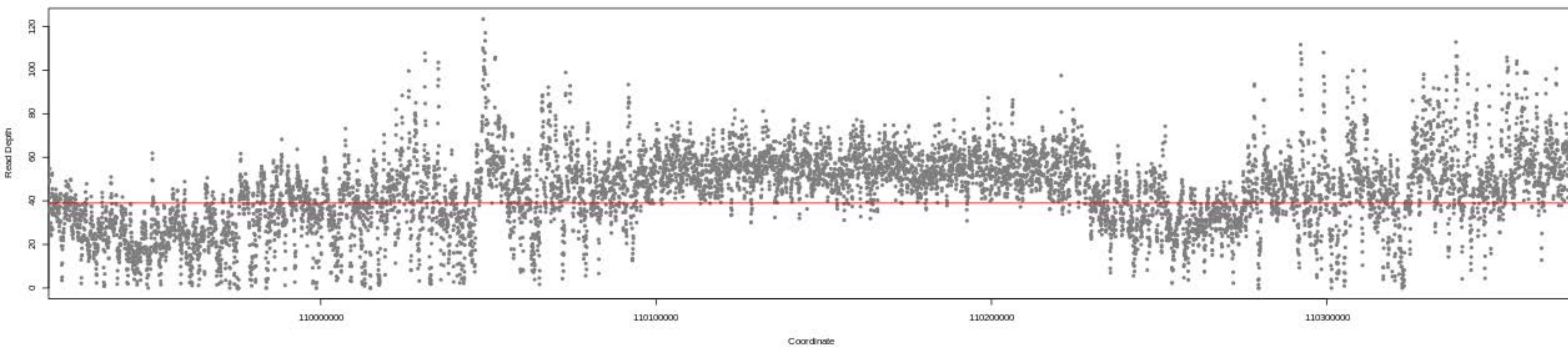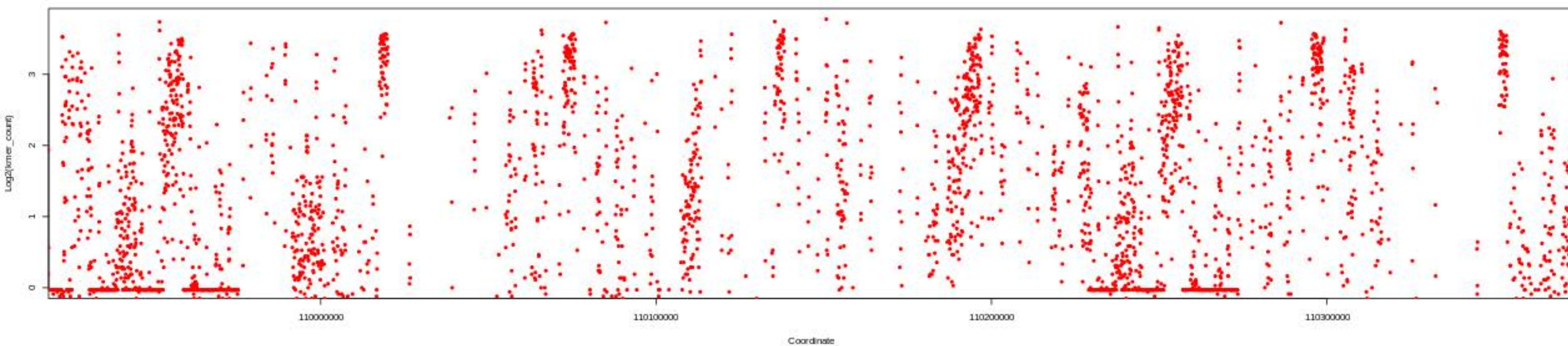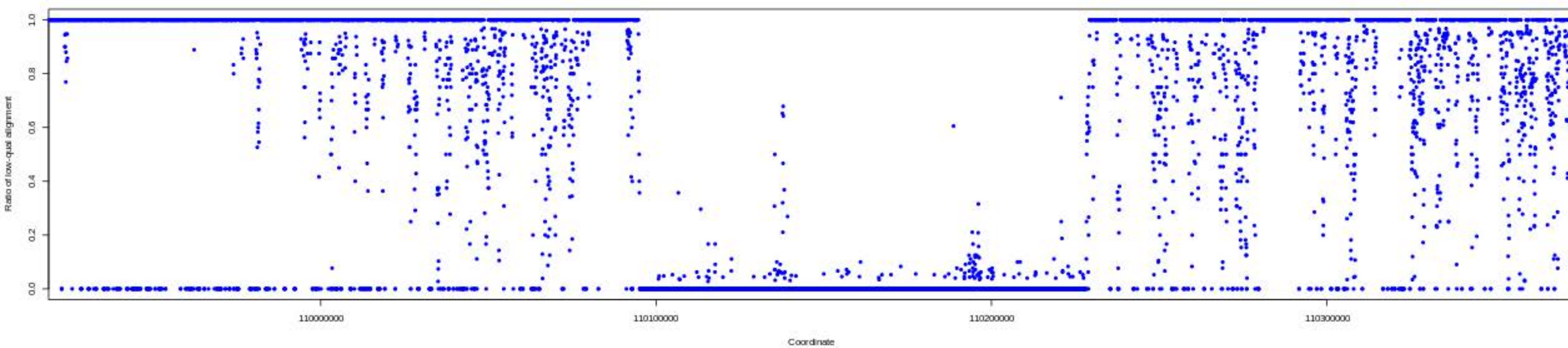

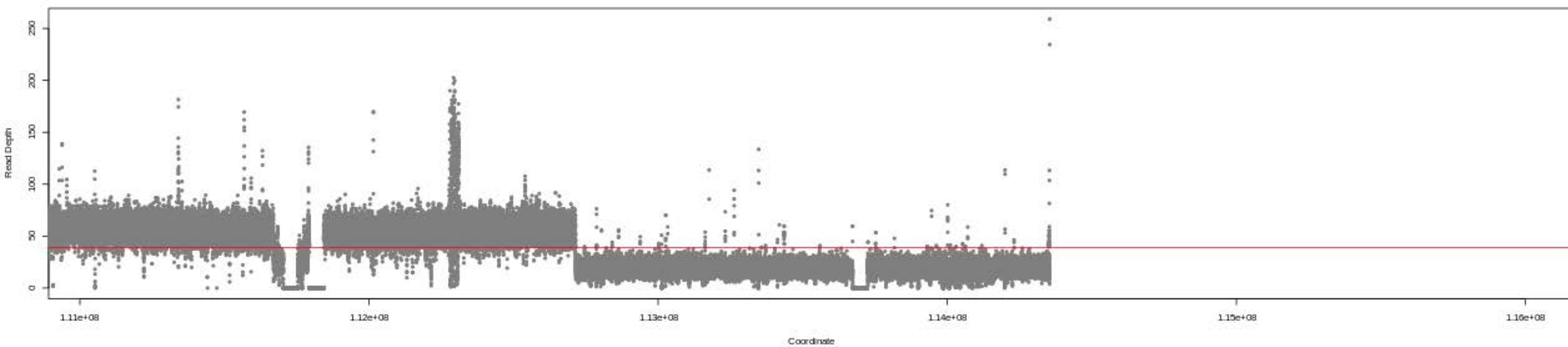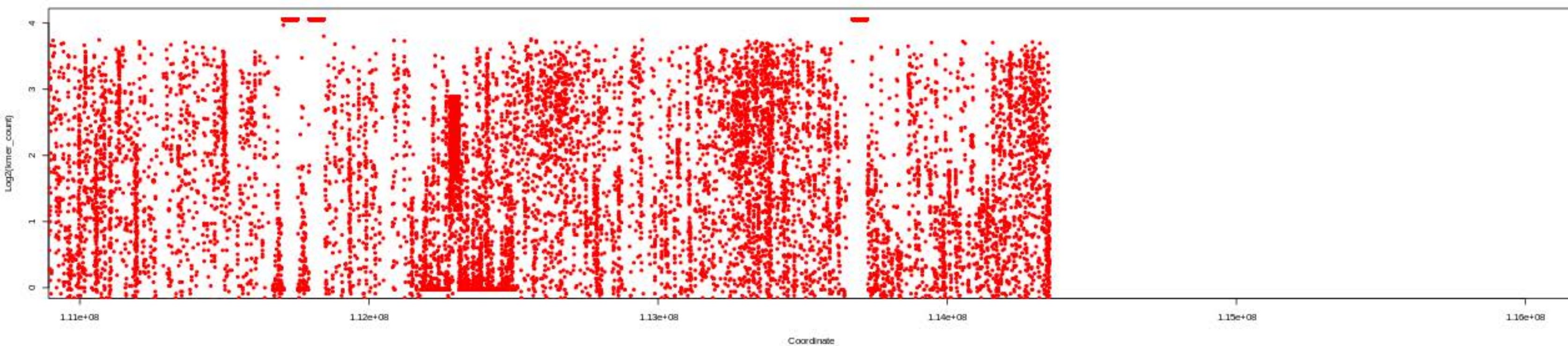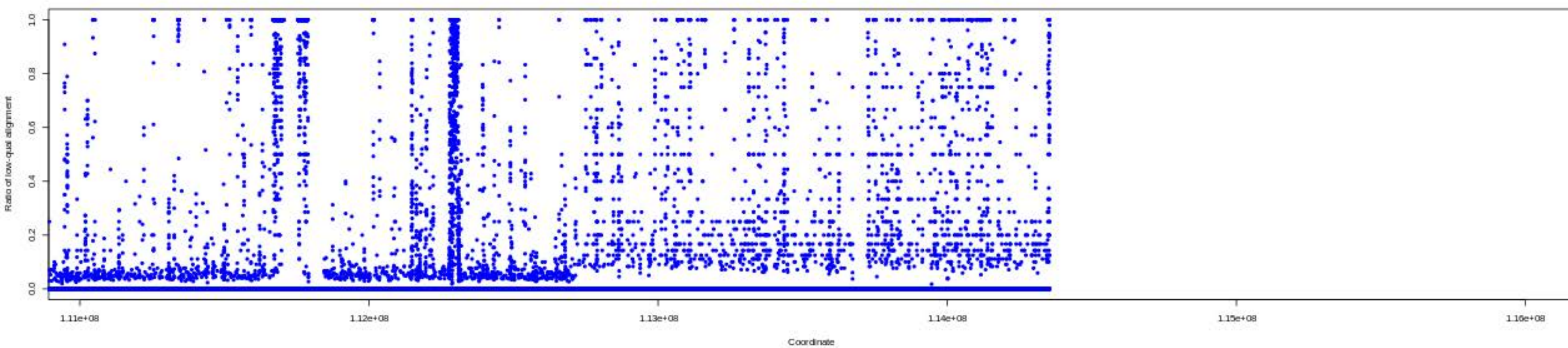

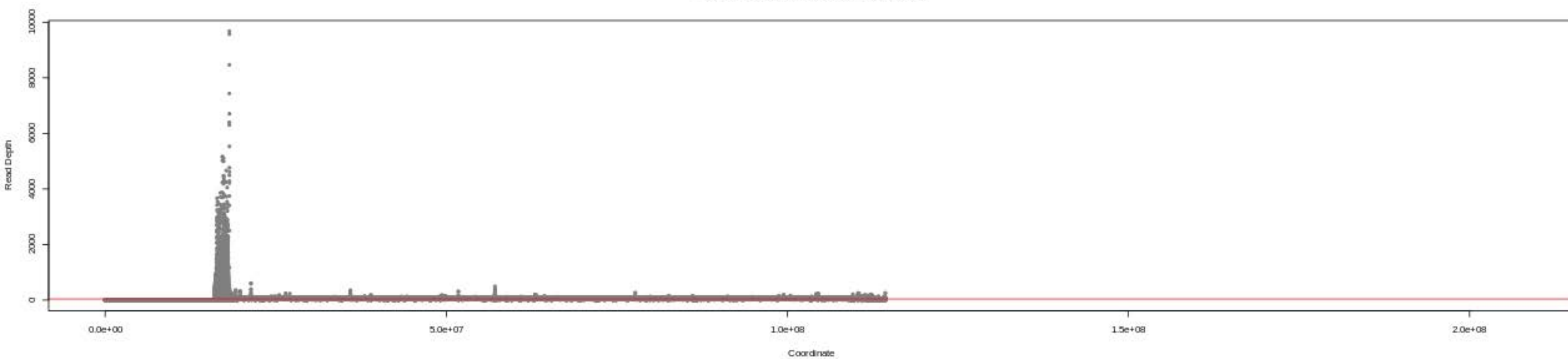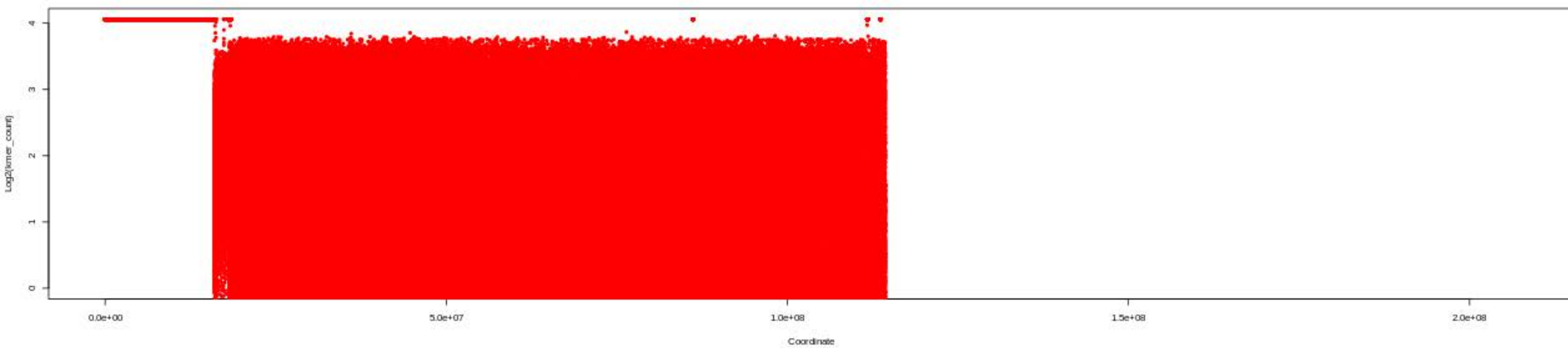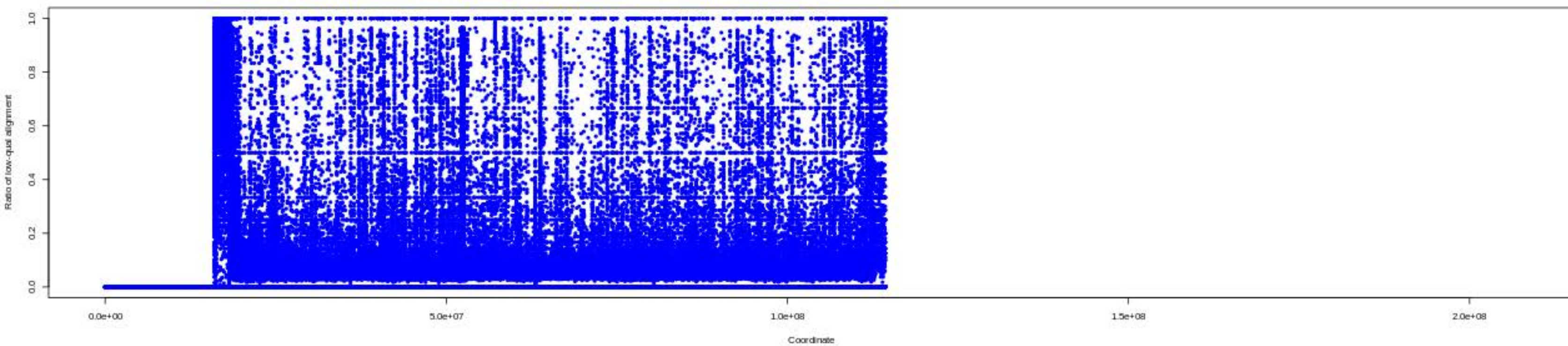

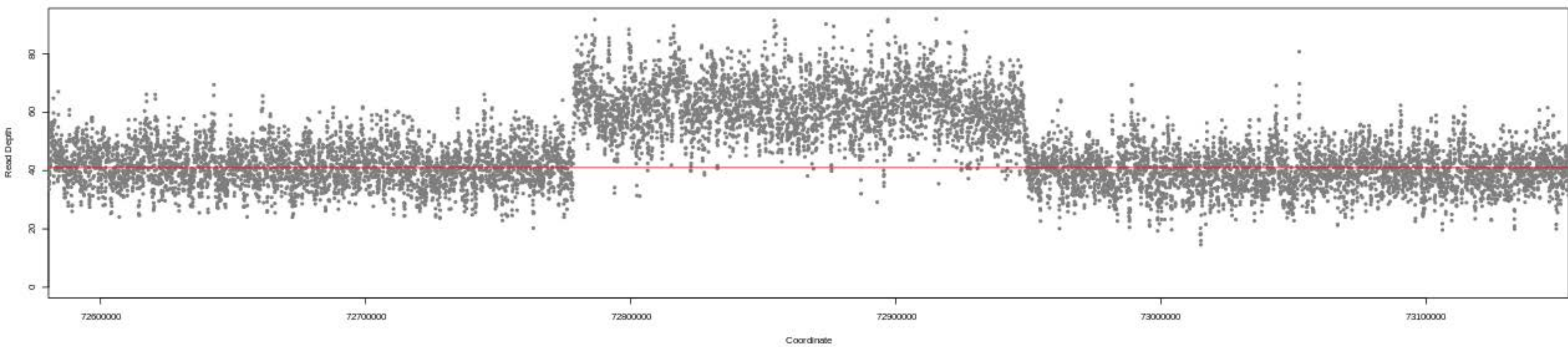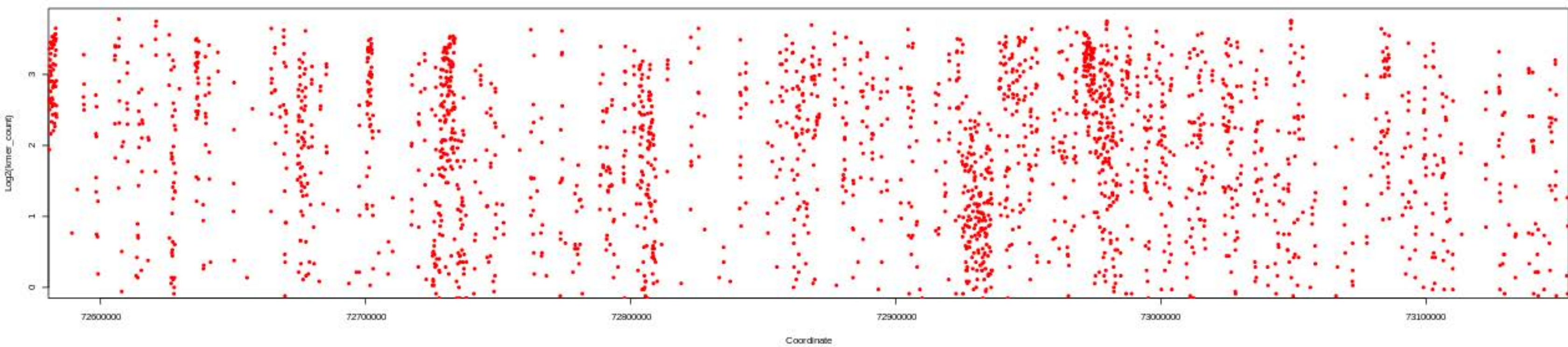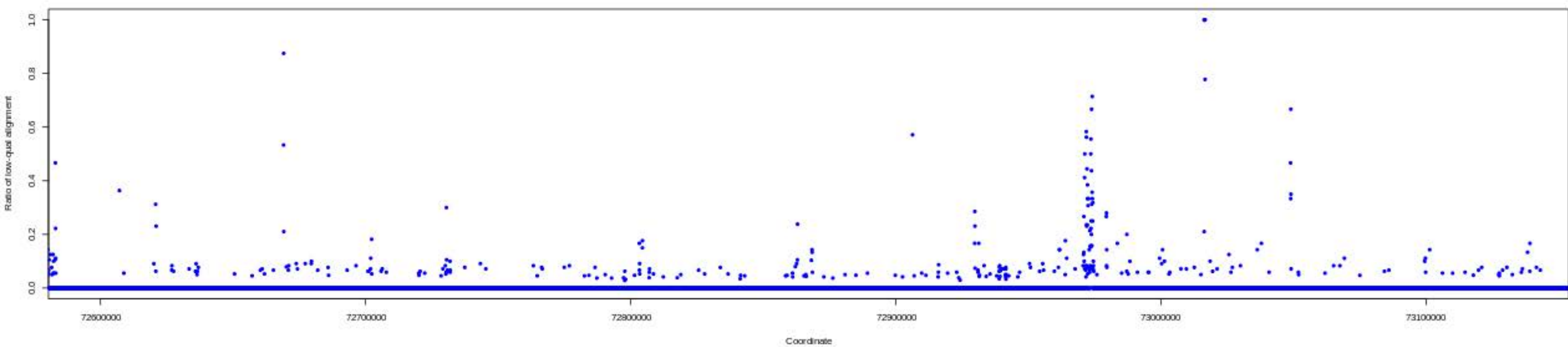

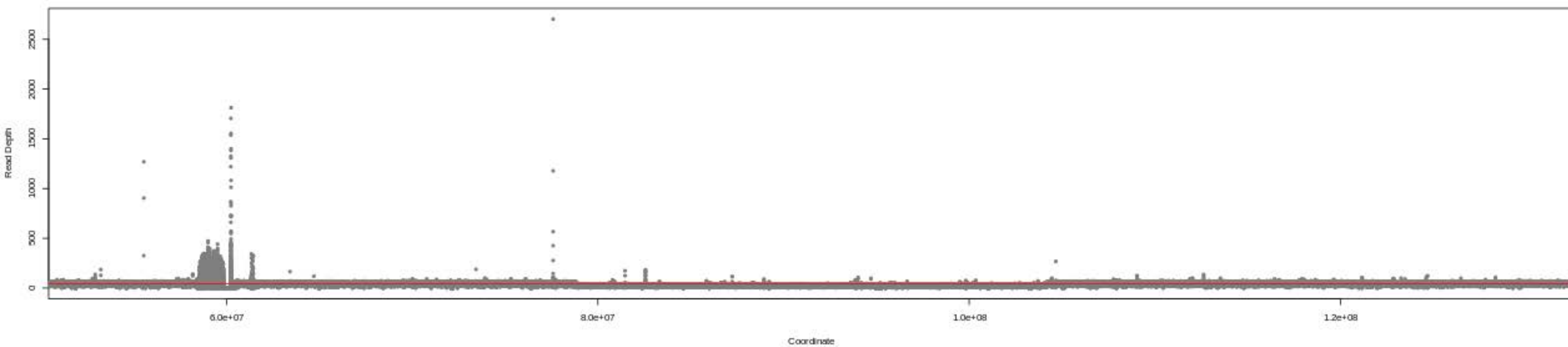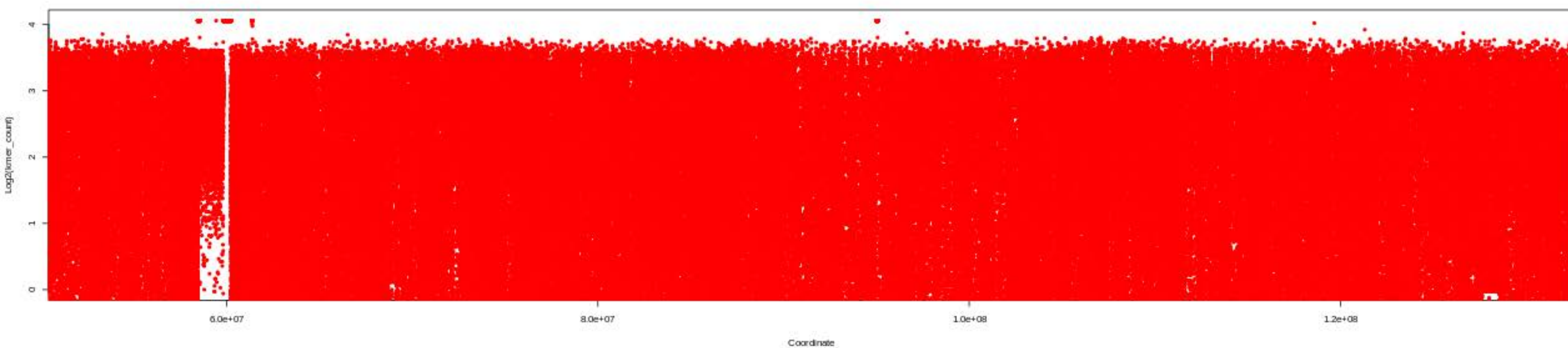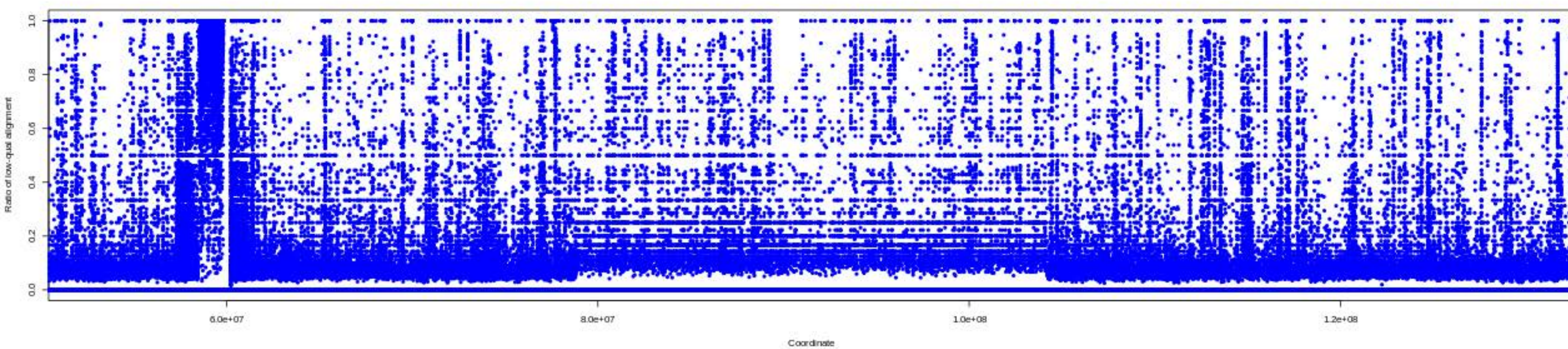

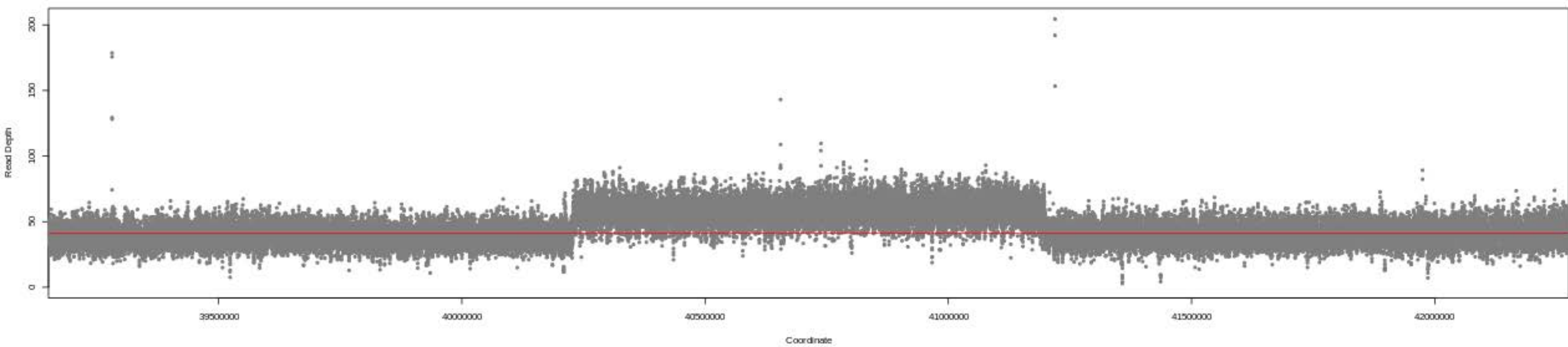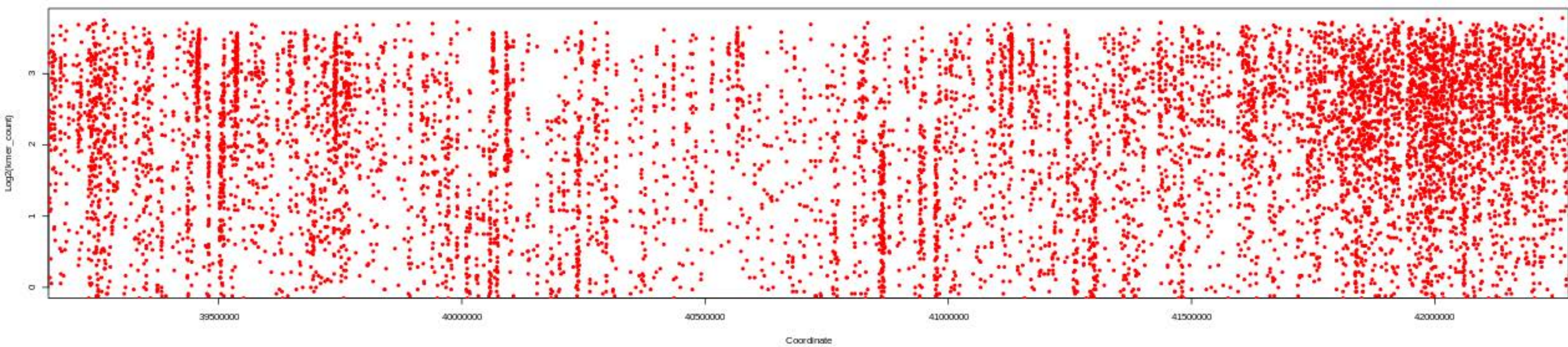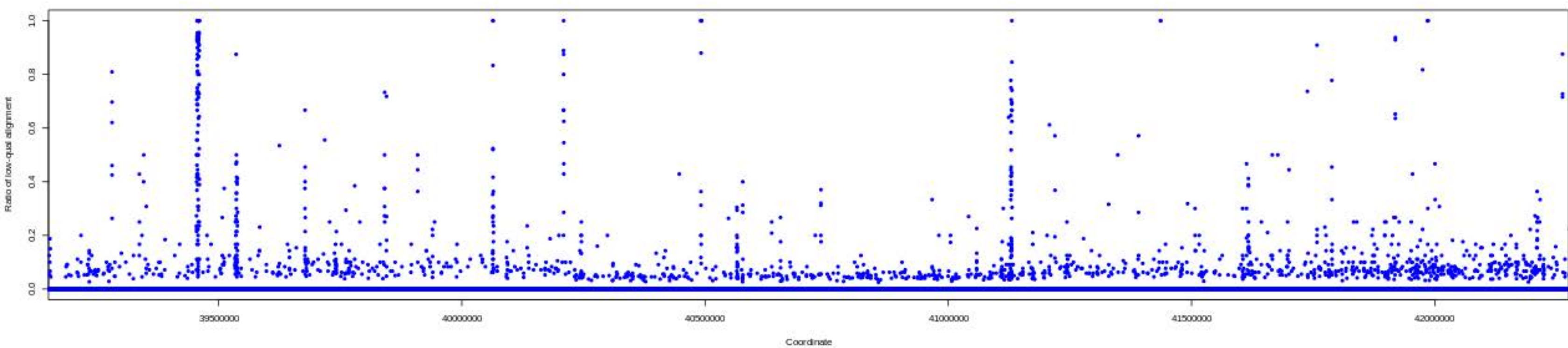

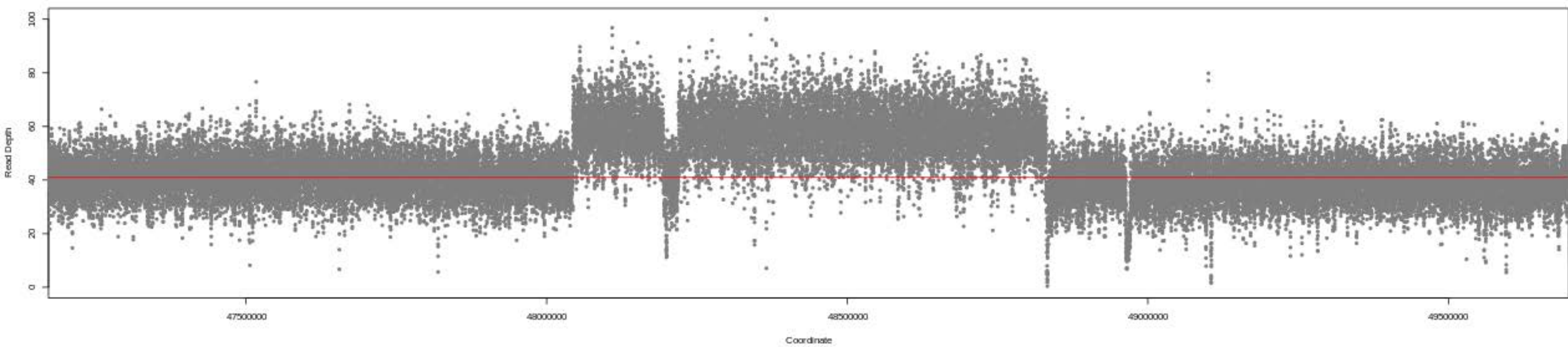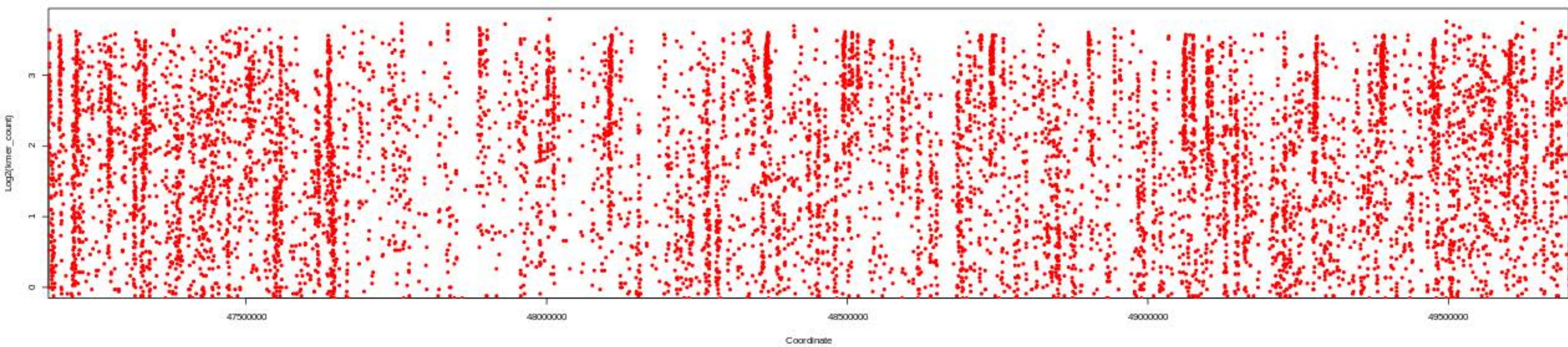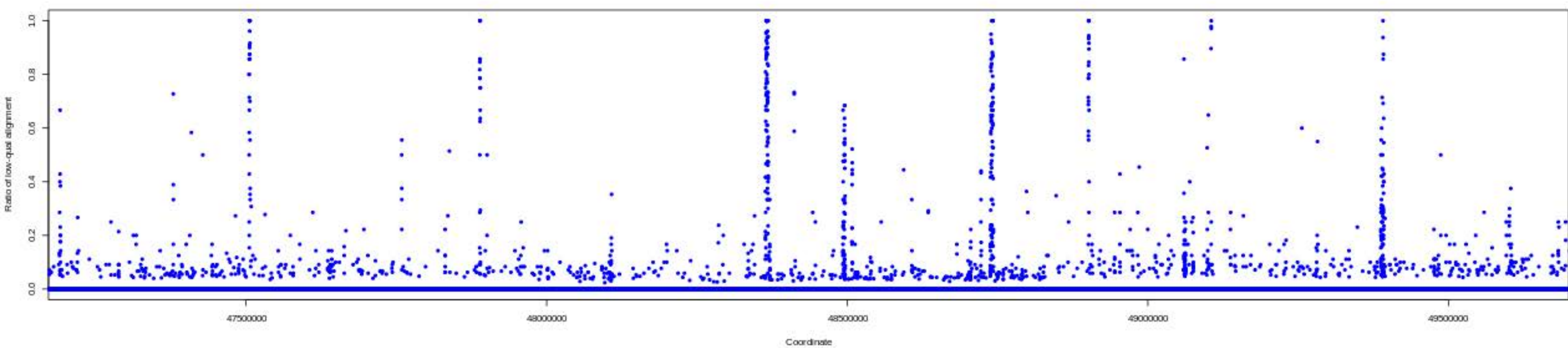

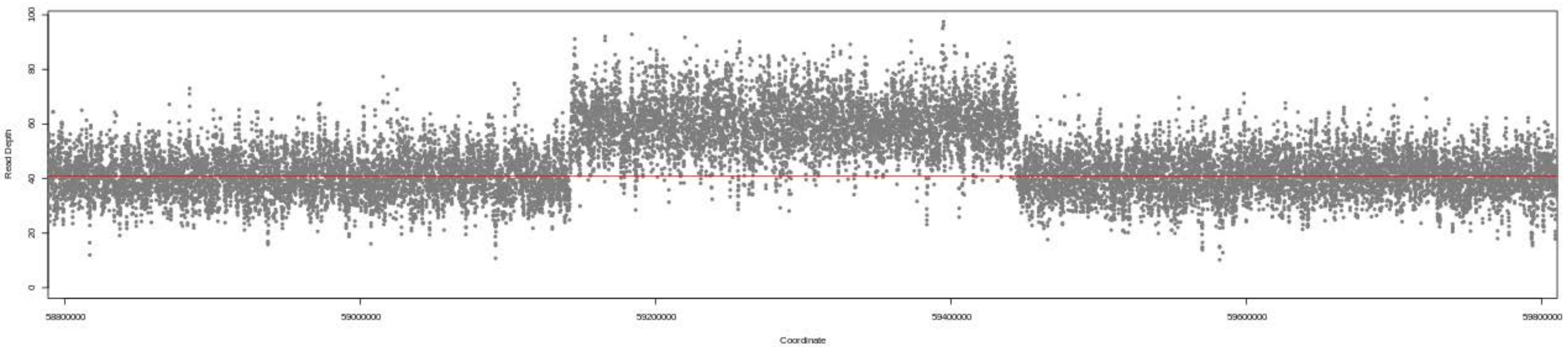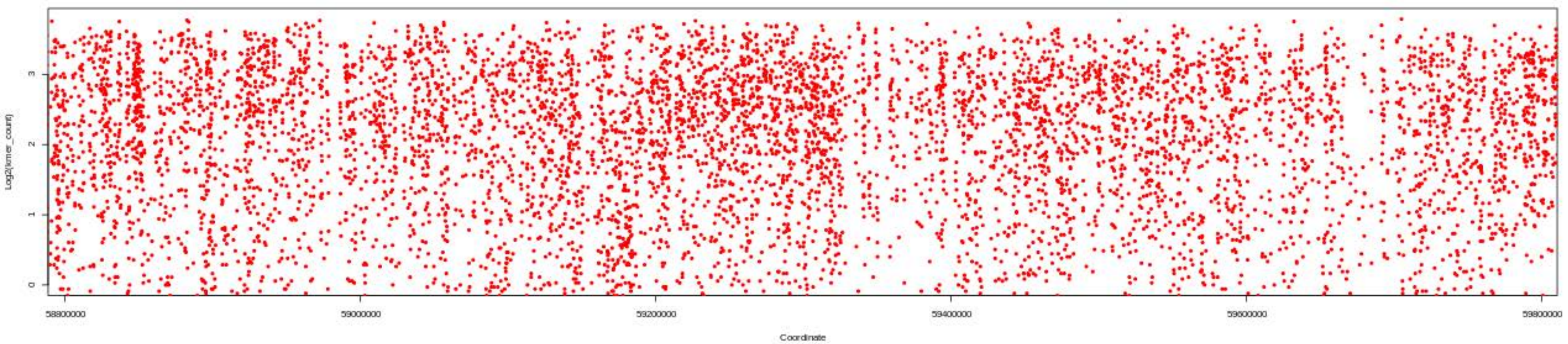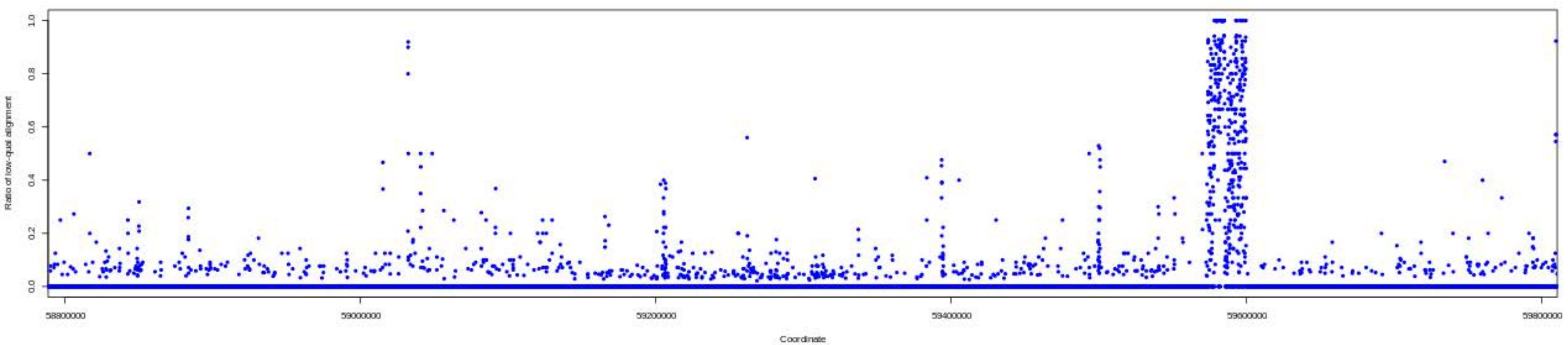

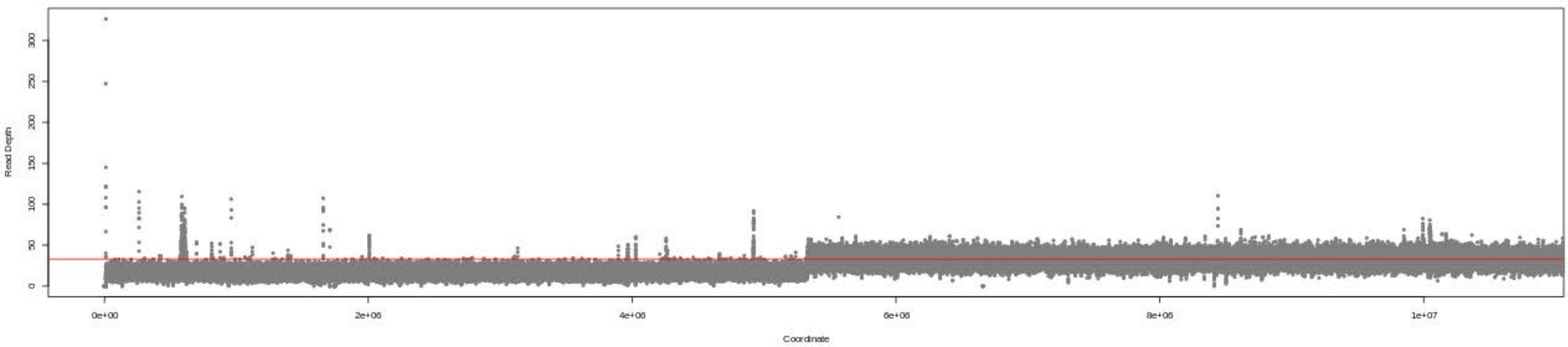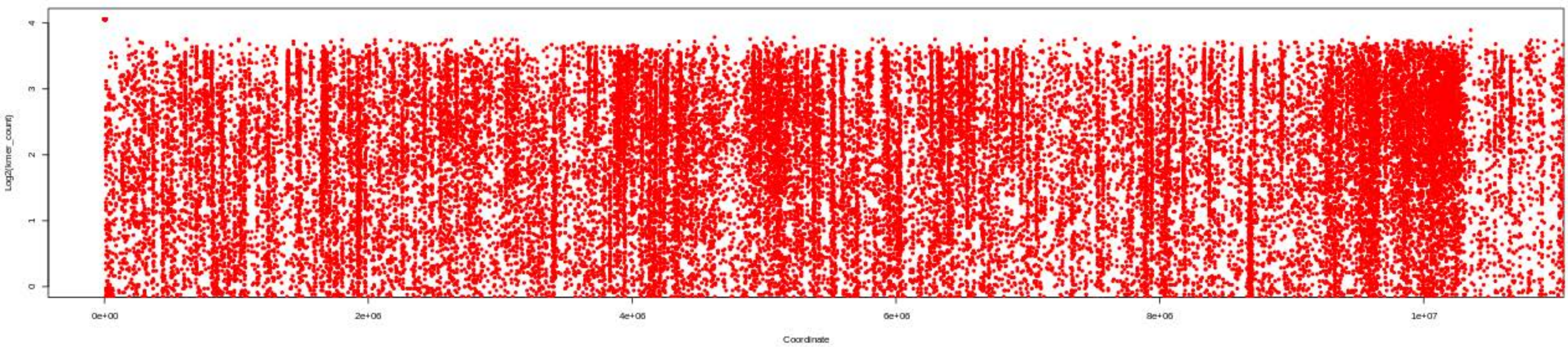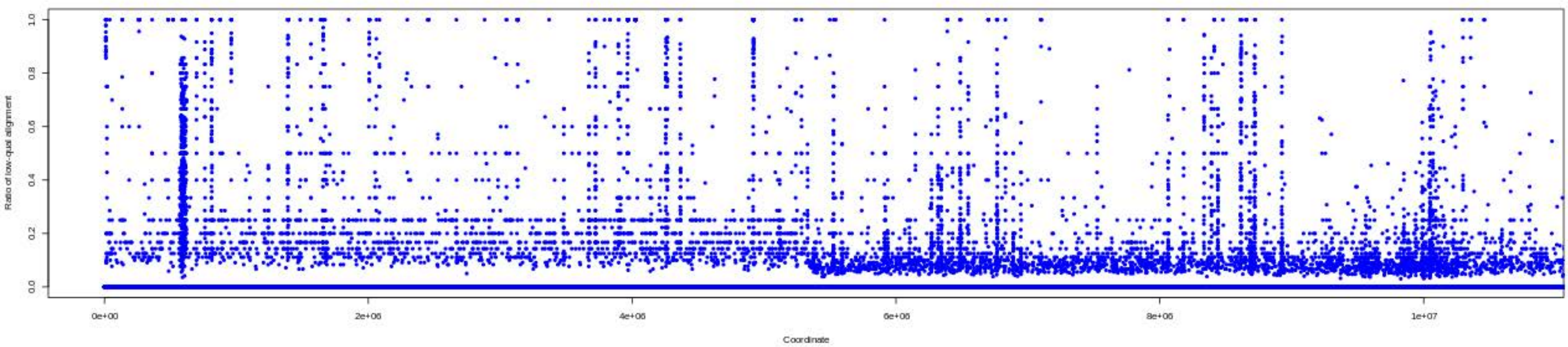

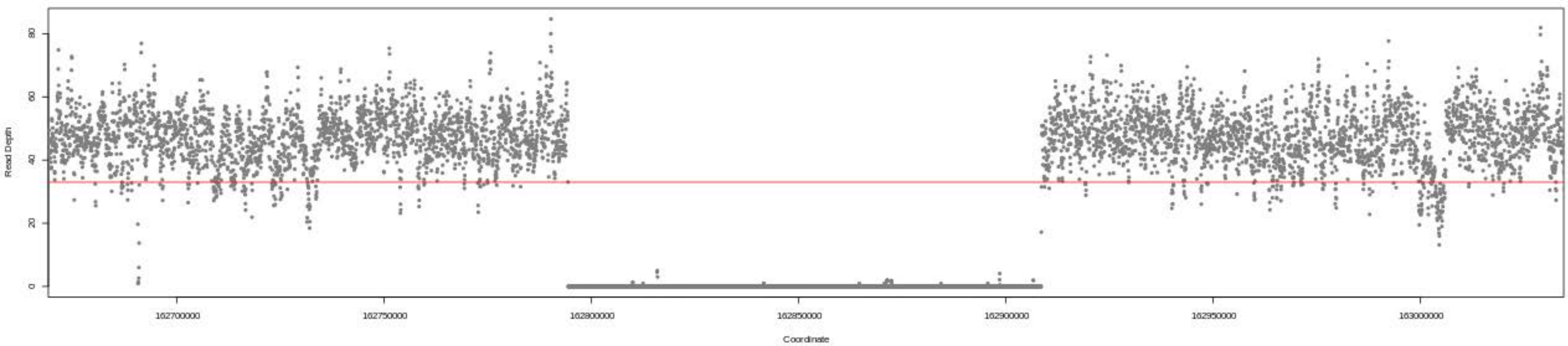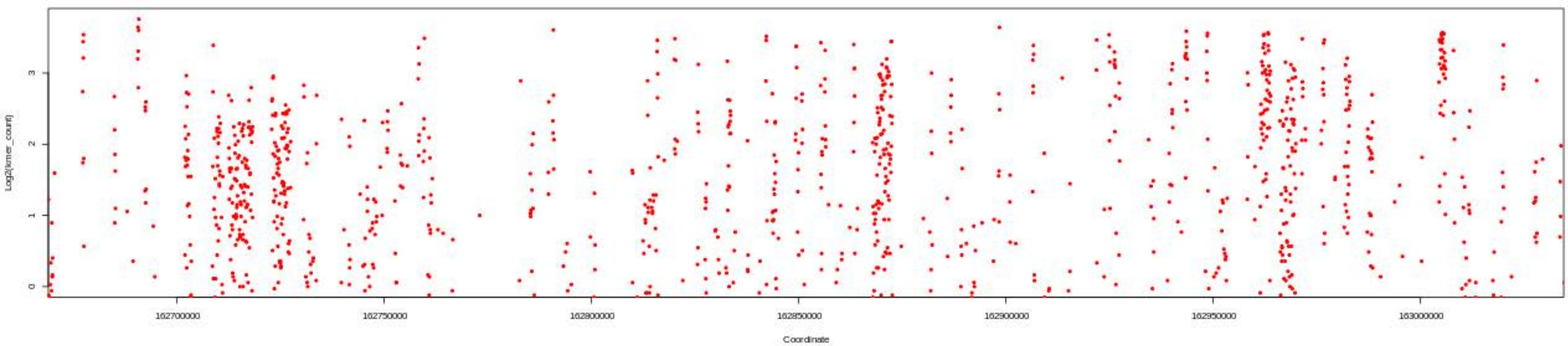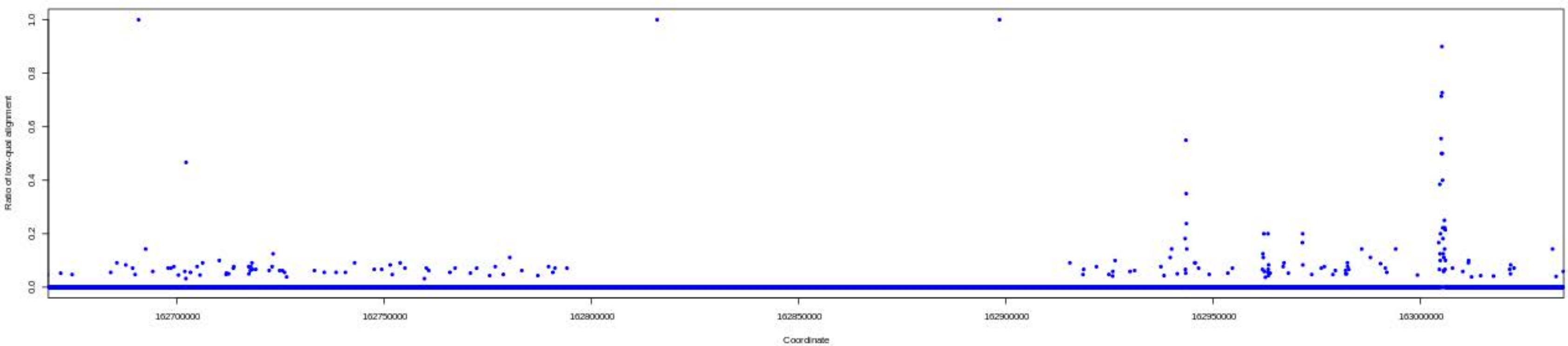

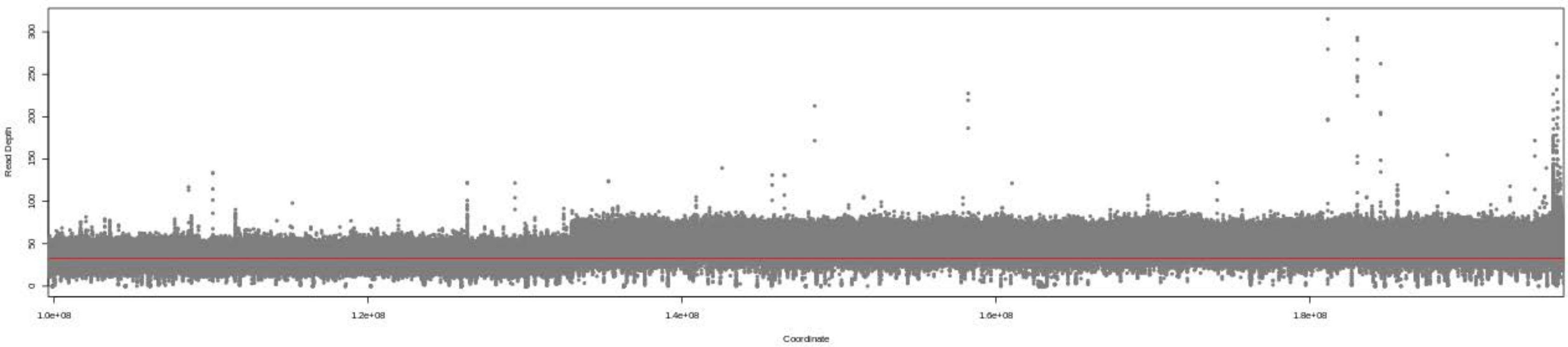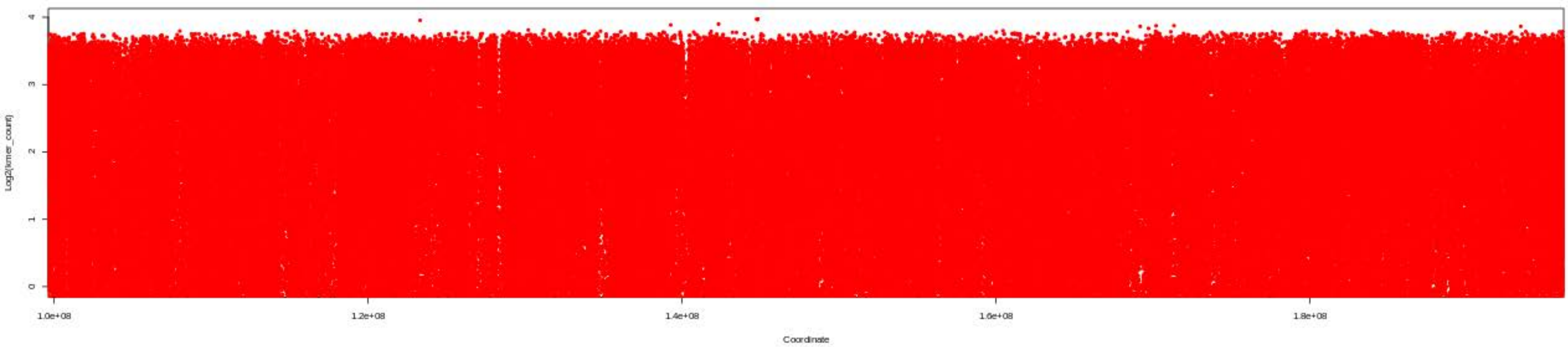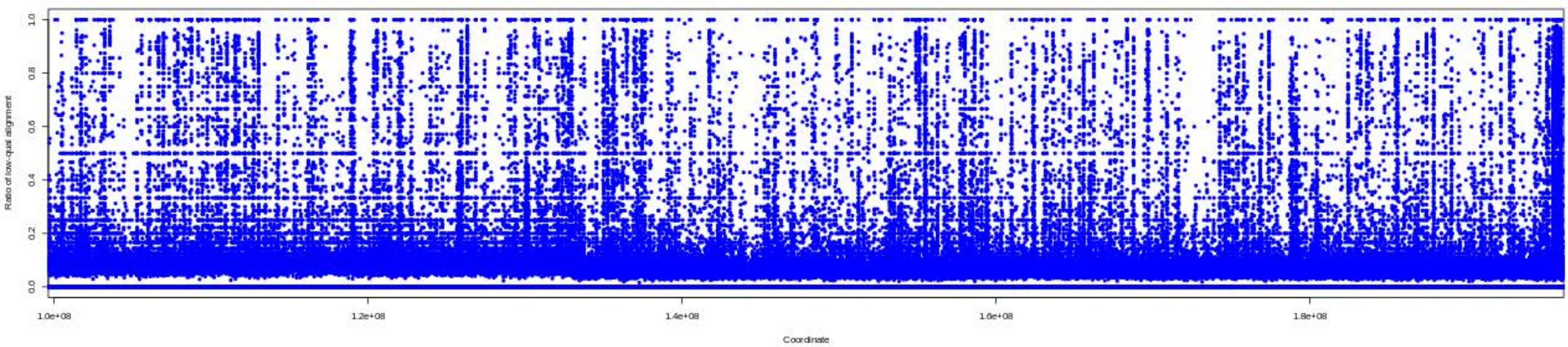

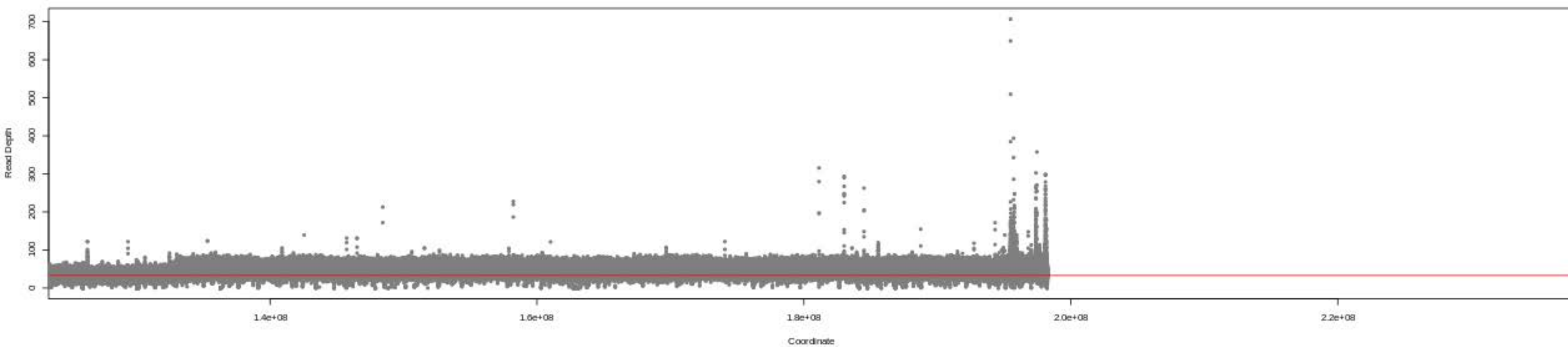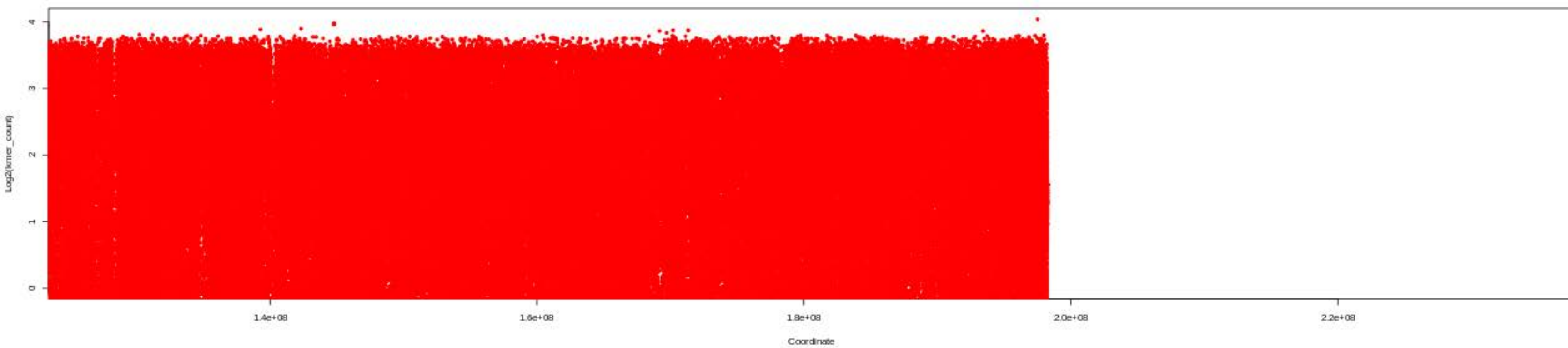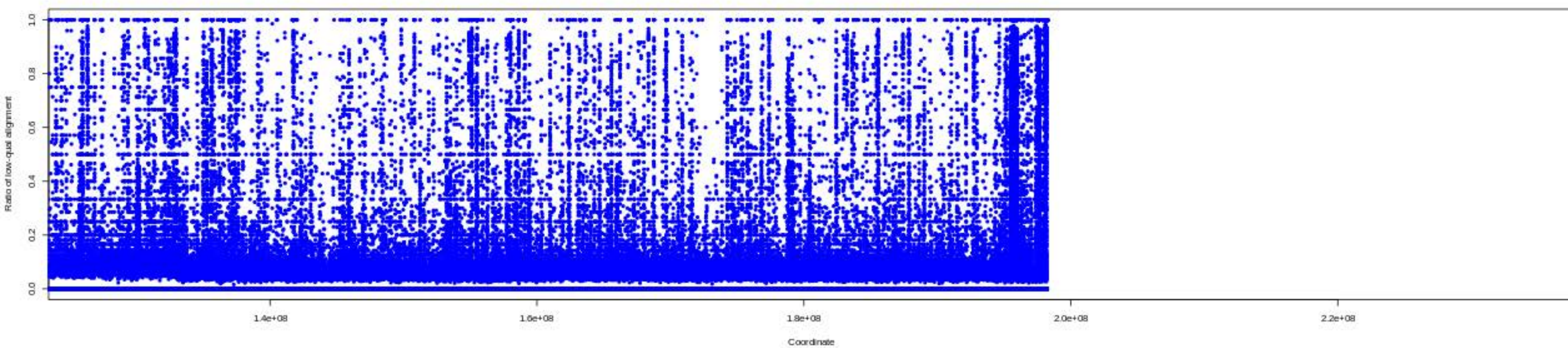

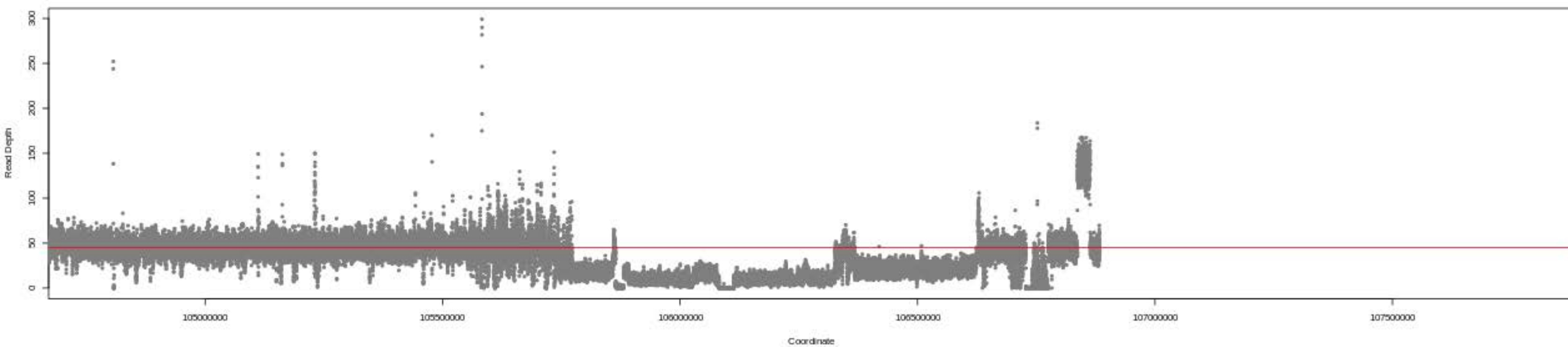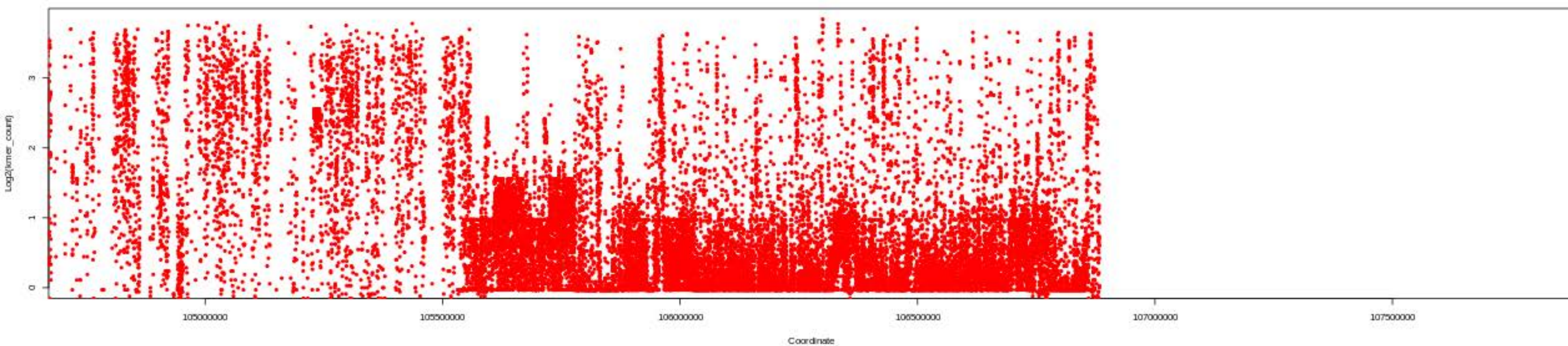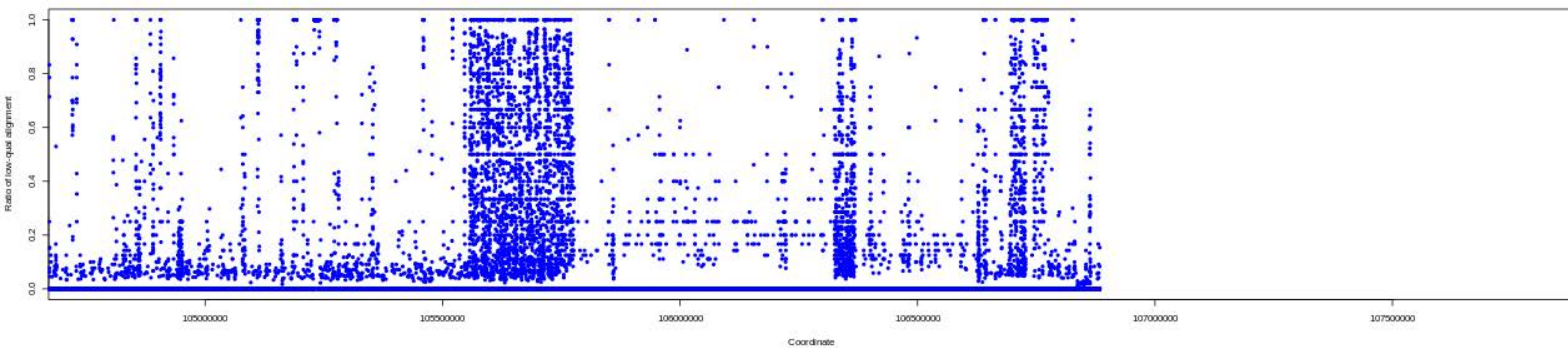

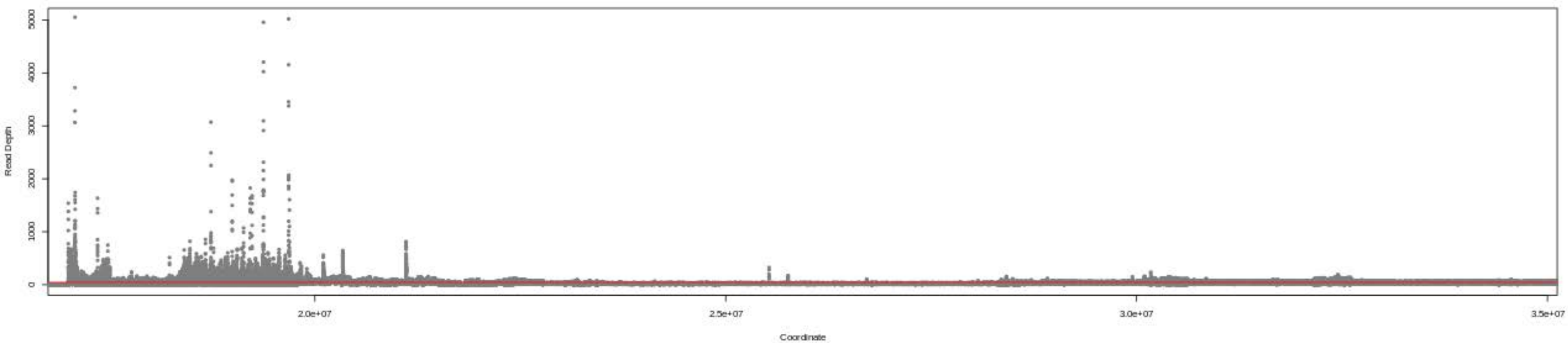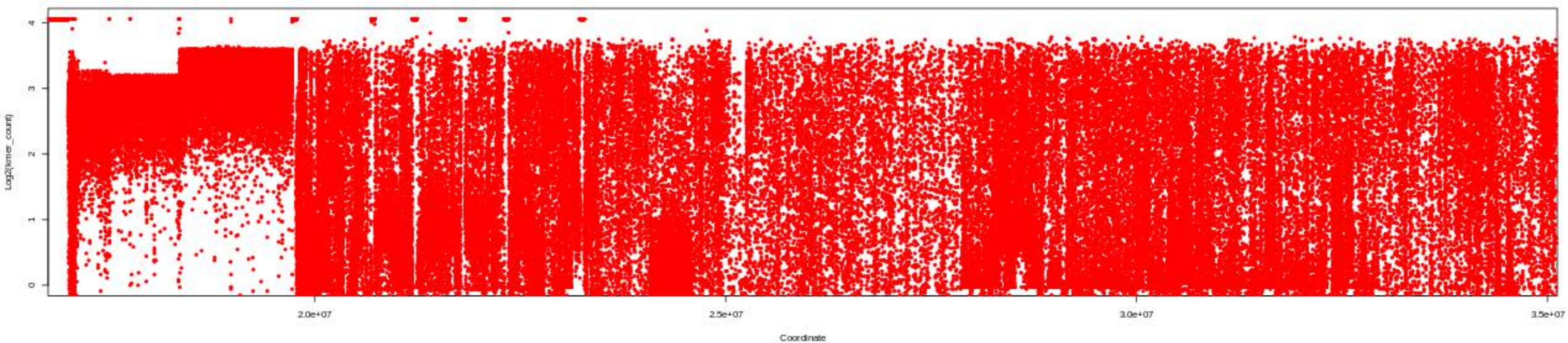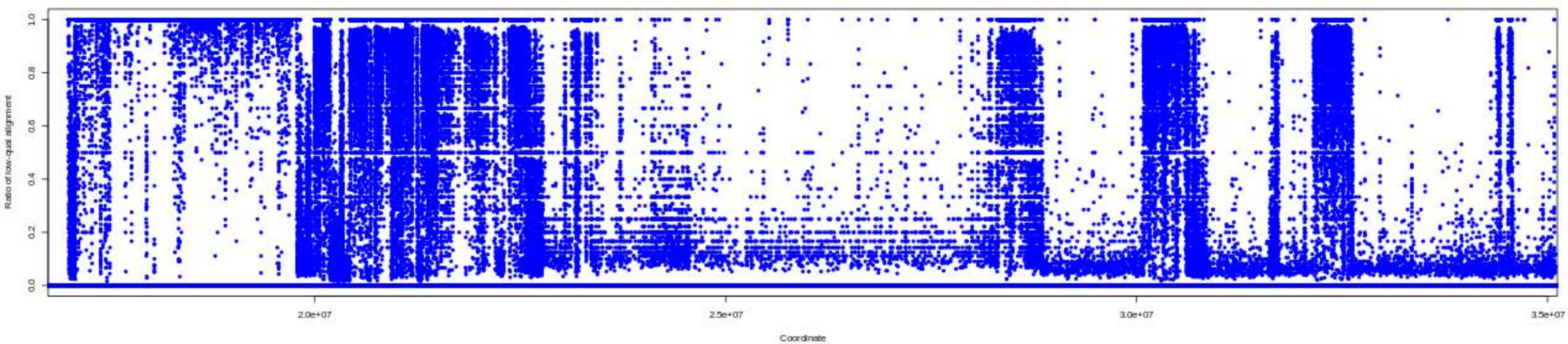

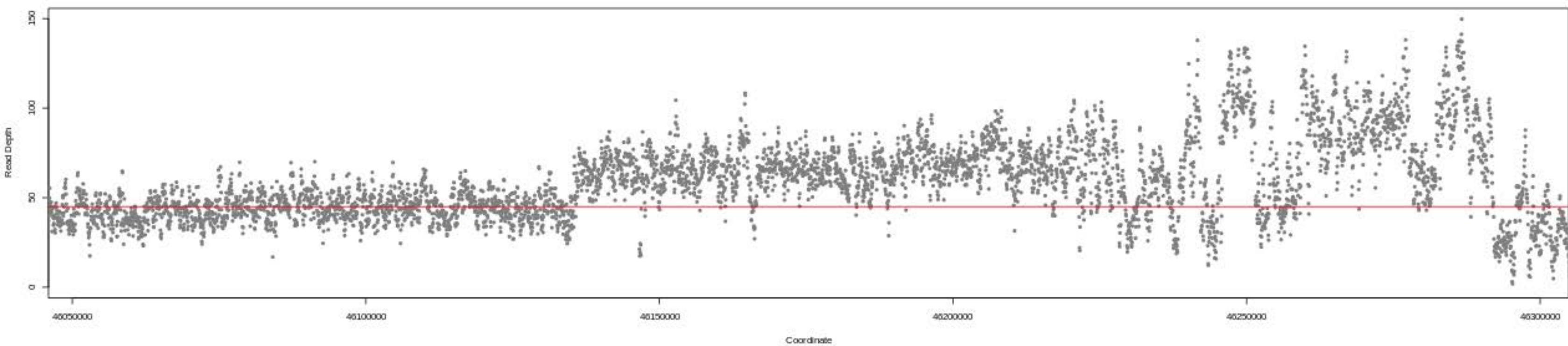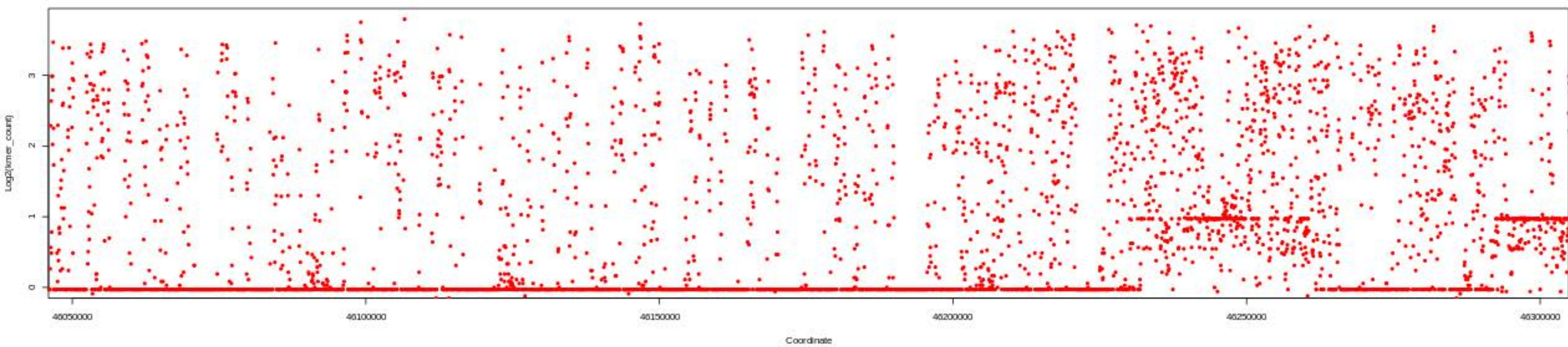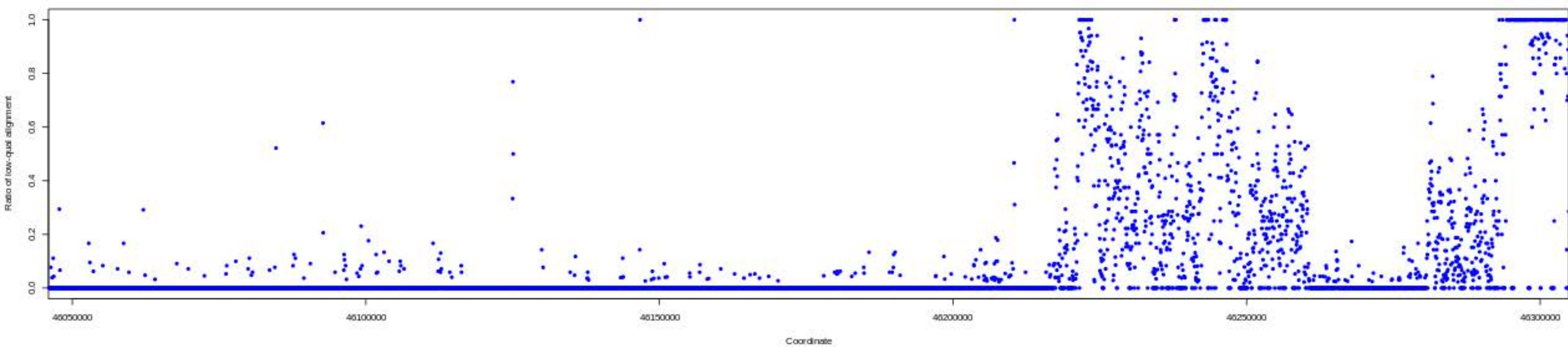

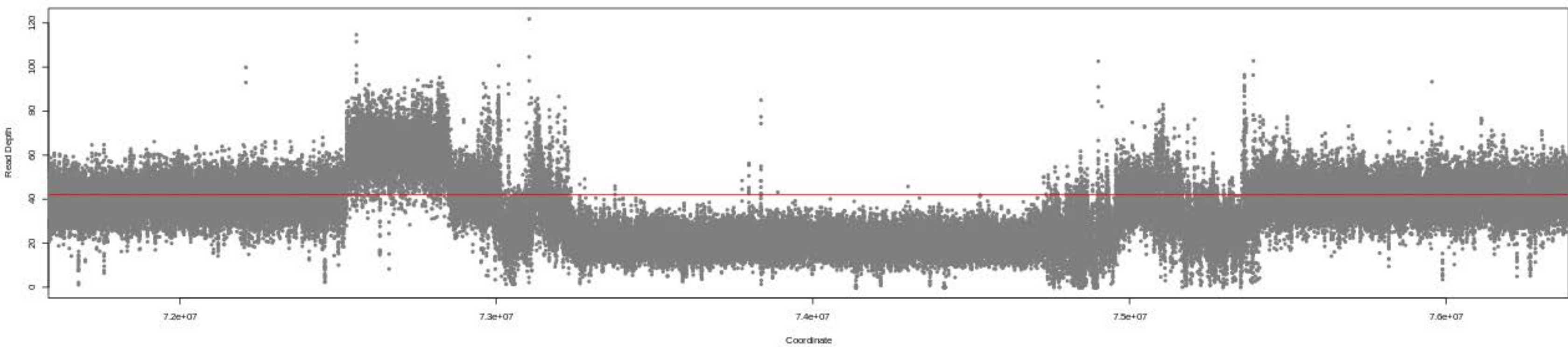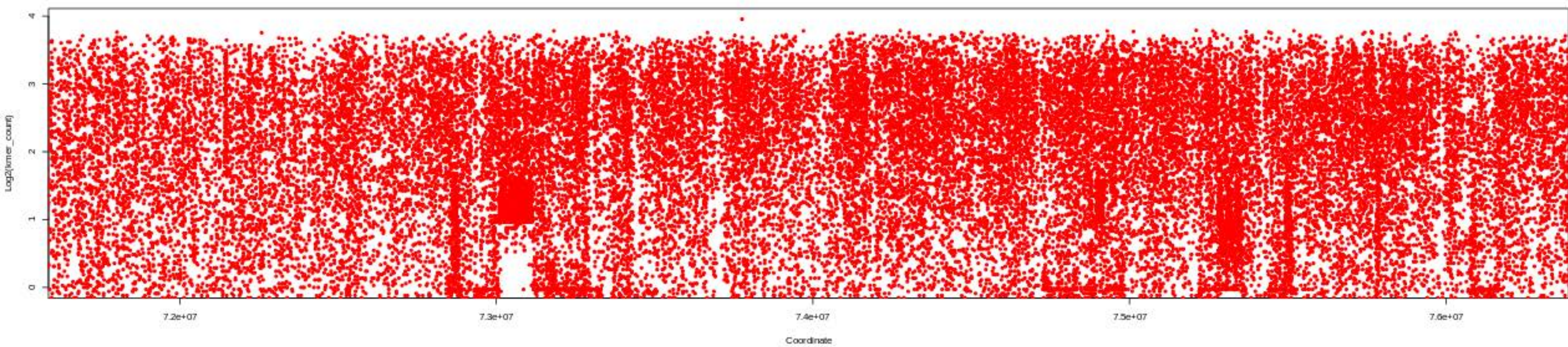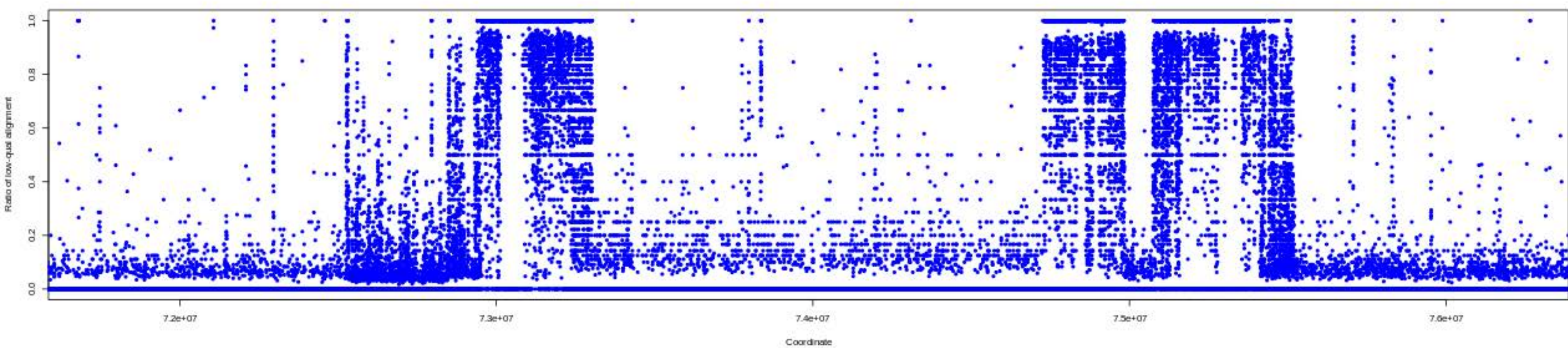

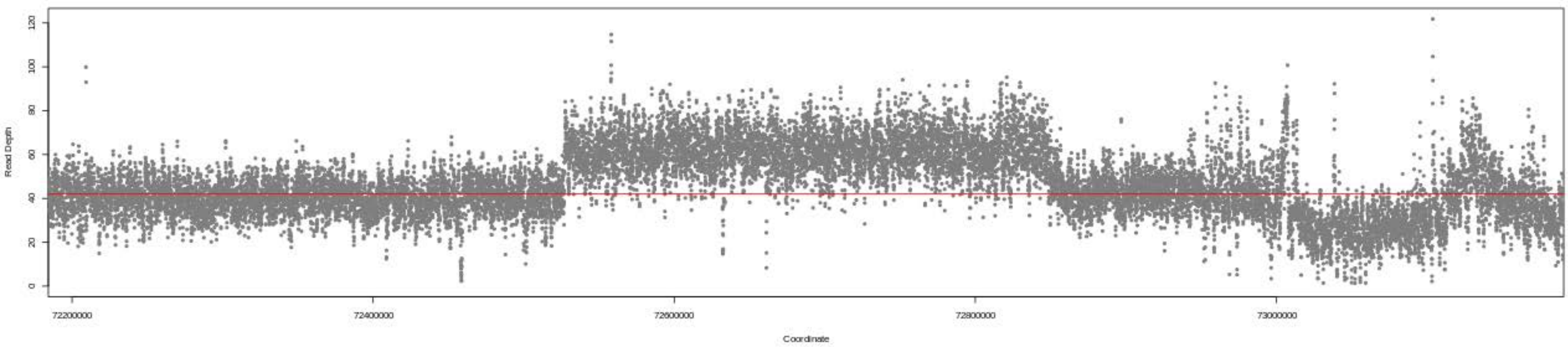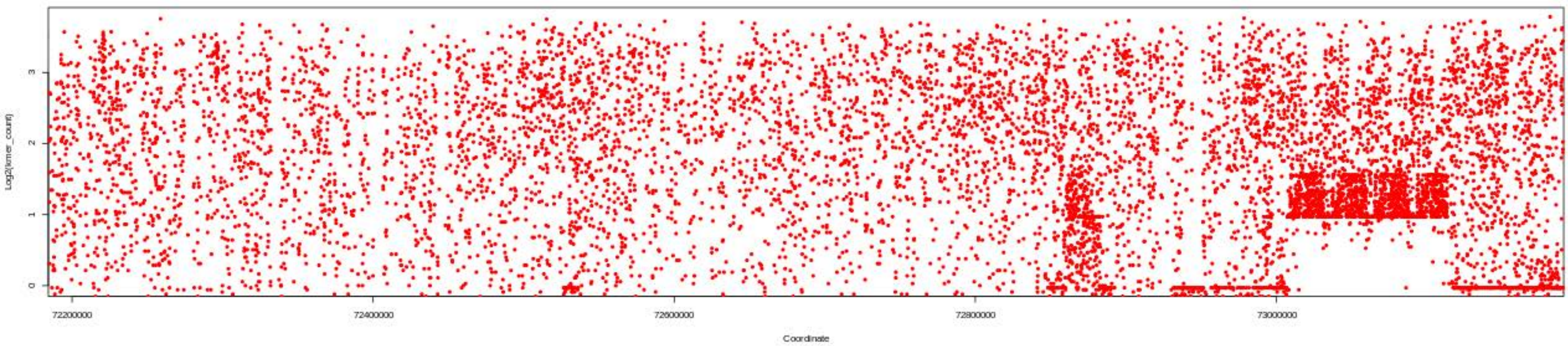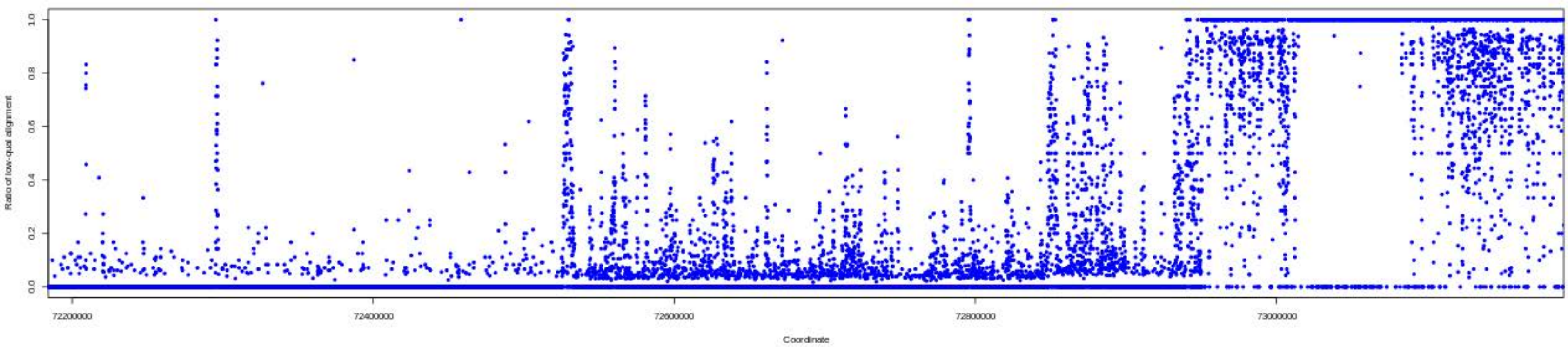

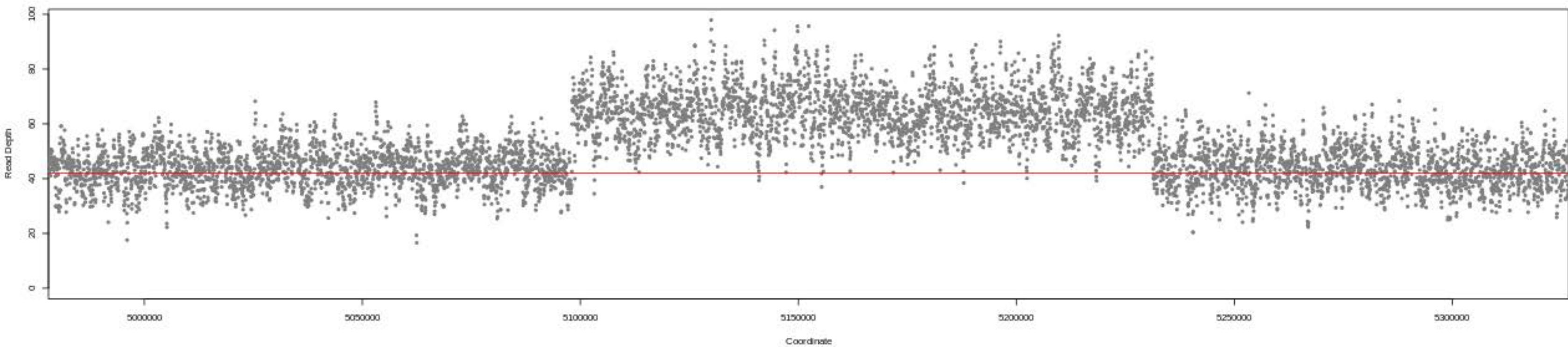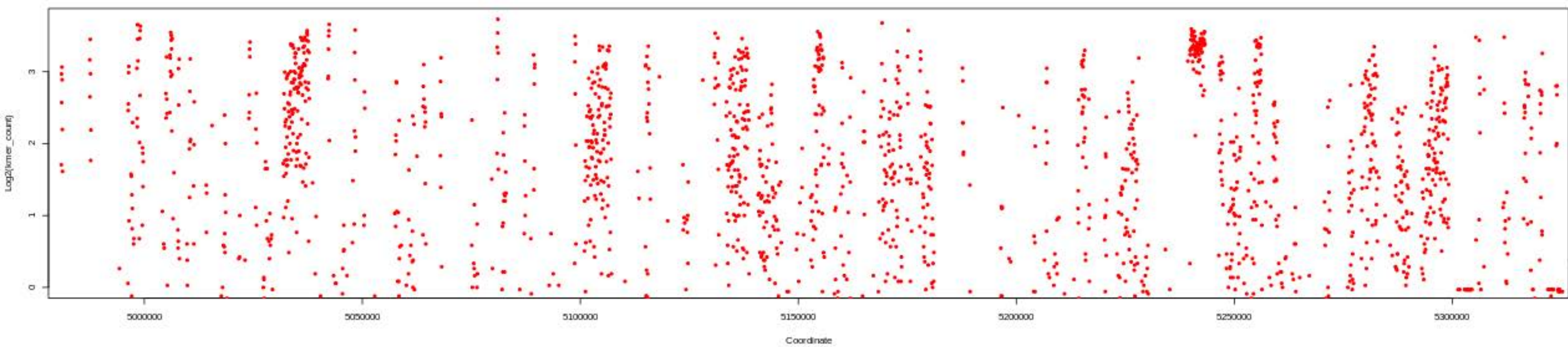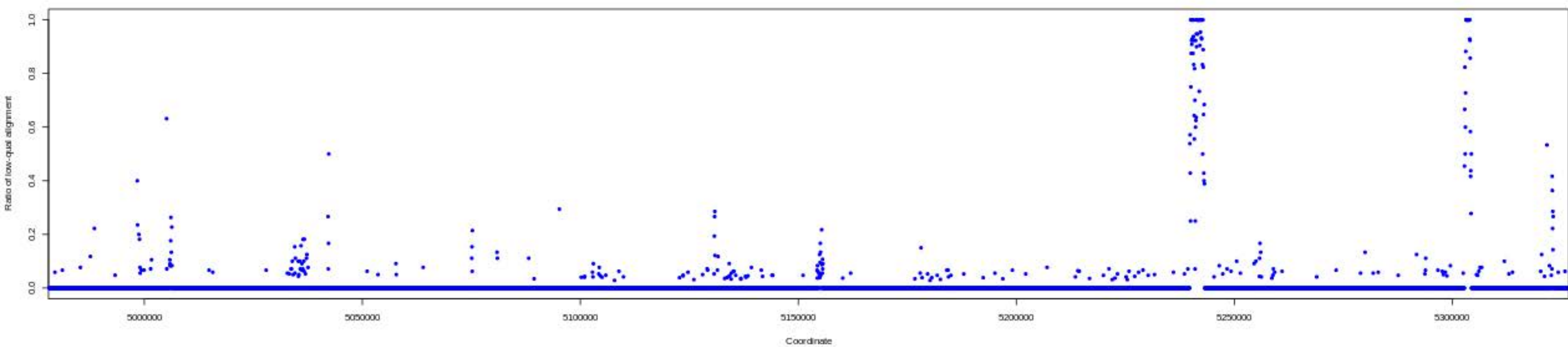

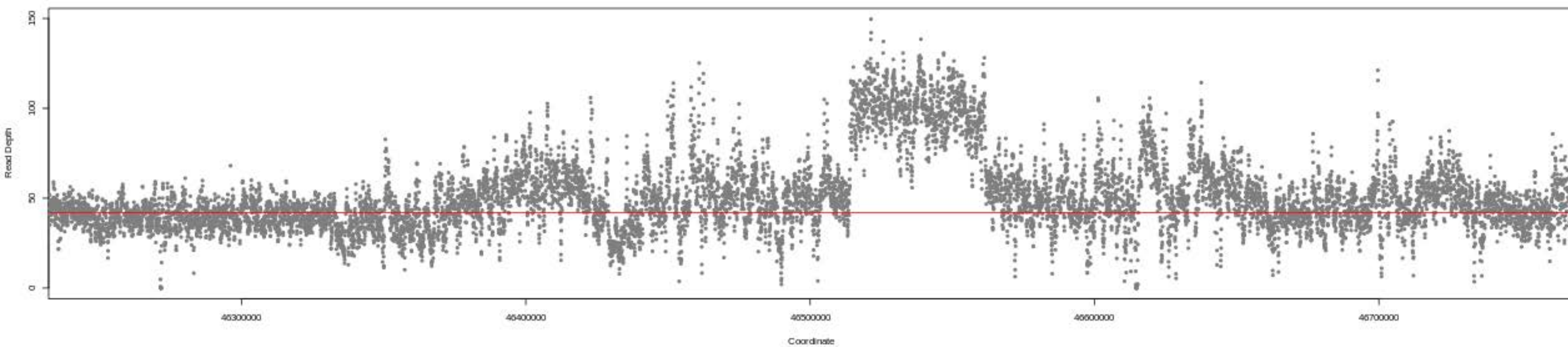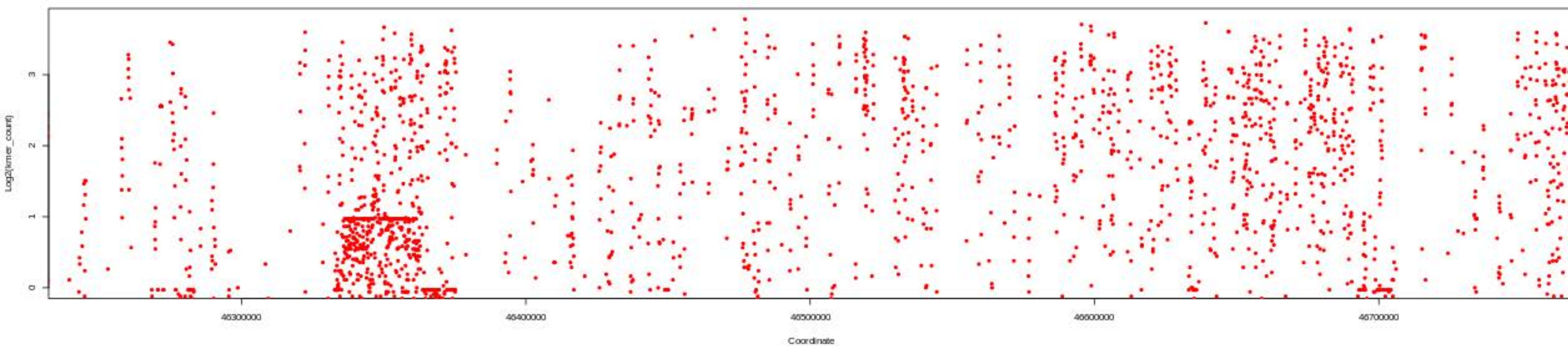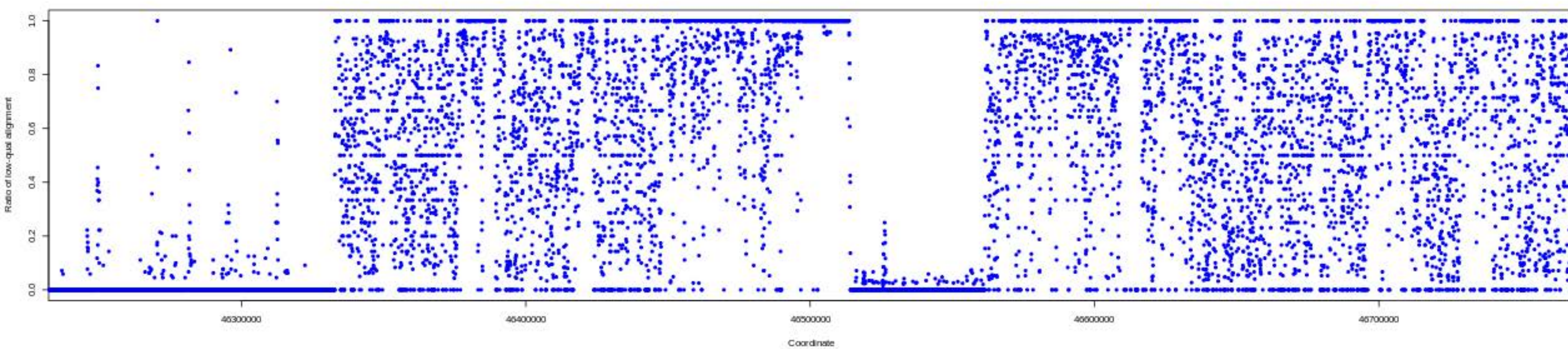

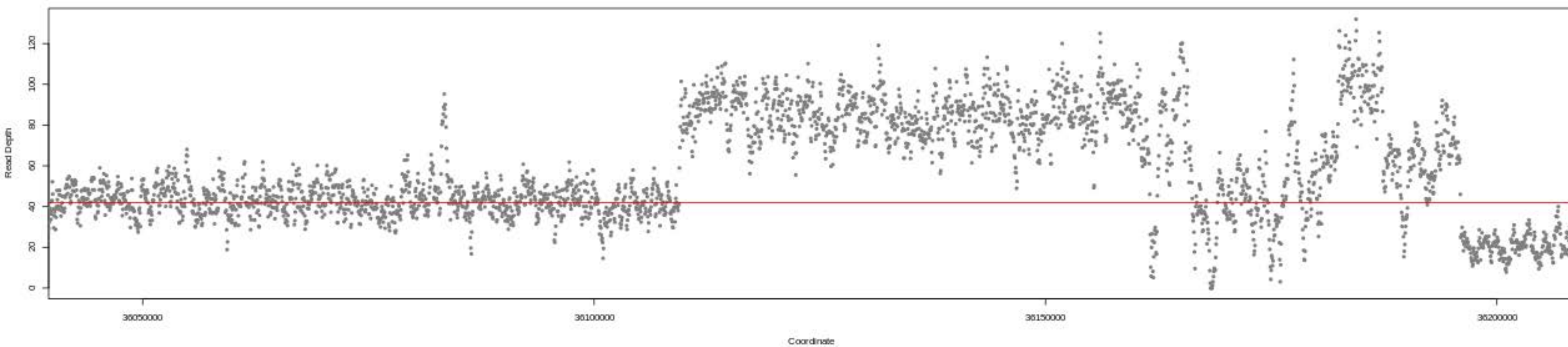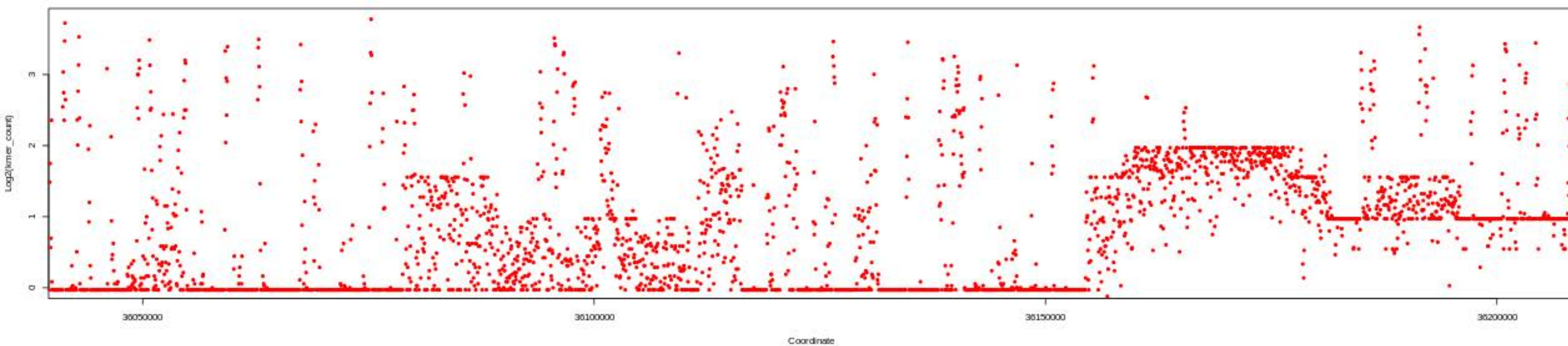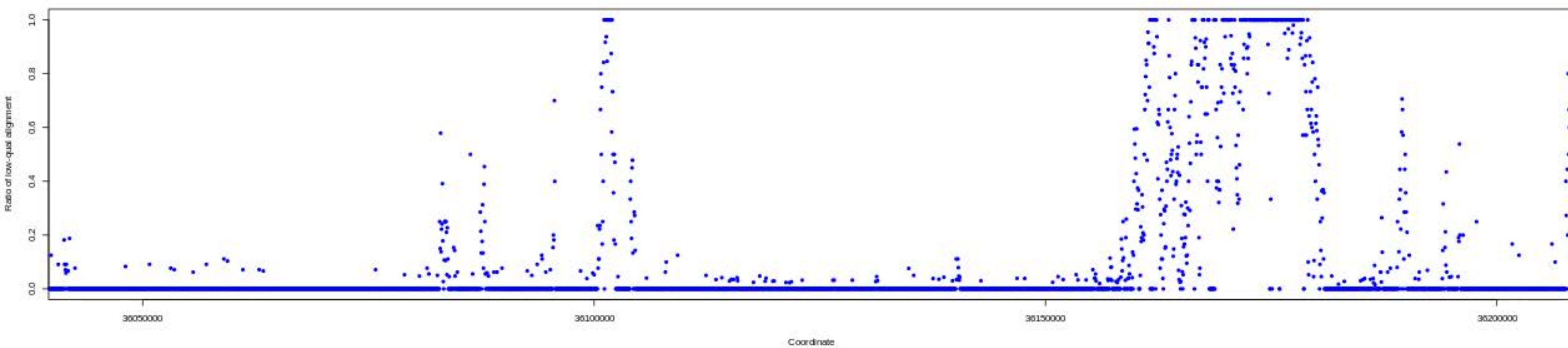

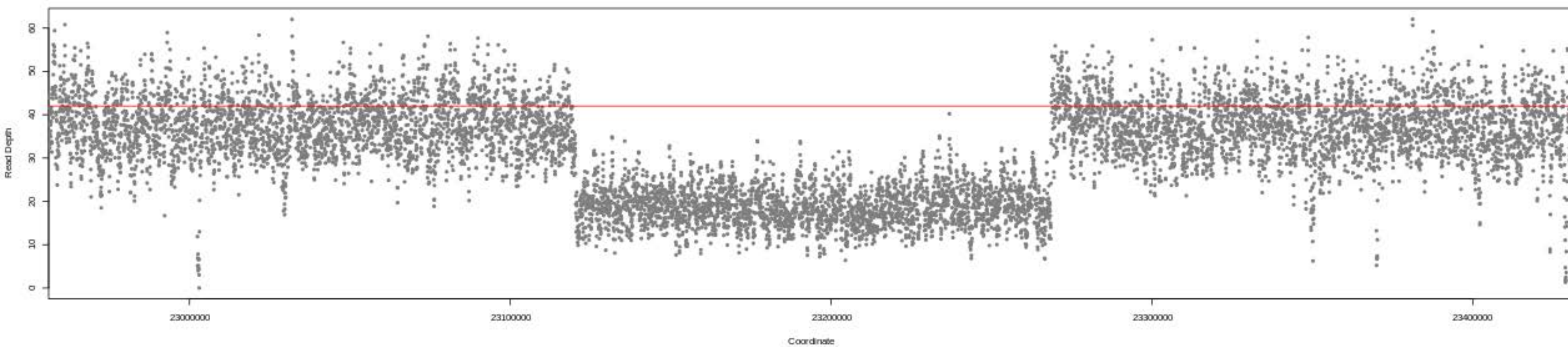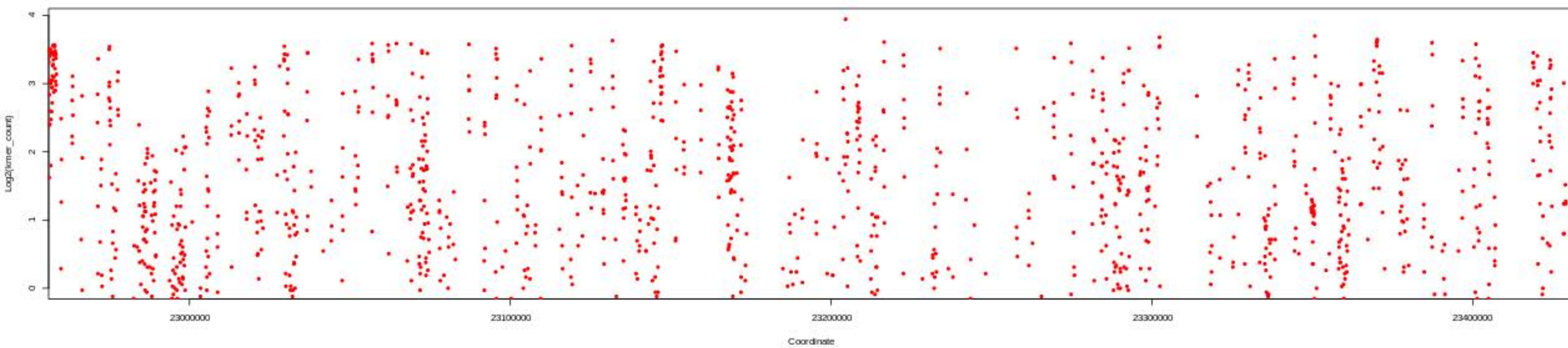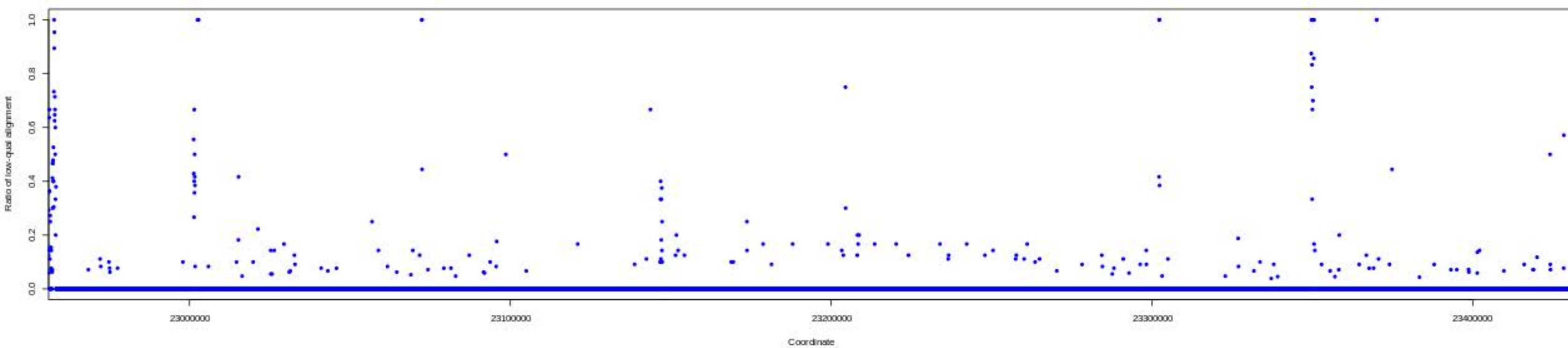

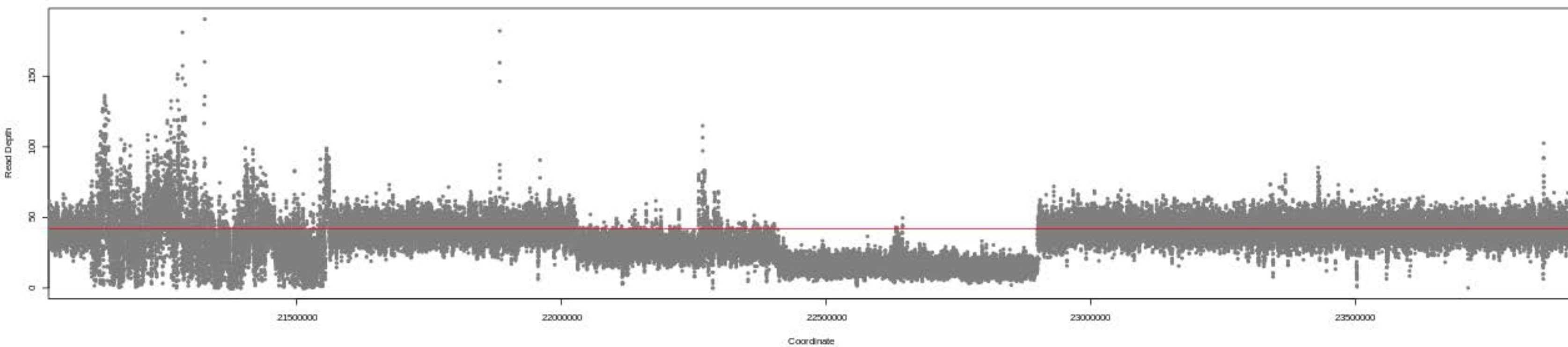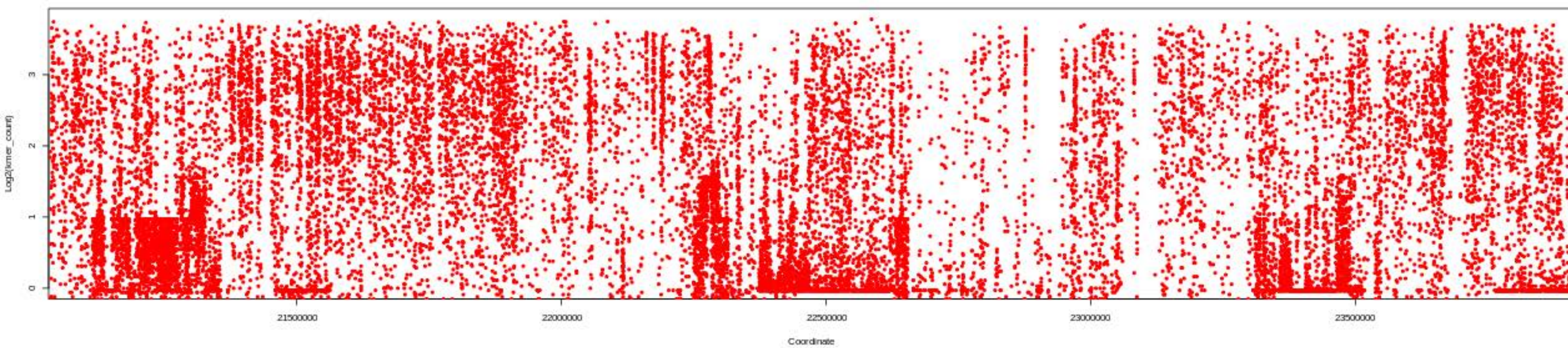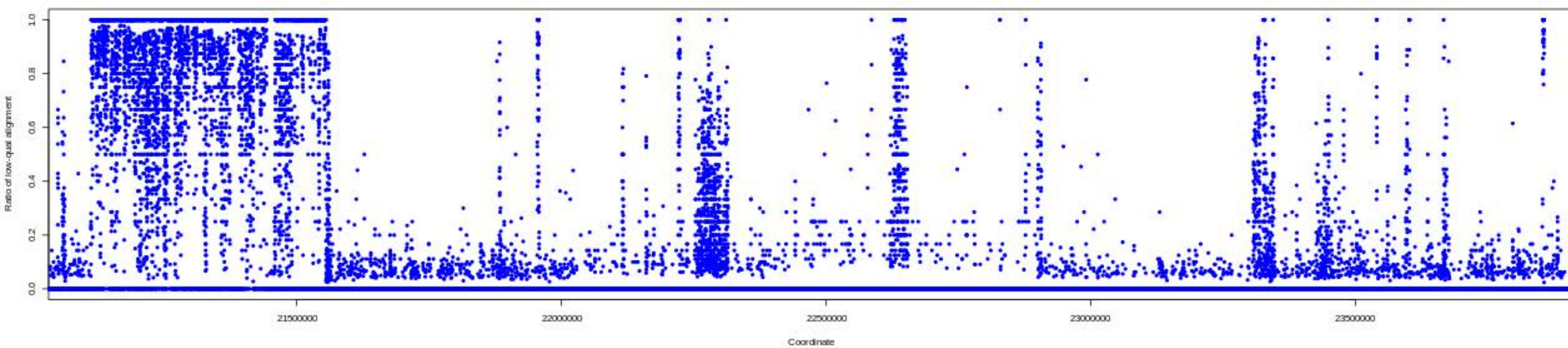

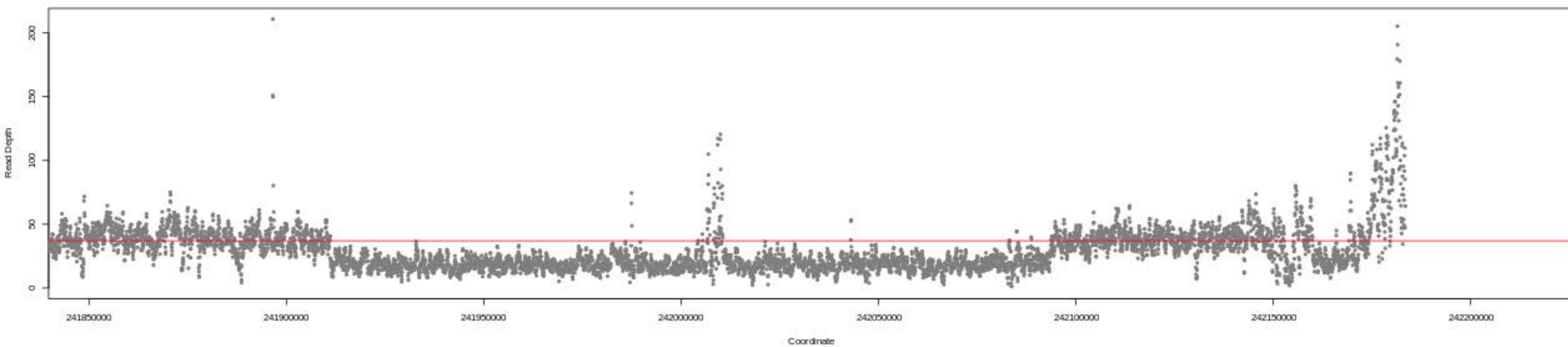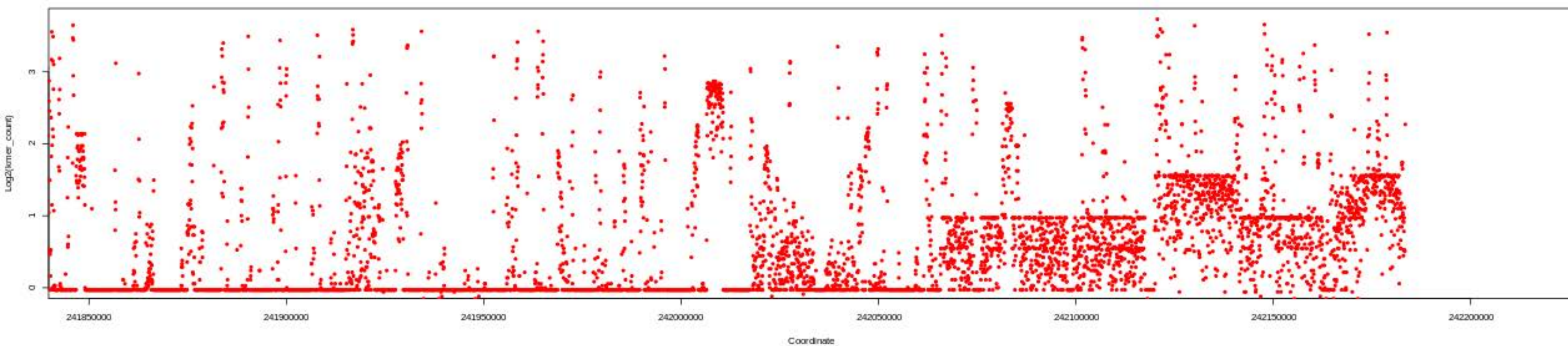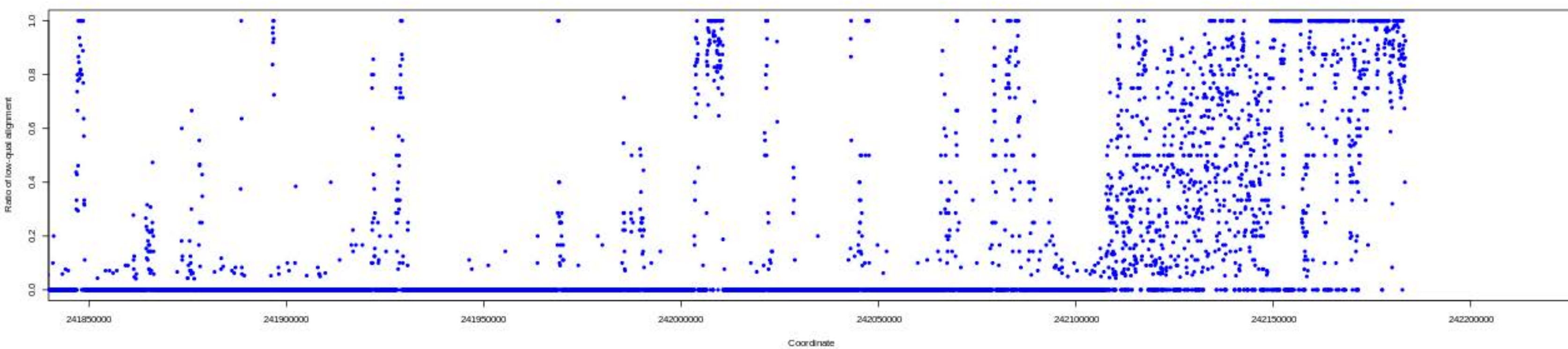

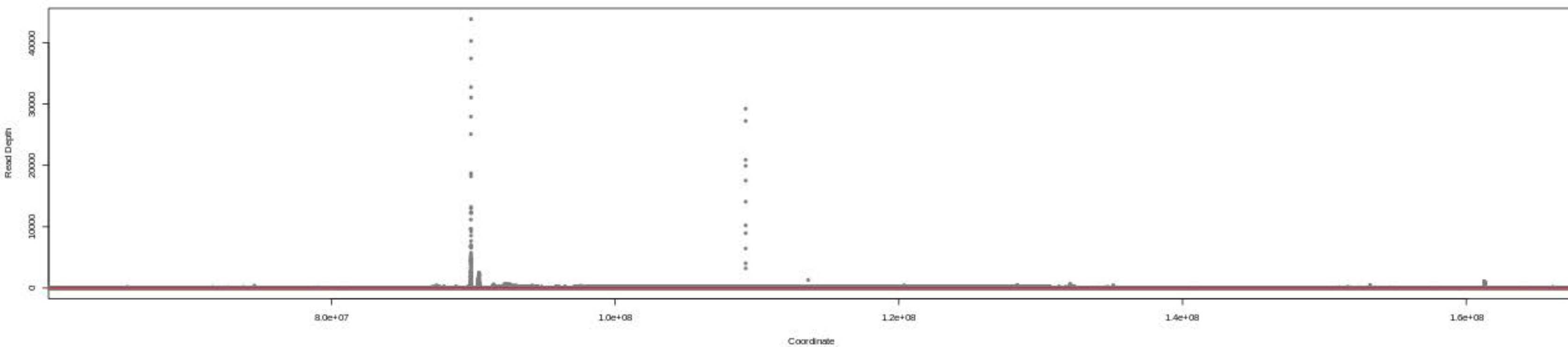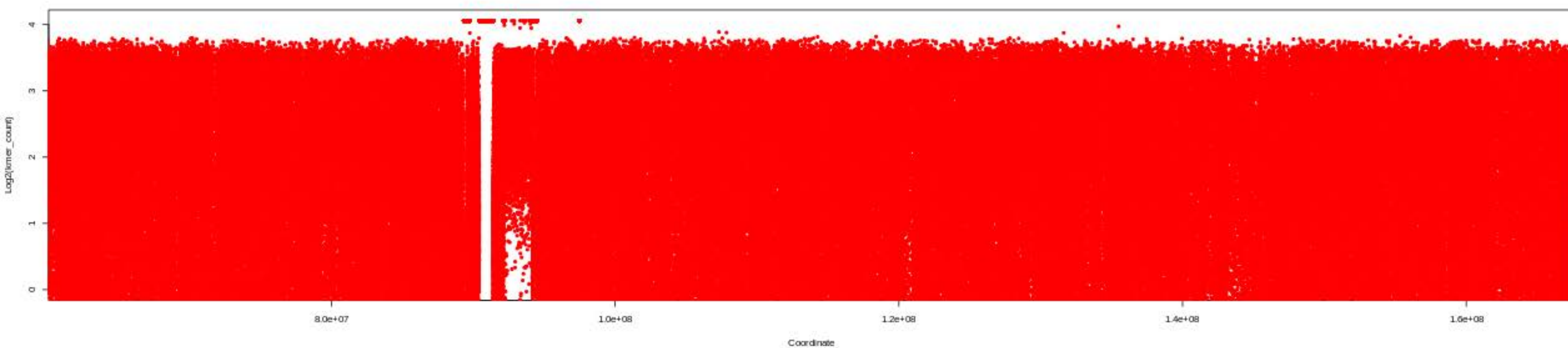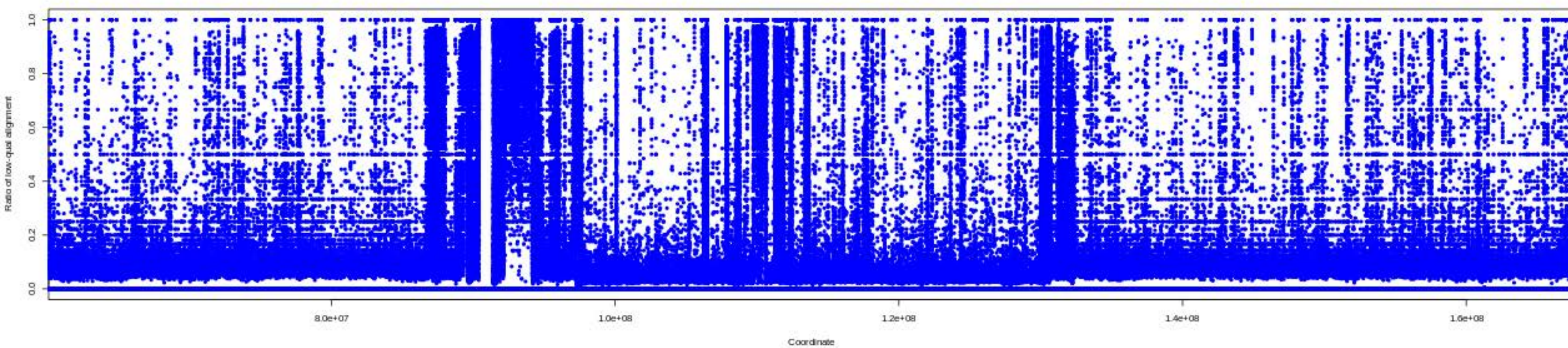

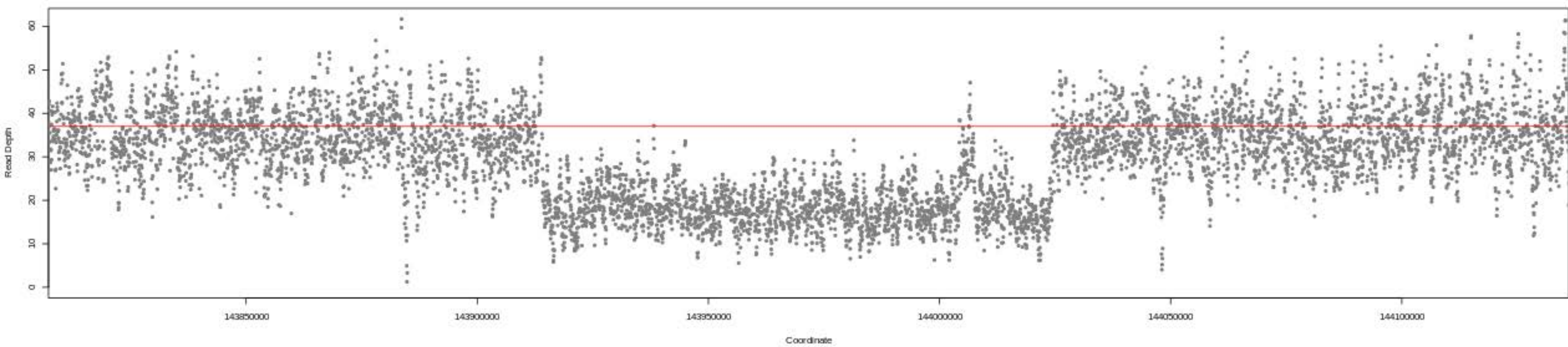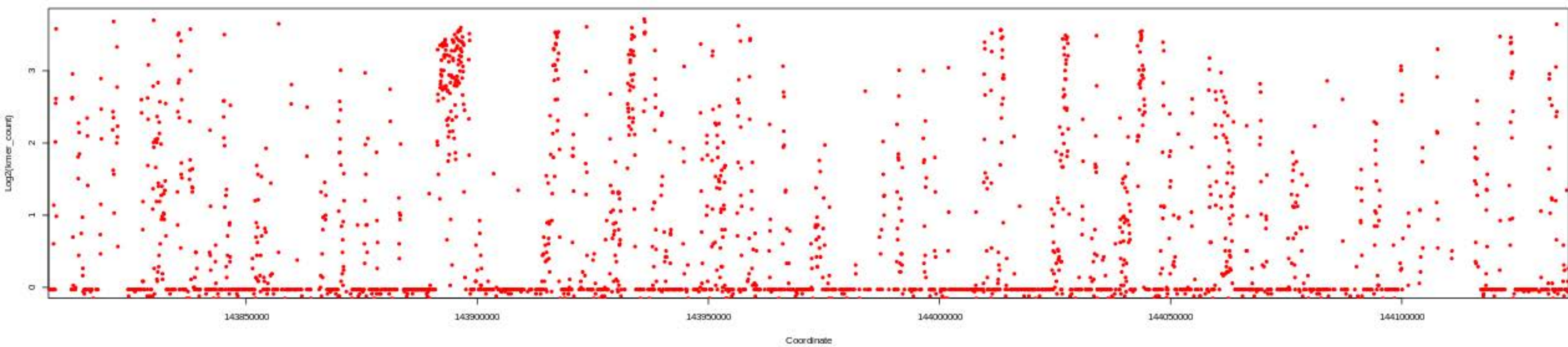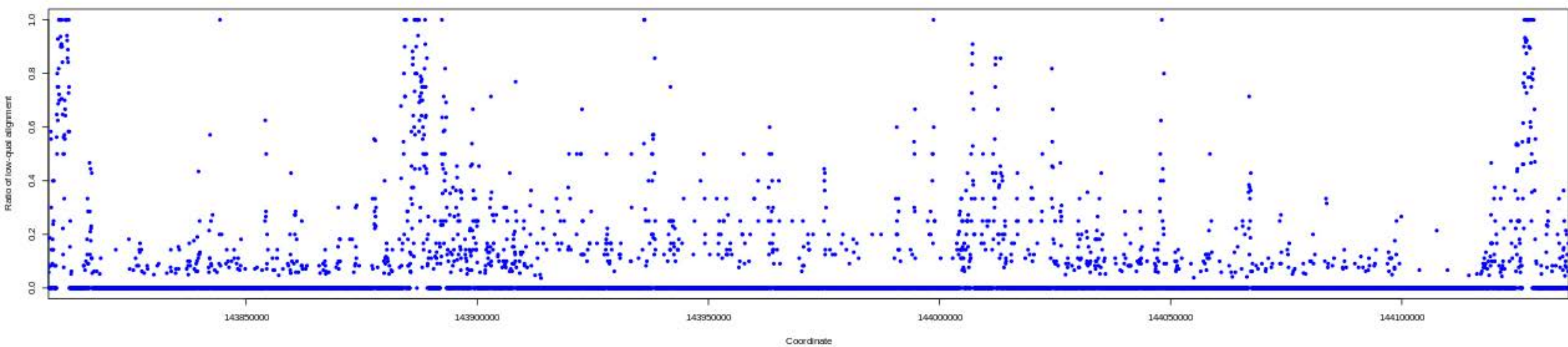

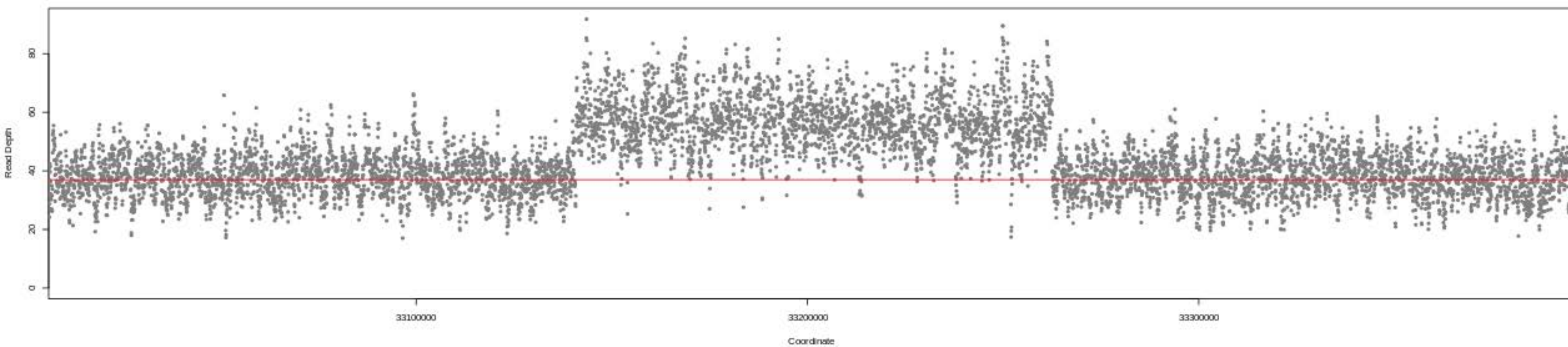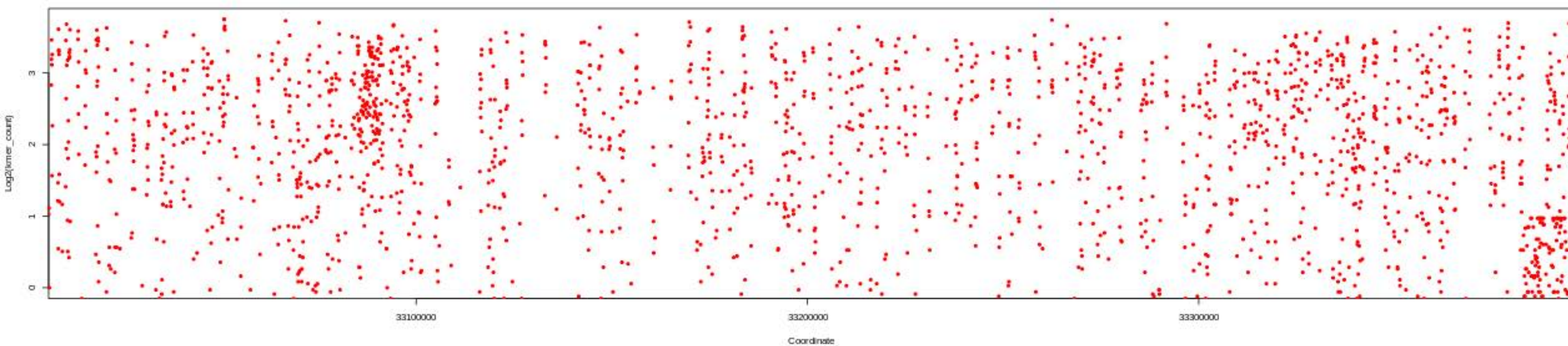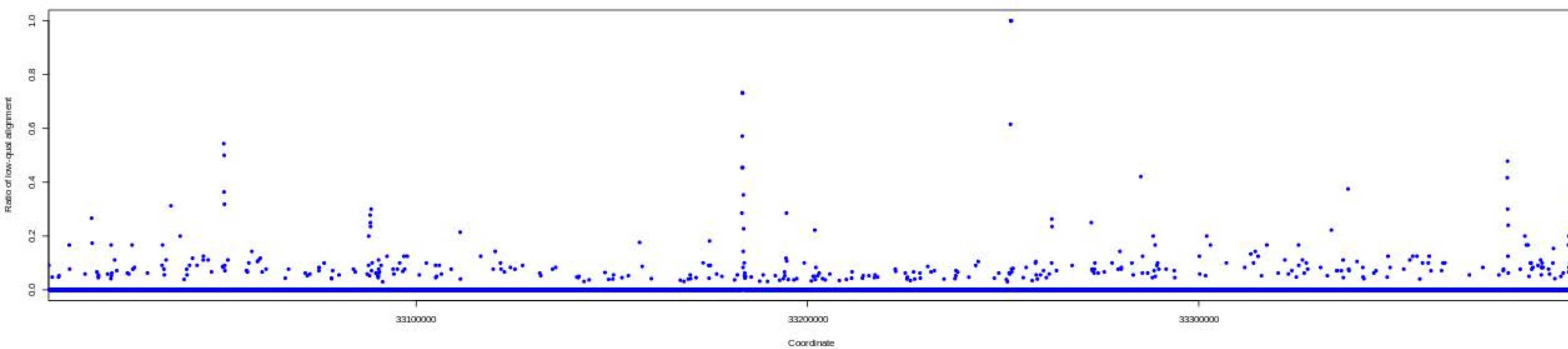

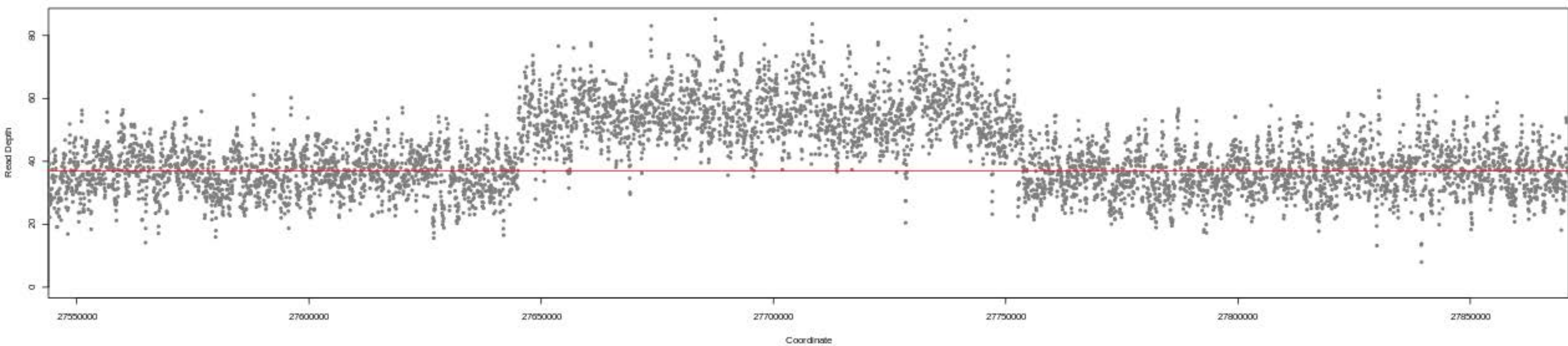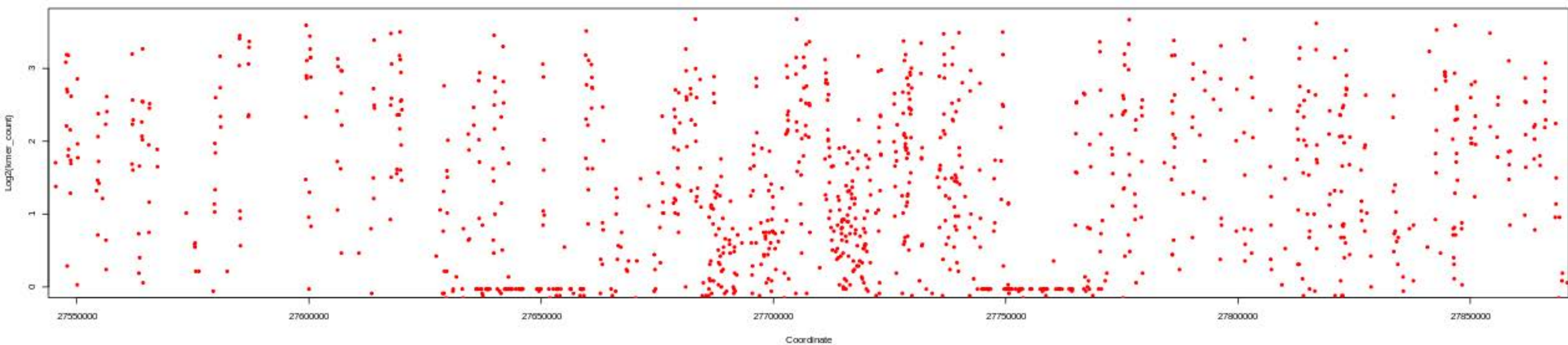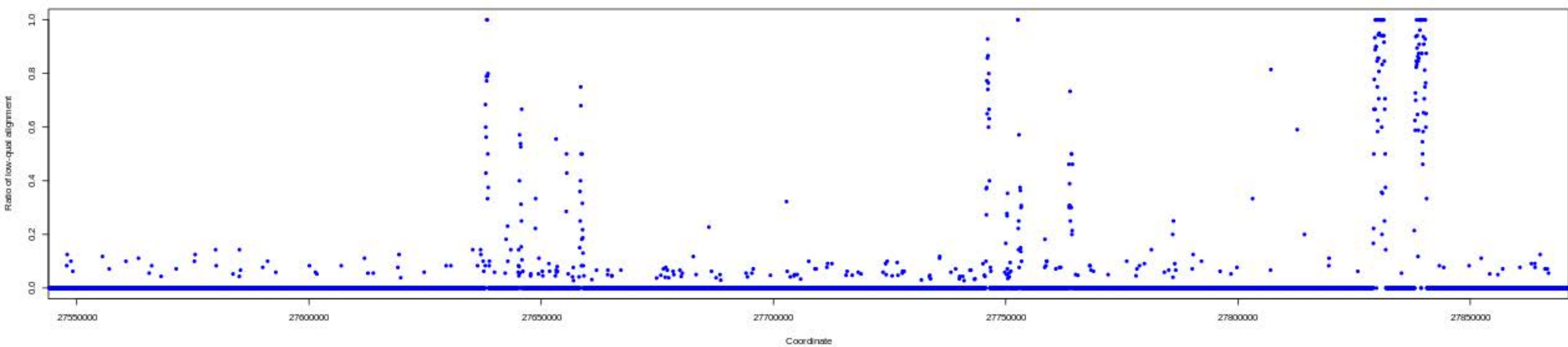

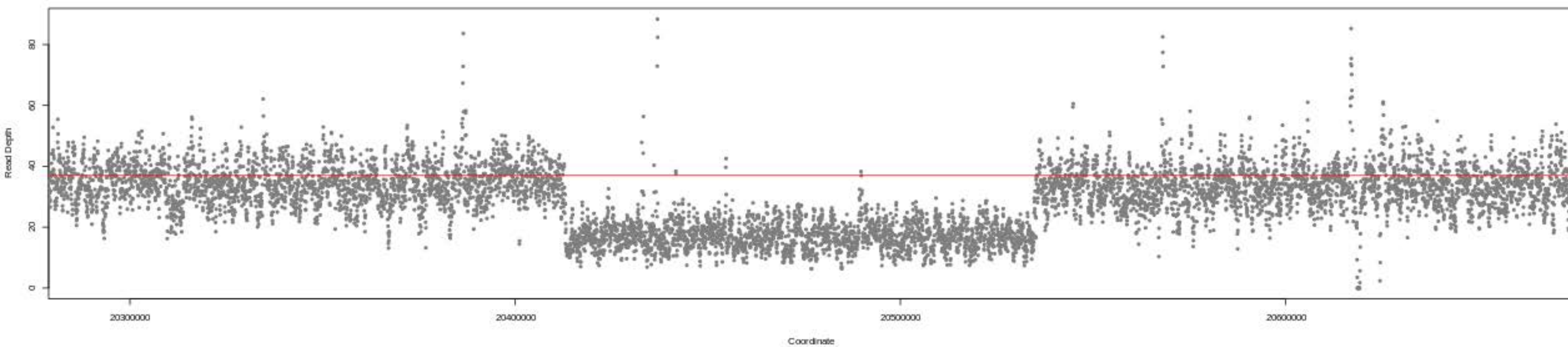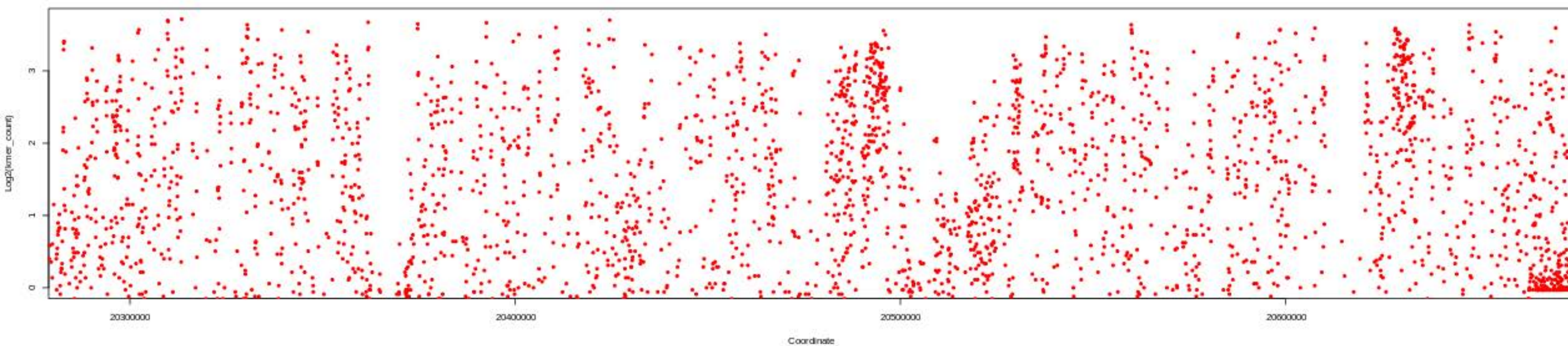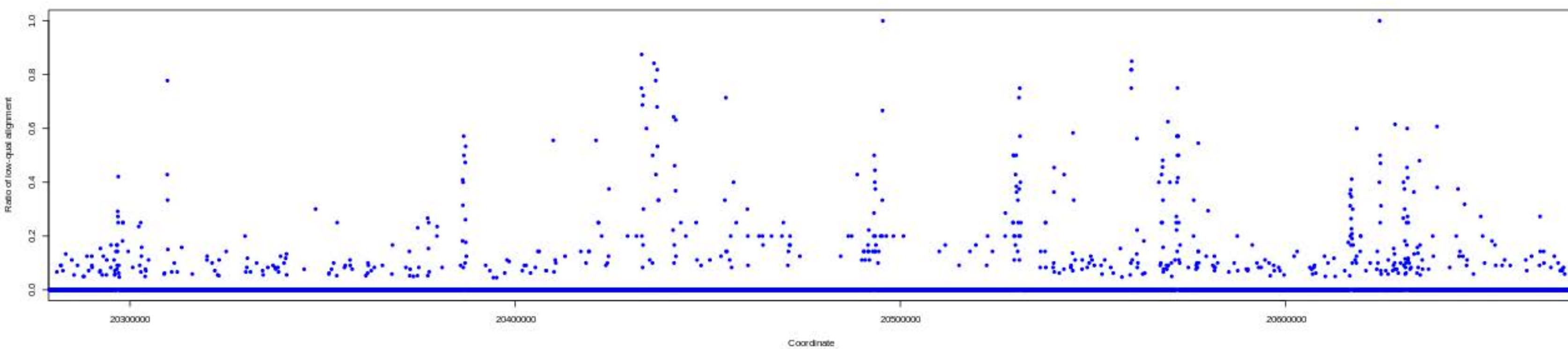

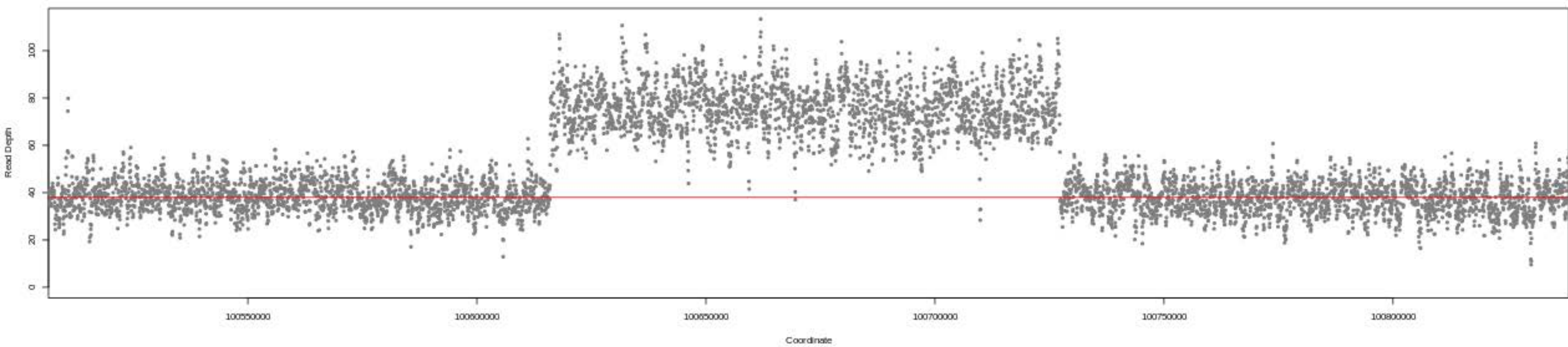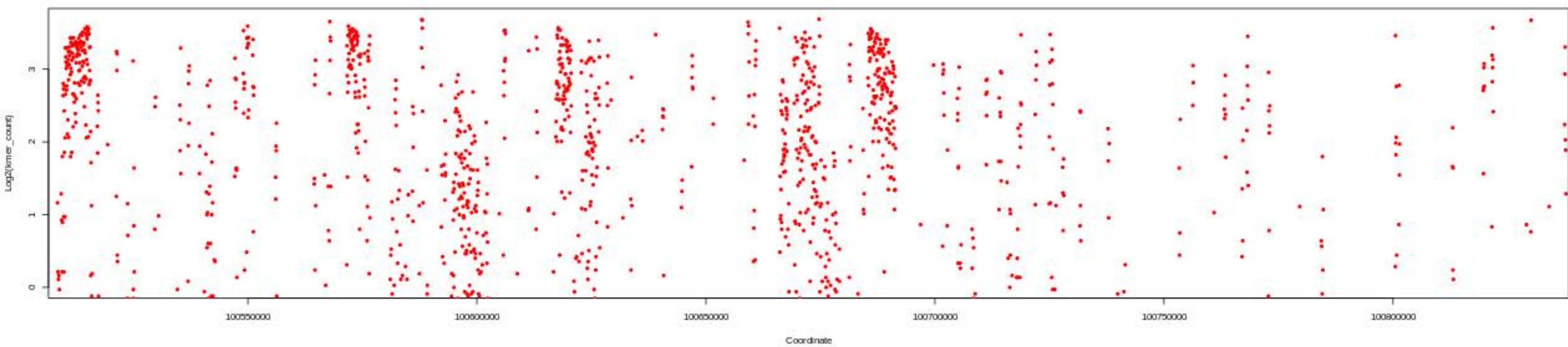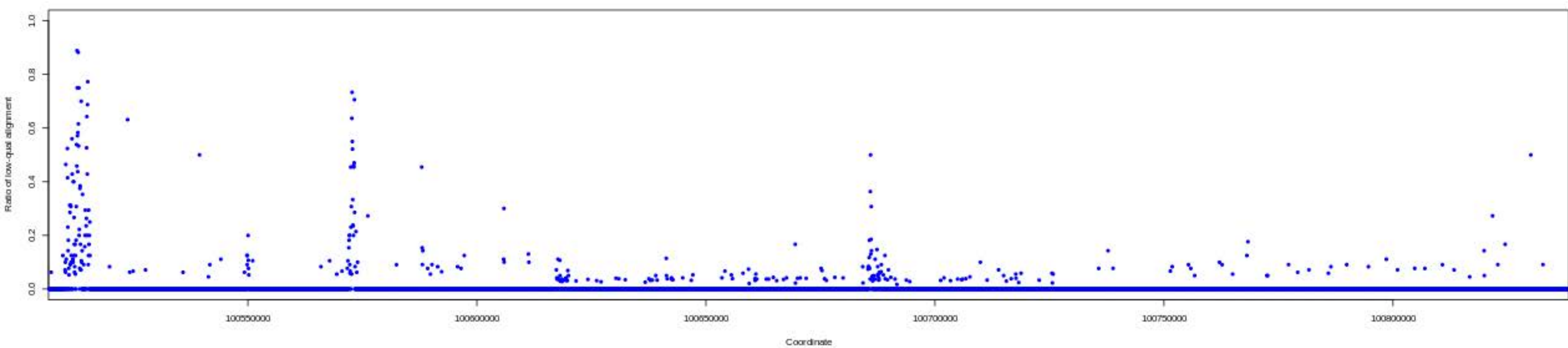

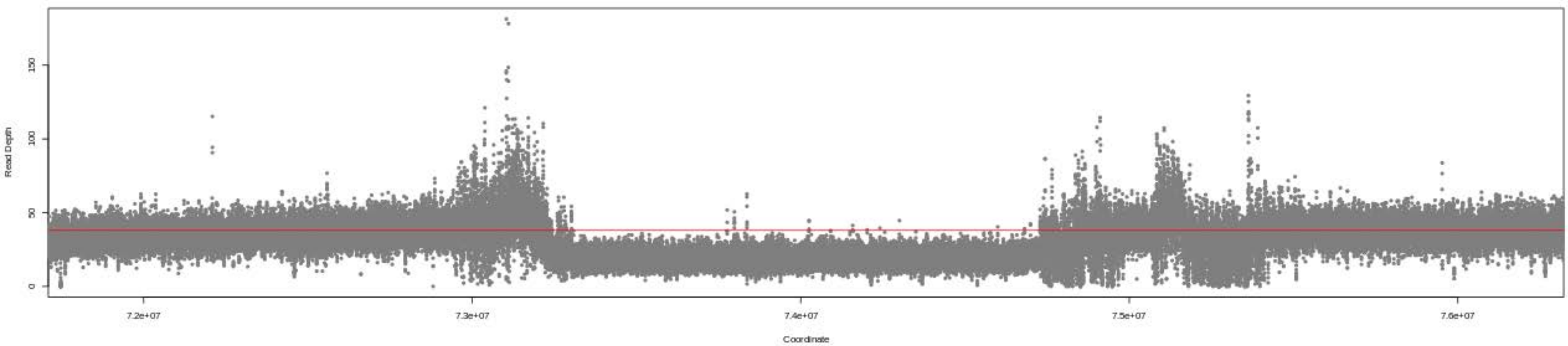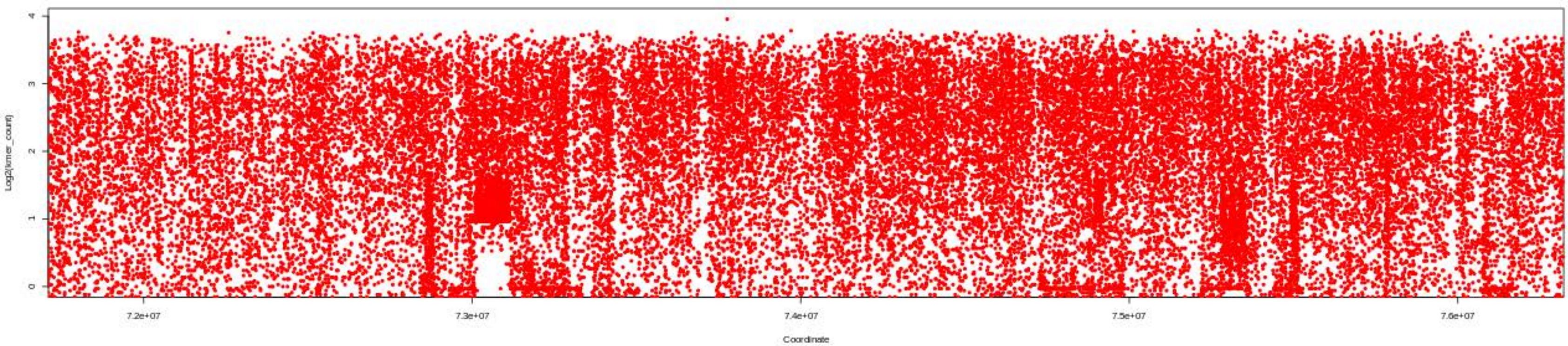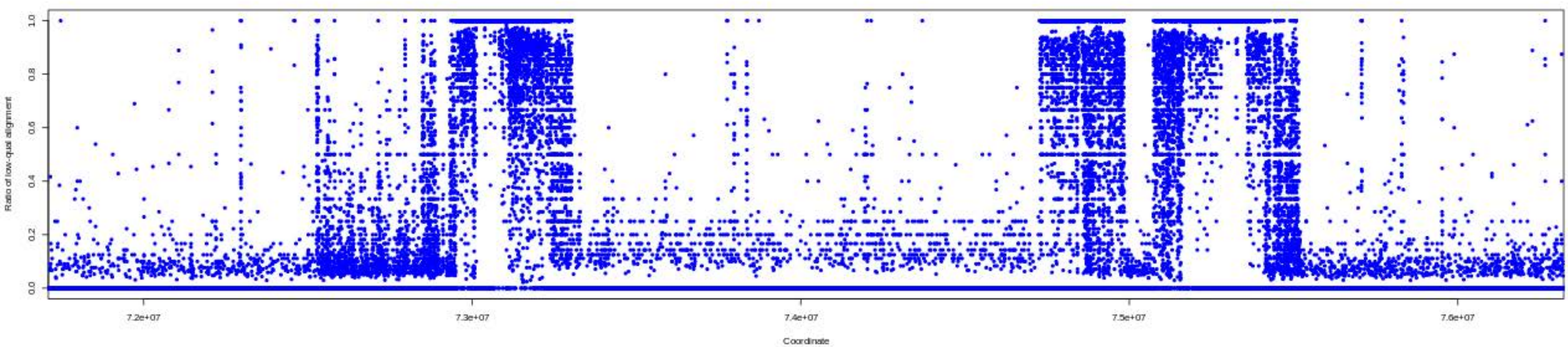

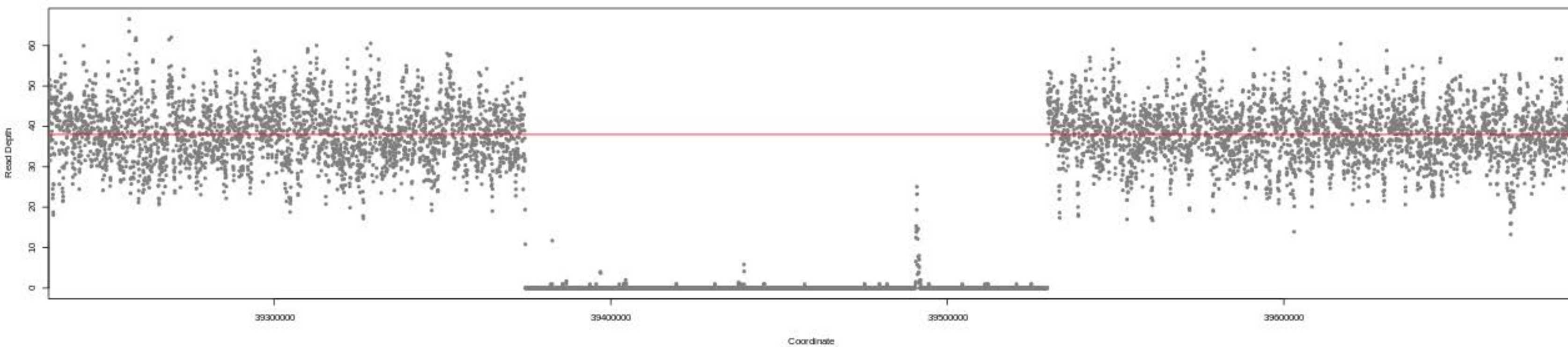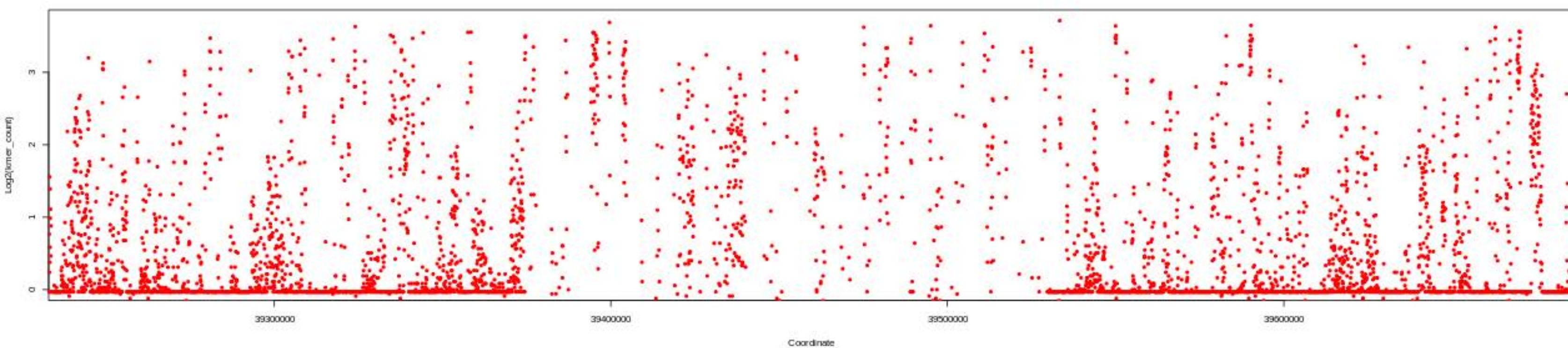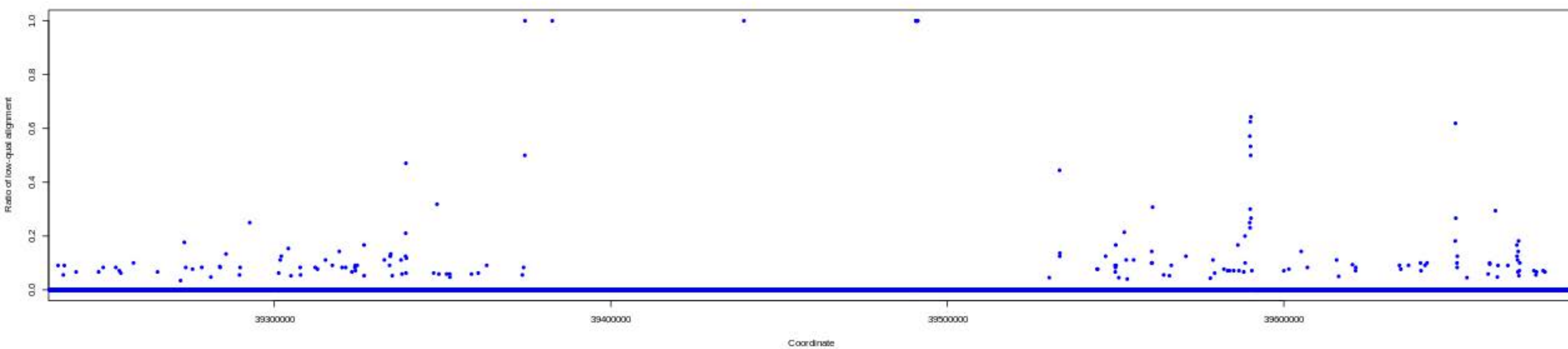

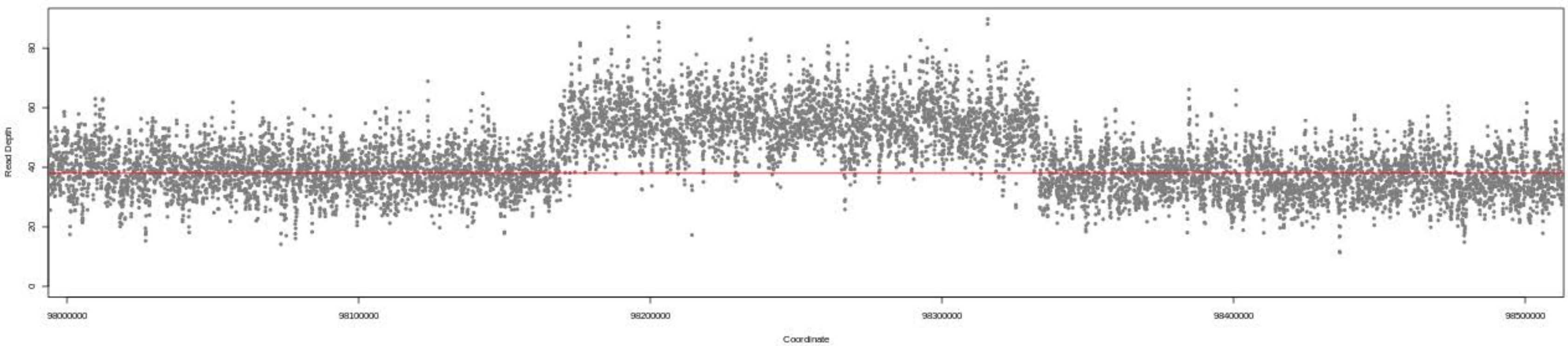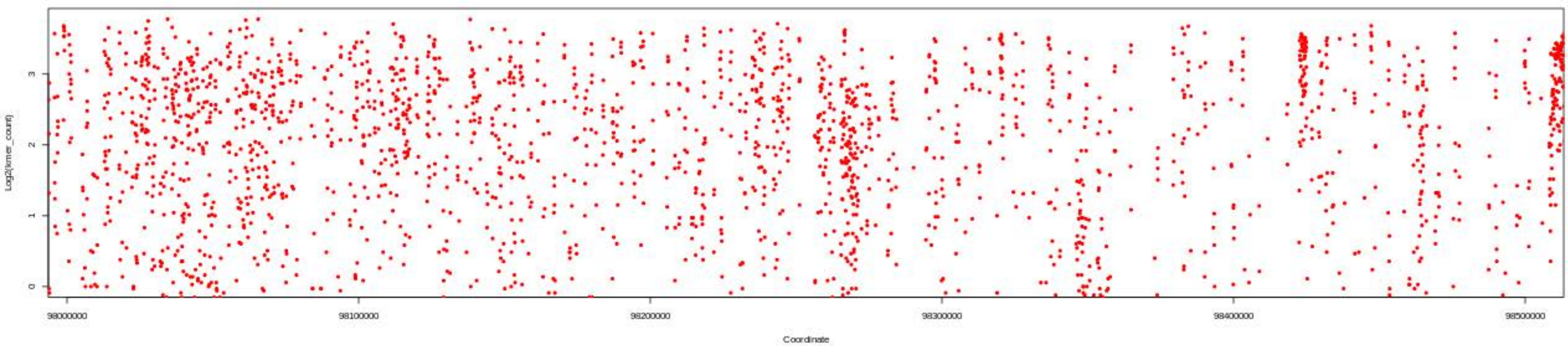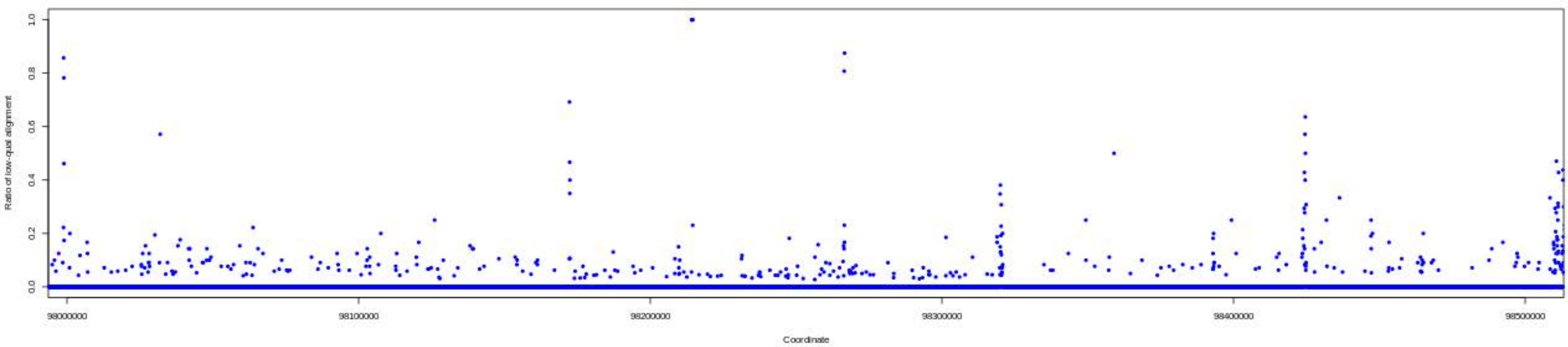

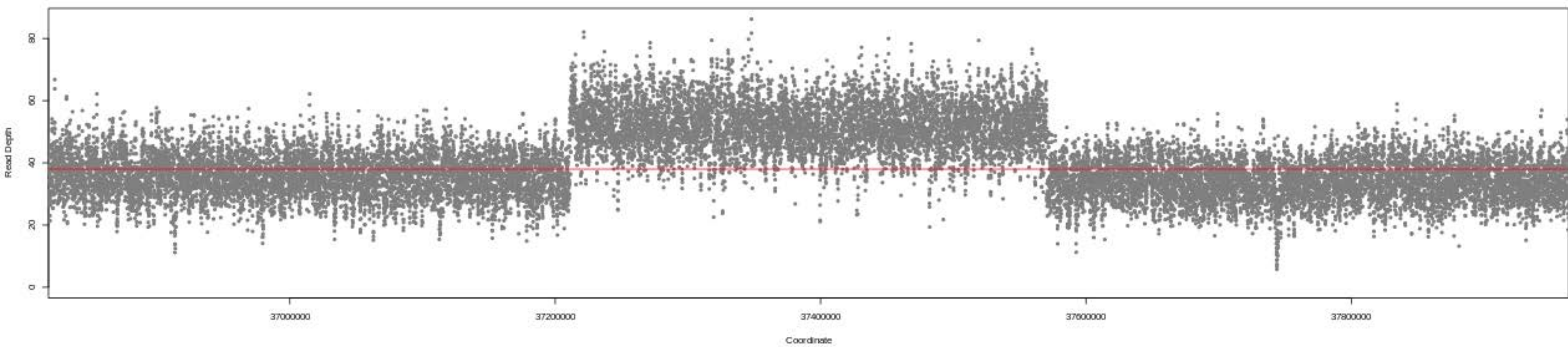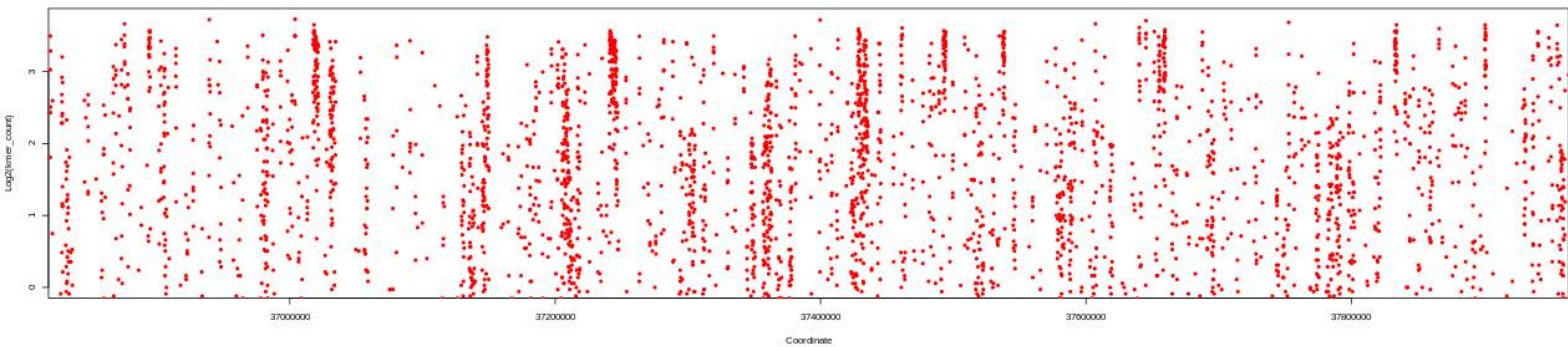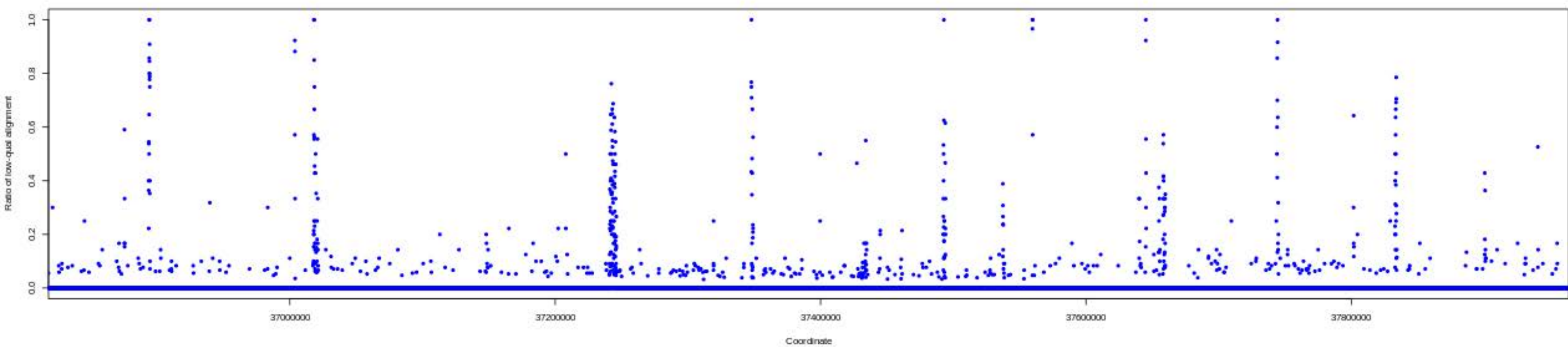

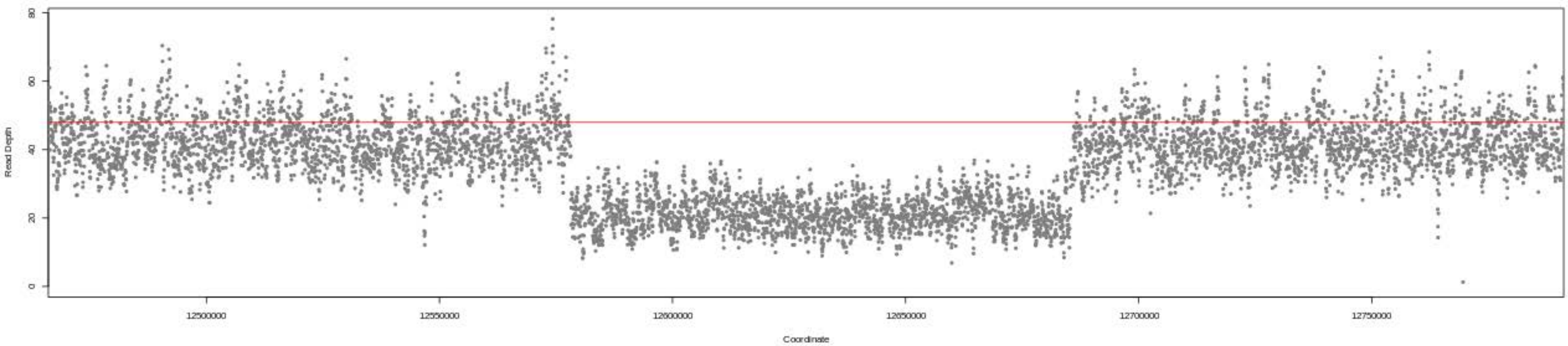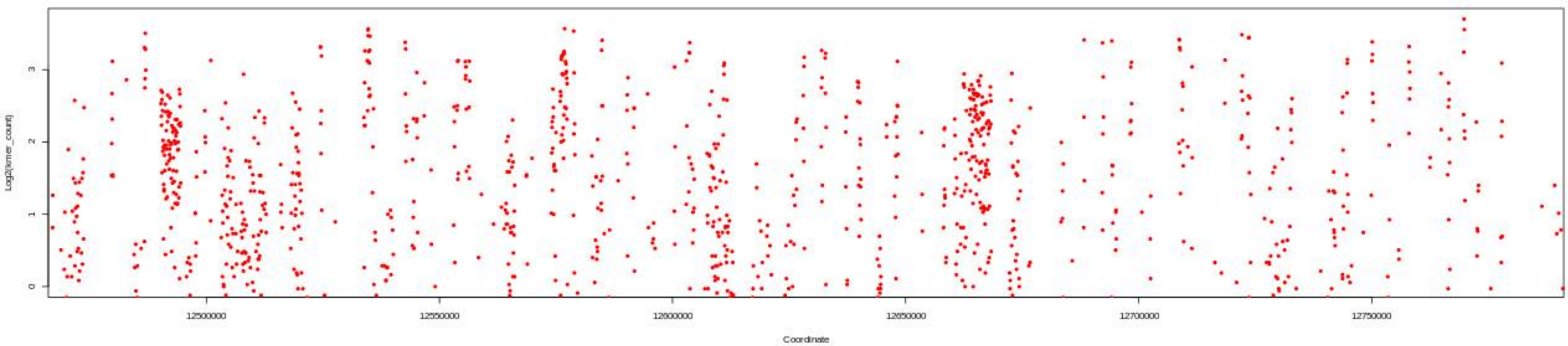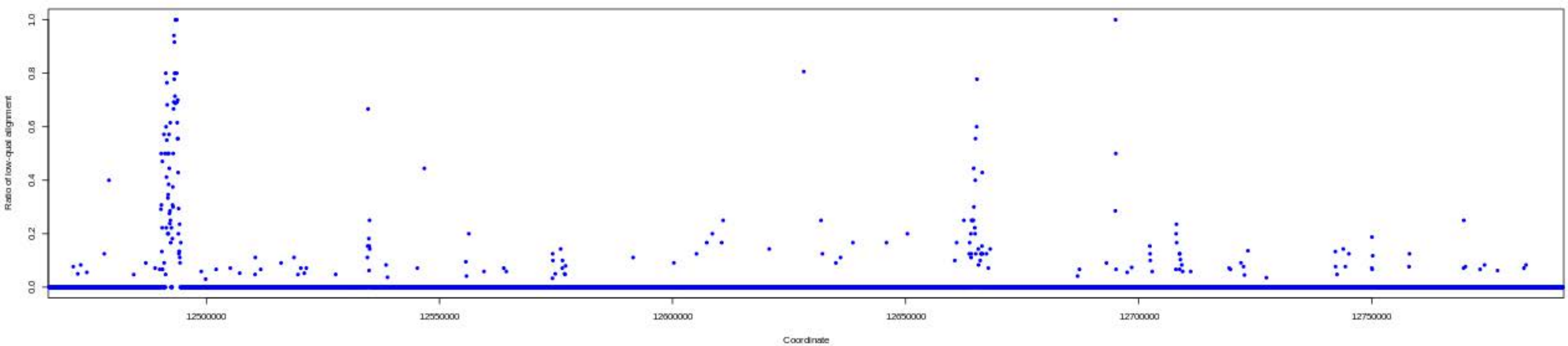

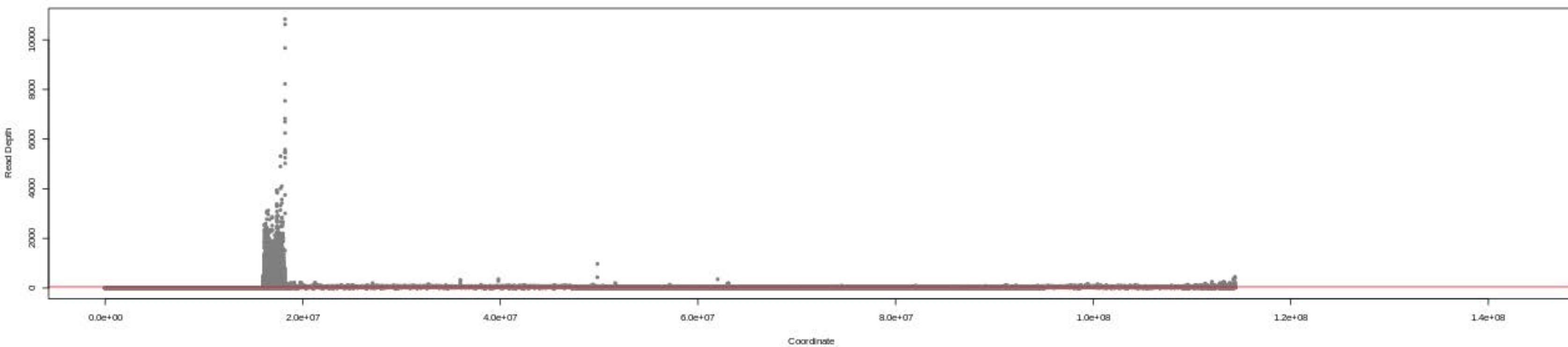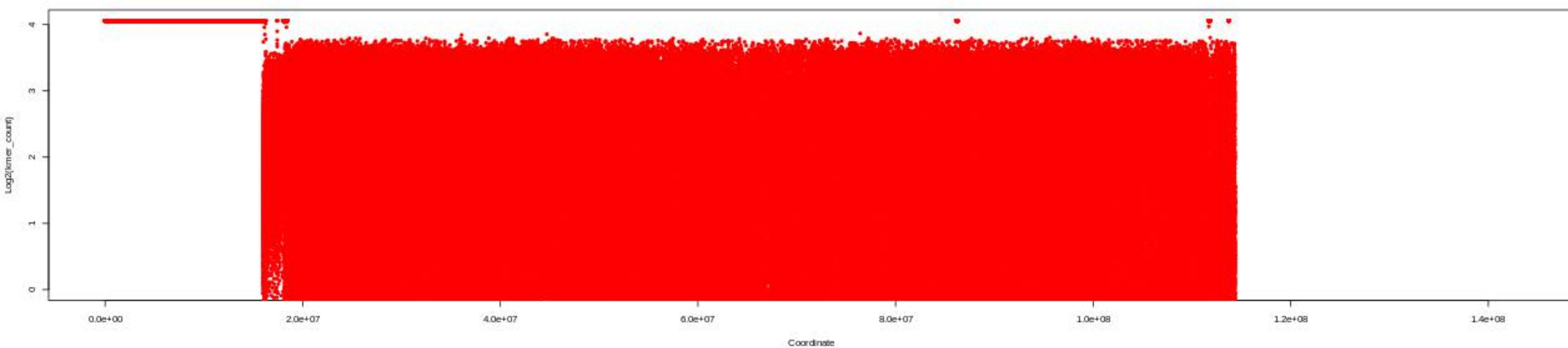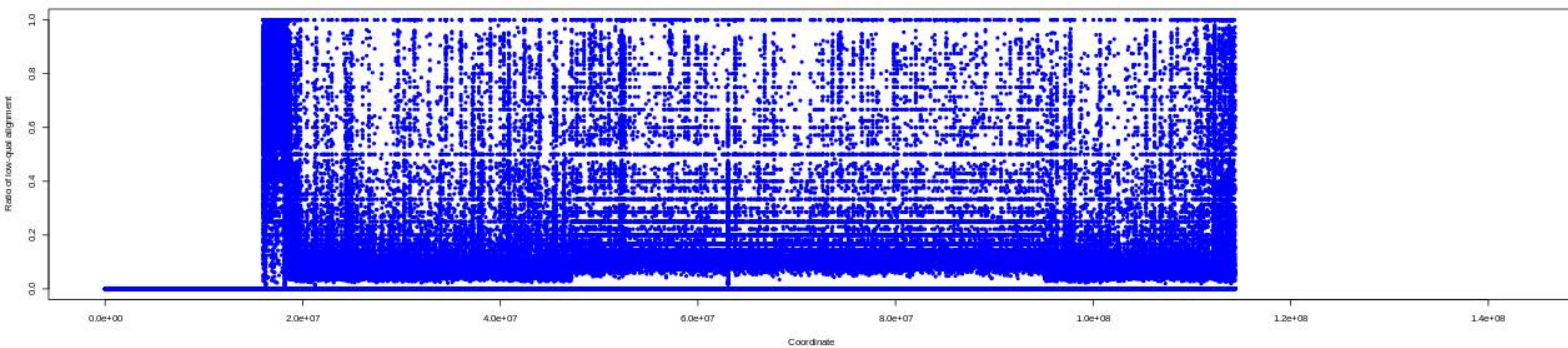

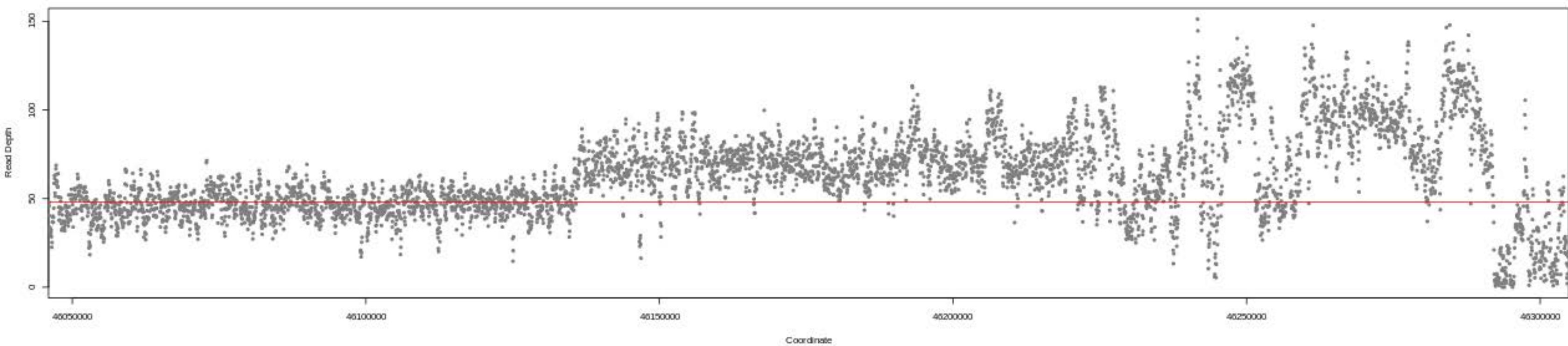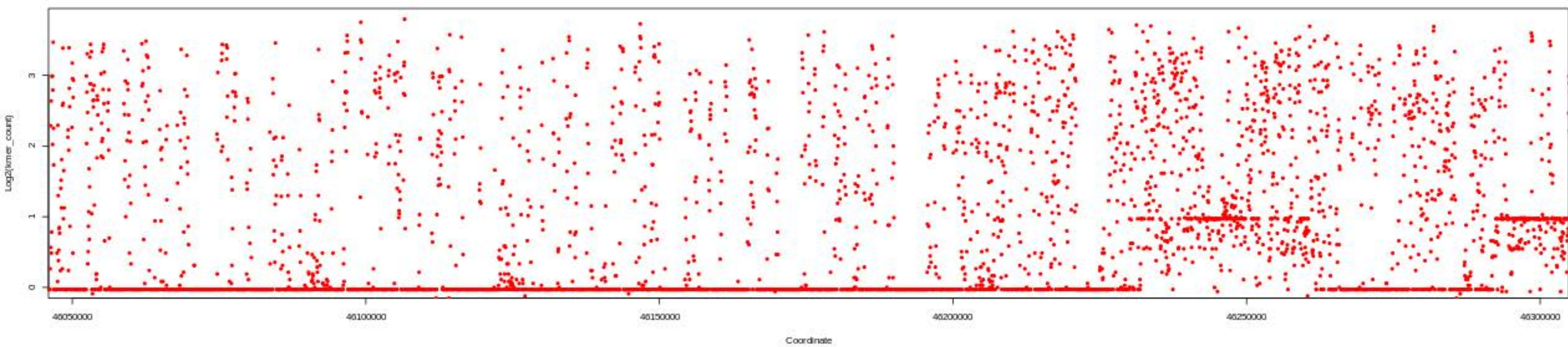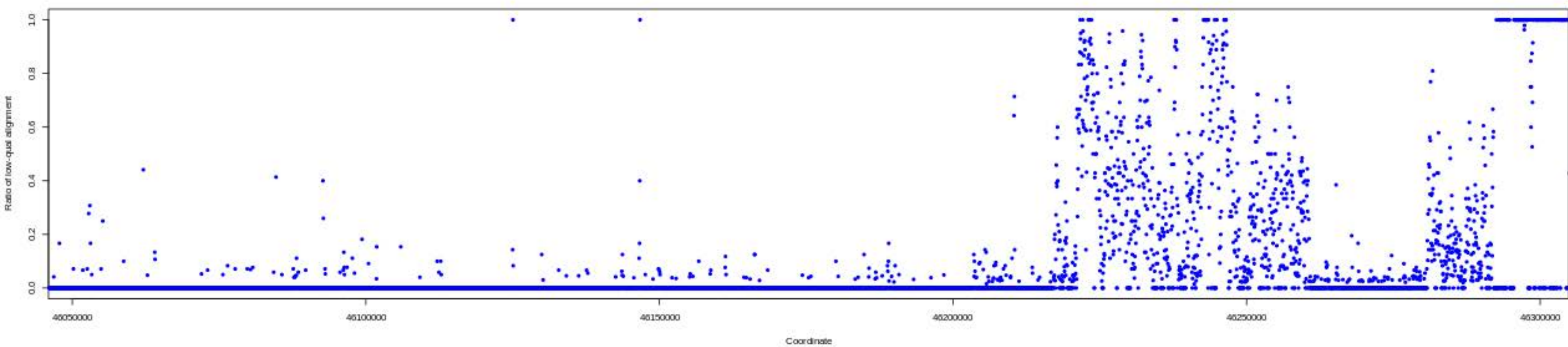

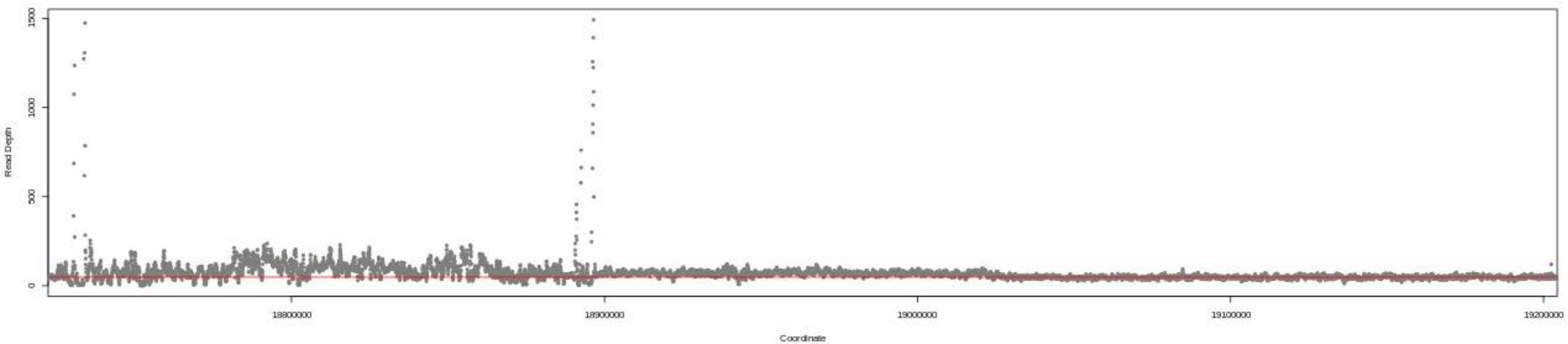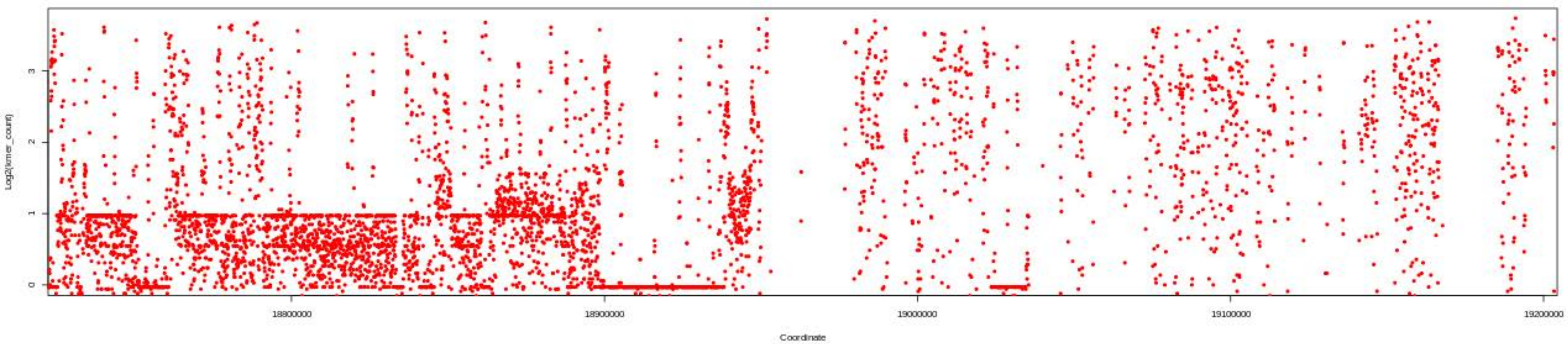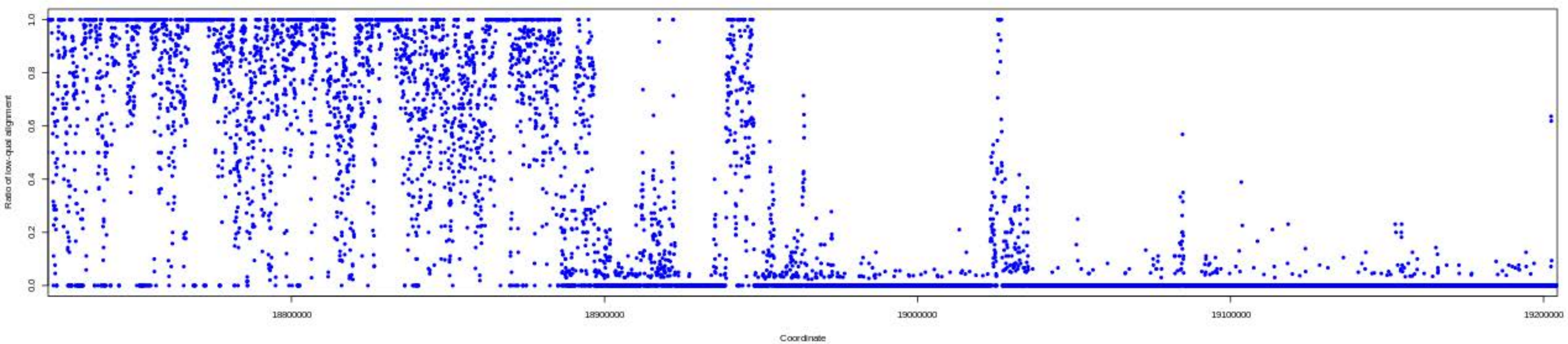

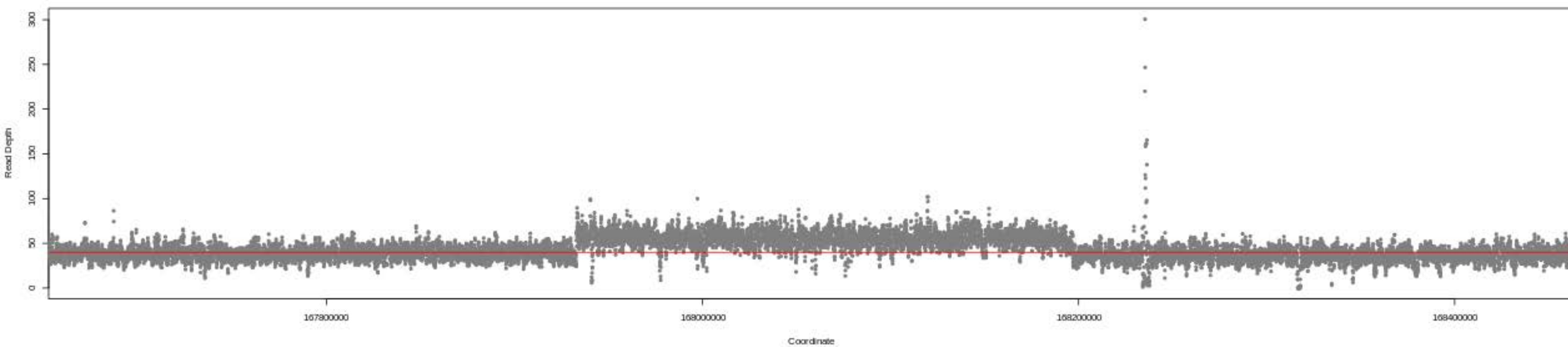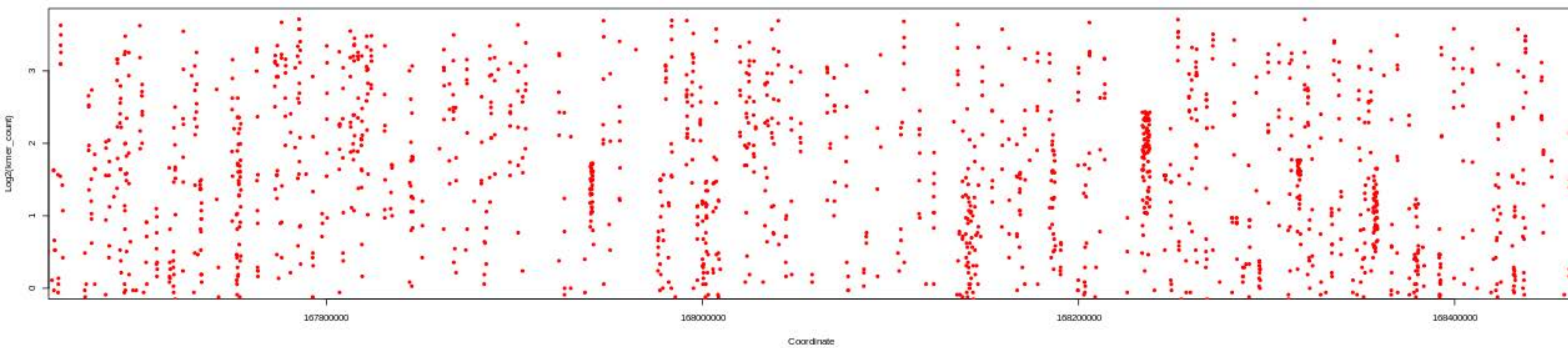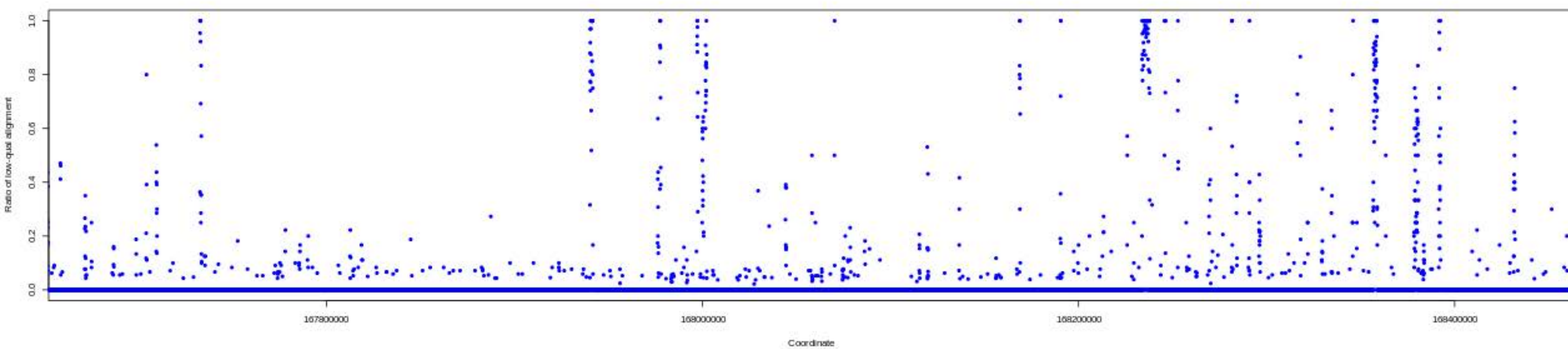

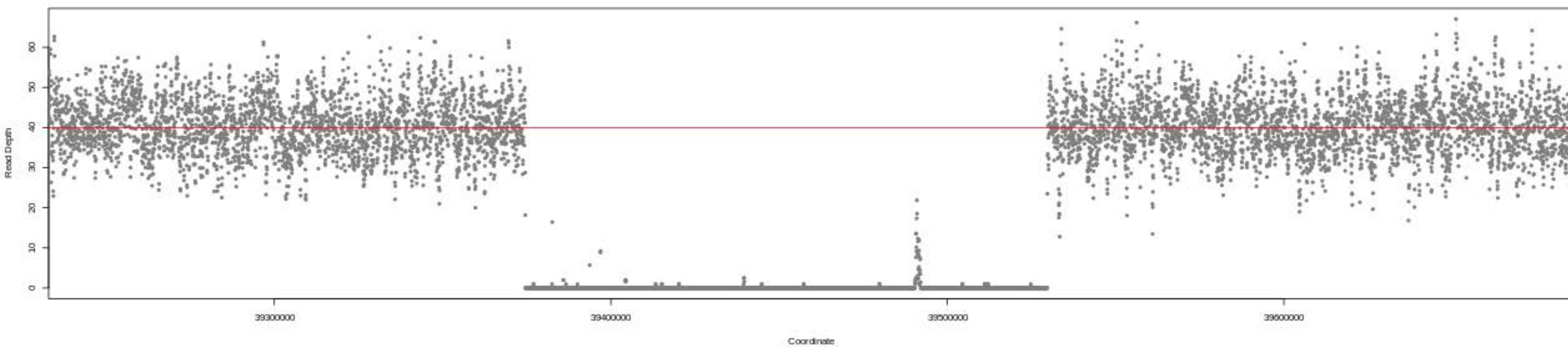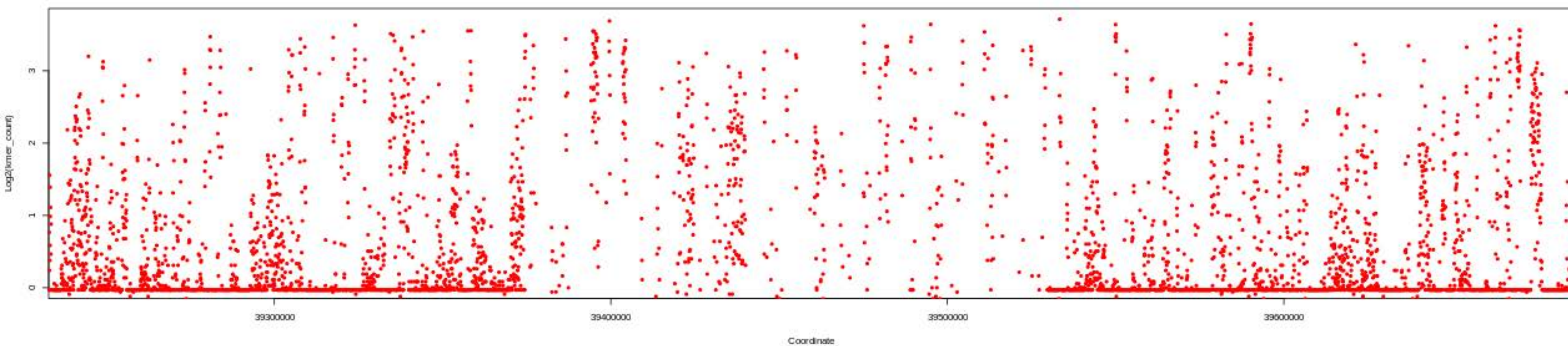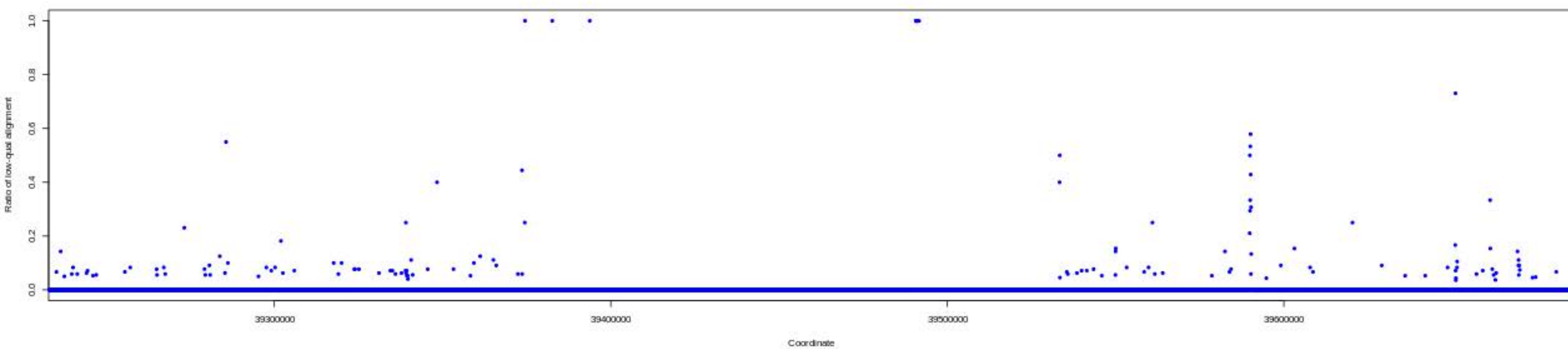

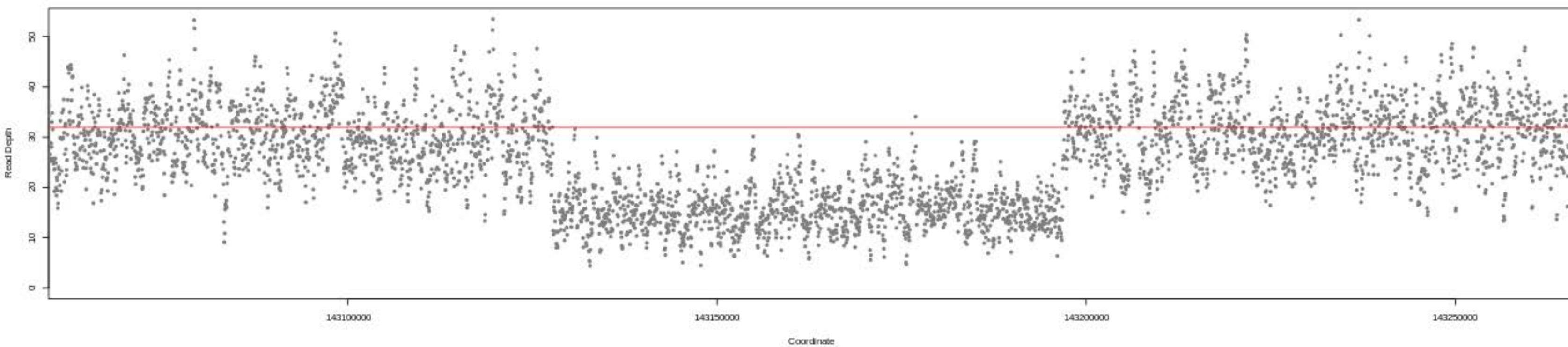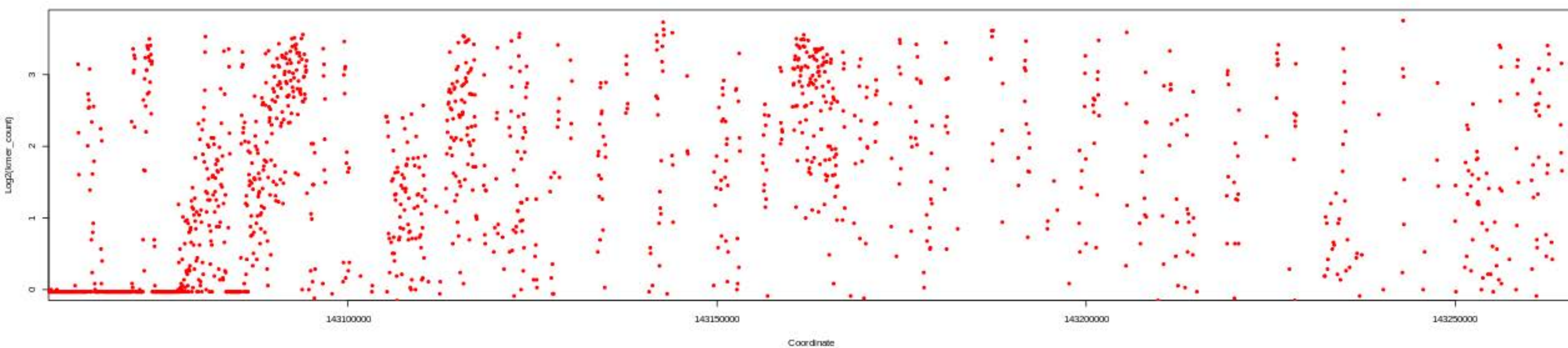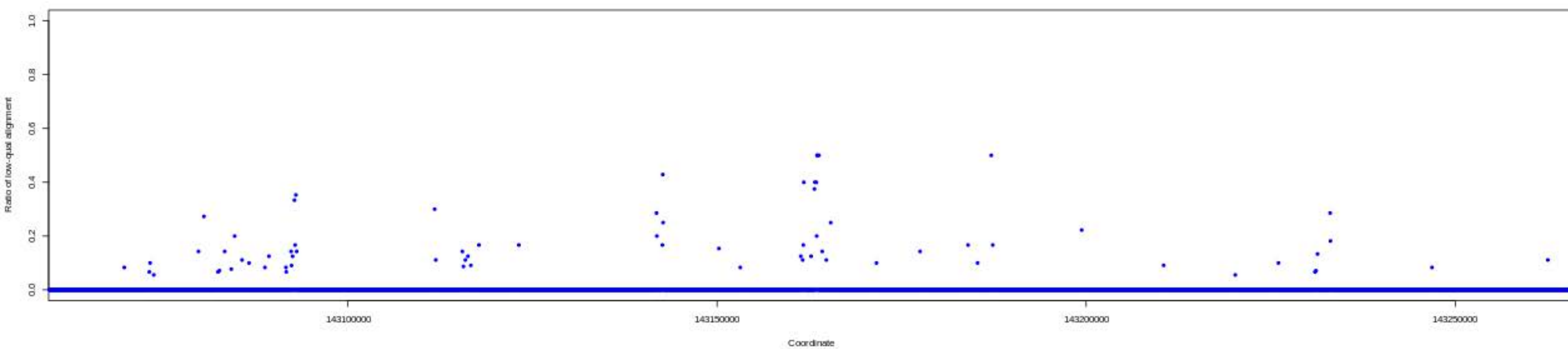

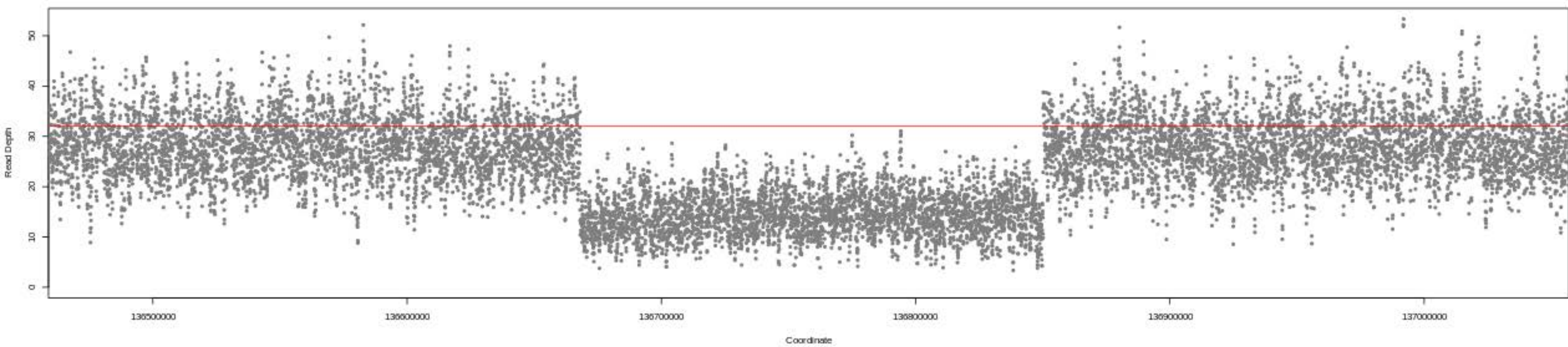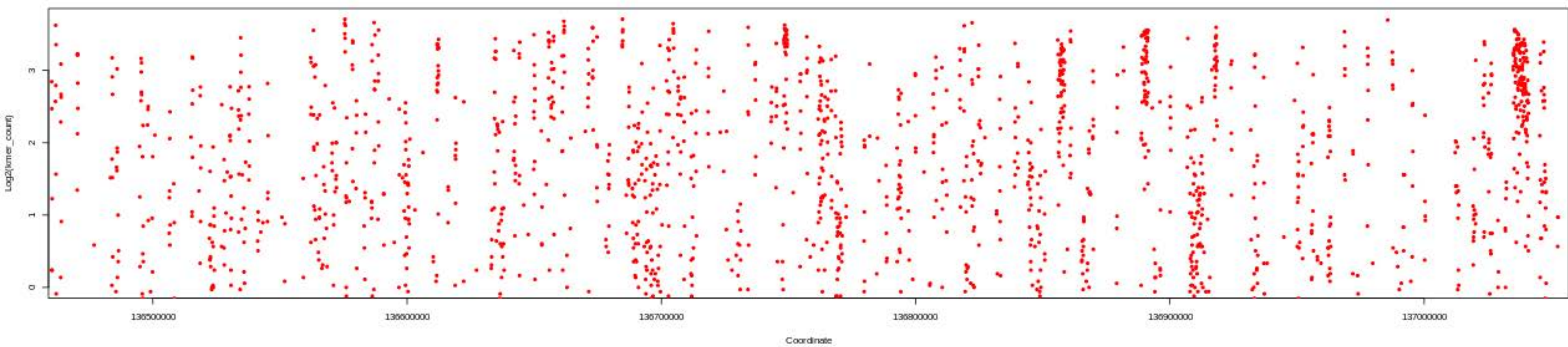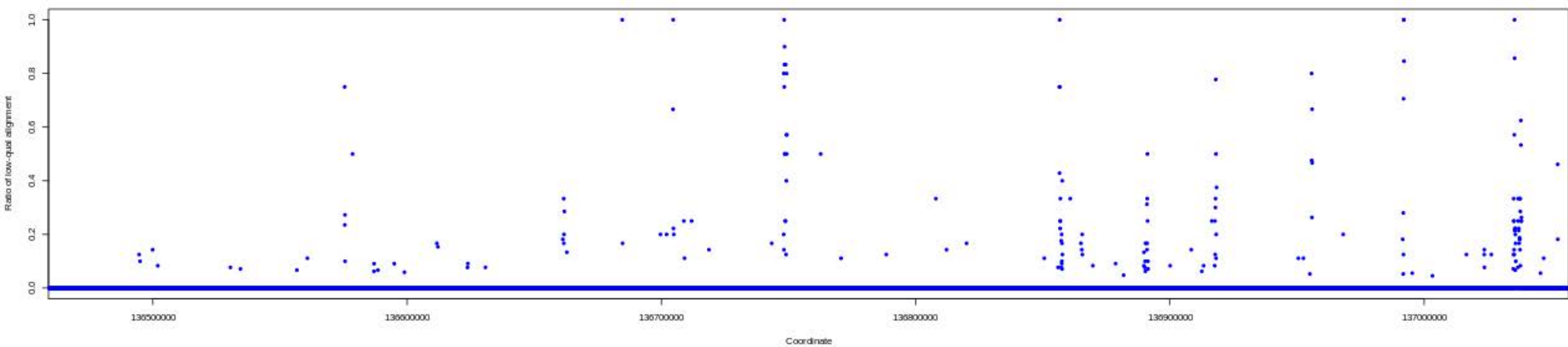

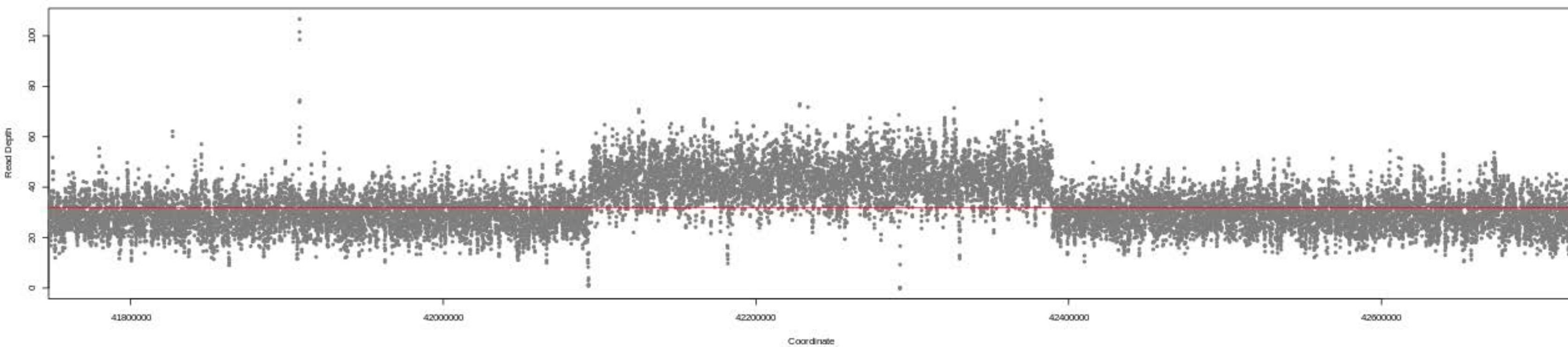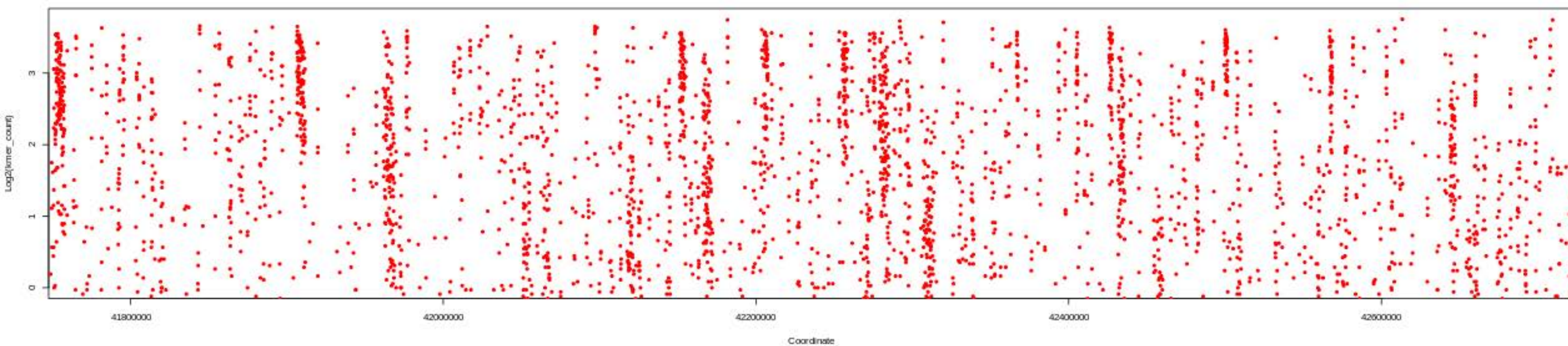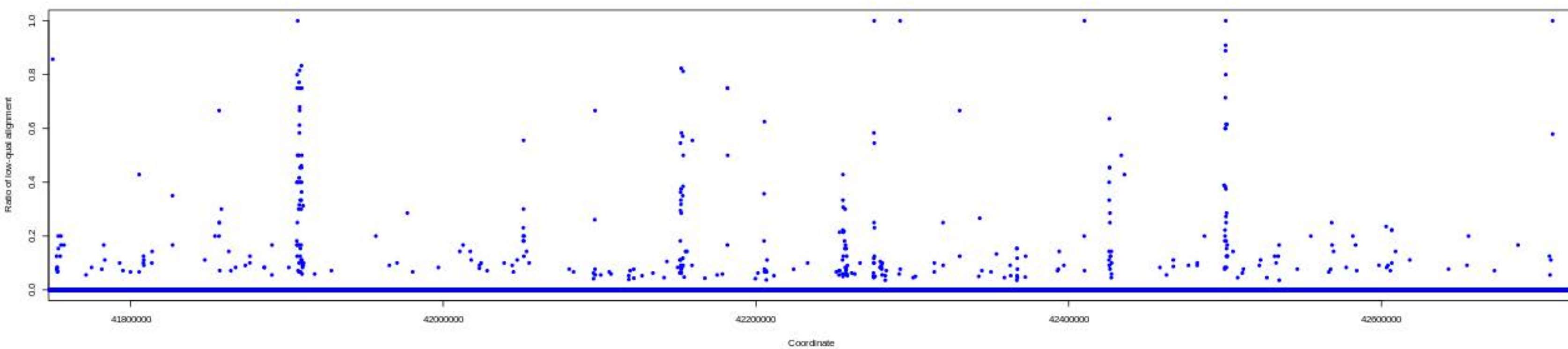

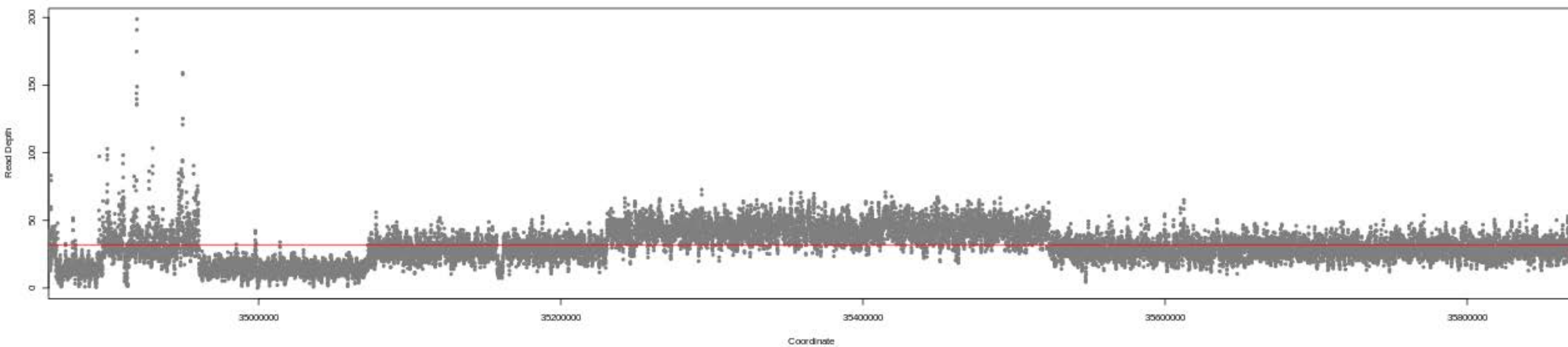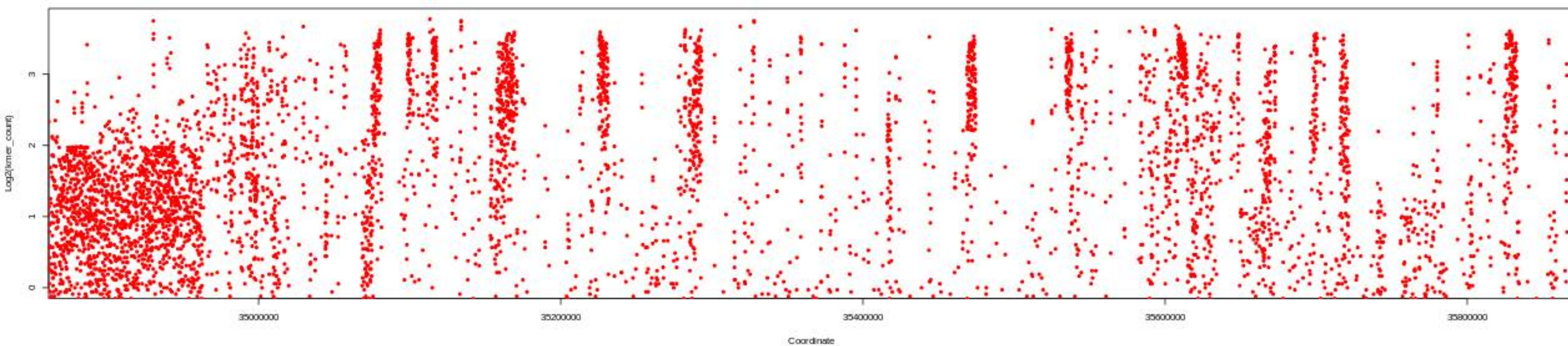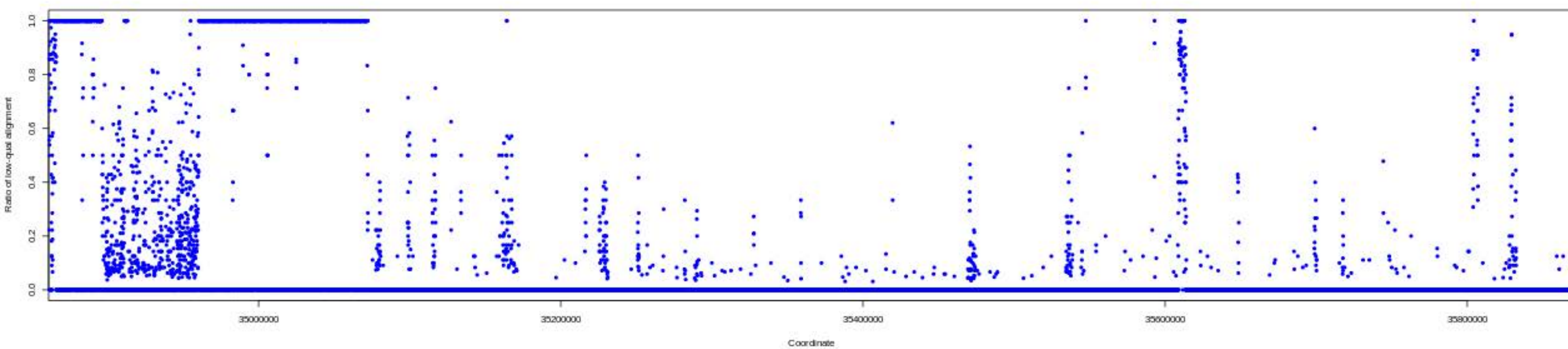

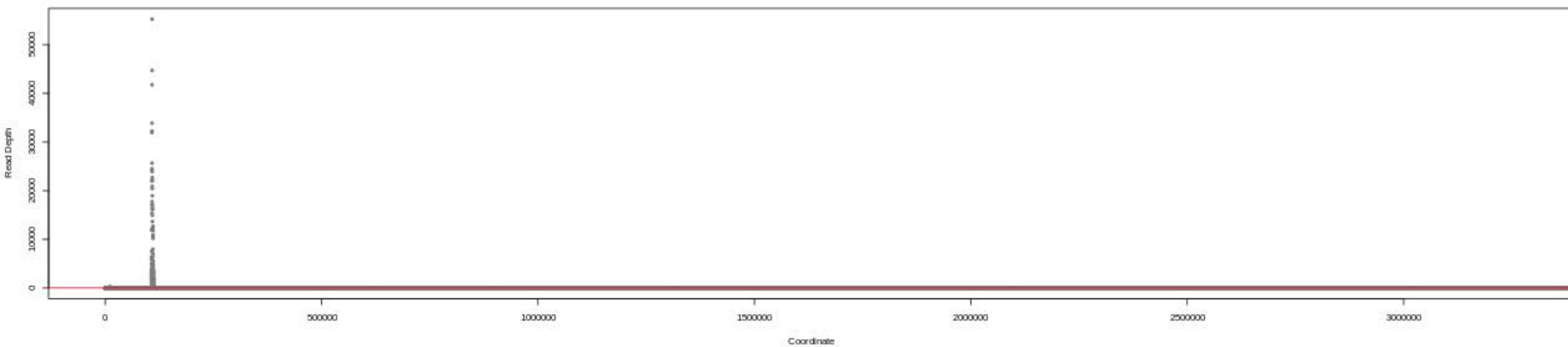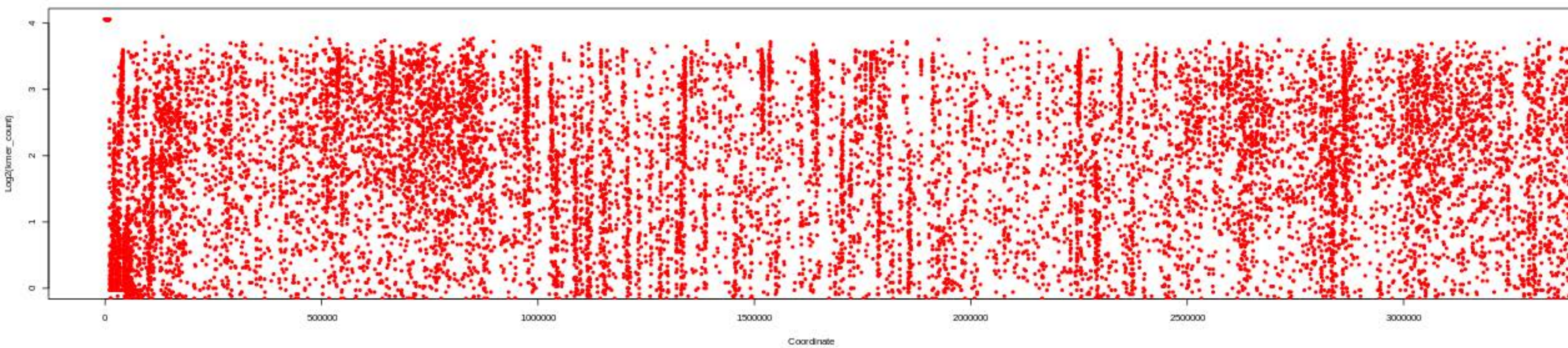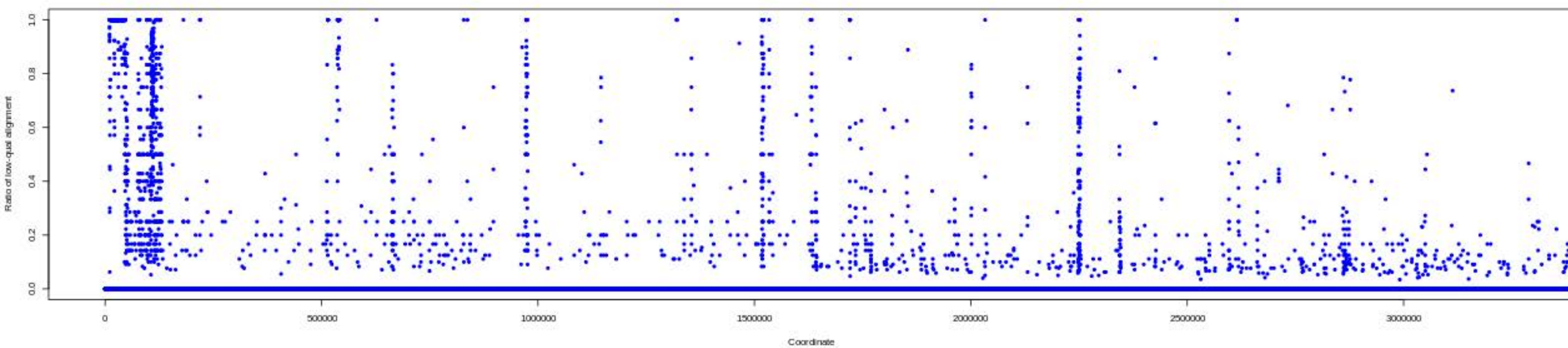

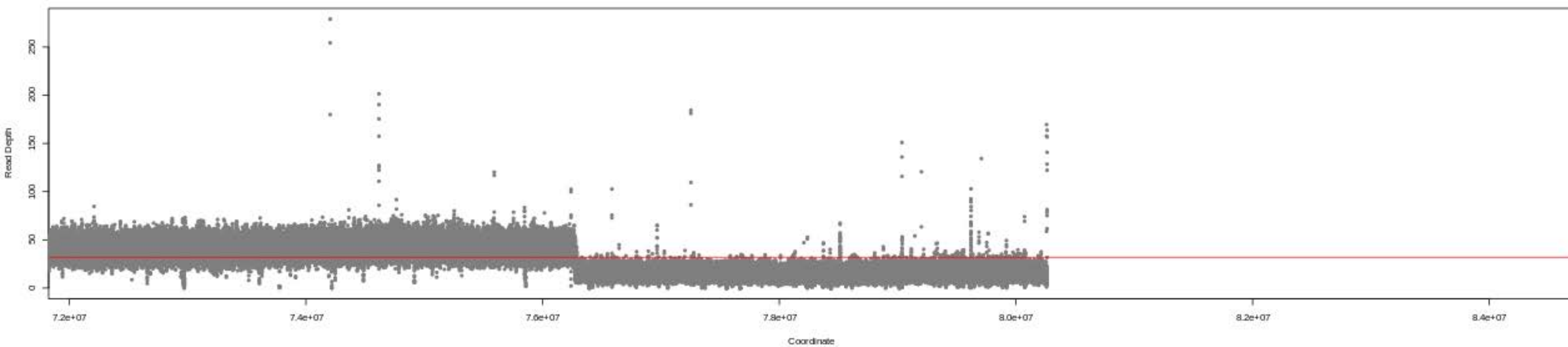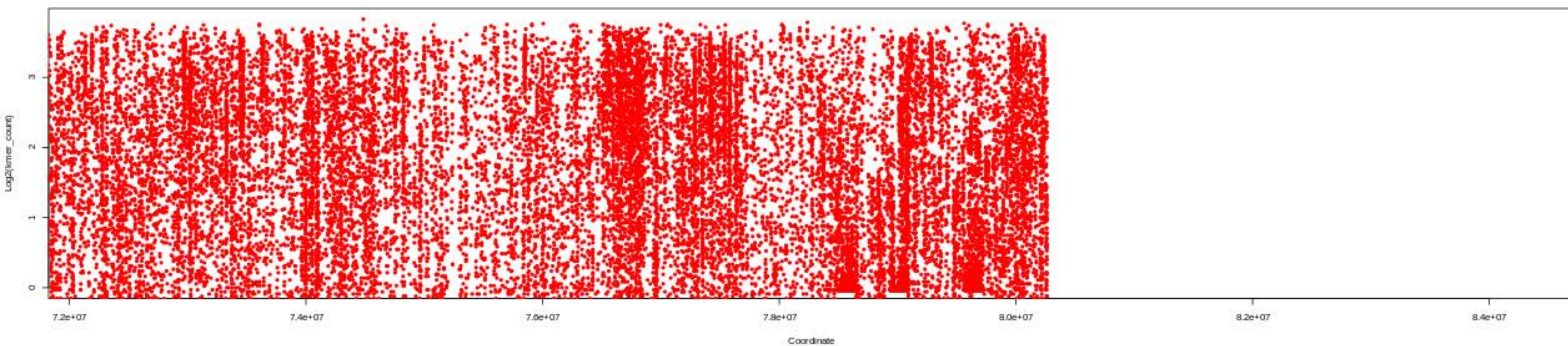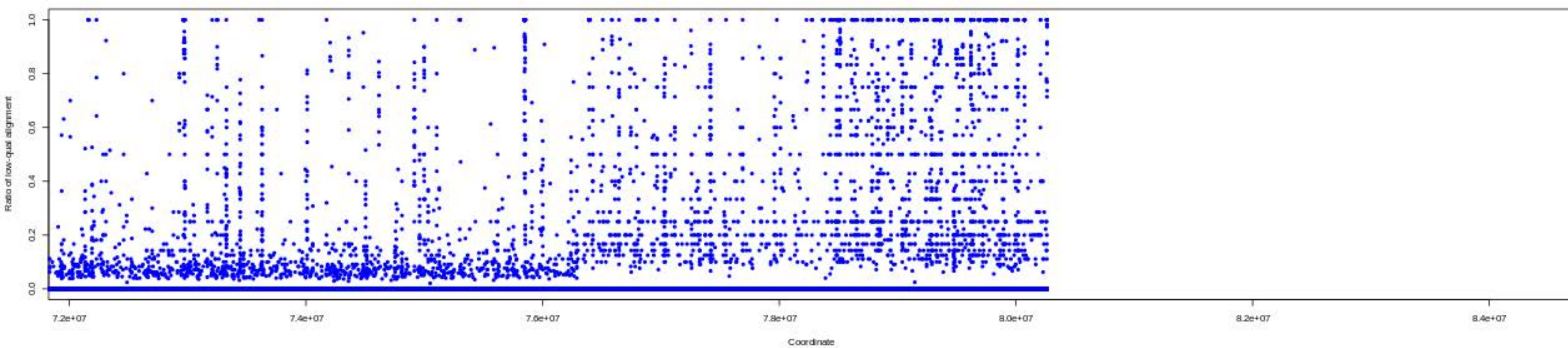

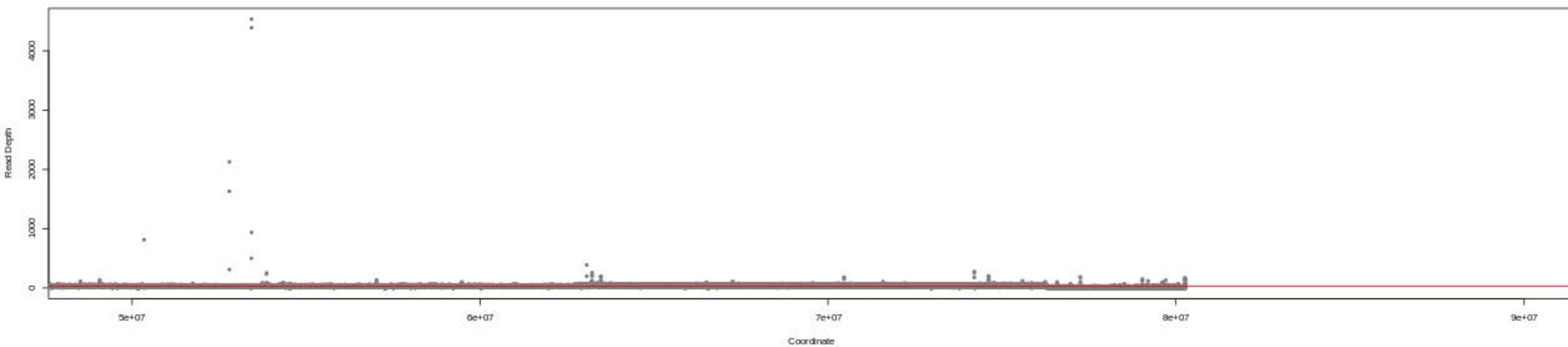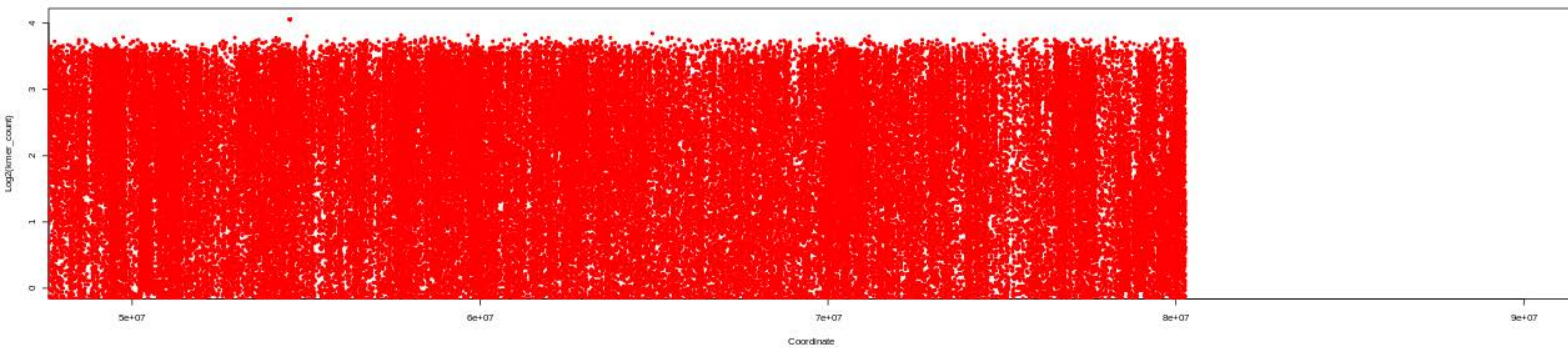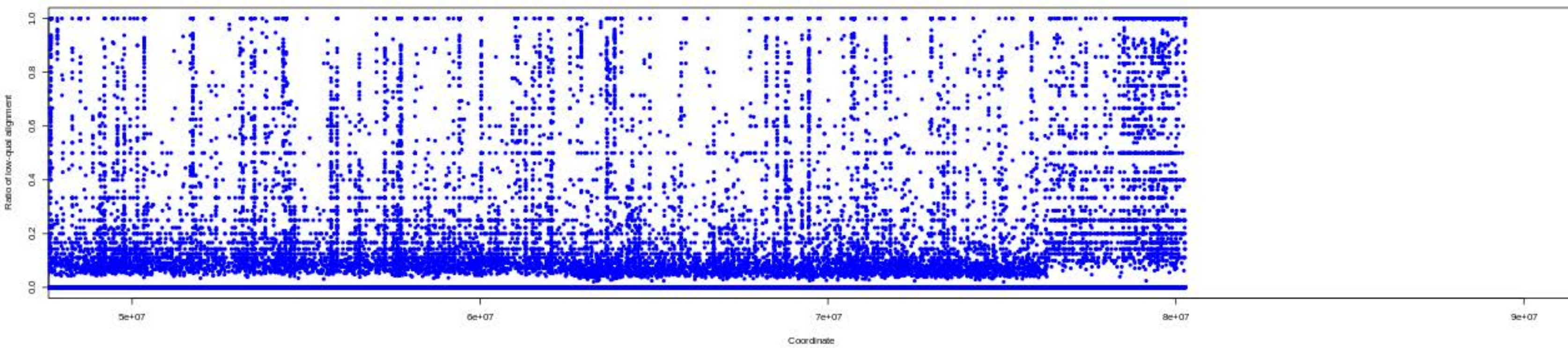

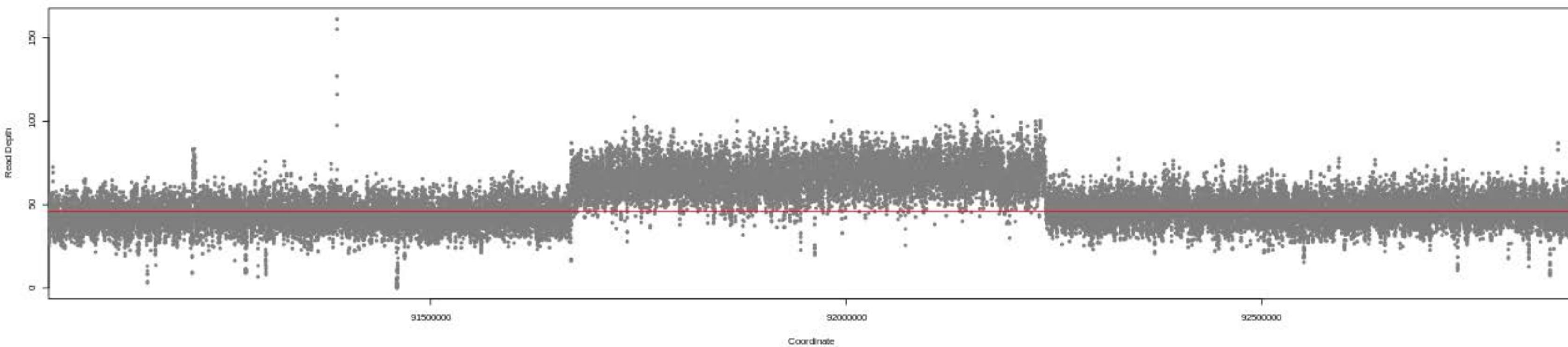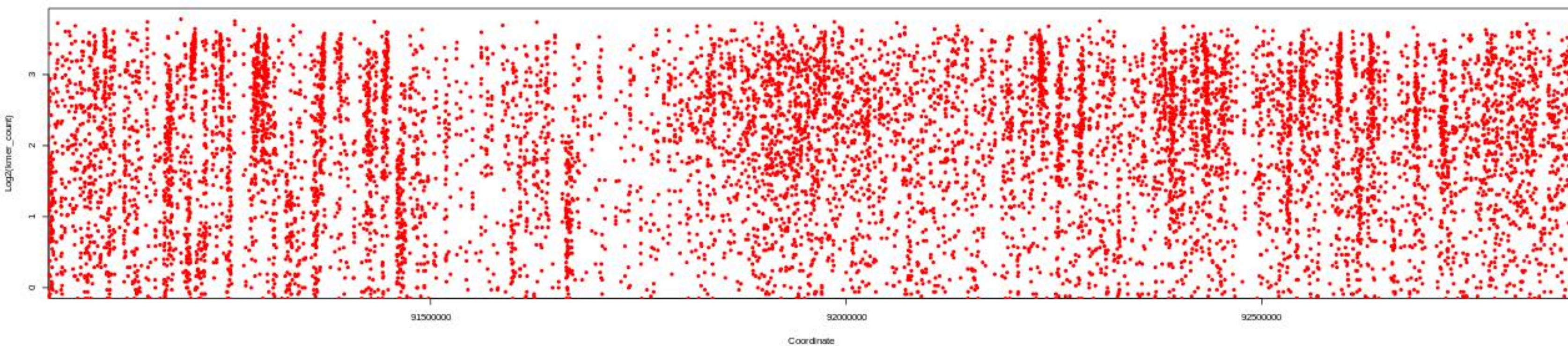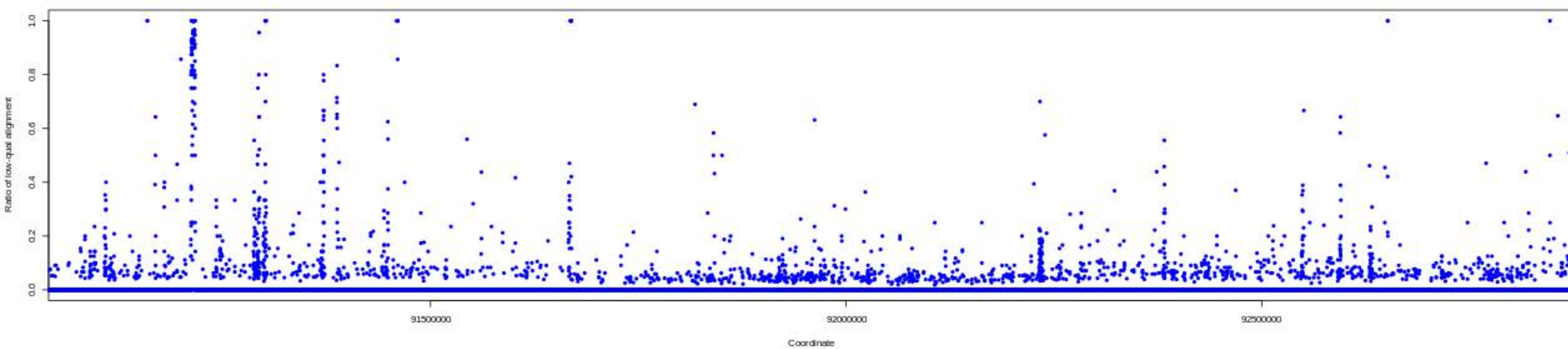

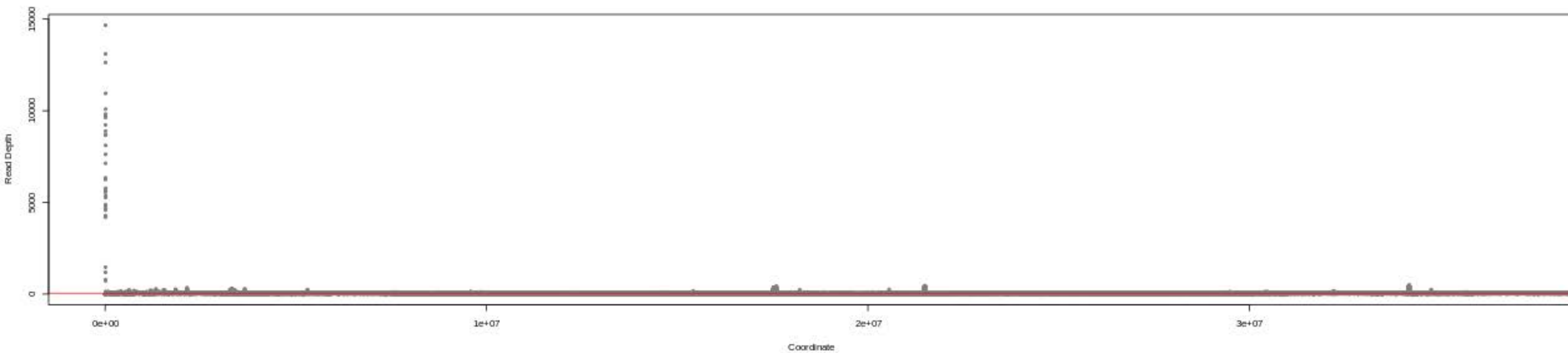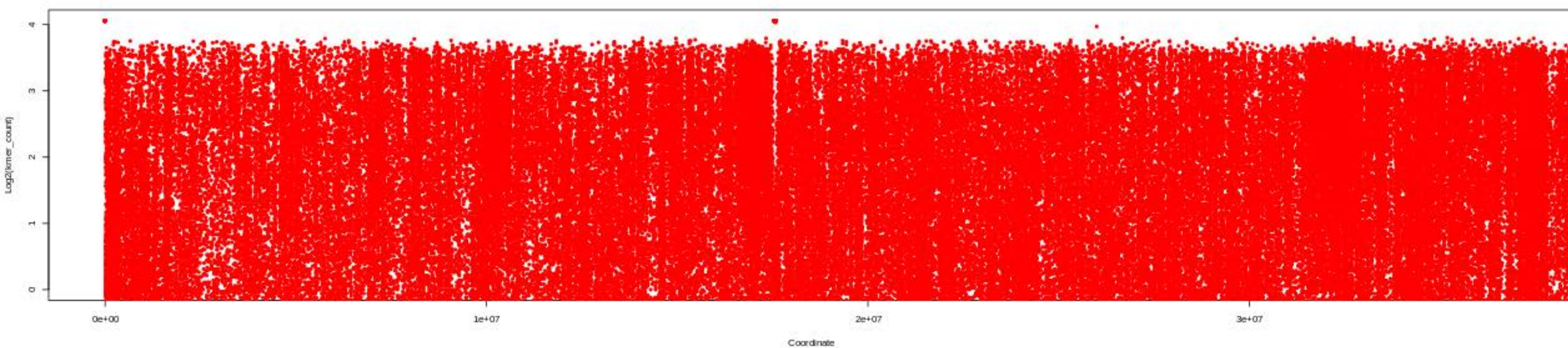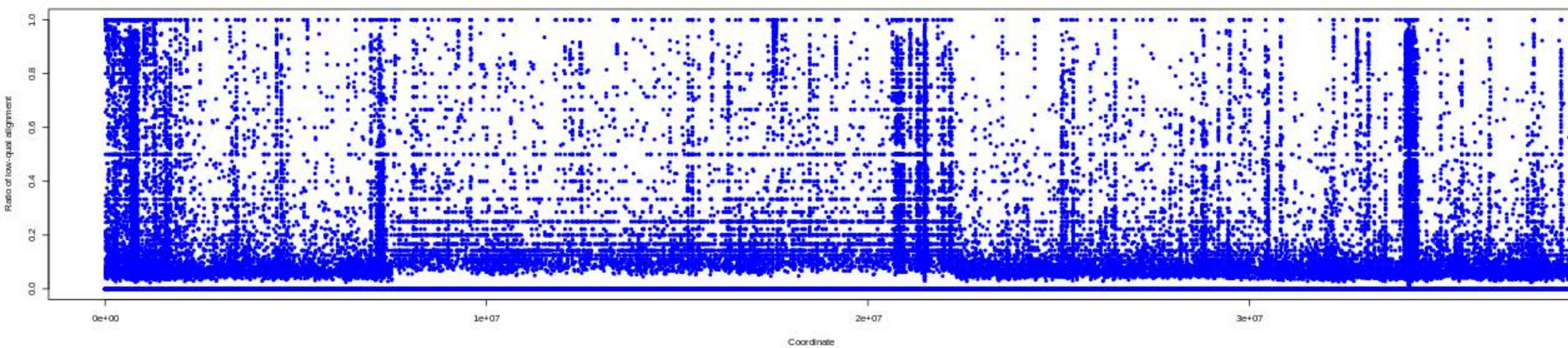

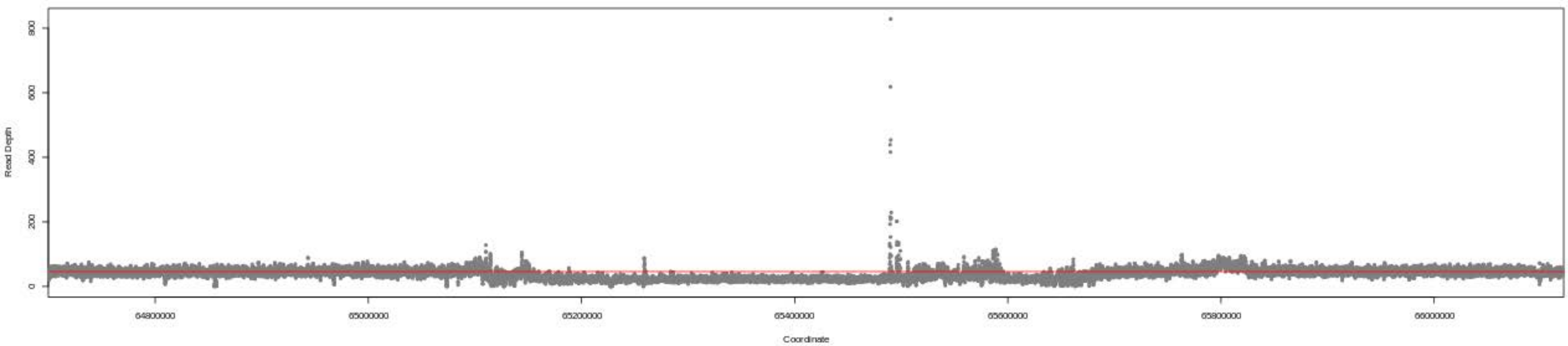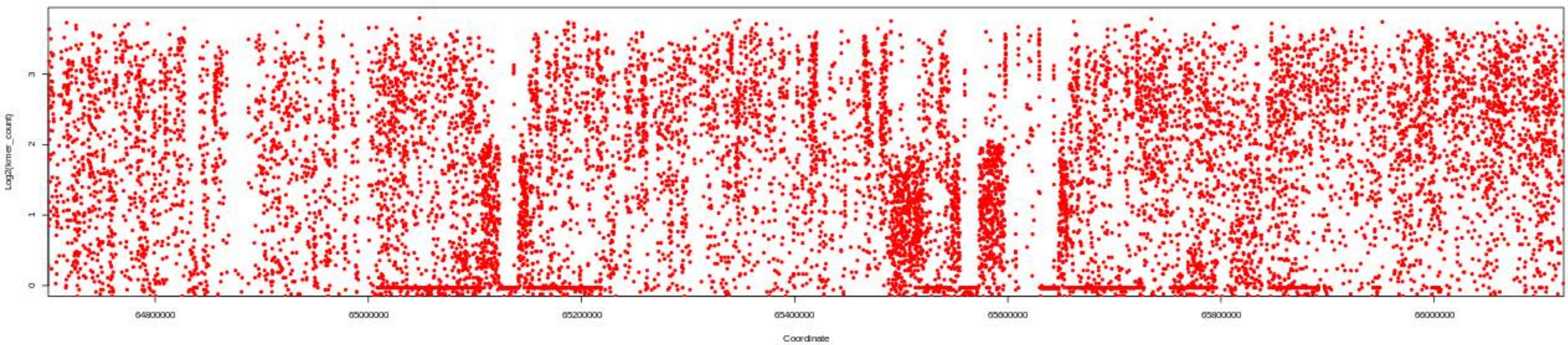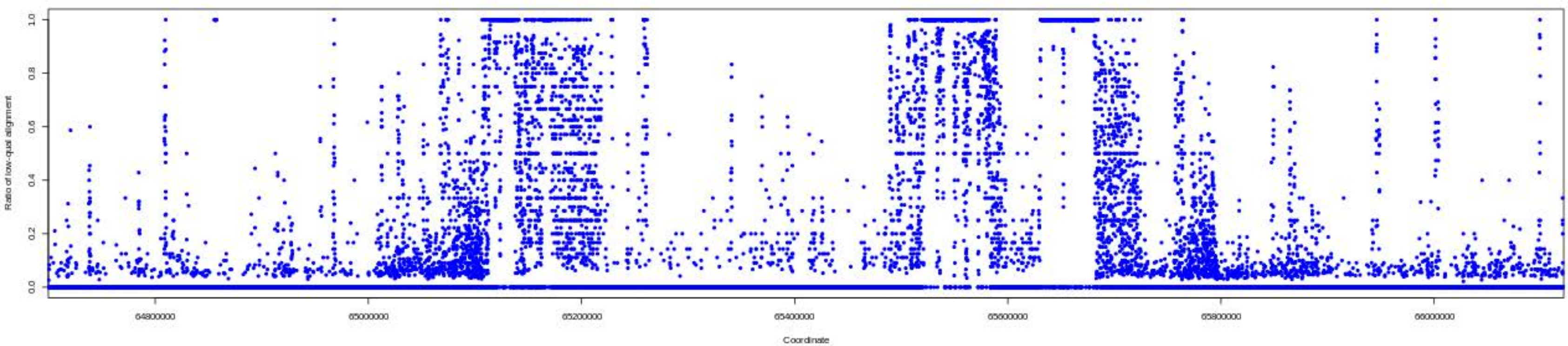

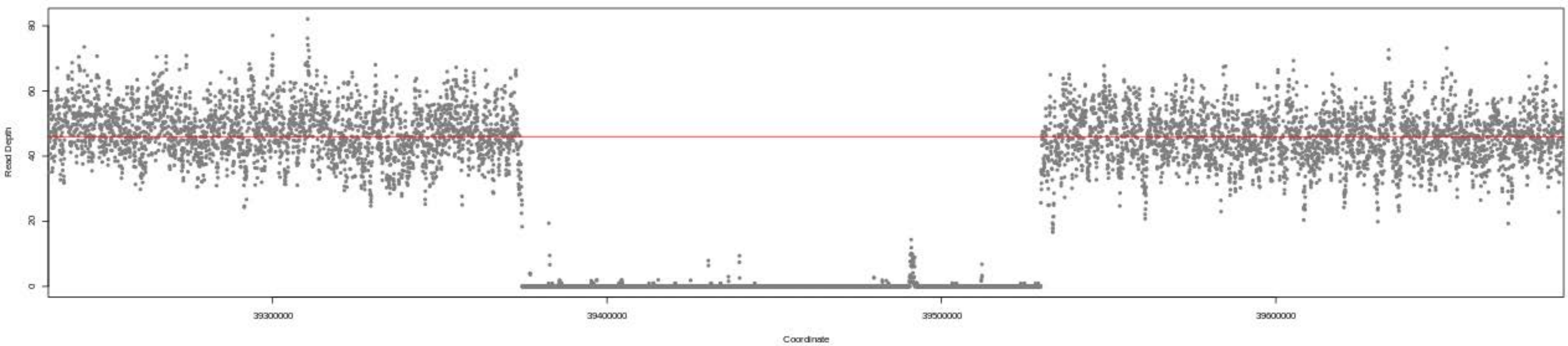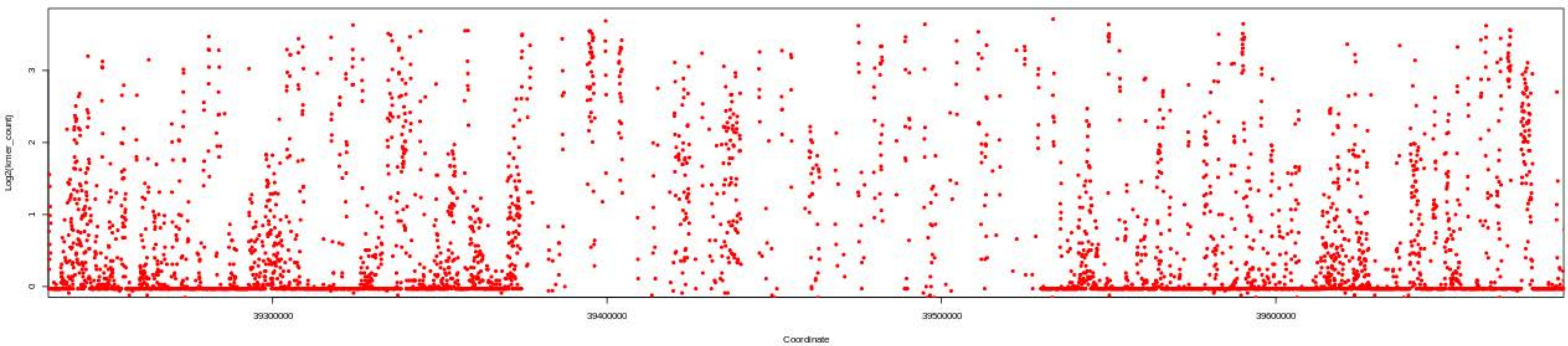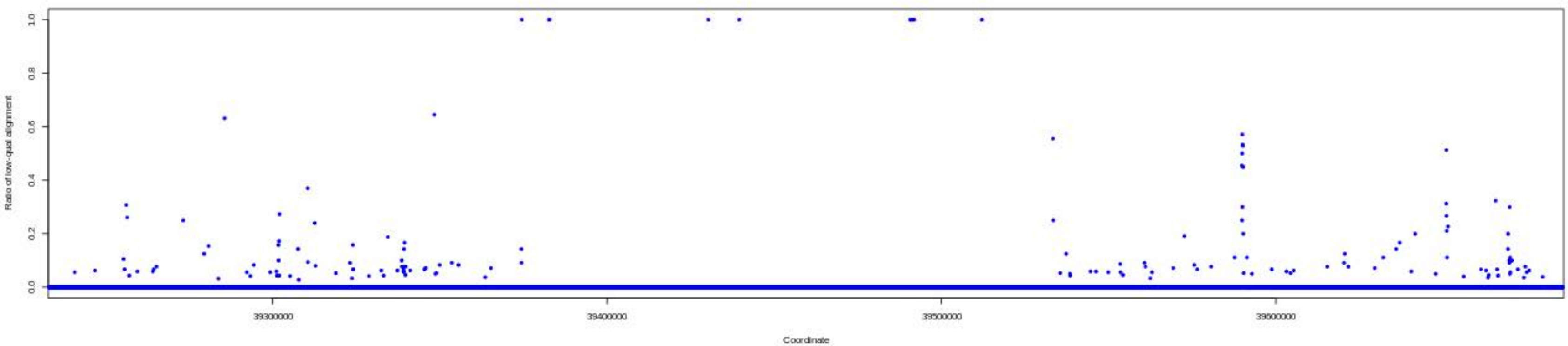

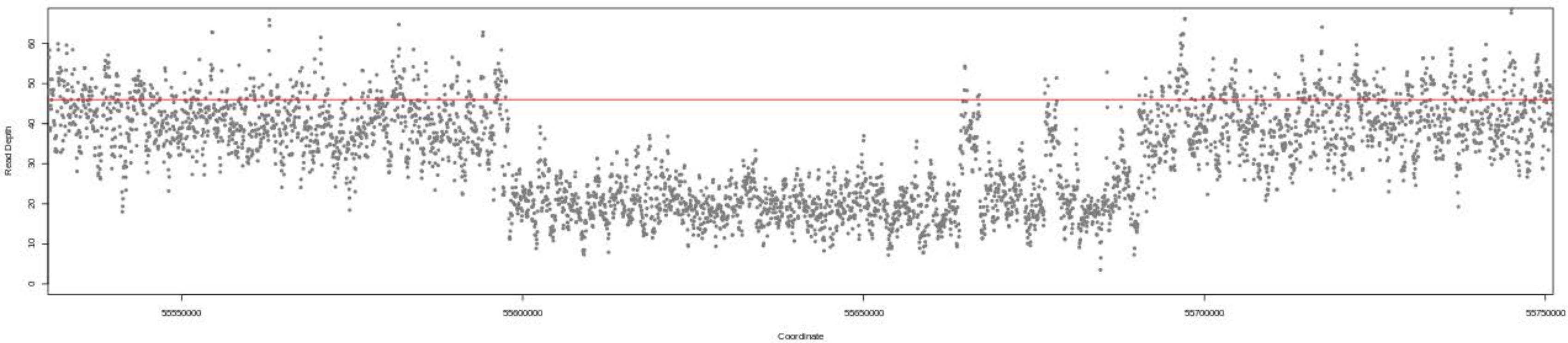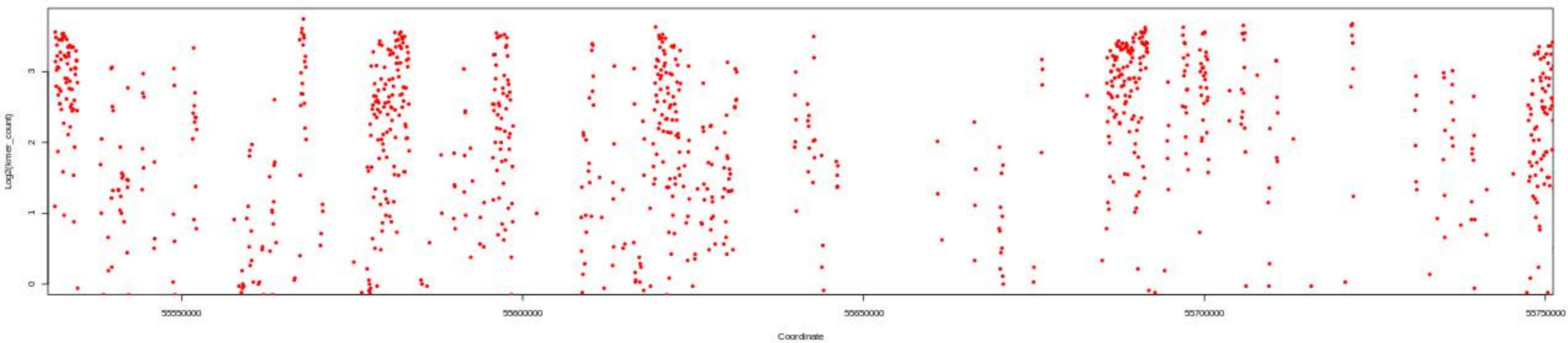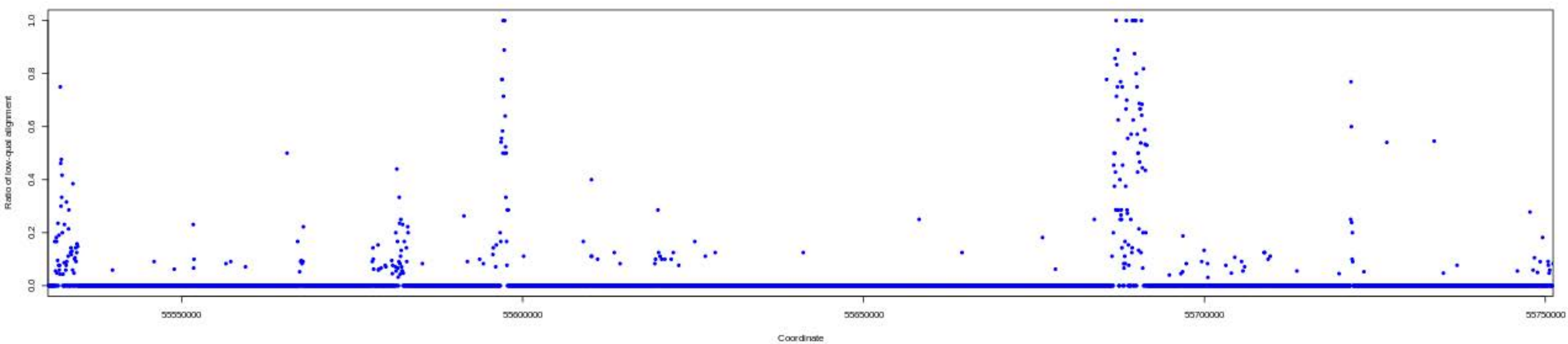

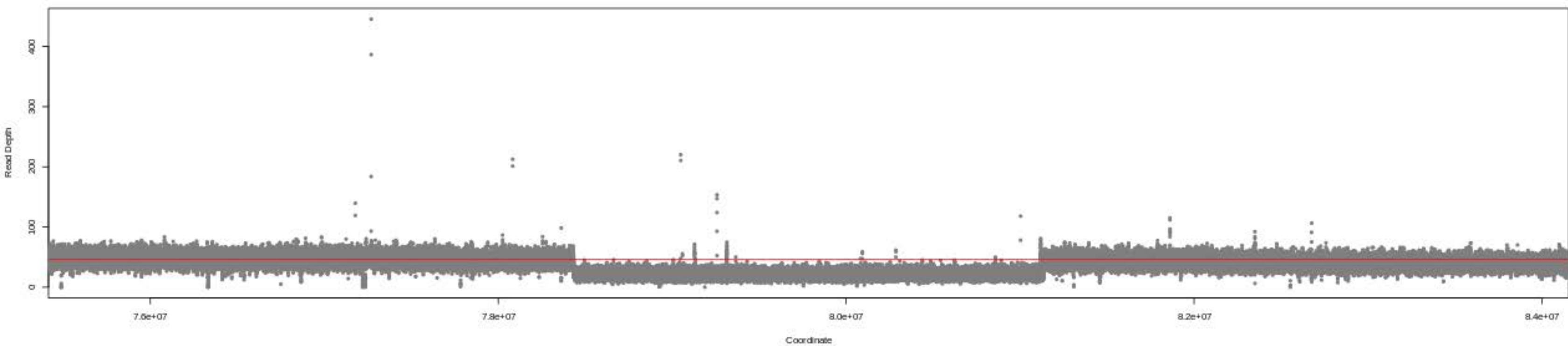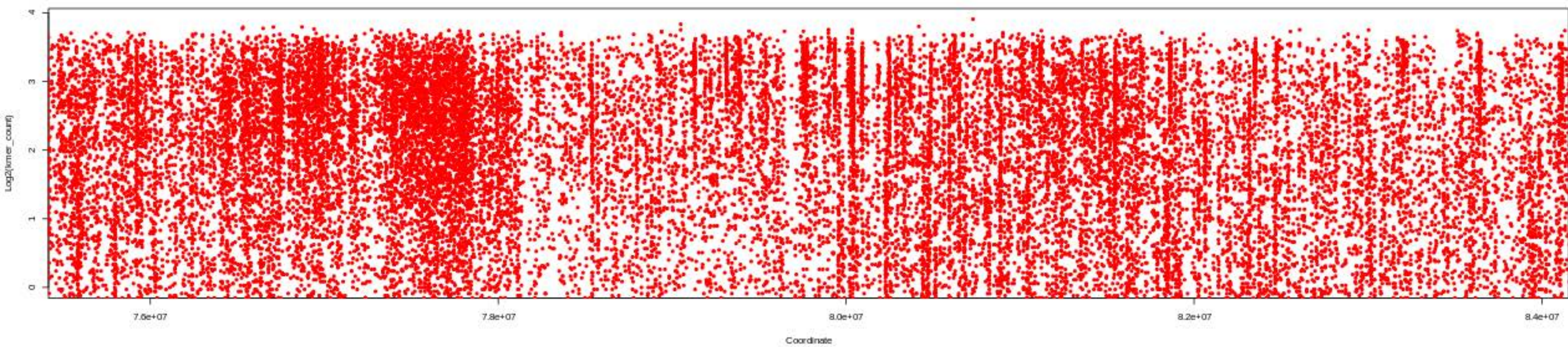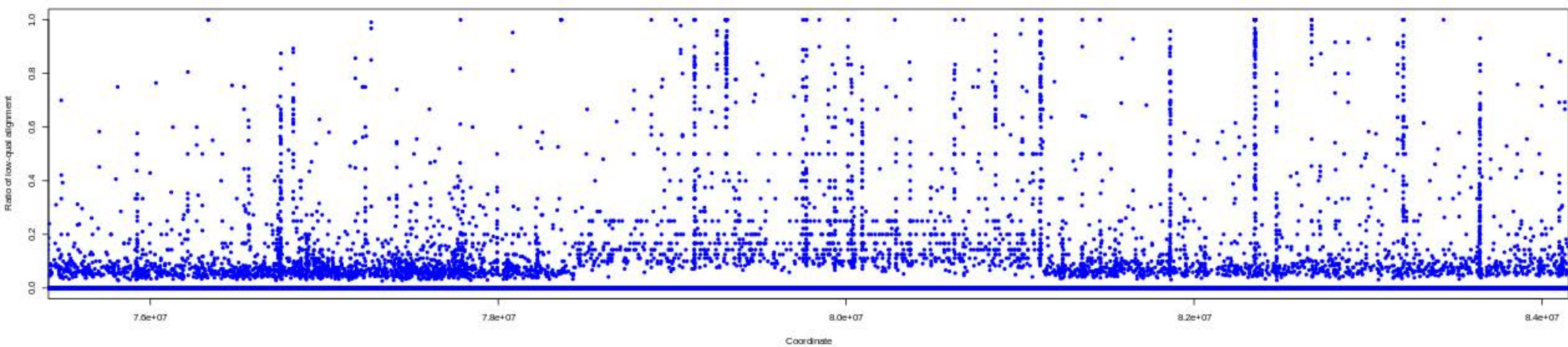

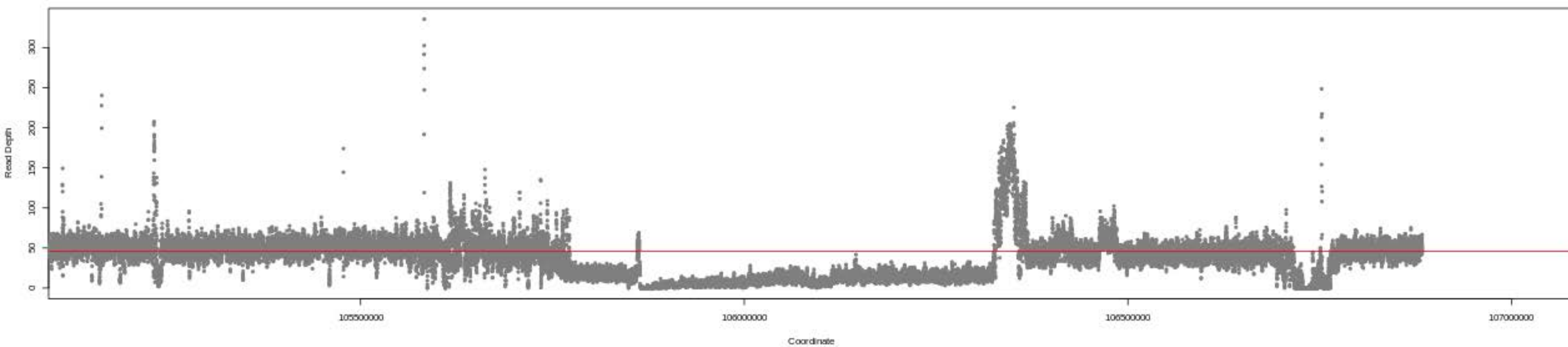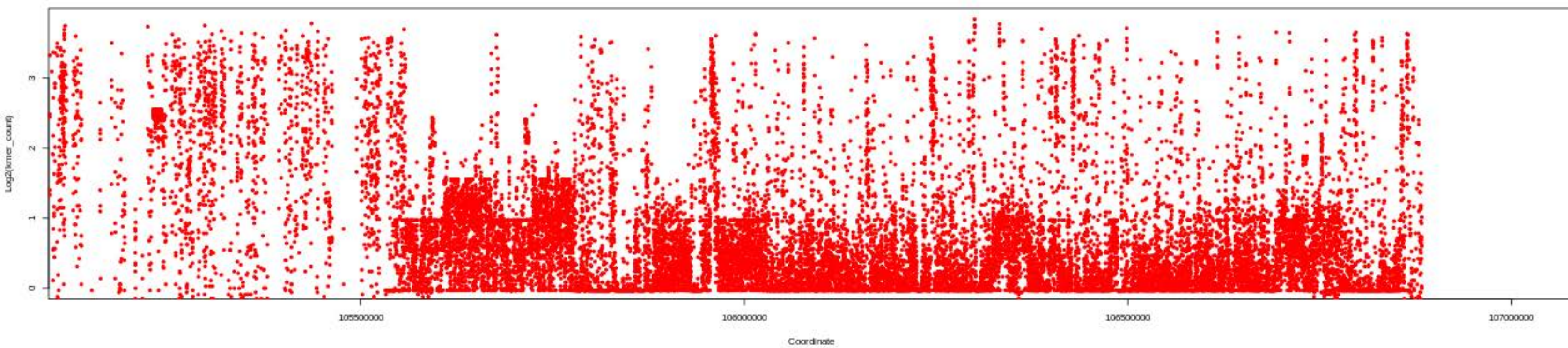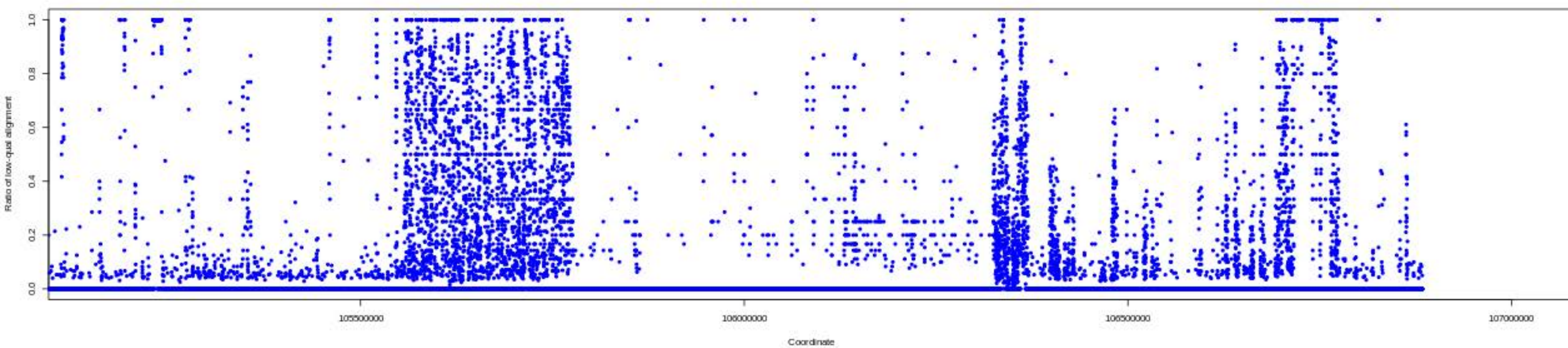

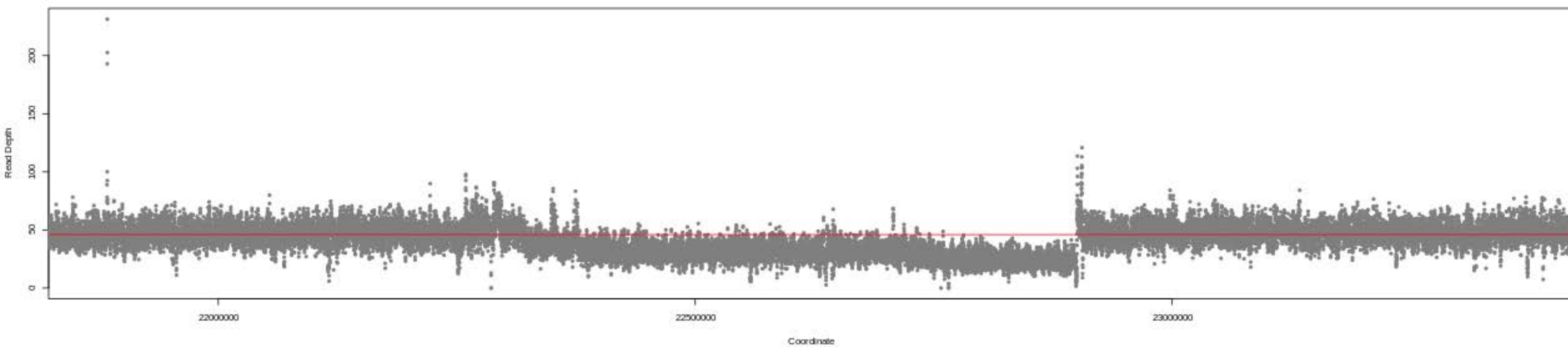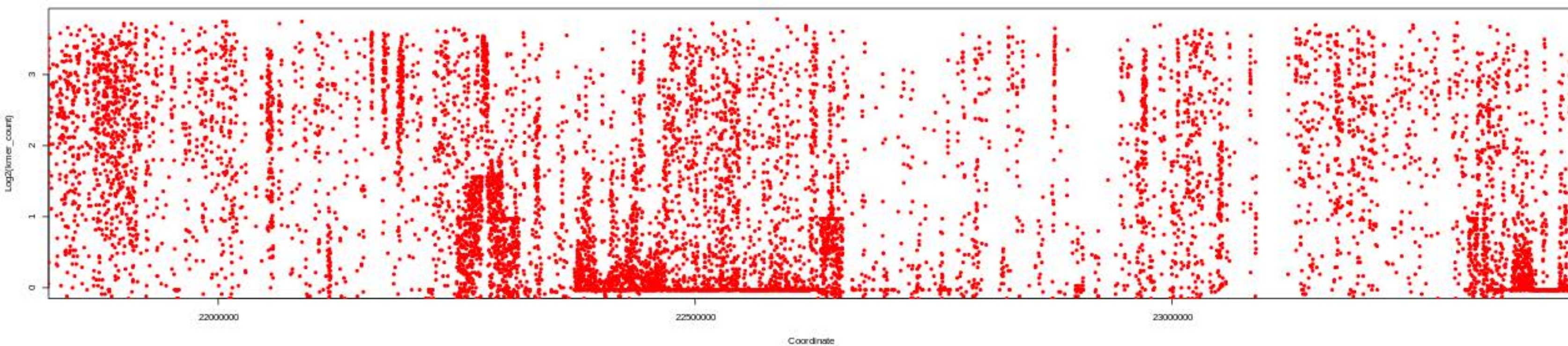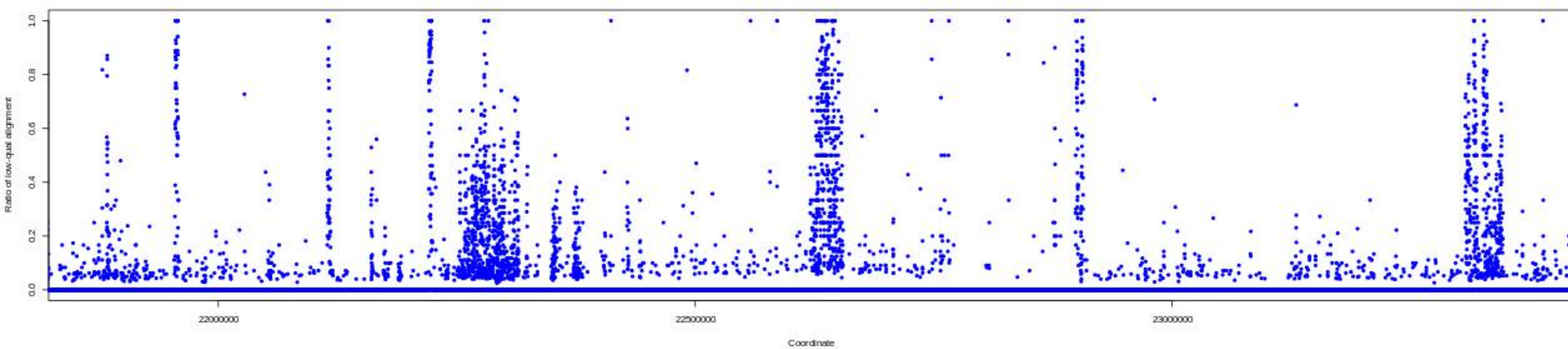

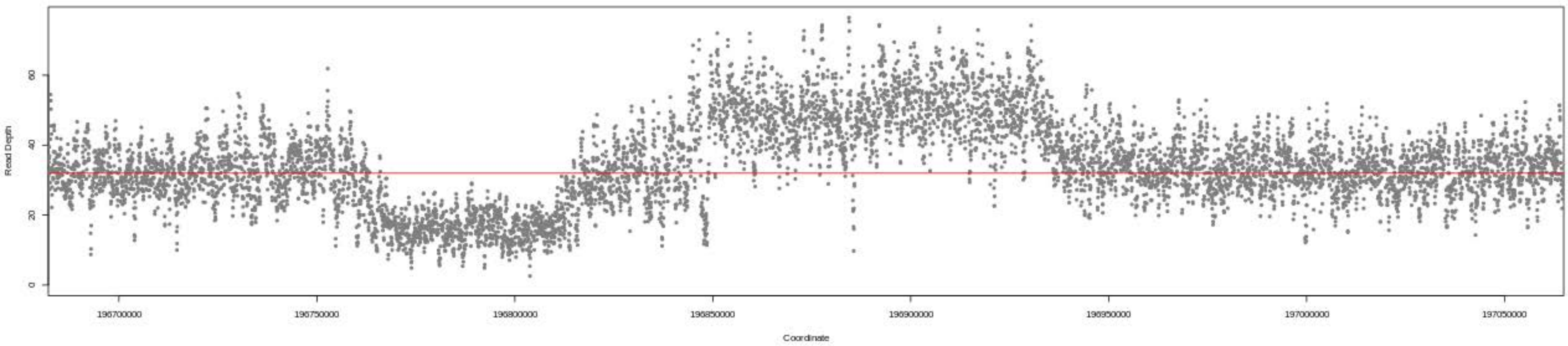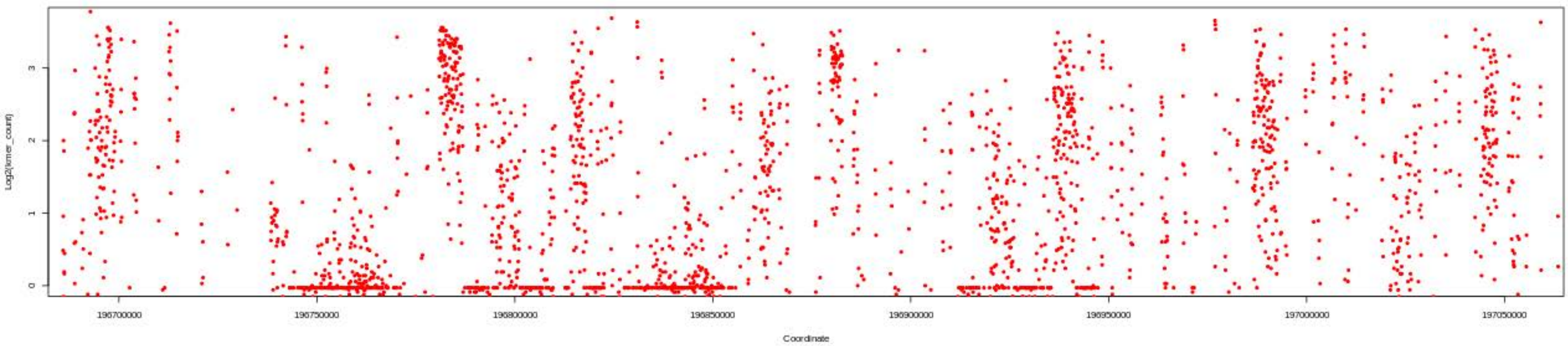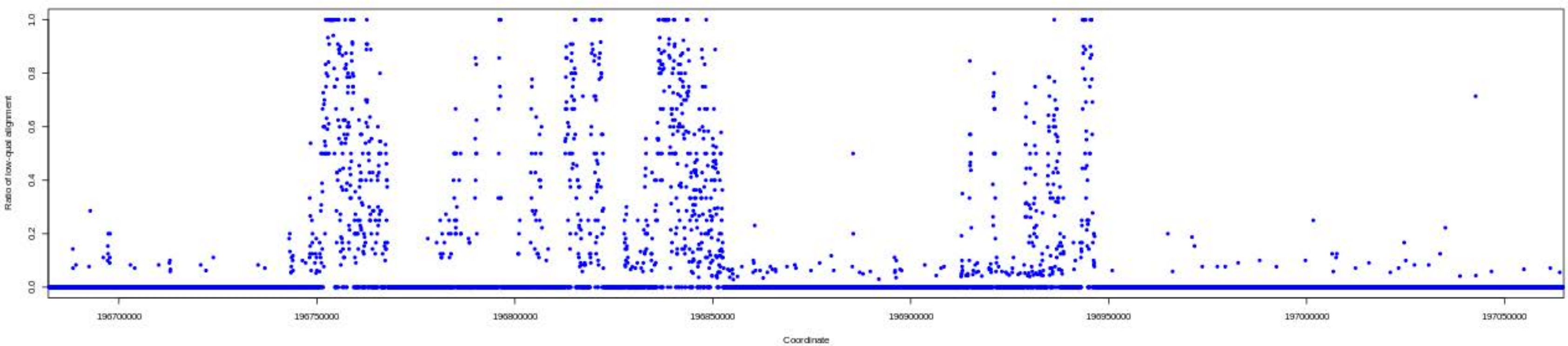

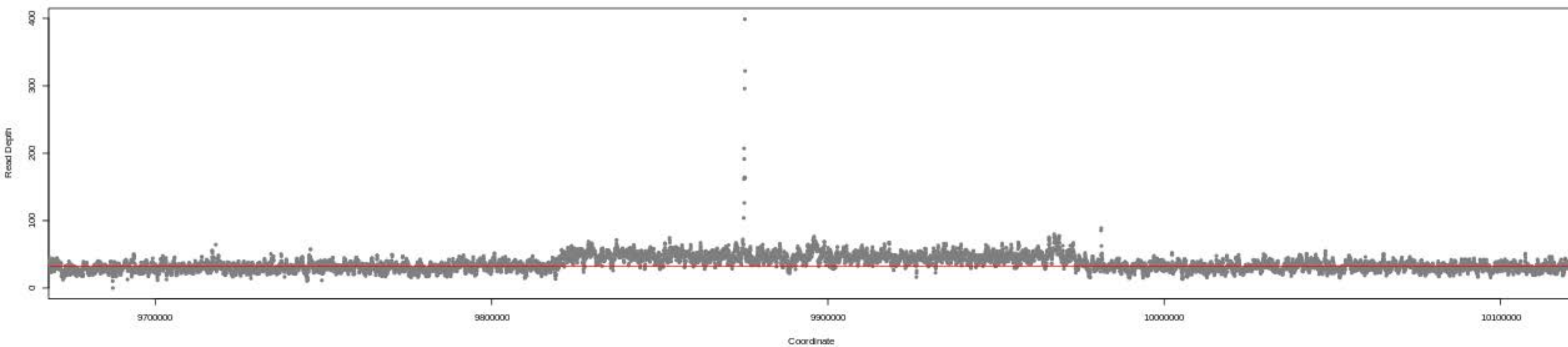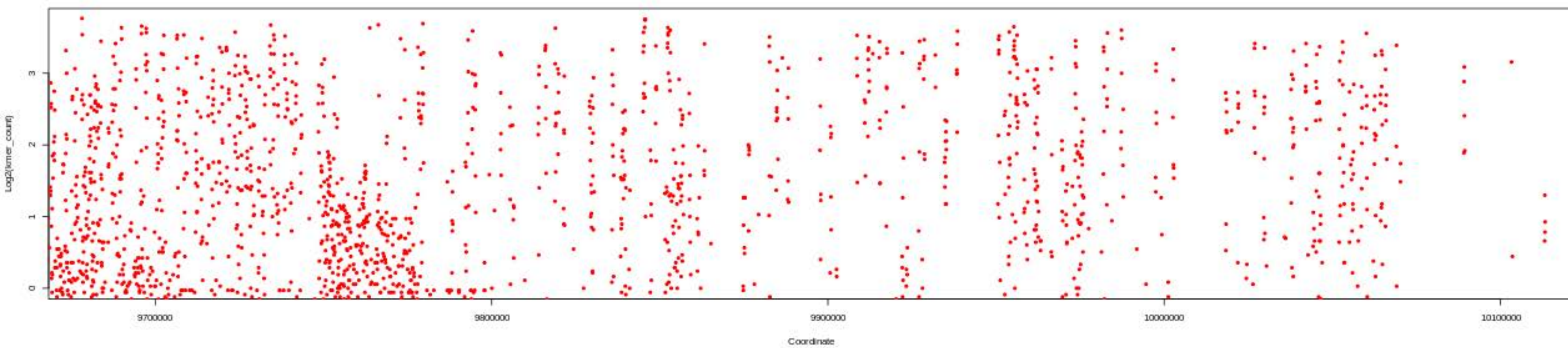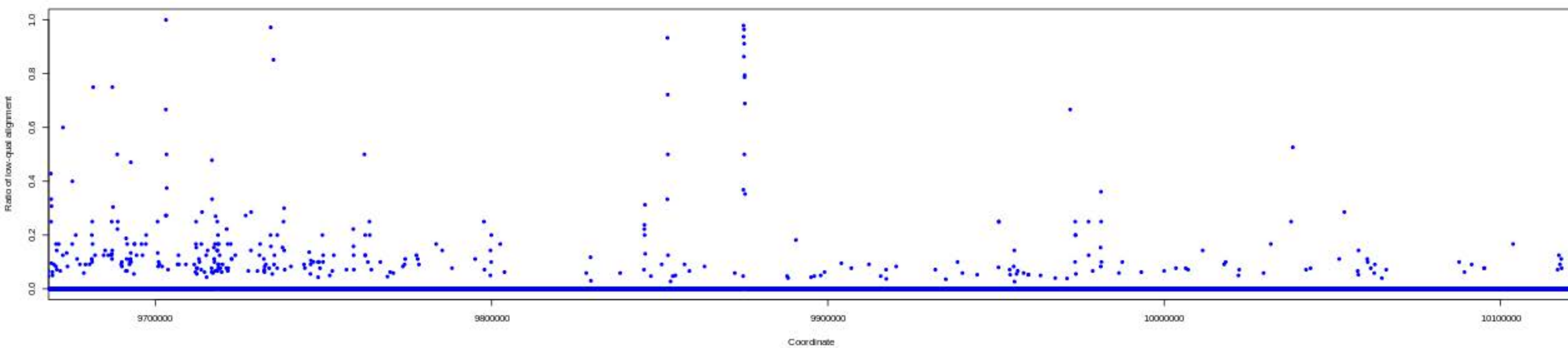

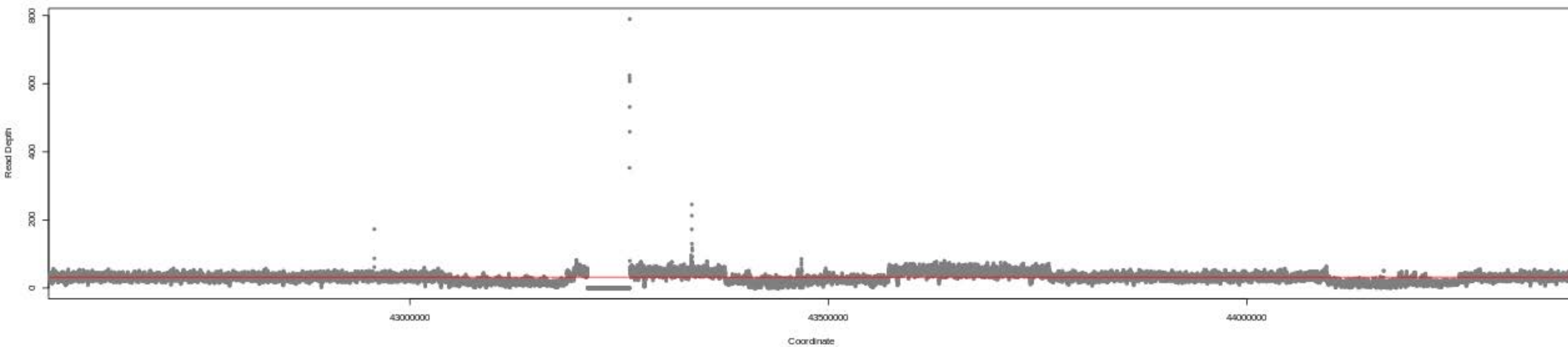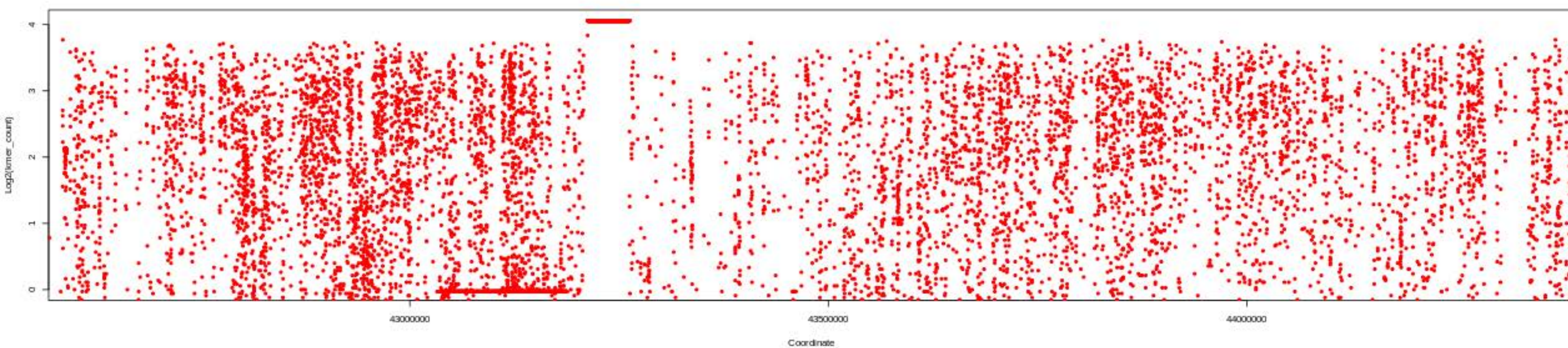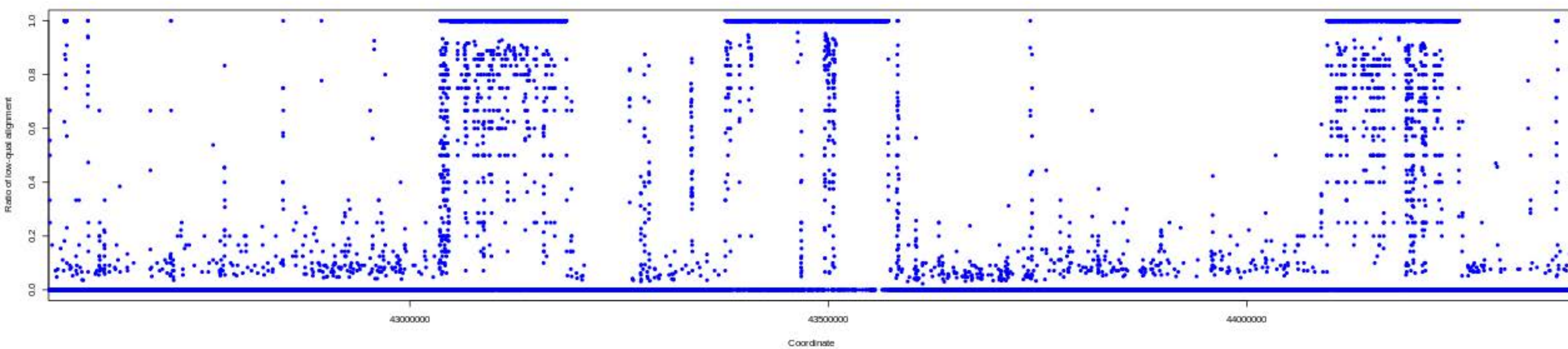

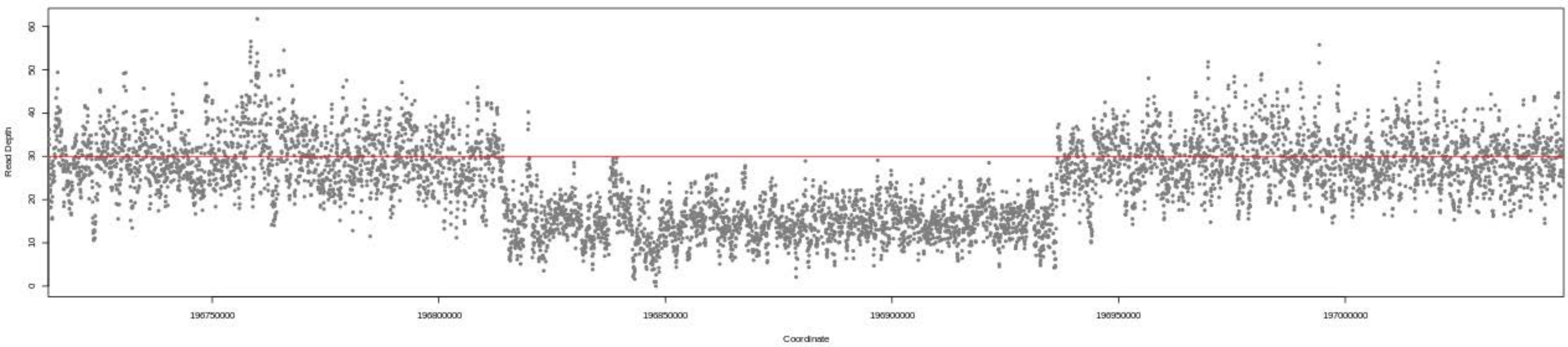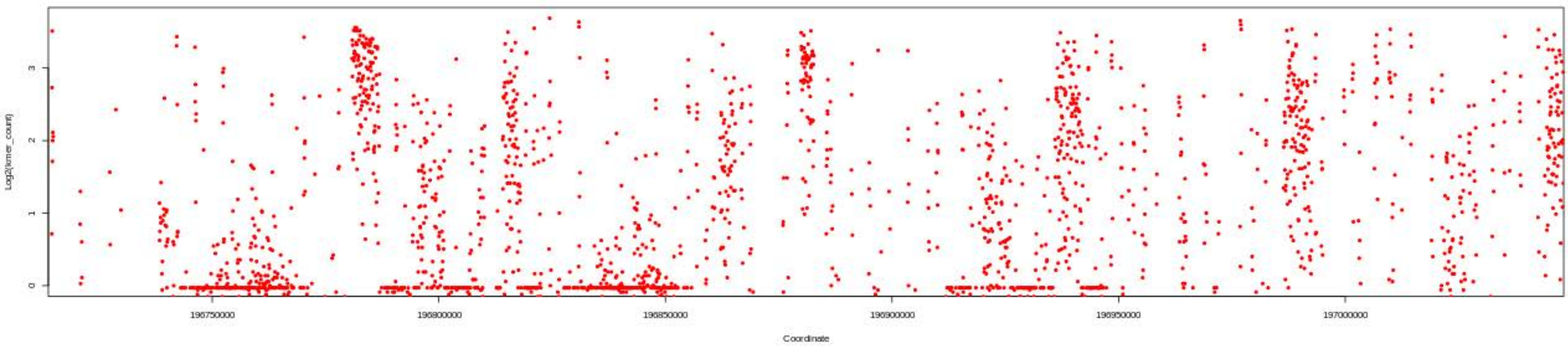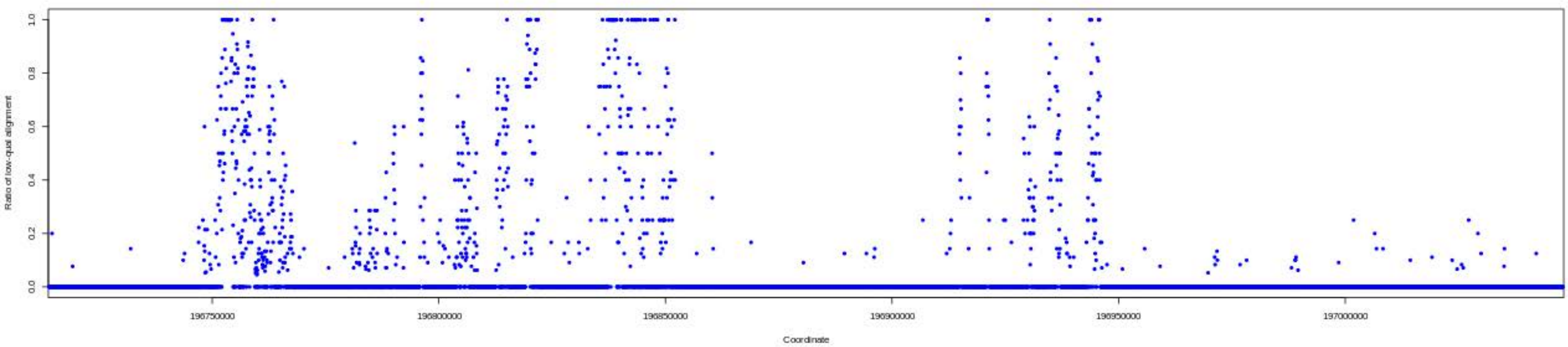

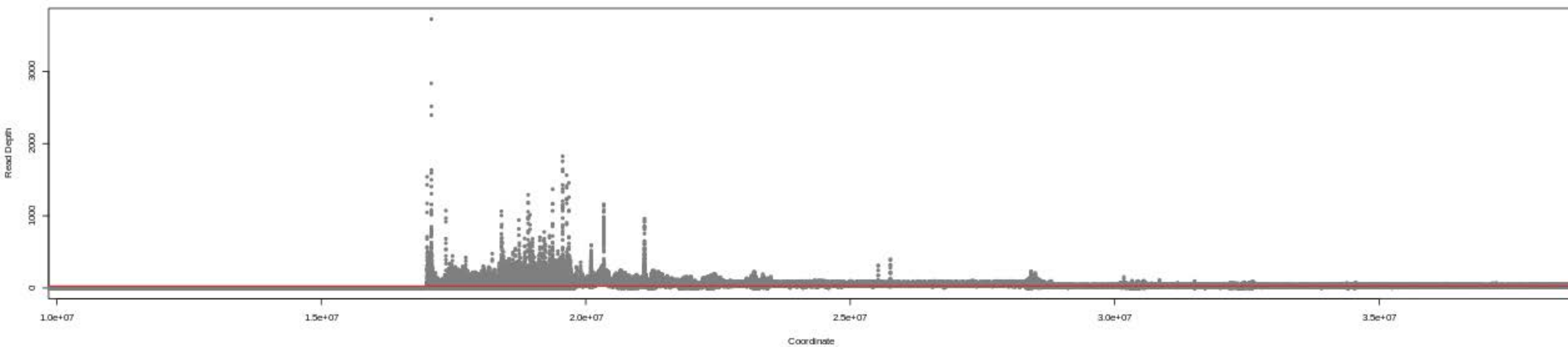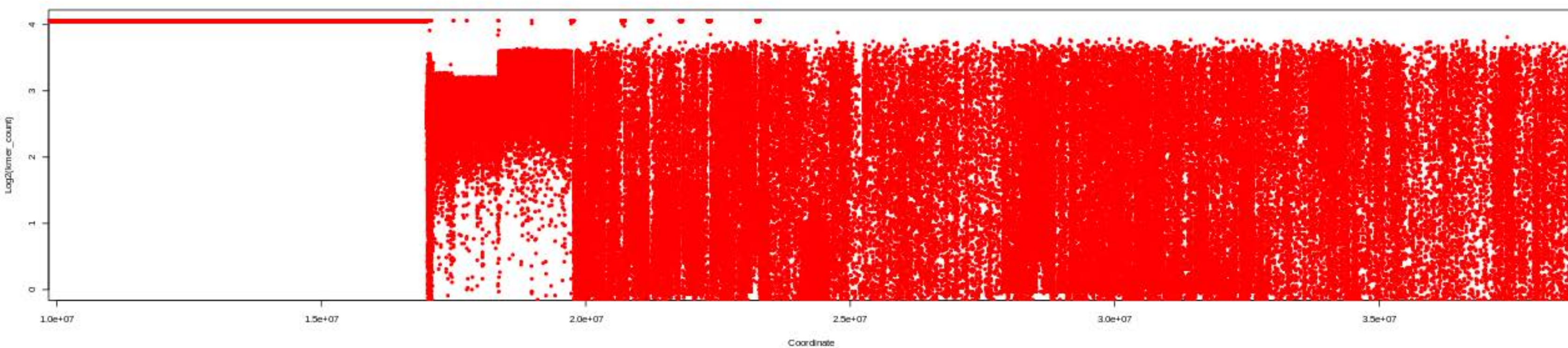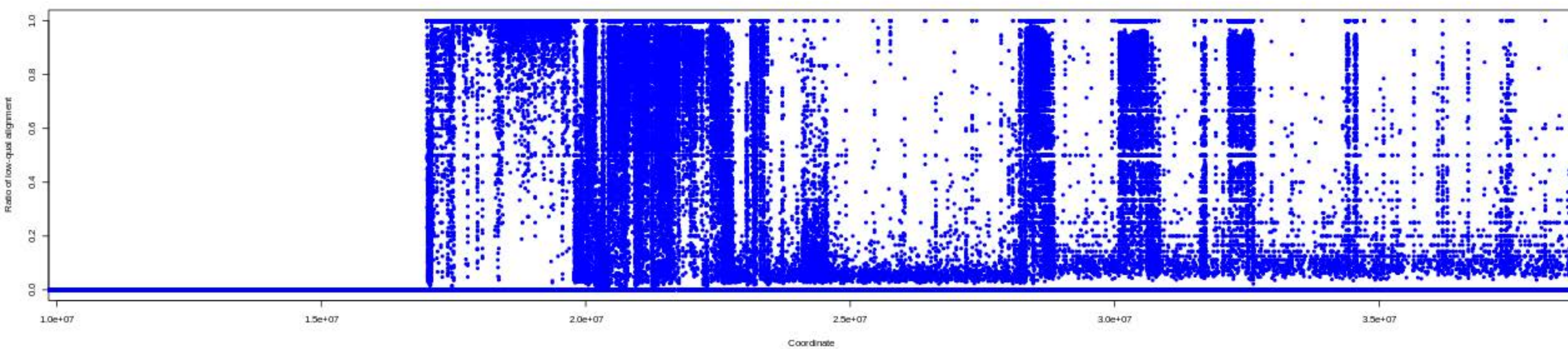

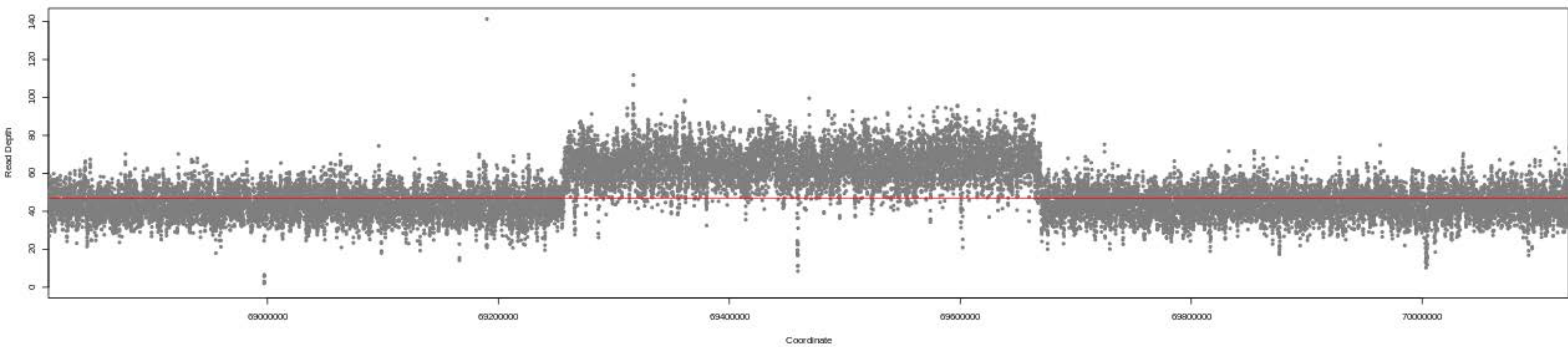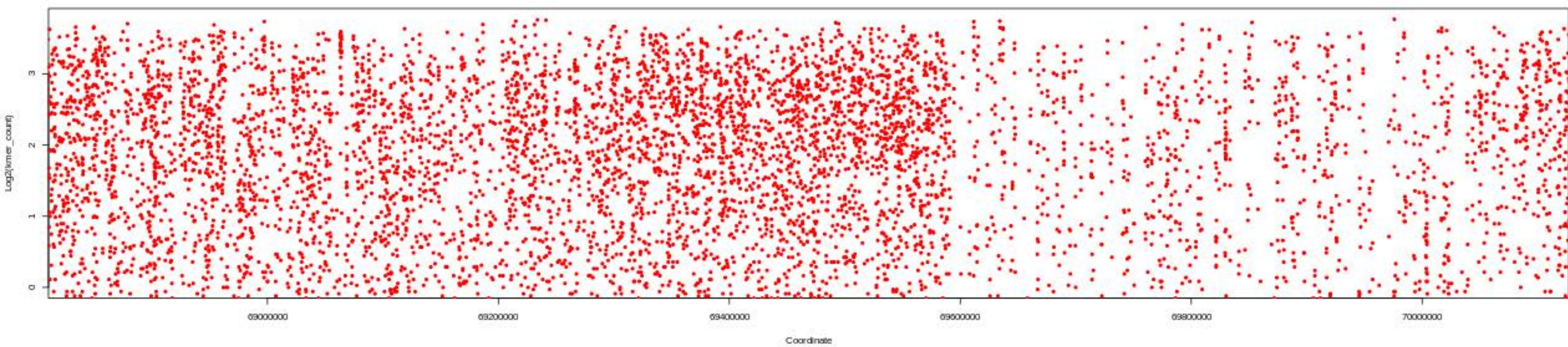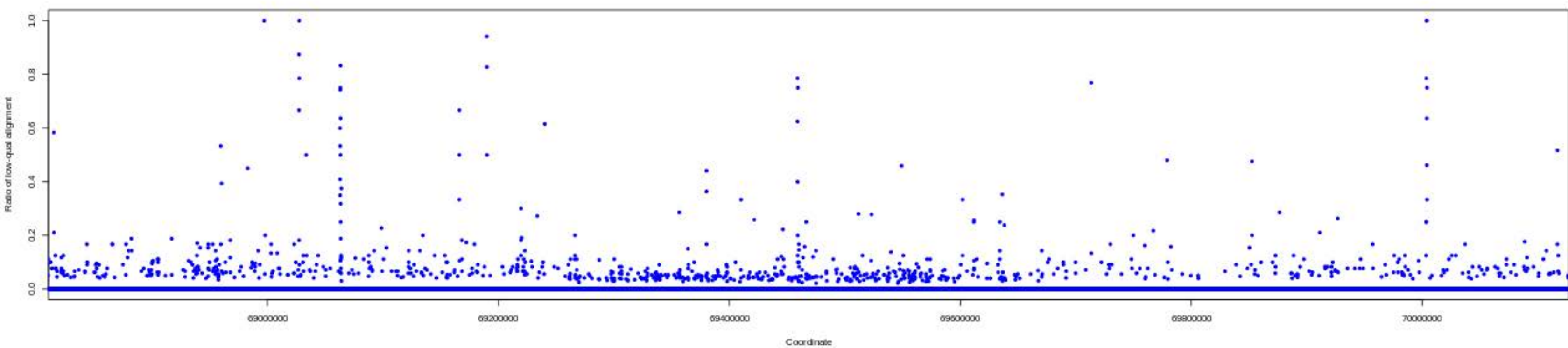

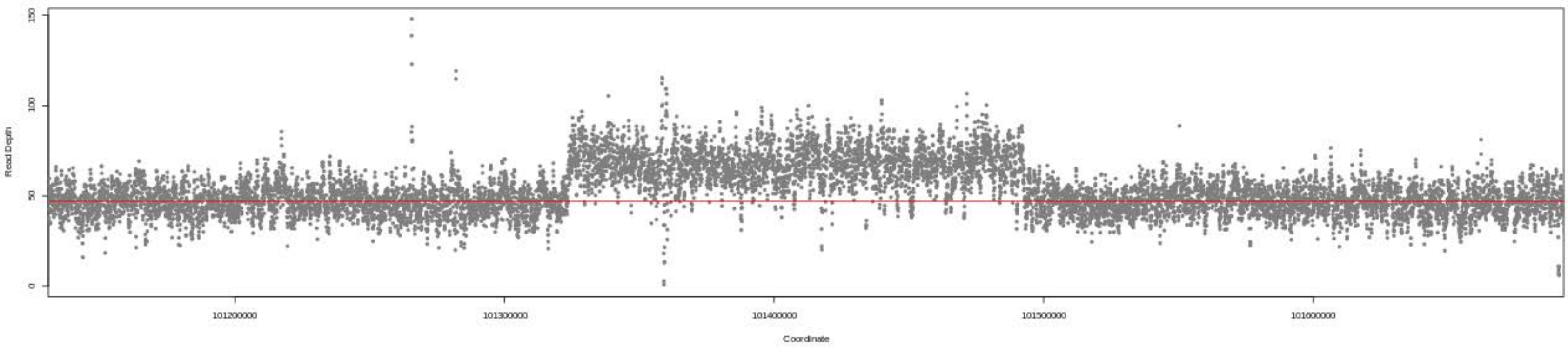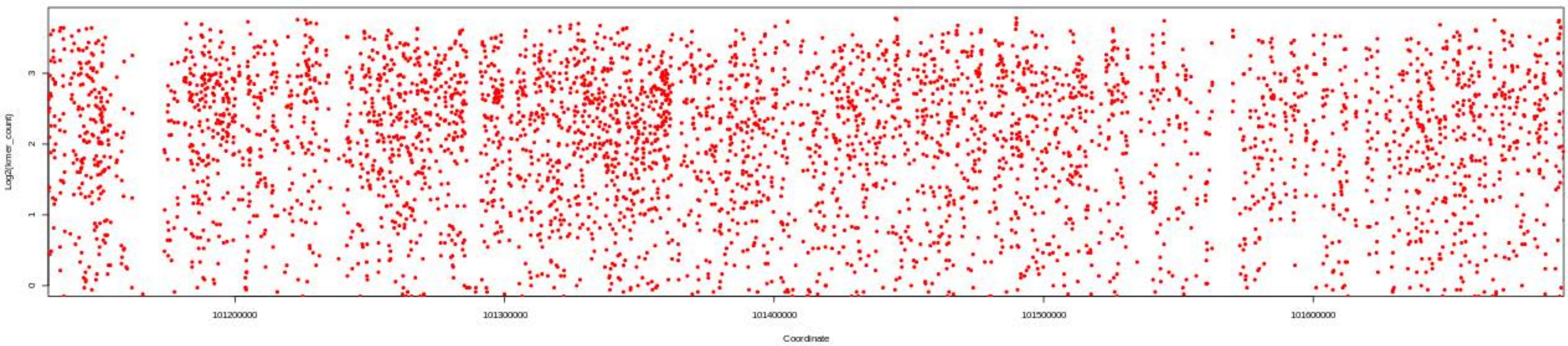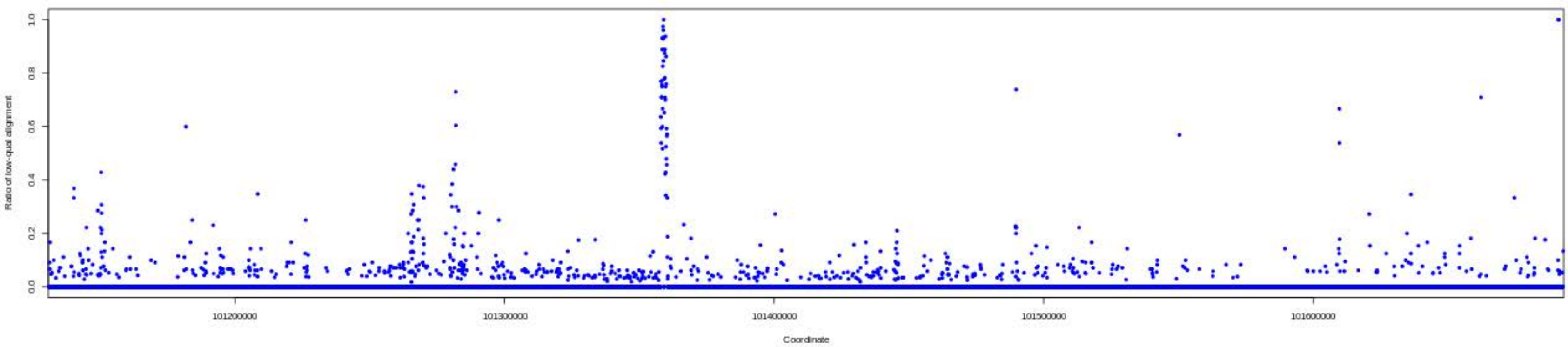

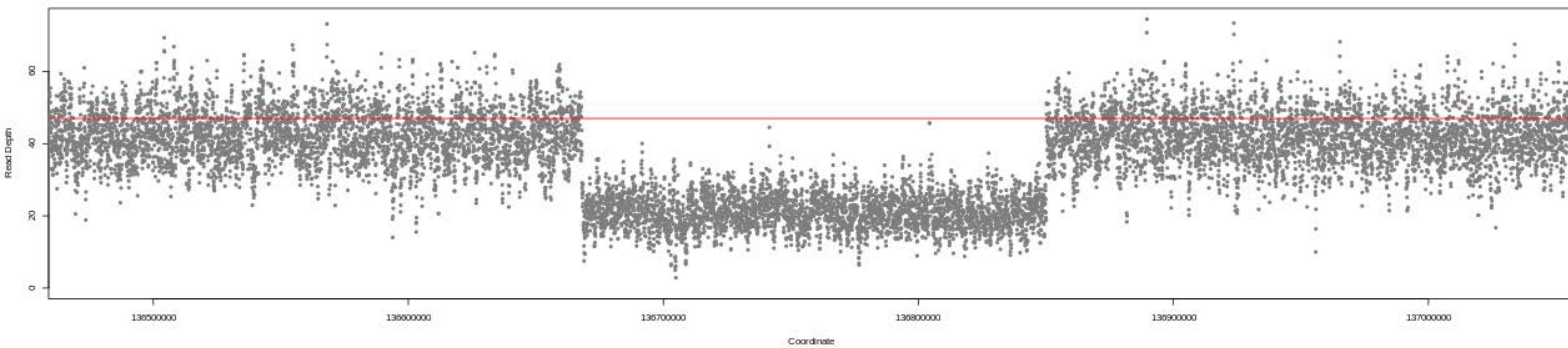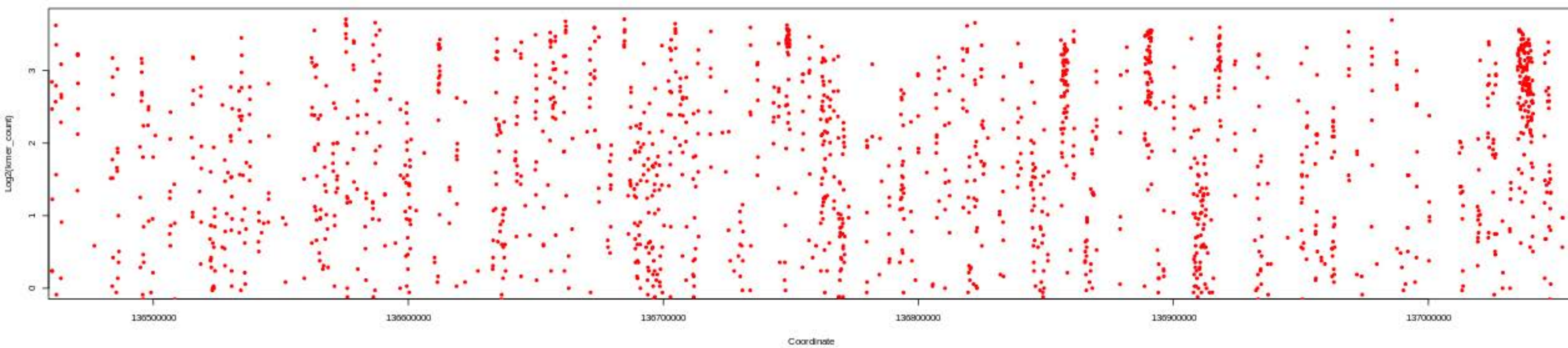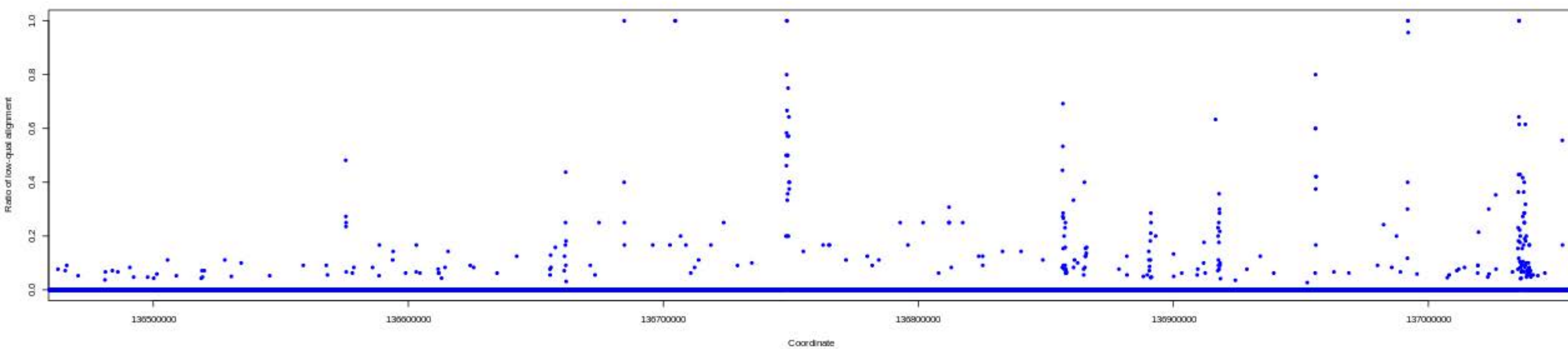

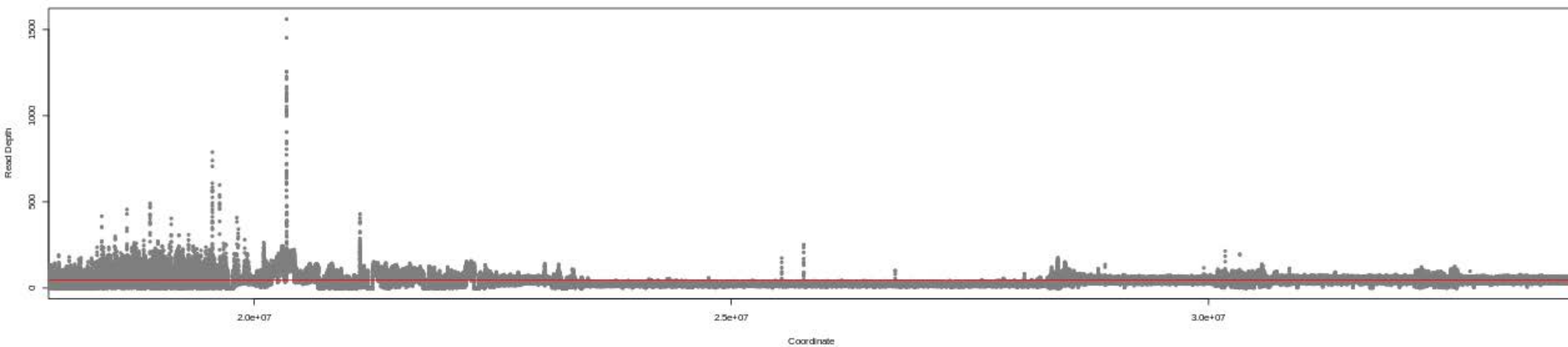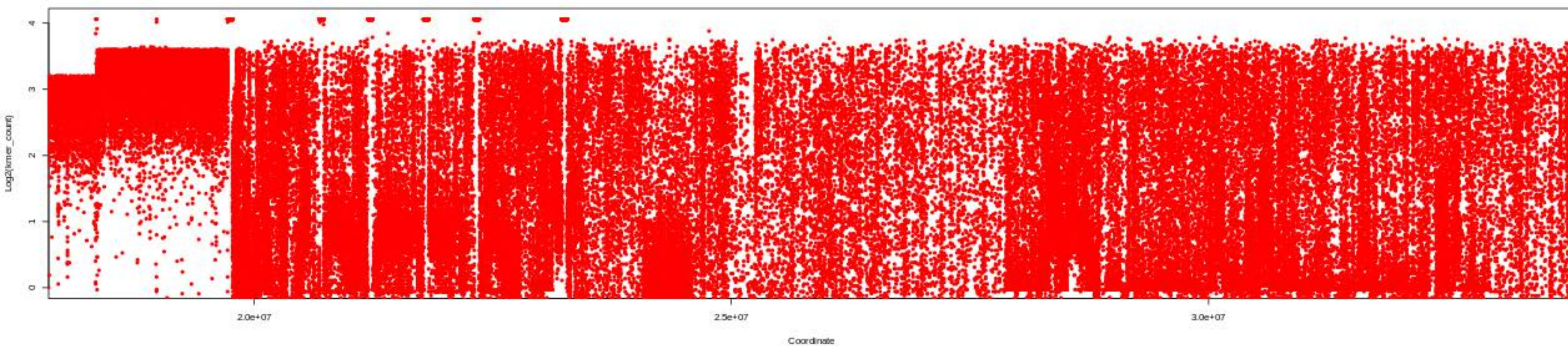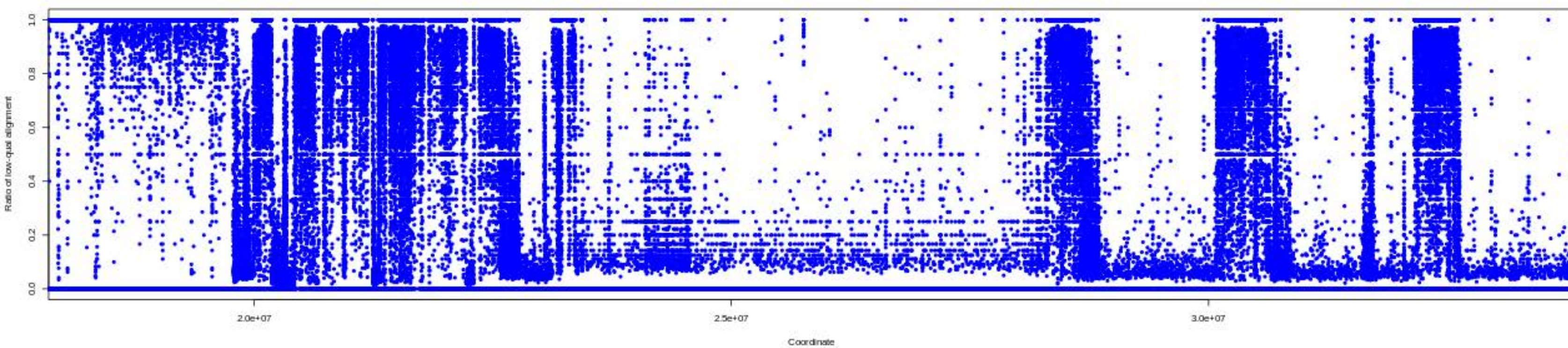

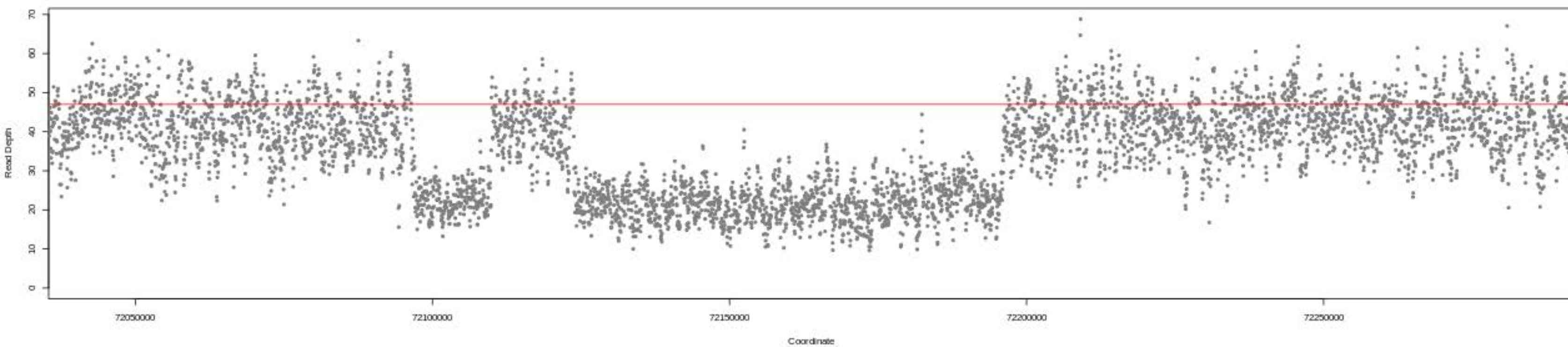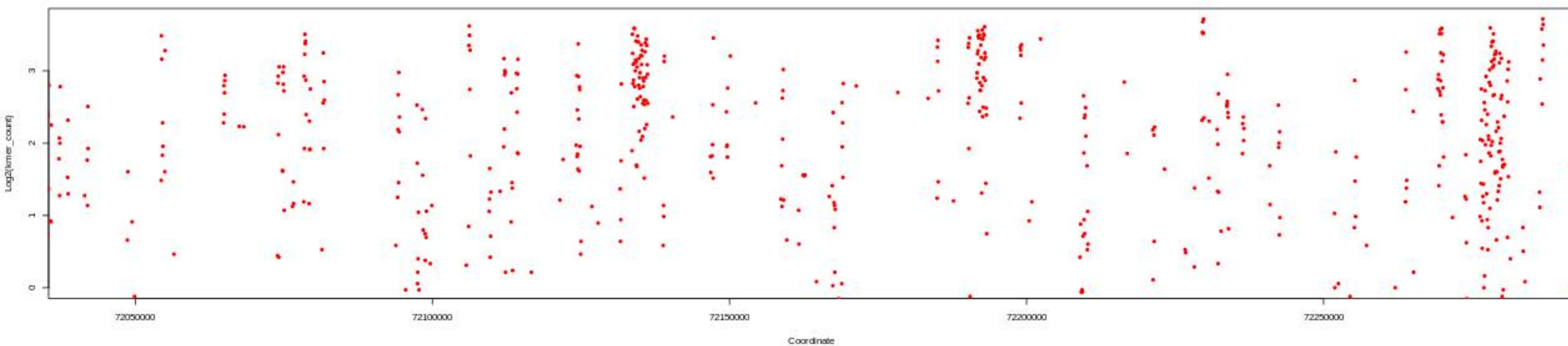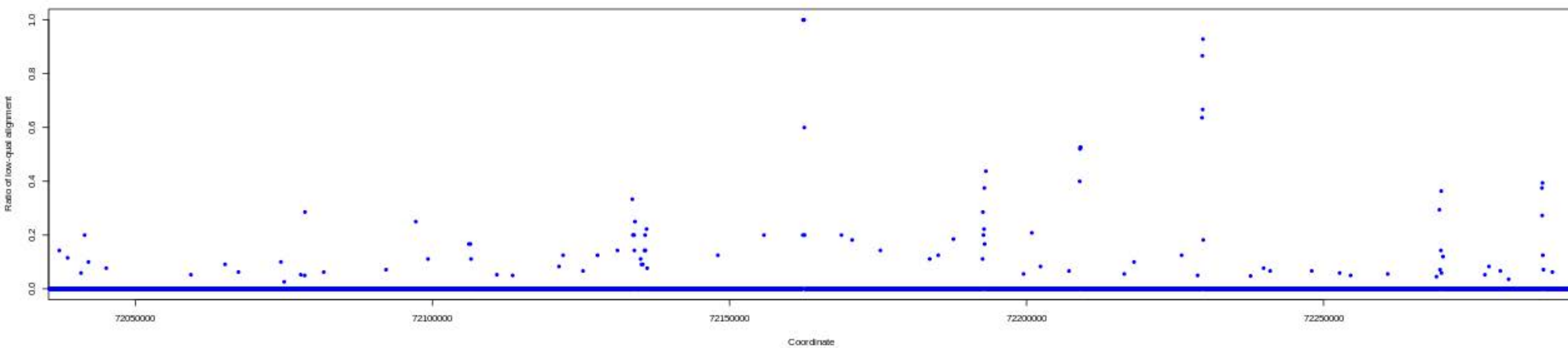

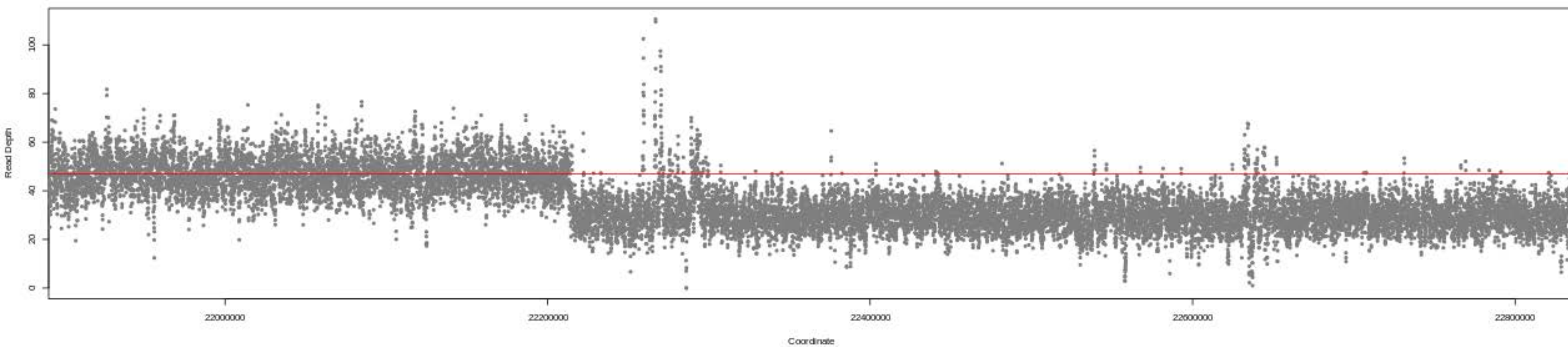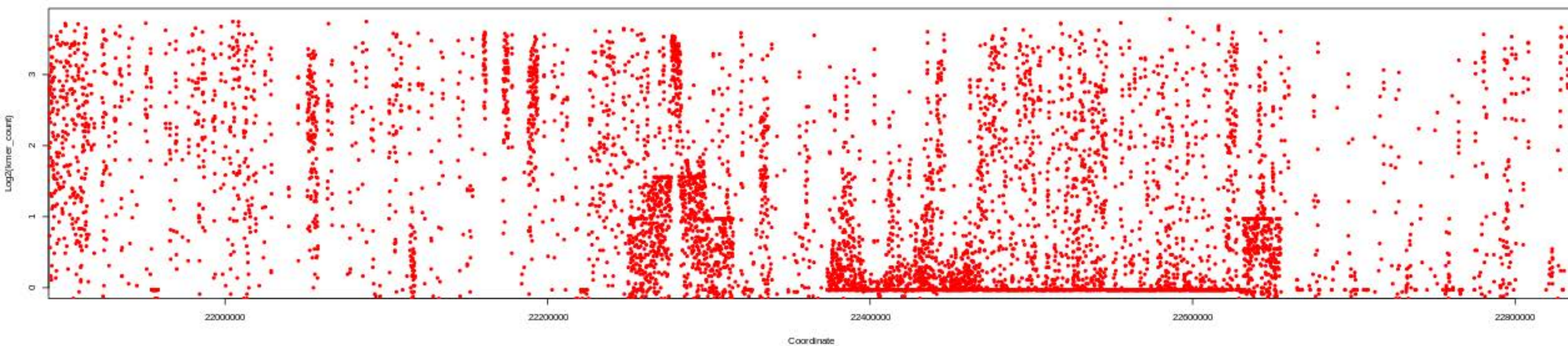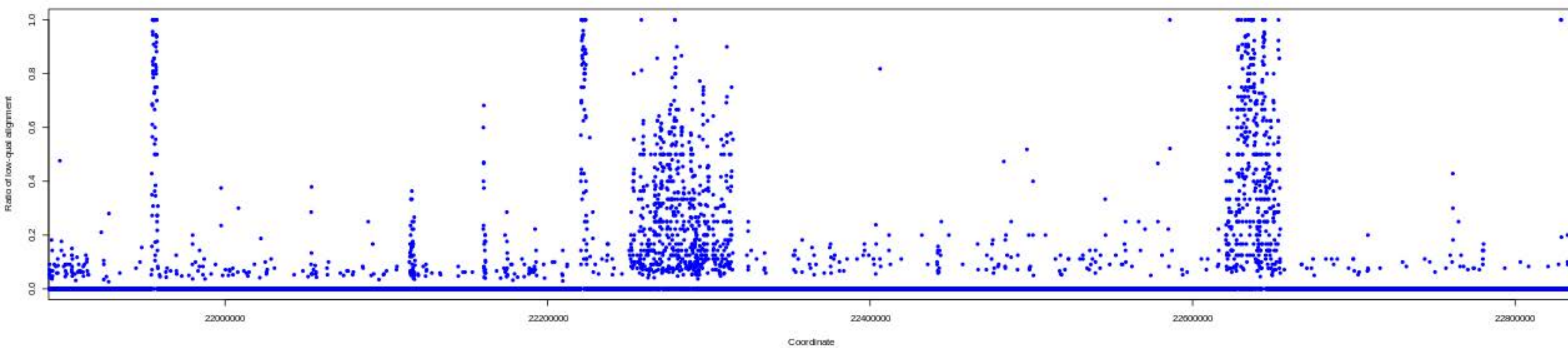

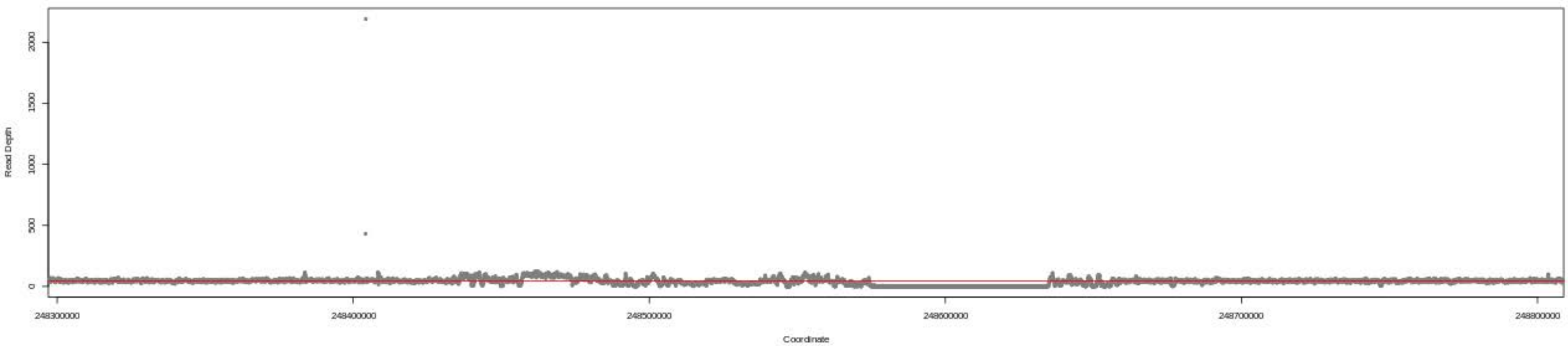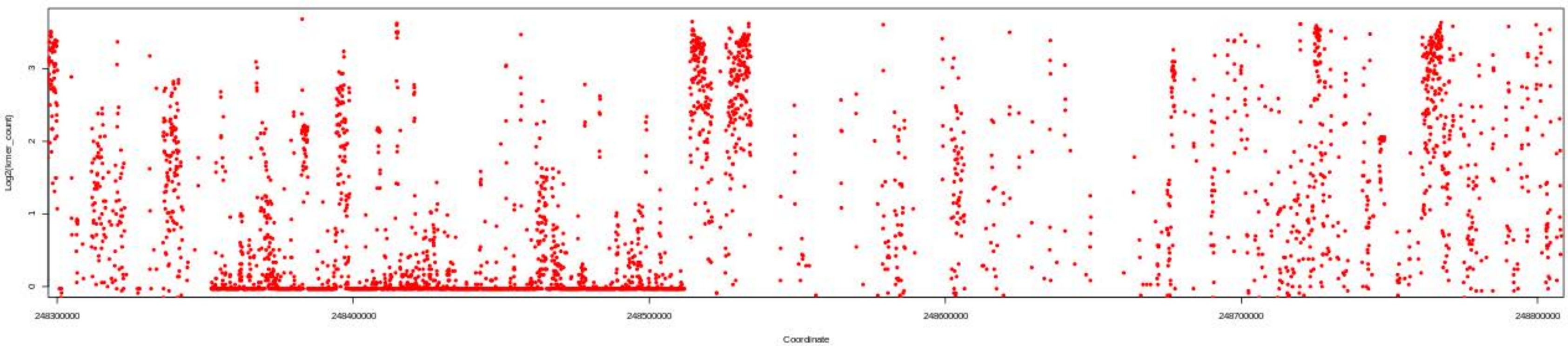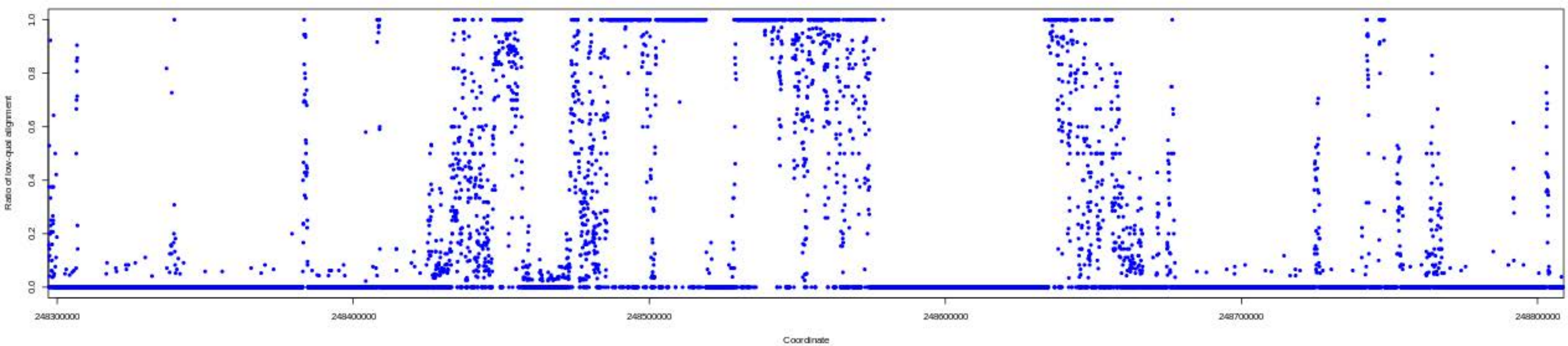

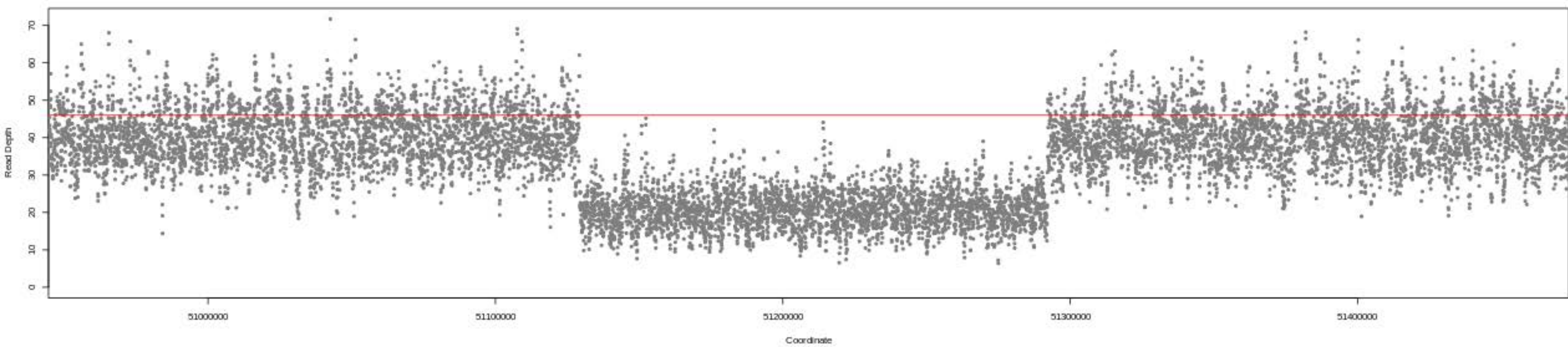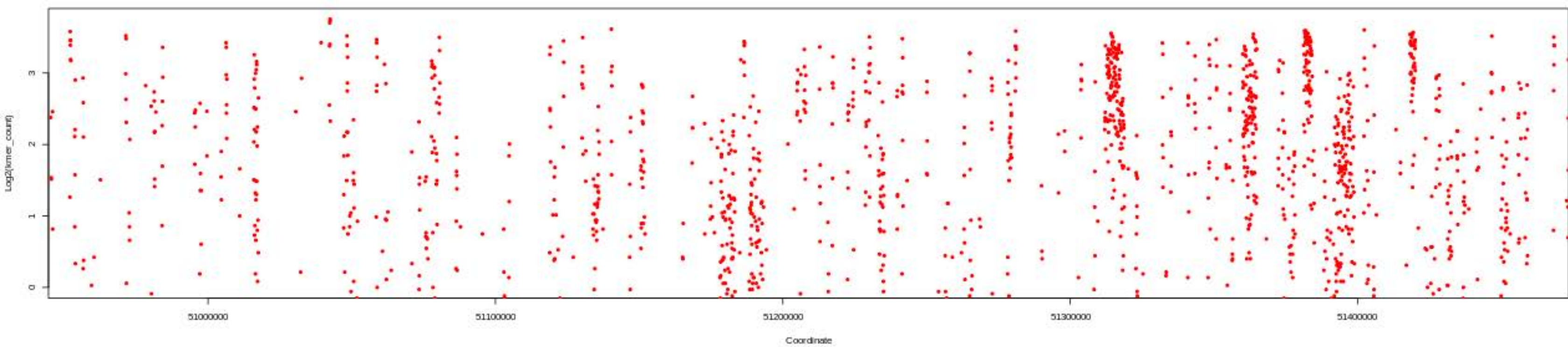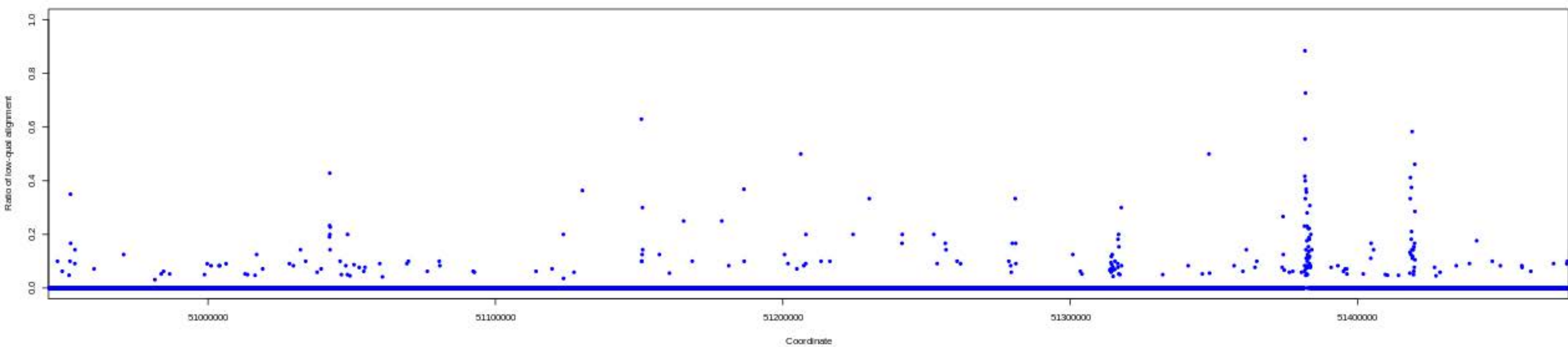

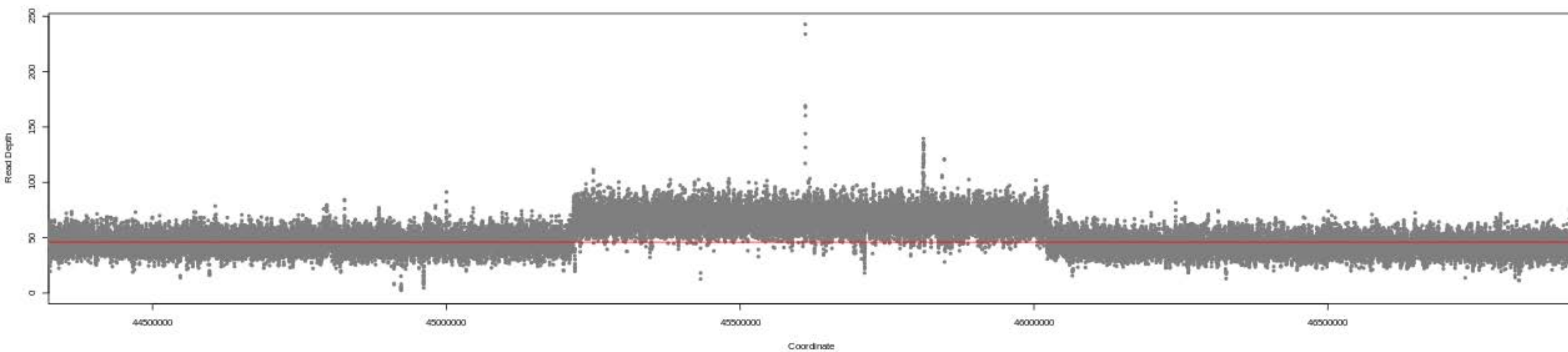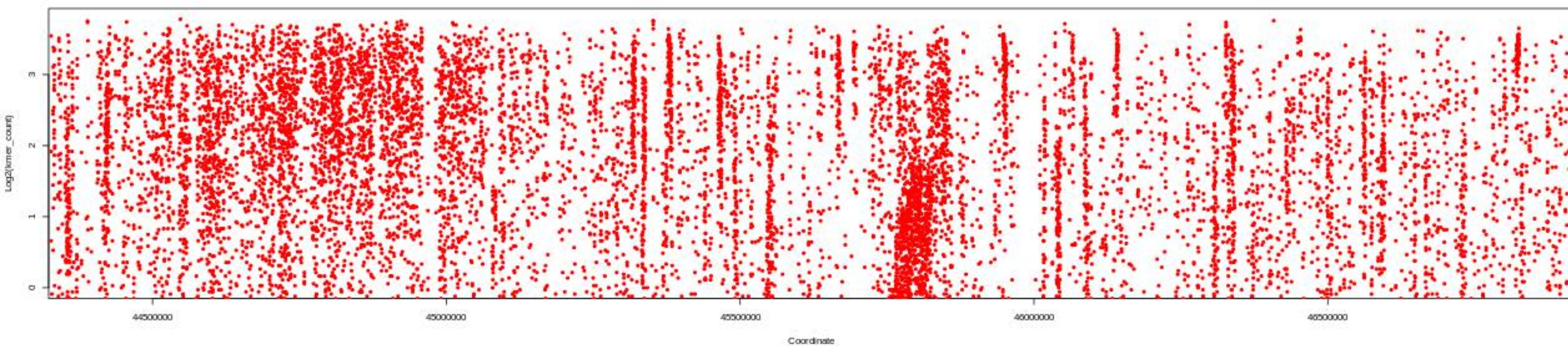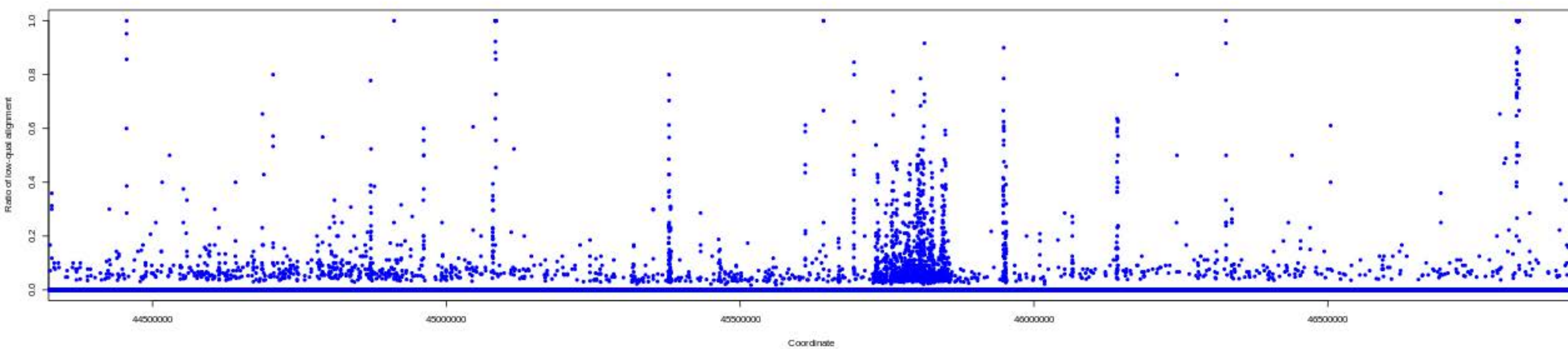

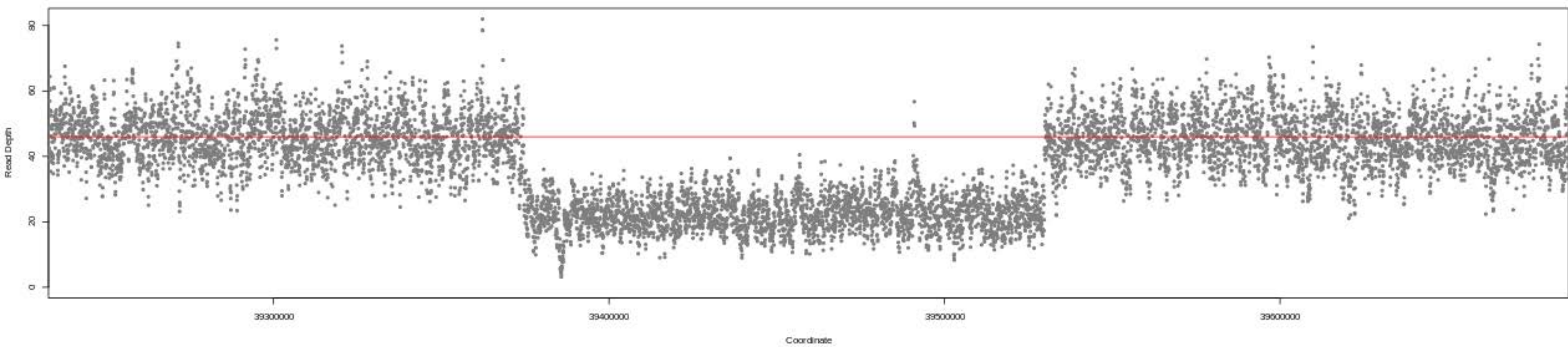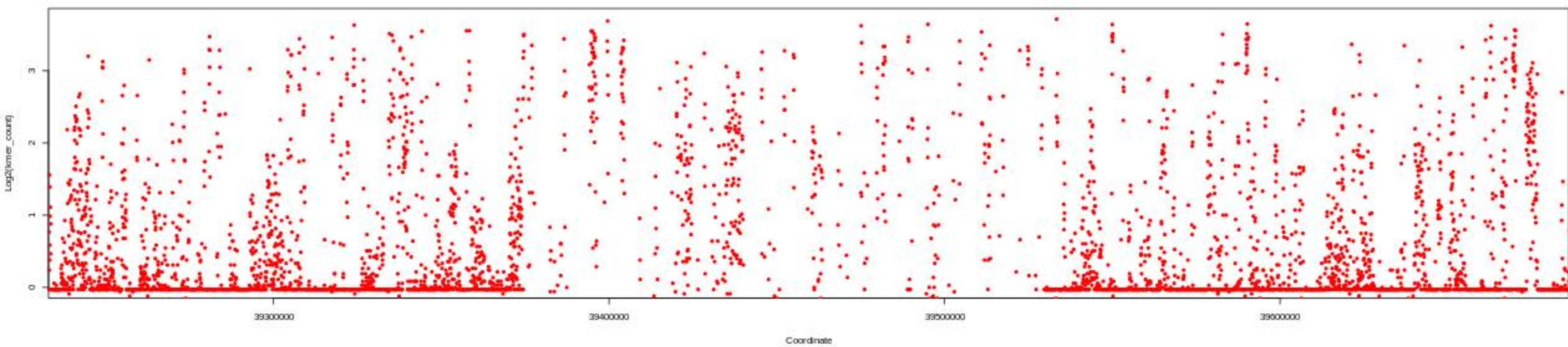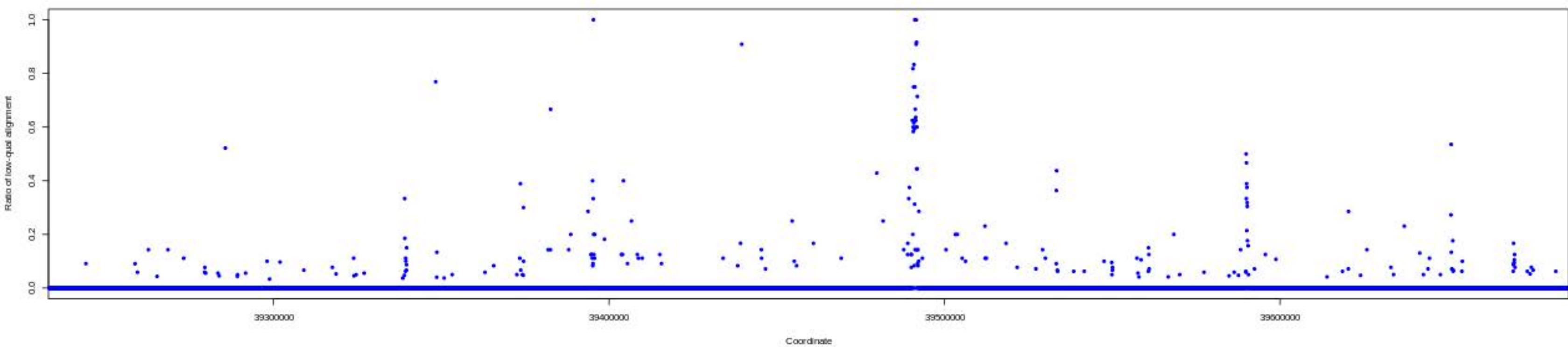

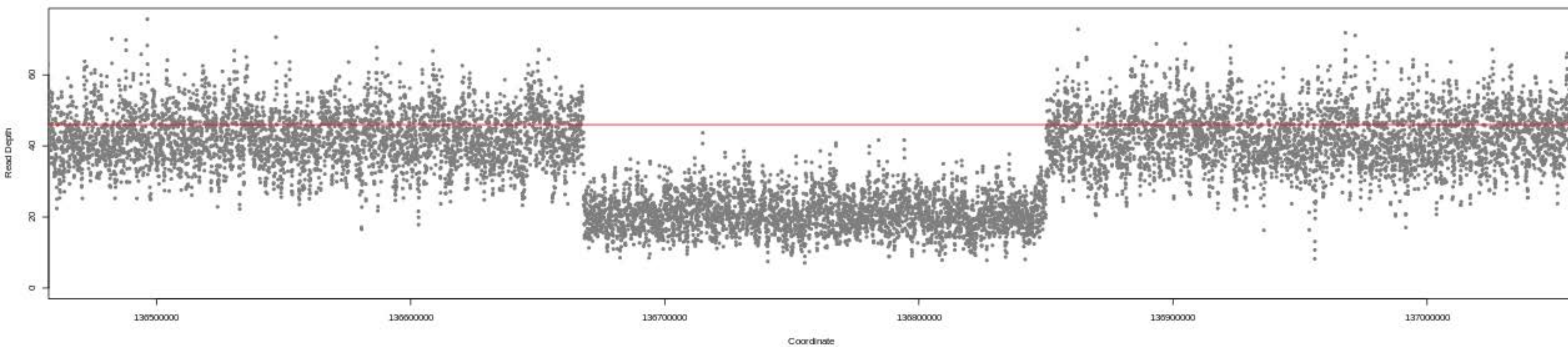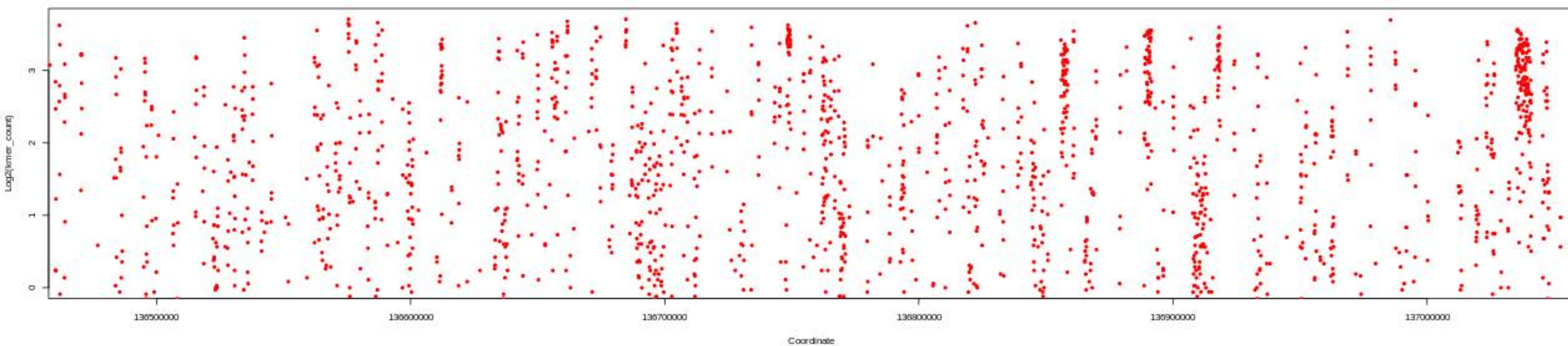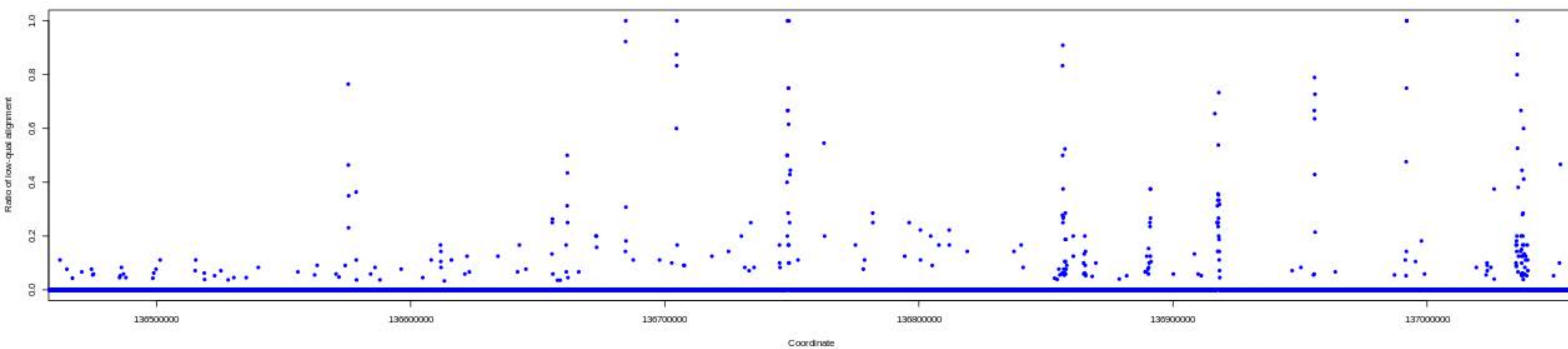

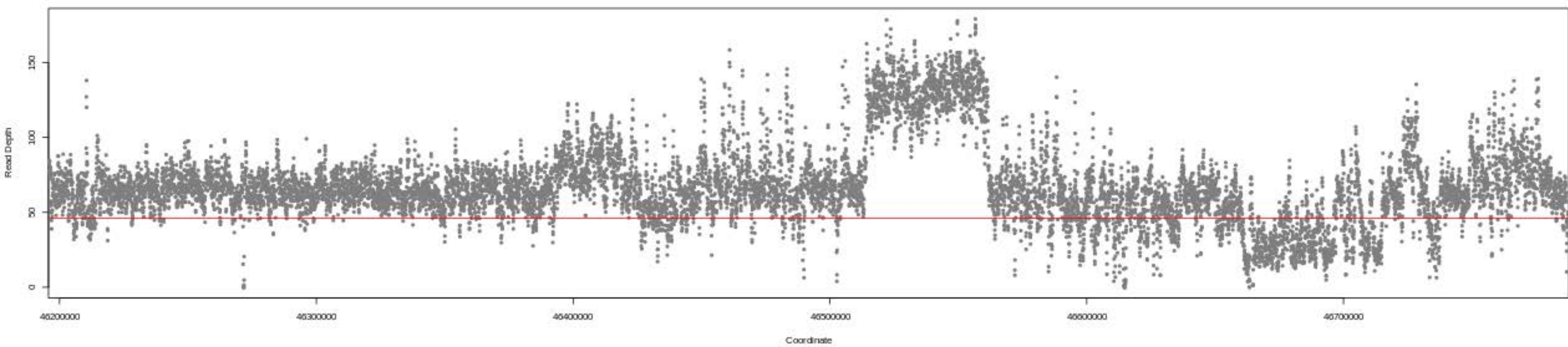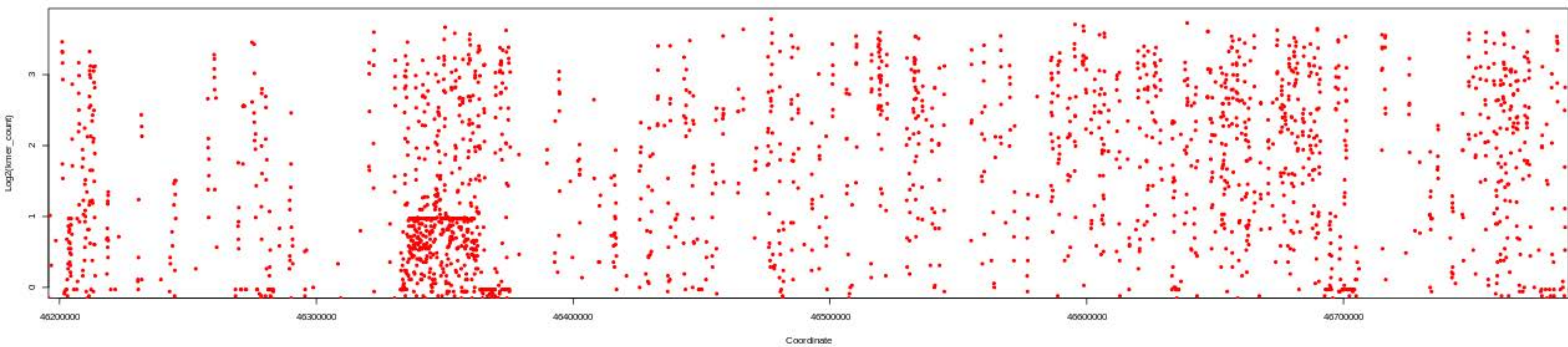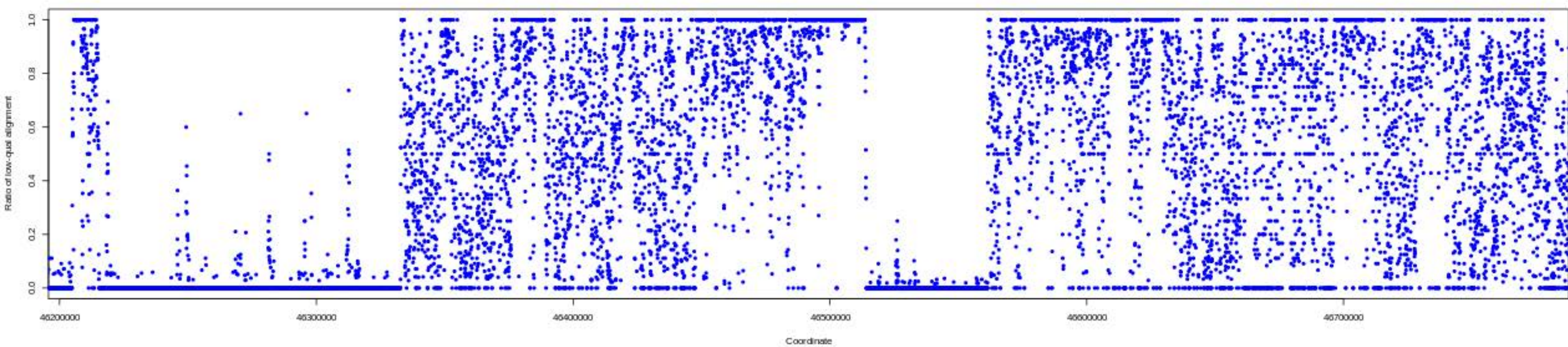

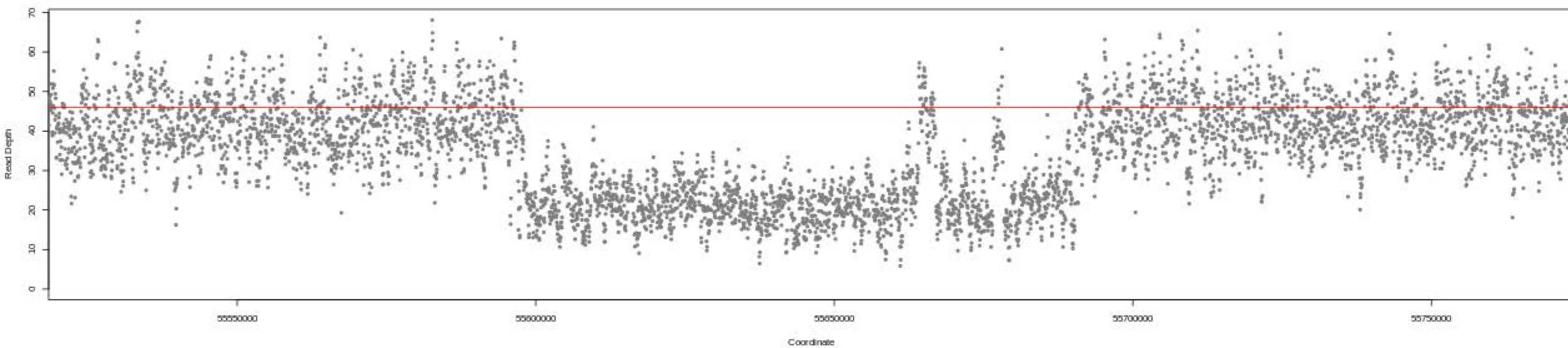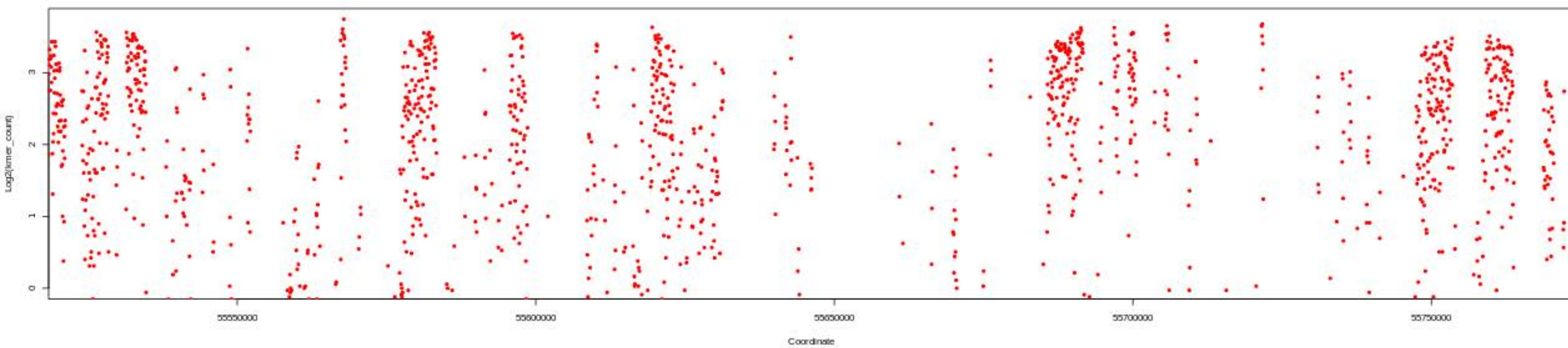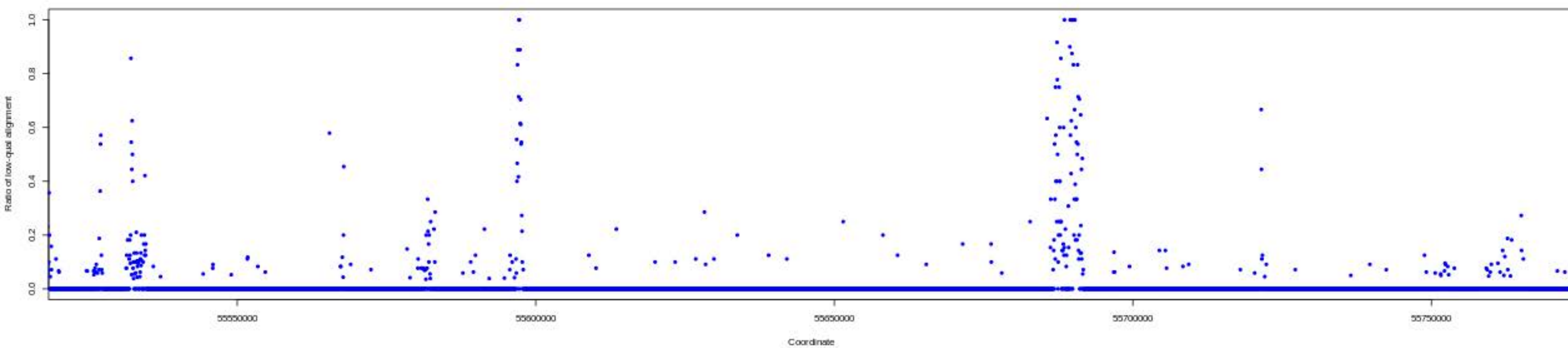

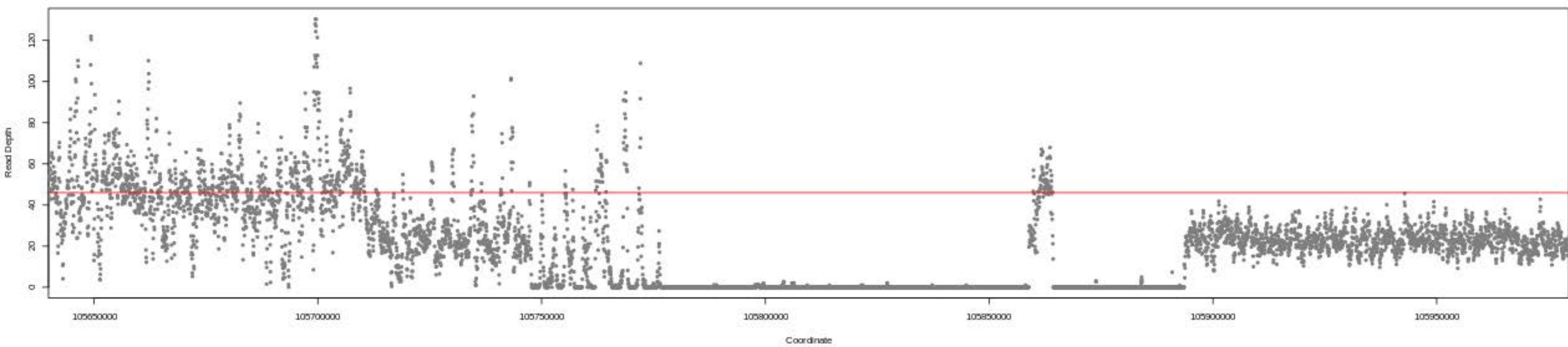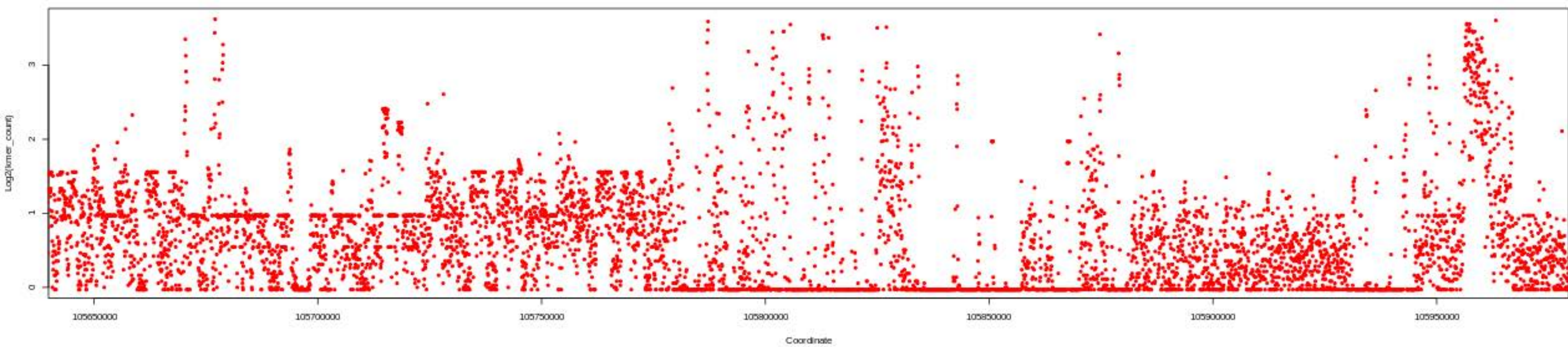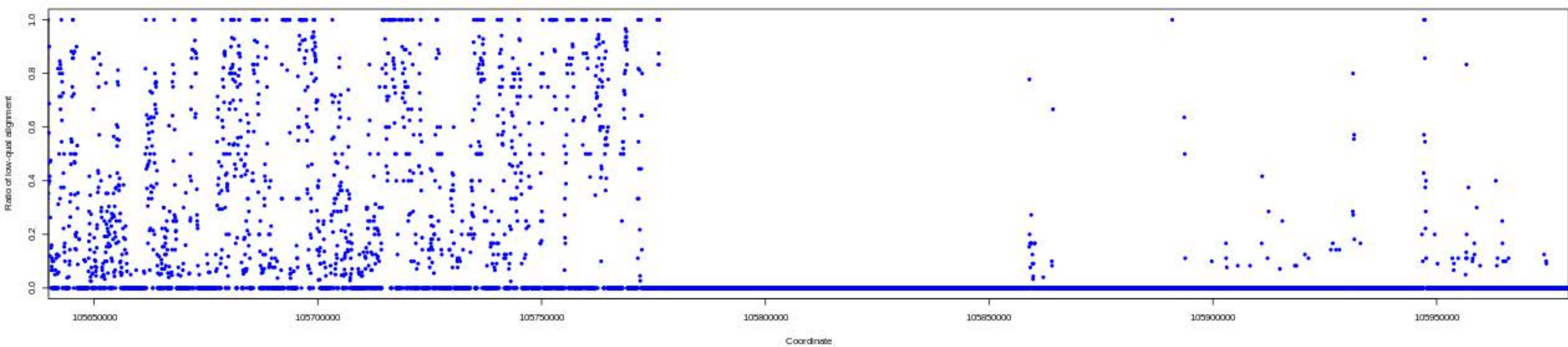

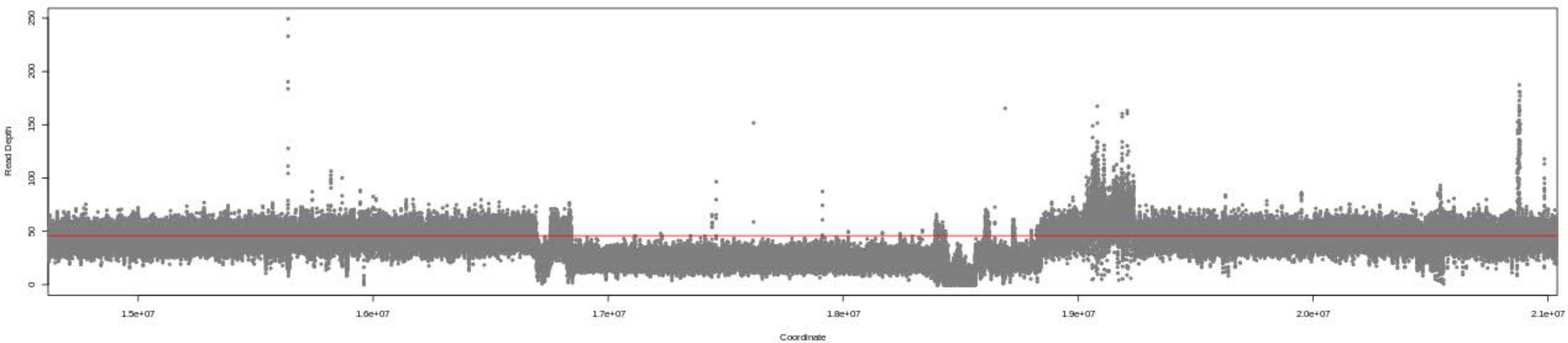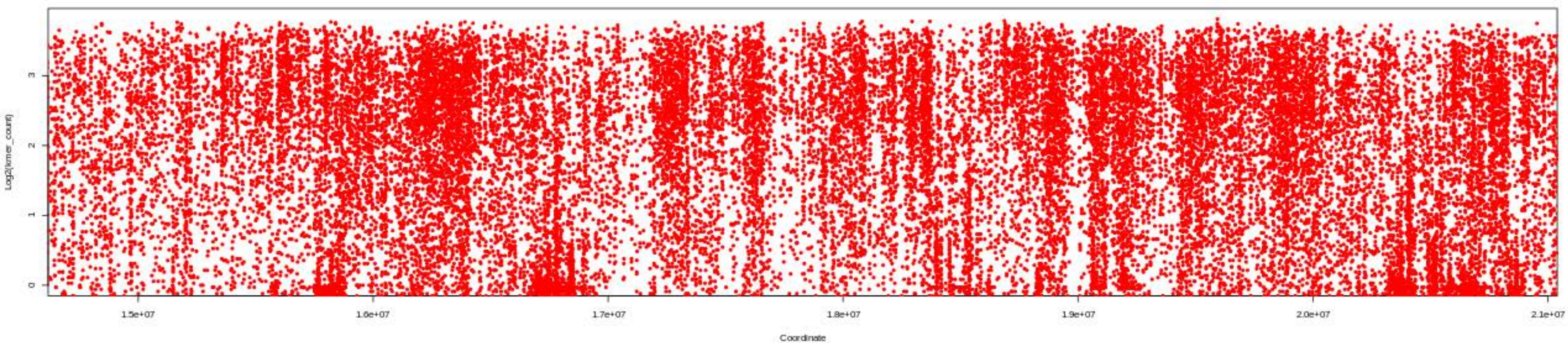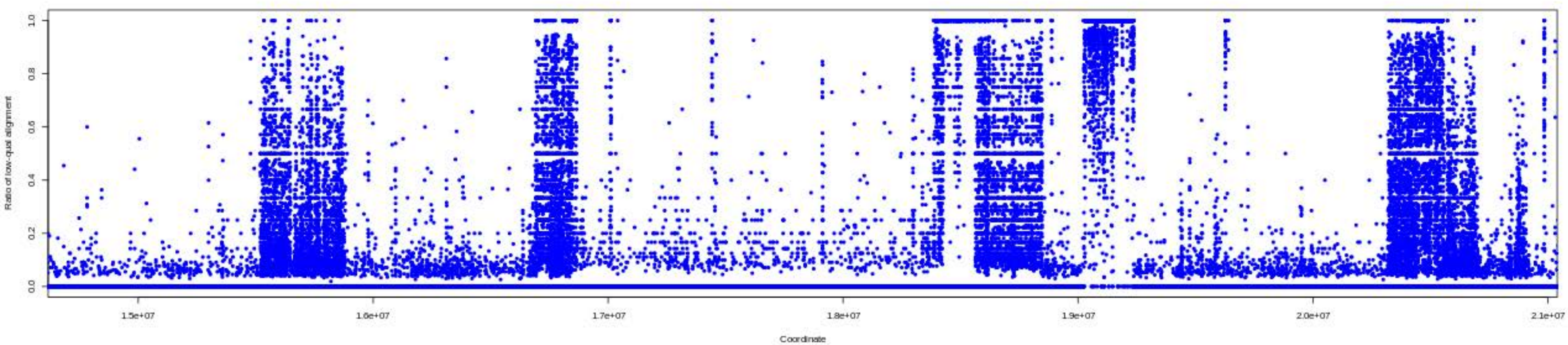

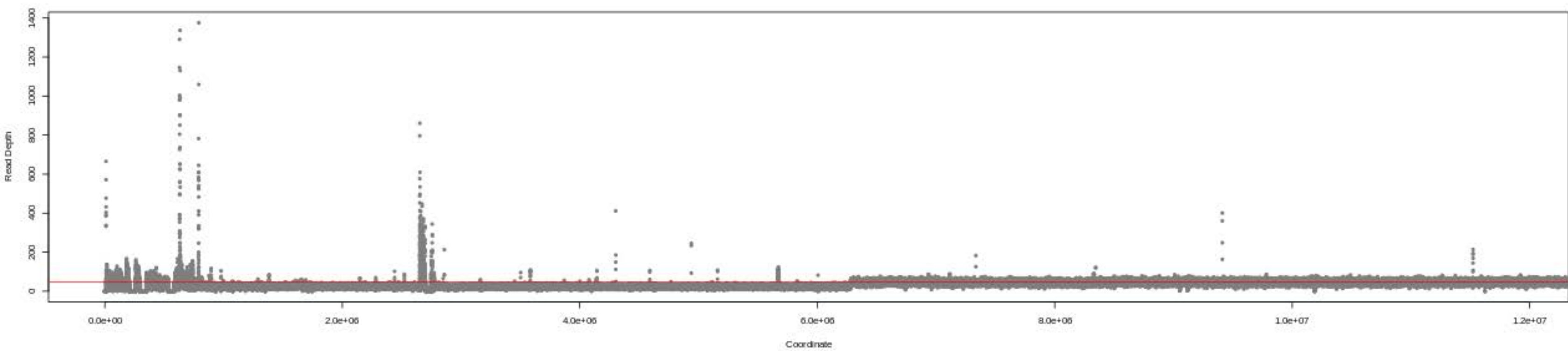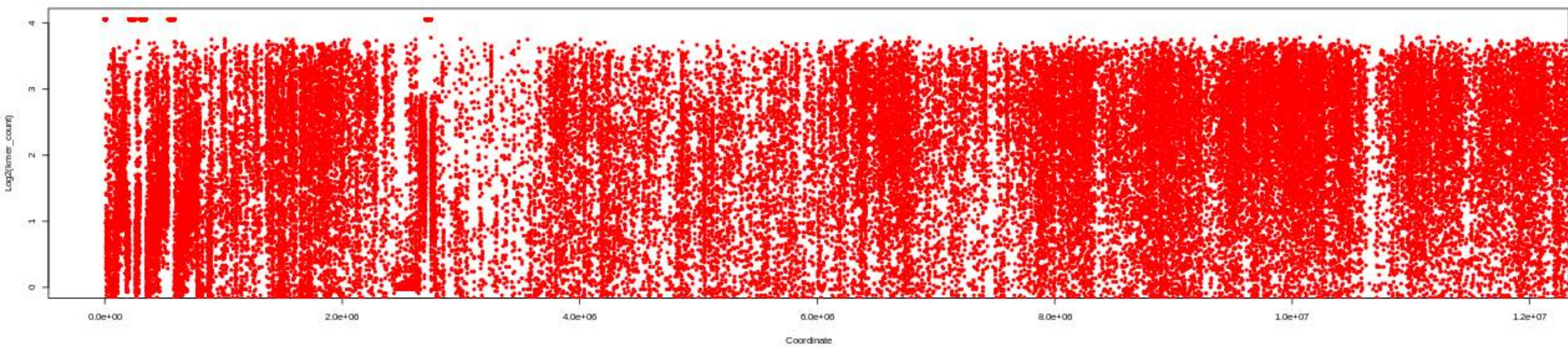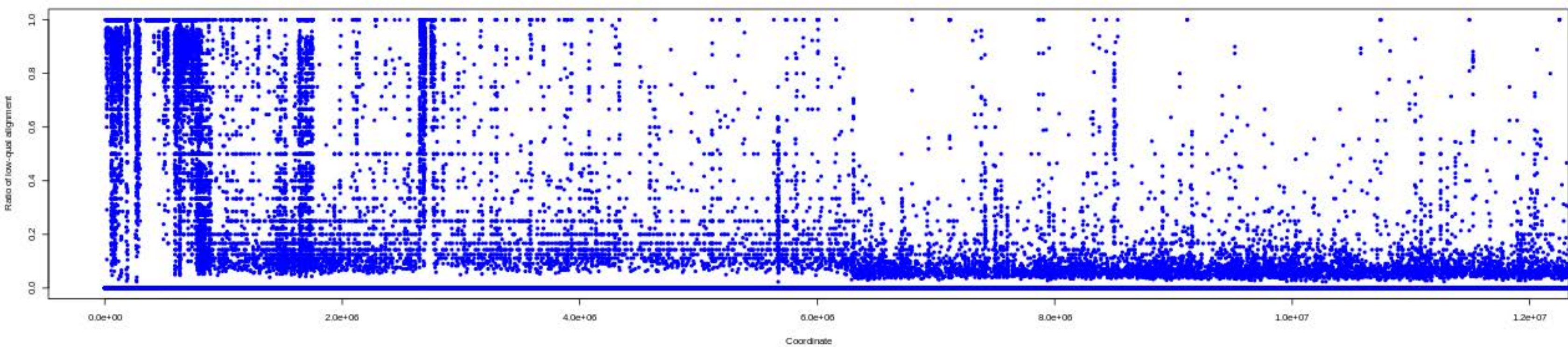

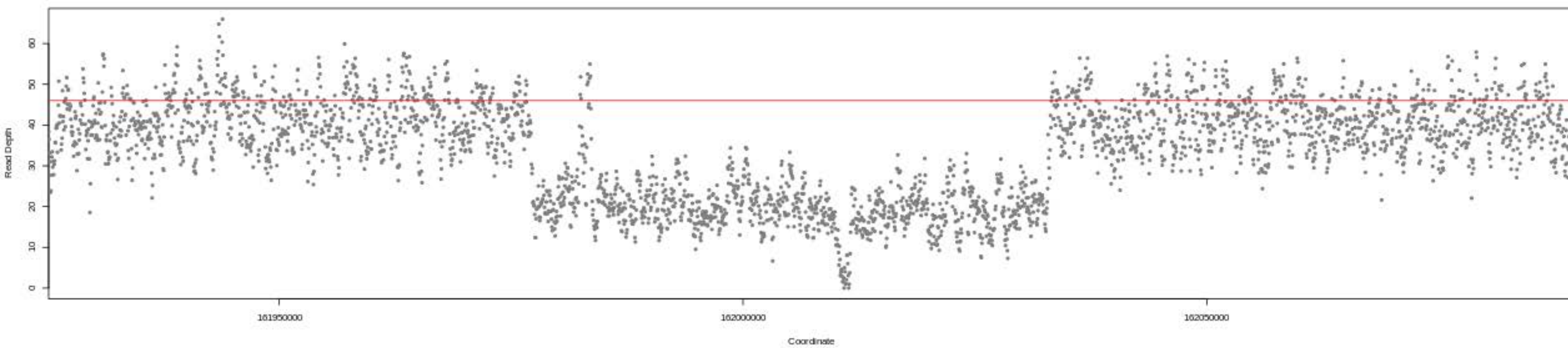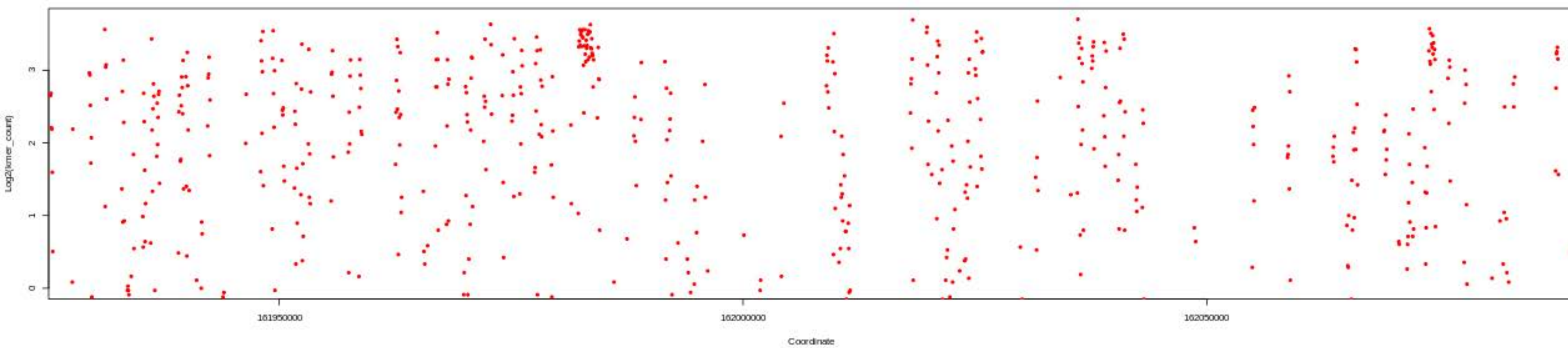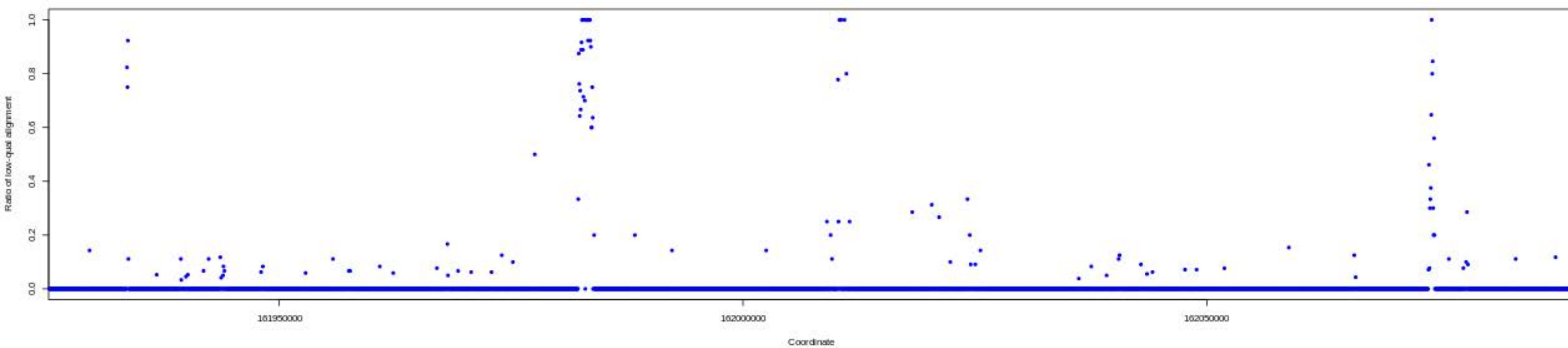

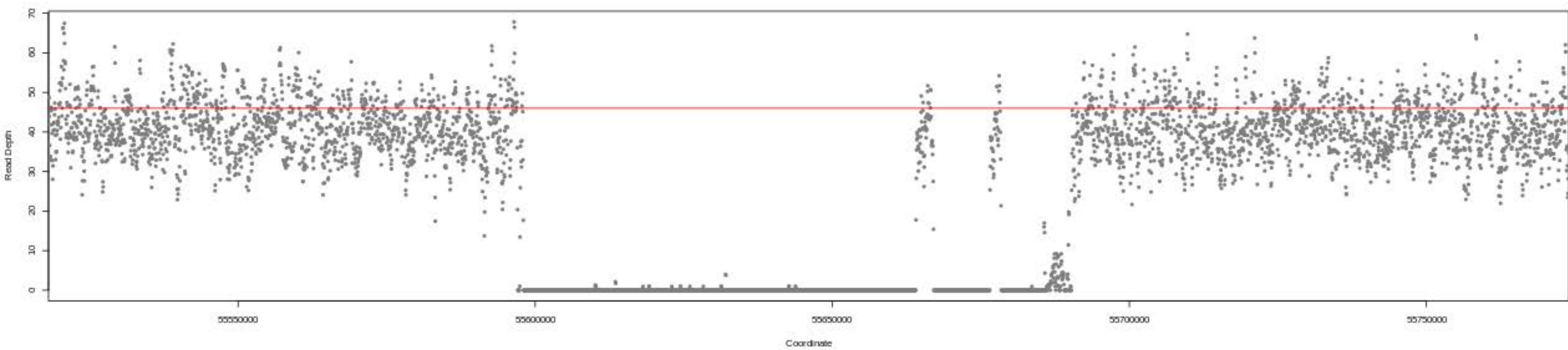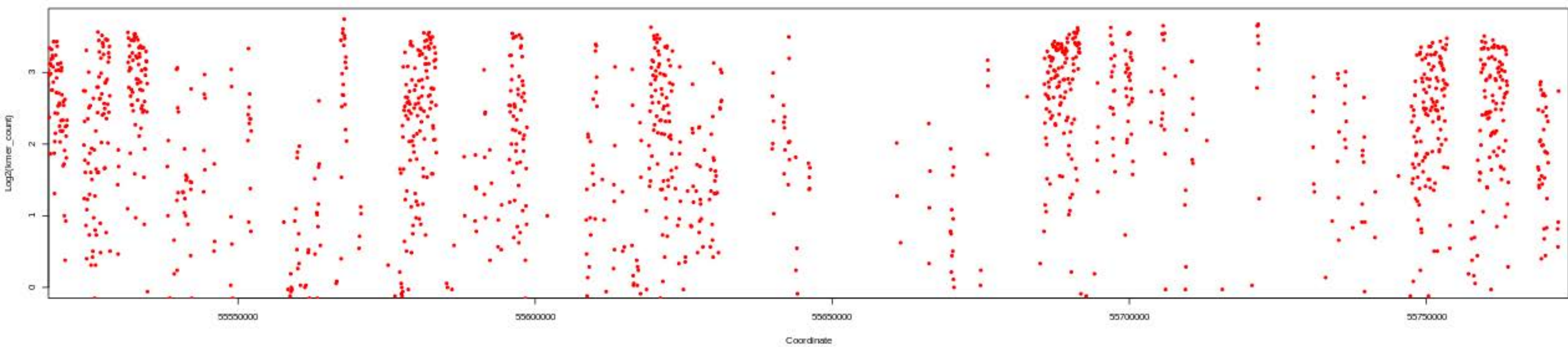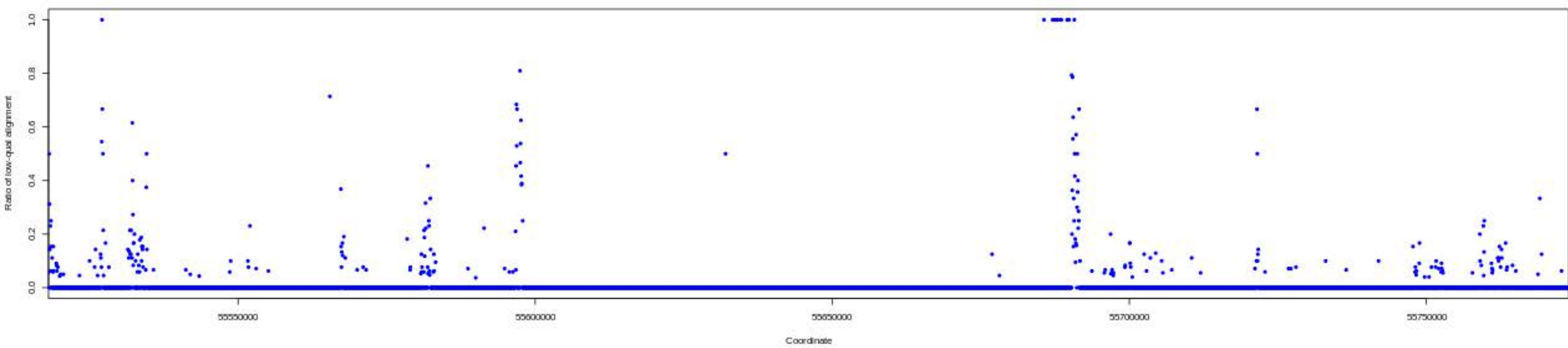

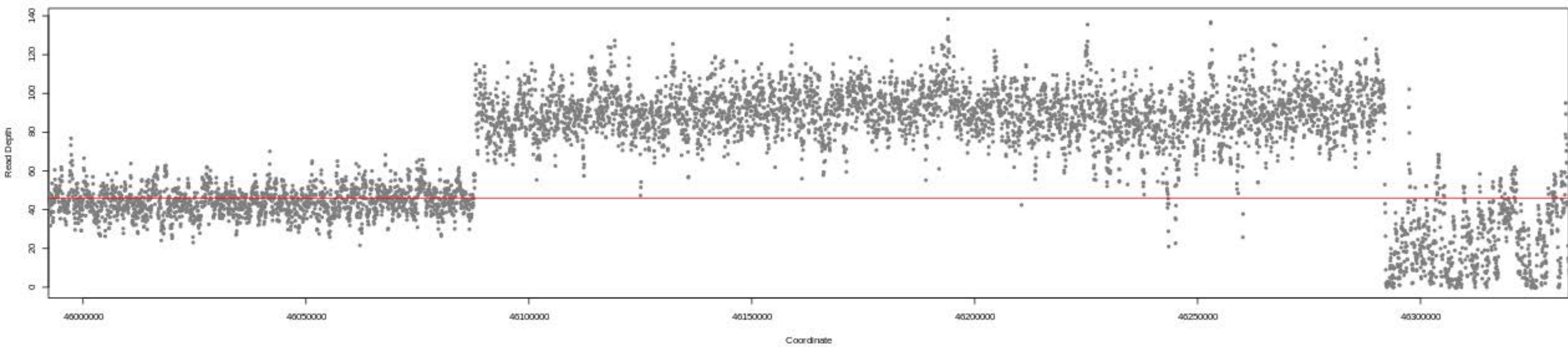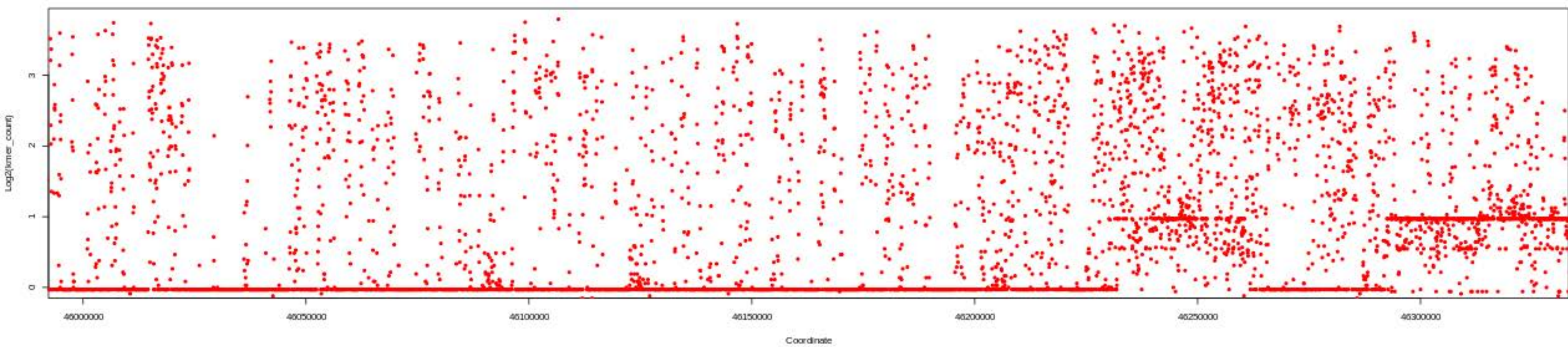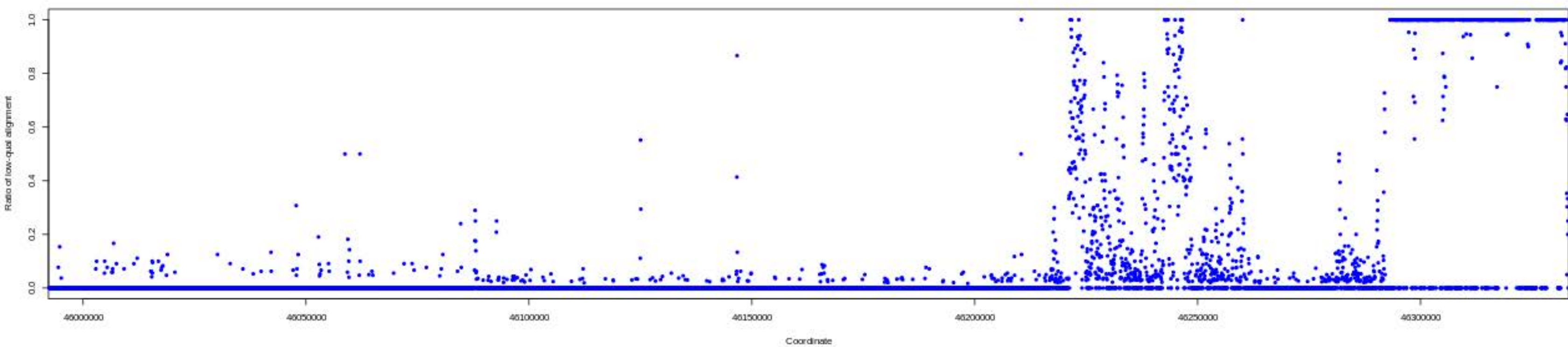

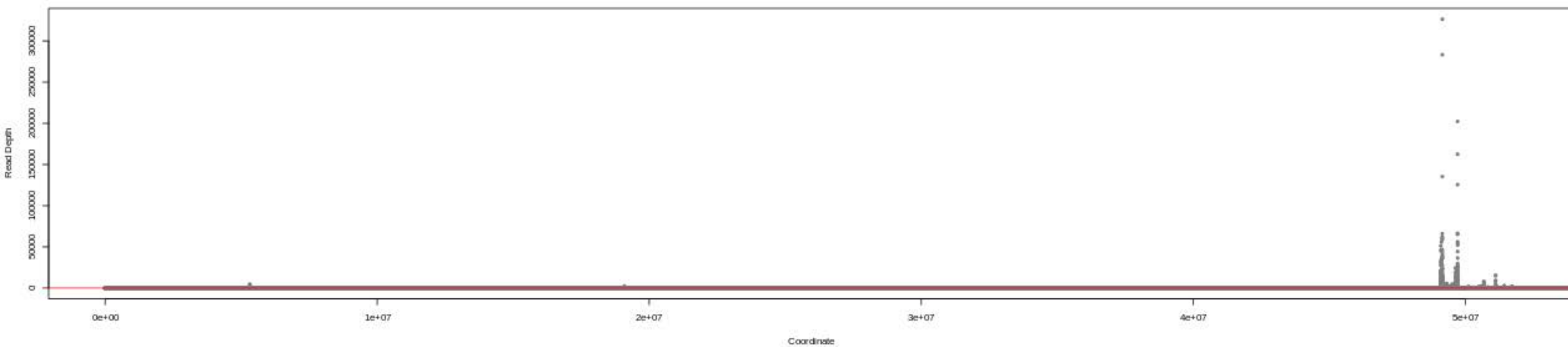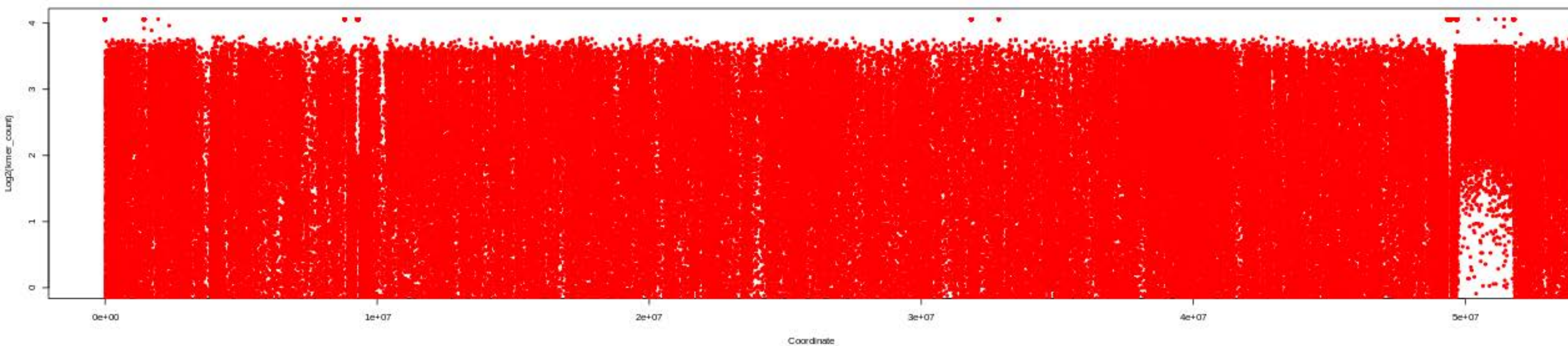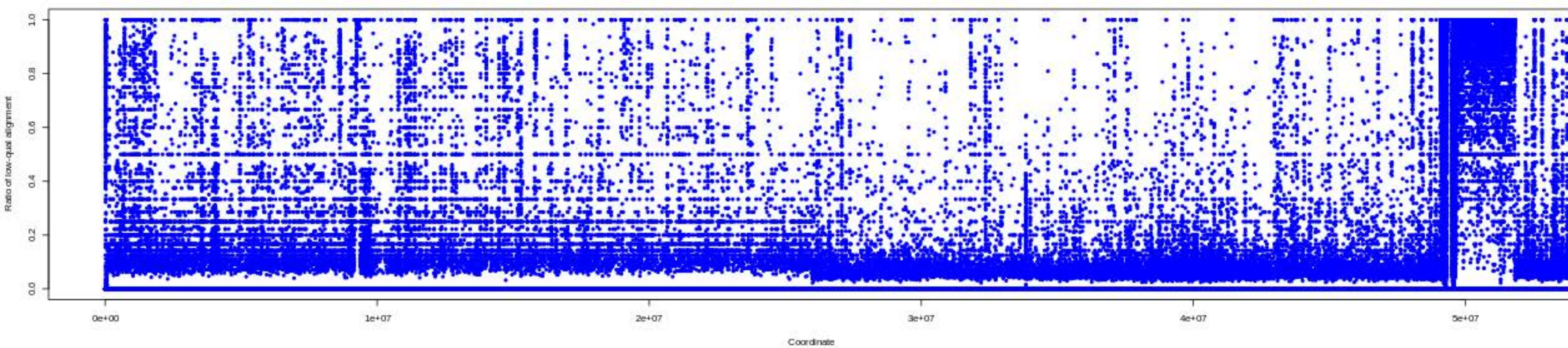

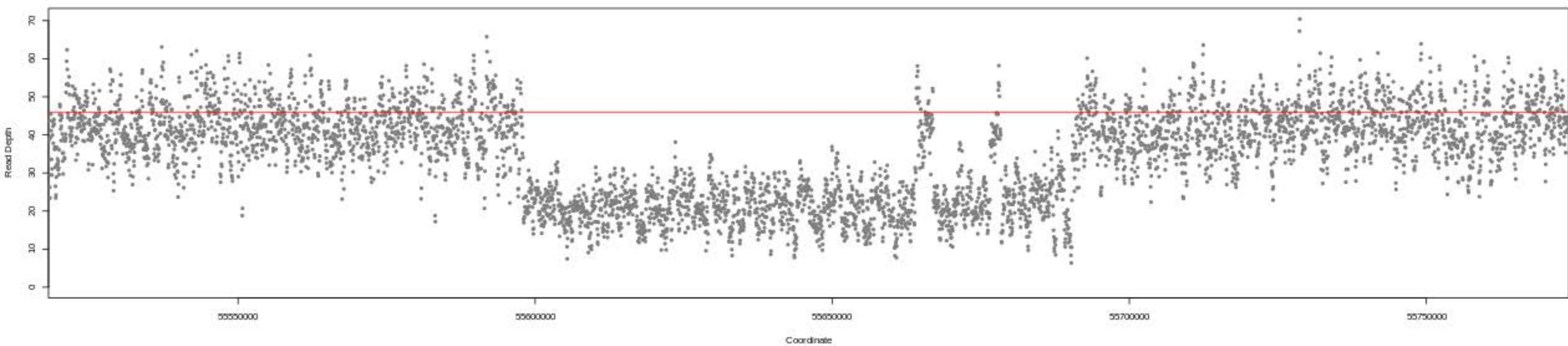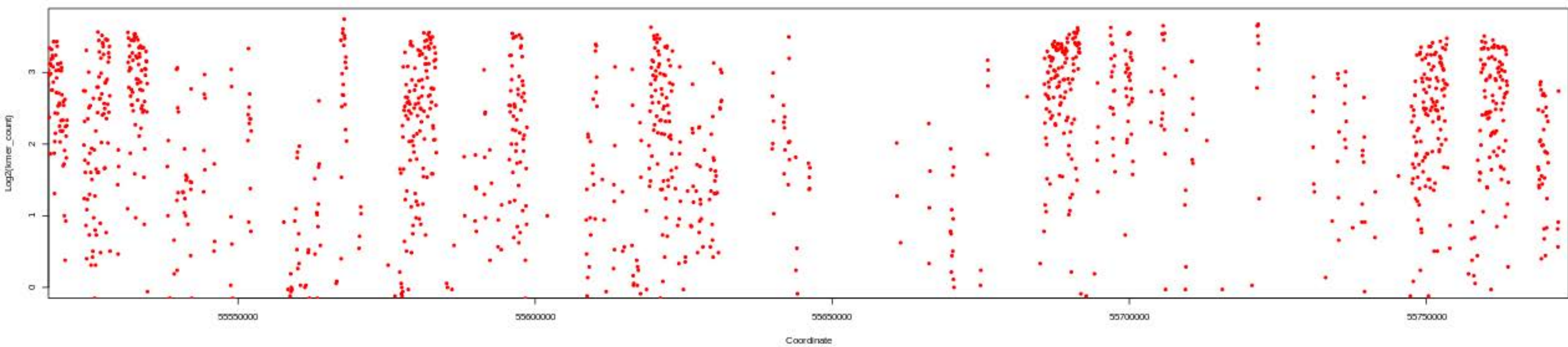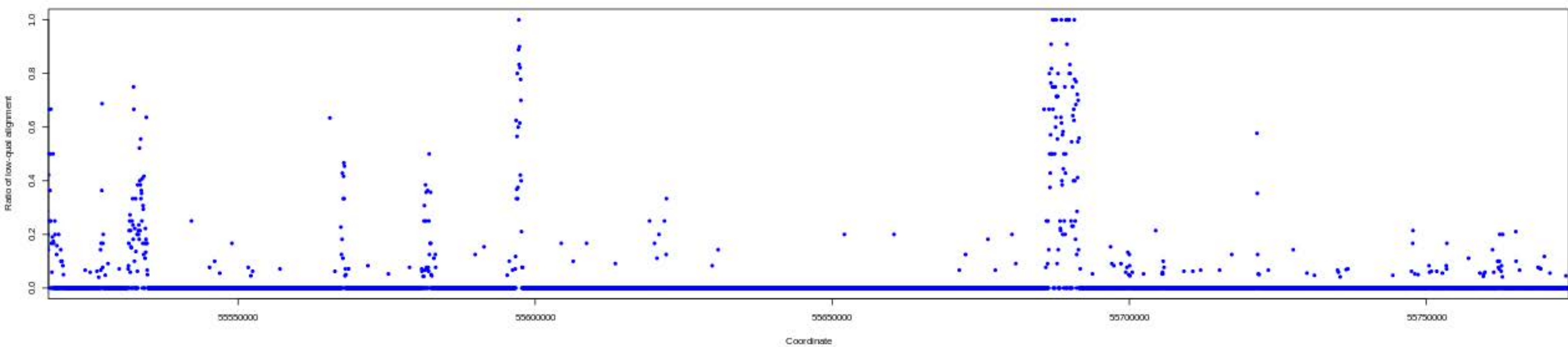

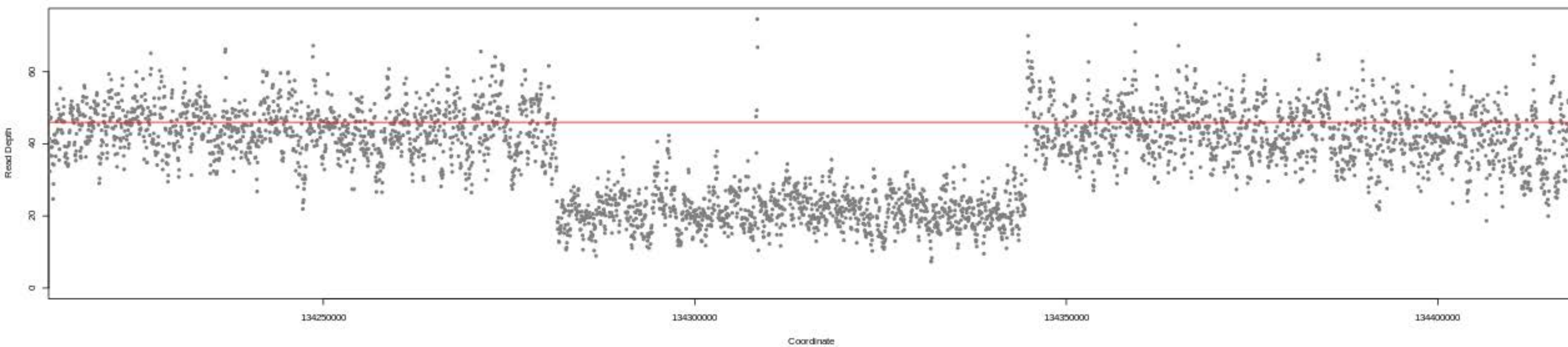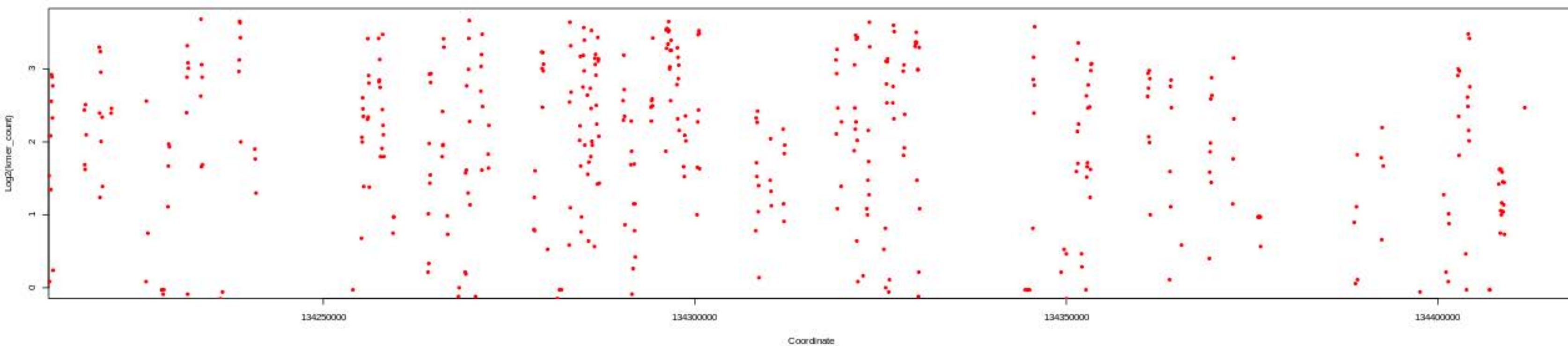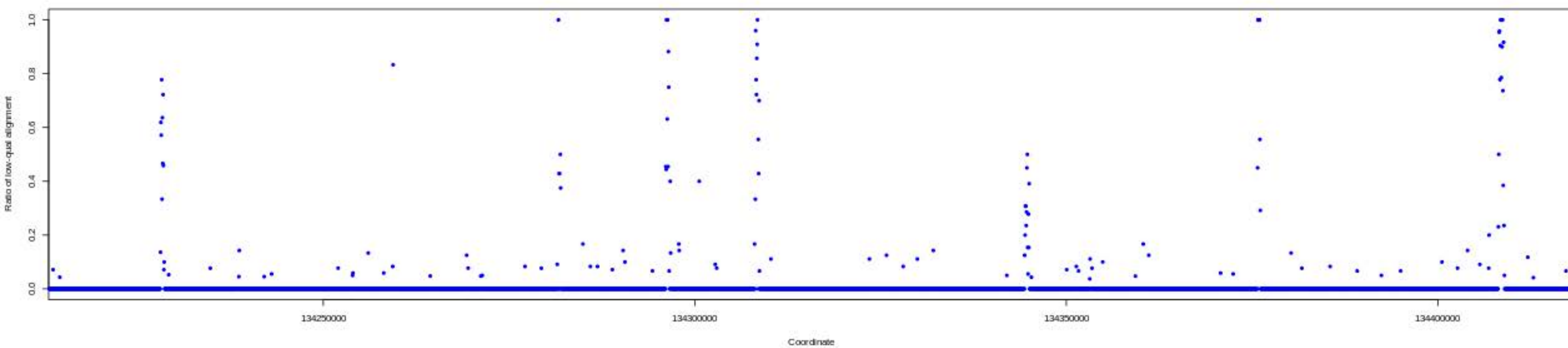

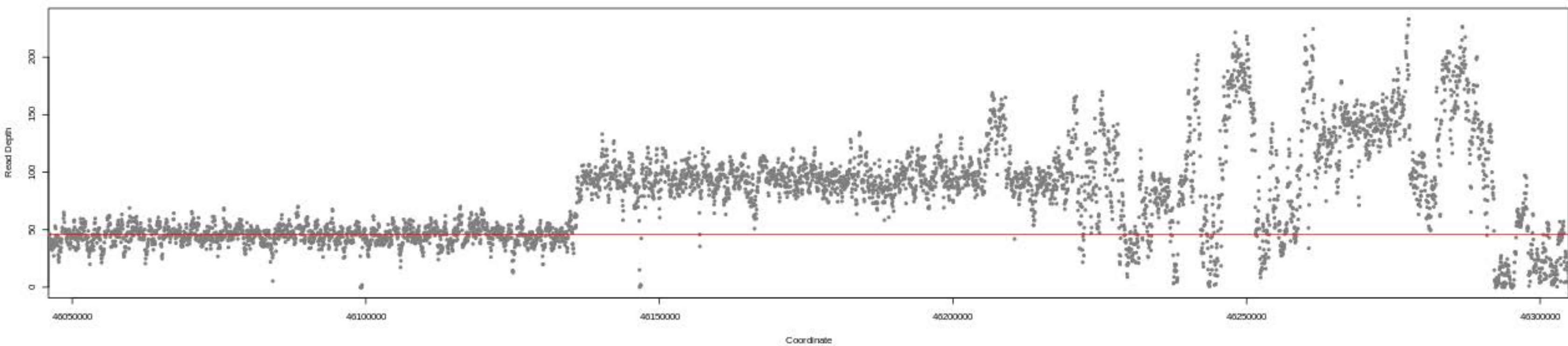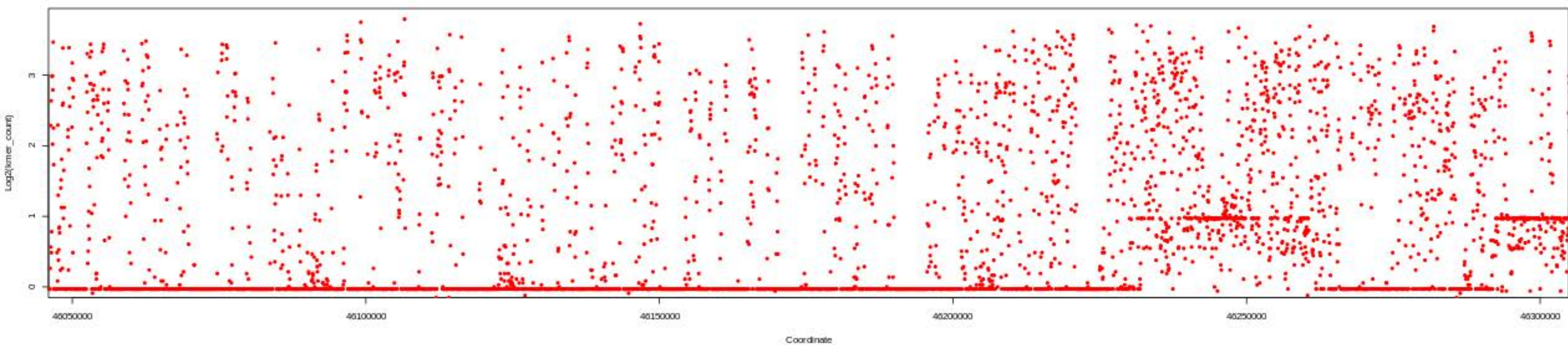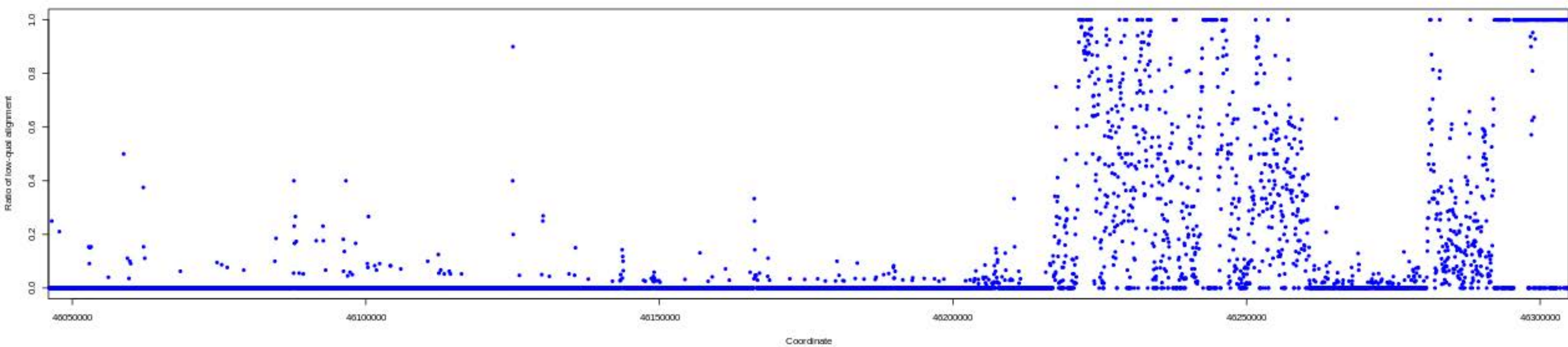

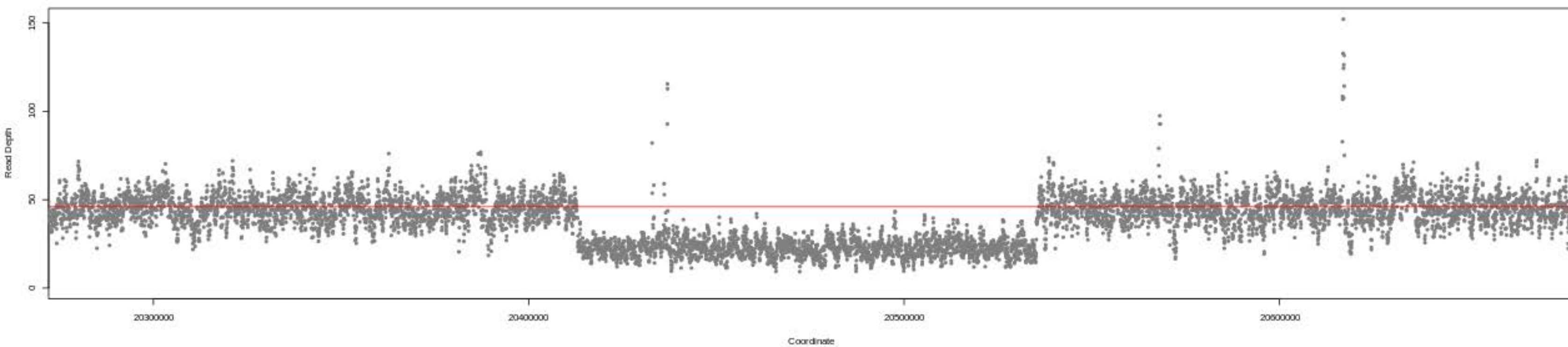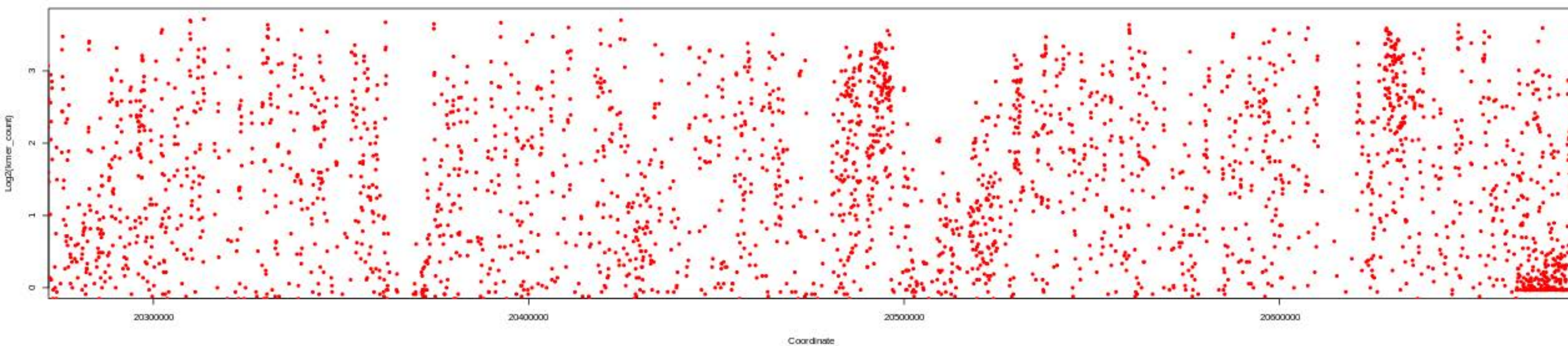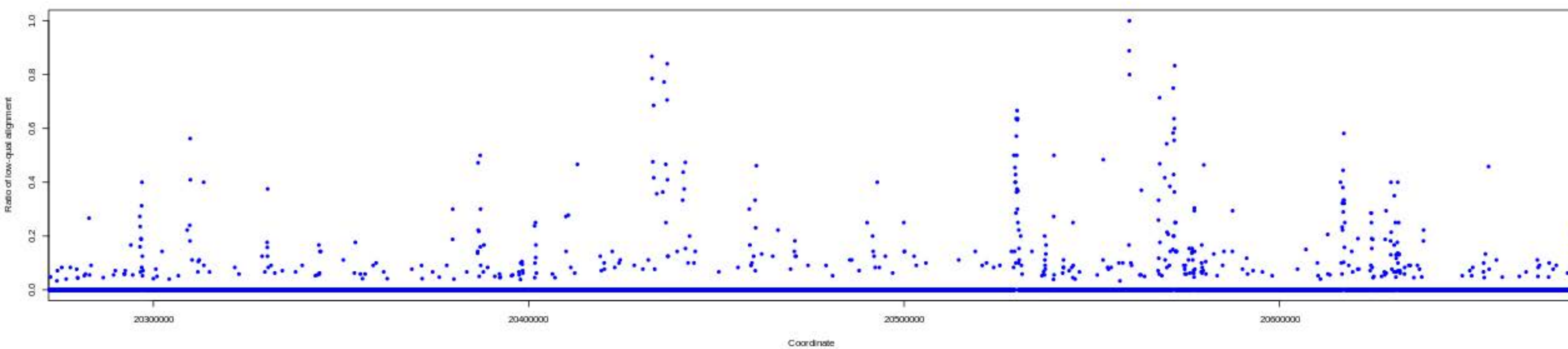

Supplement: Supplementary Figure S1 — Profiles of 112 CNVs in the truth set presented by Read Depth, log2(k-mer count) and Ratio of low-quality alignment, respectively Each CNV consists of three panels that are, from the top to bottom, (1) Read Depth colored gray, (2) log2(k-mer_count) colored red, and (3) Ratio of low-quality alignment colored blue. [file mmc2.pdf]

**Figure S2.**

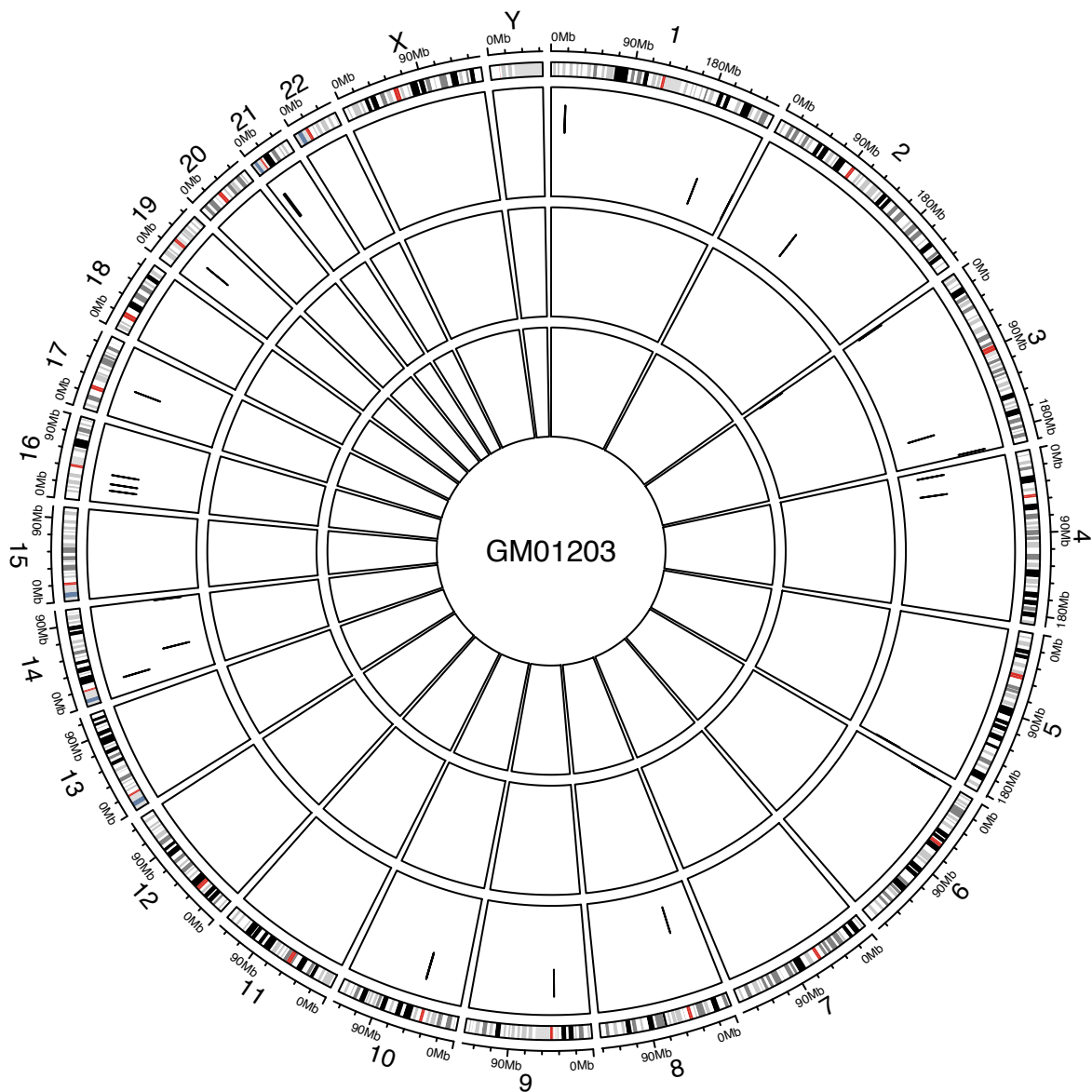

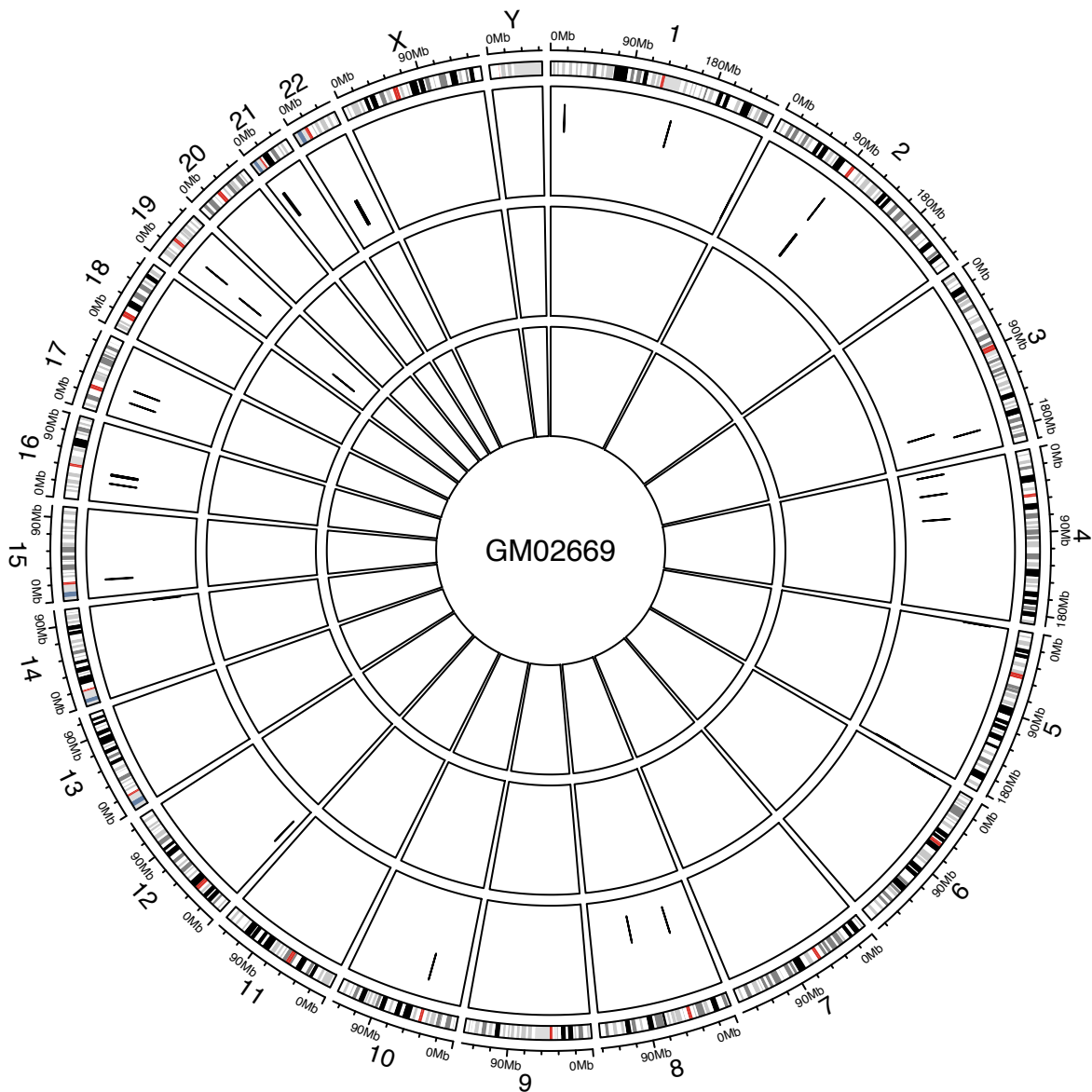

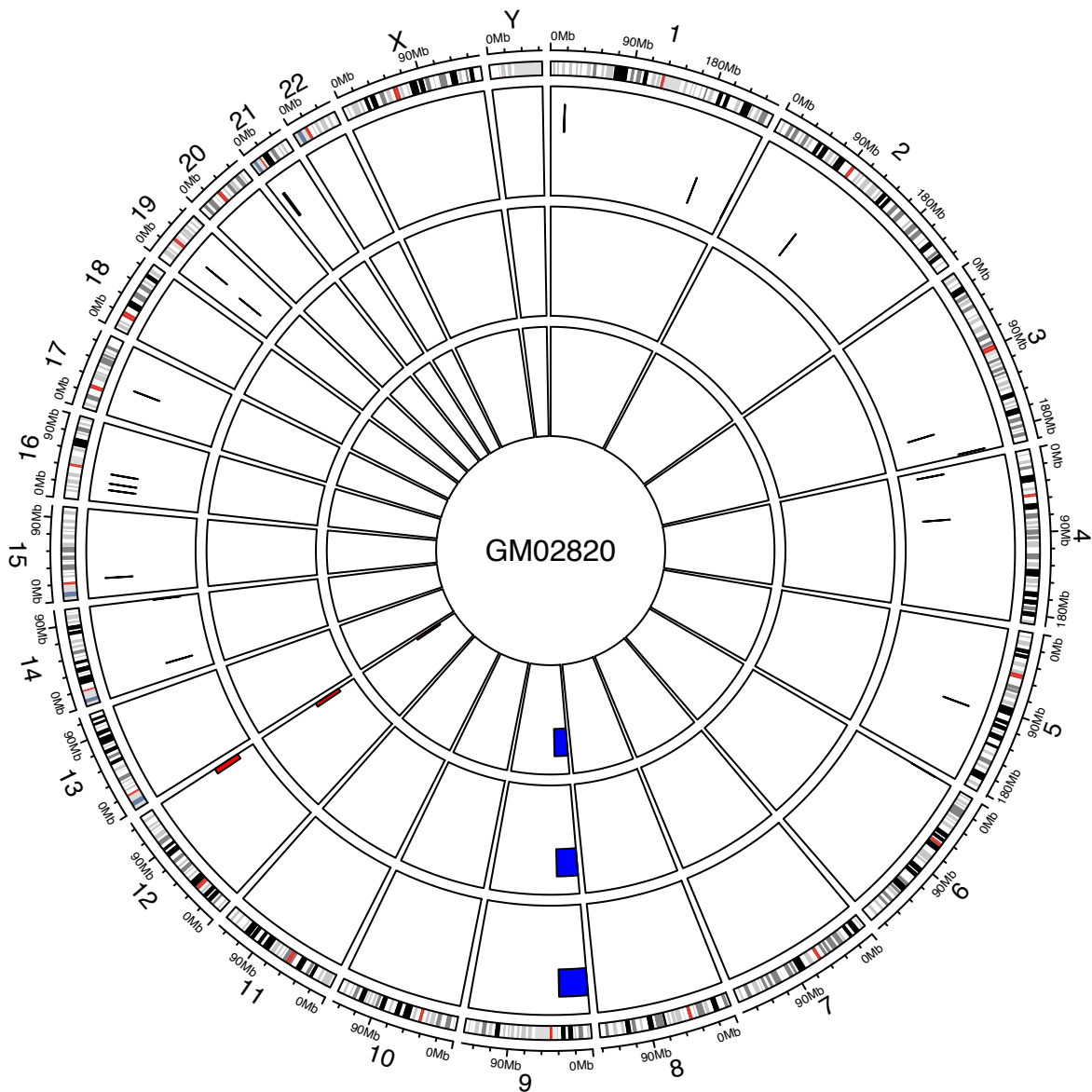

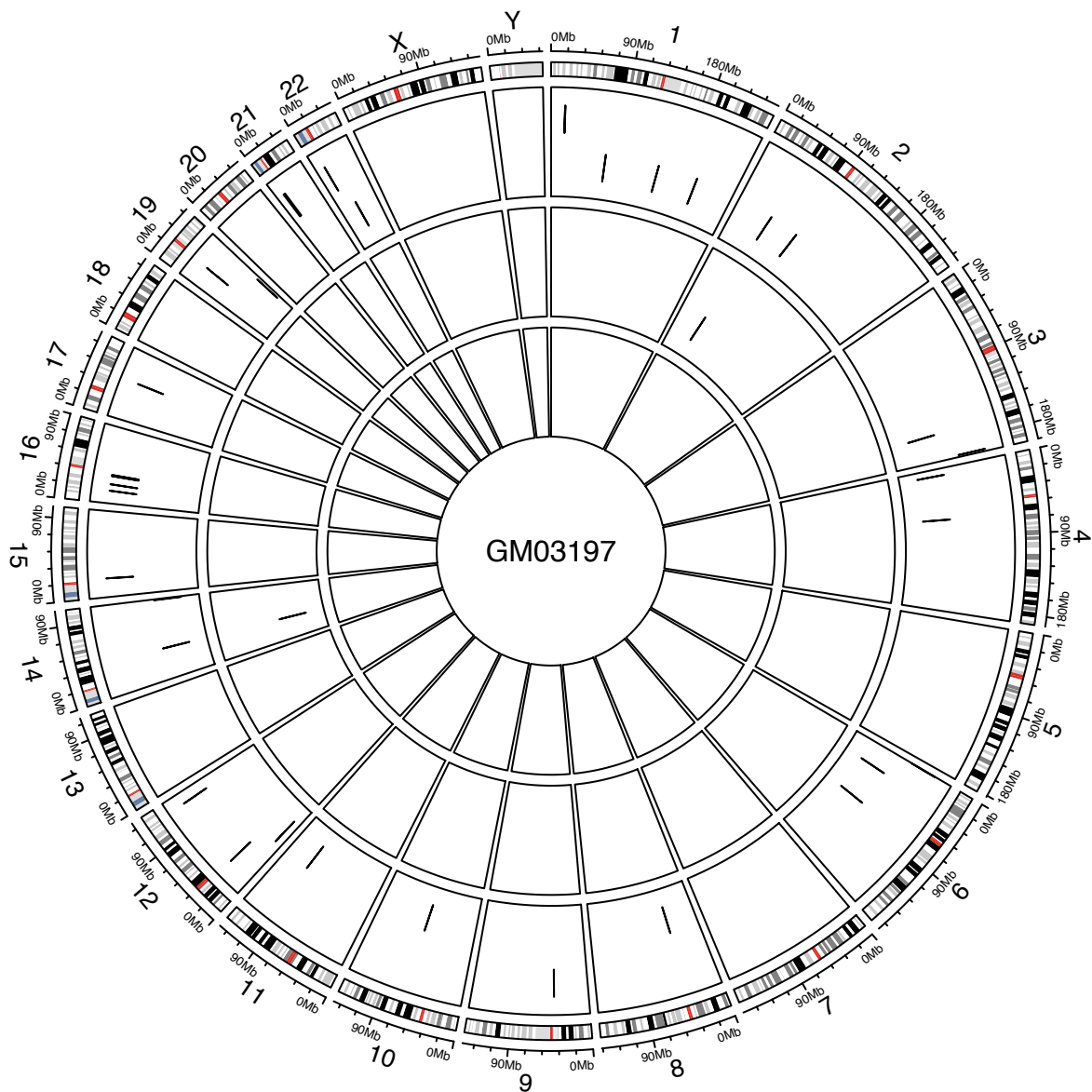

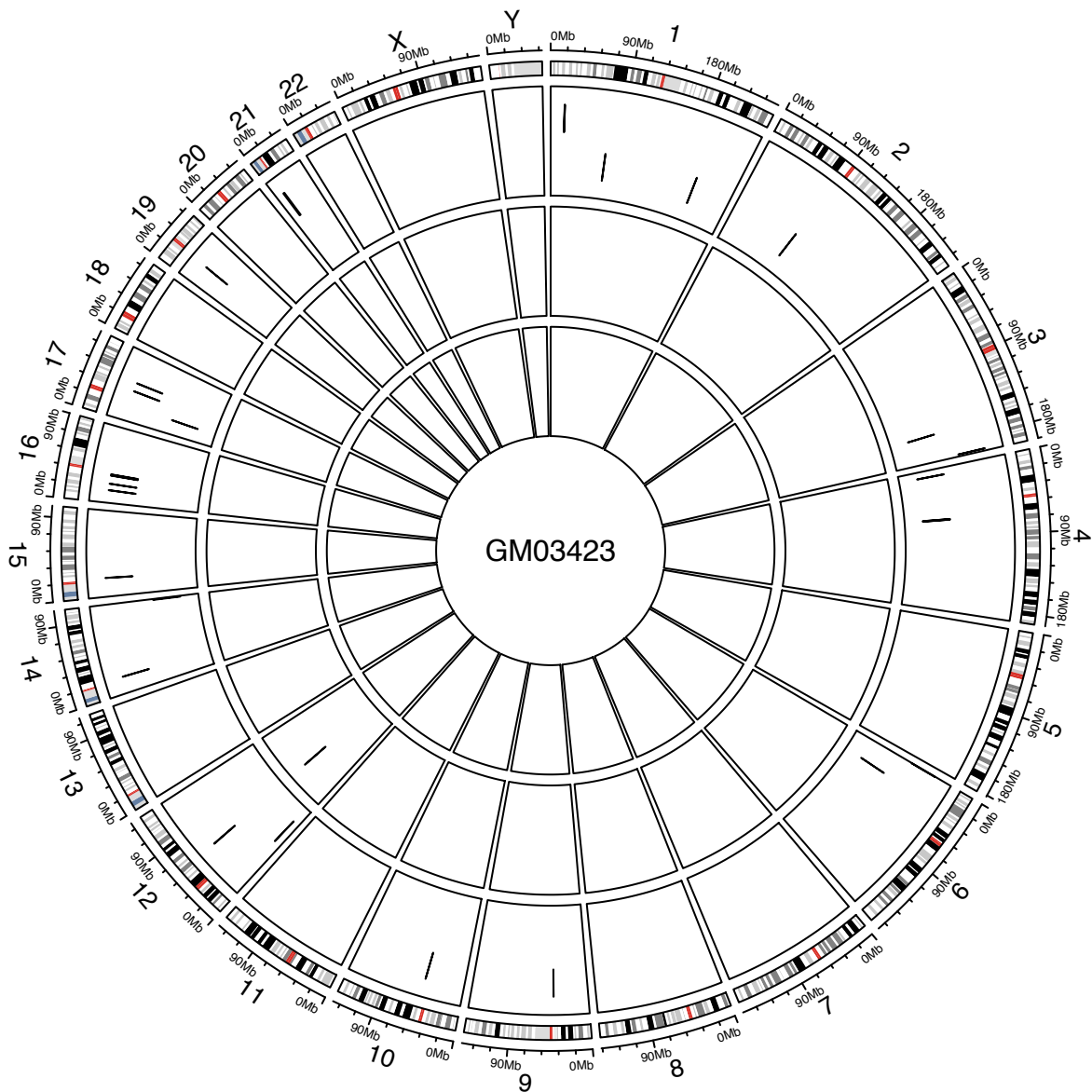

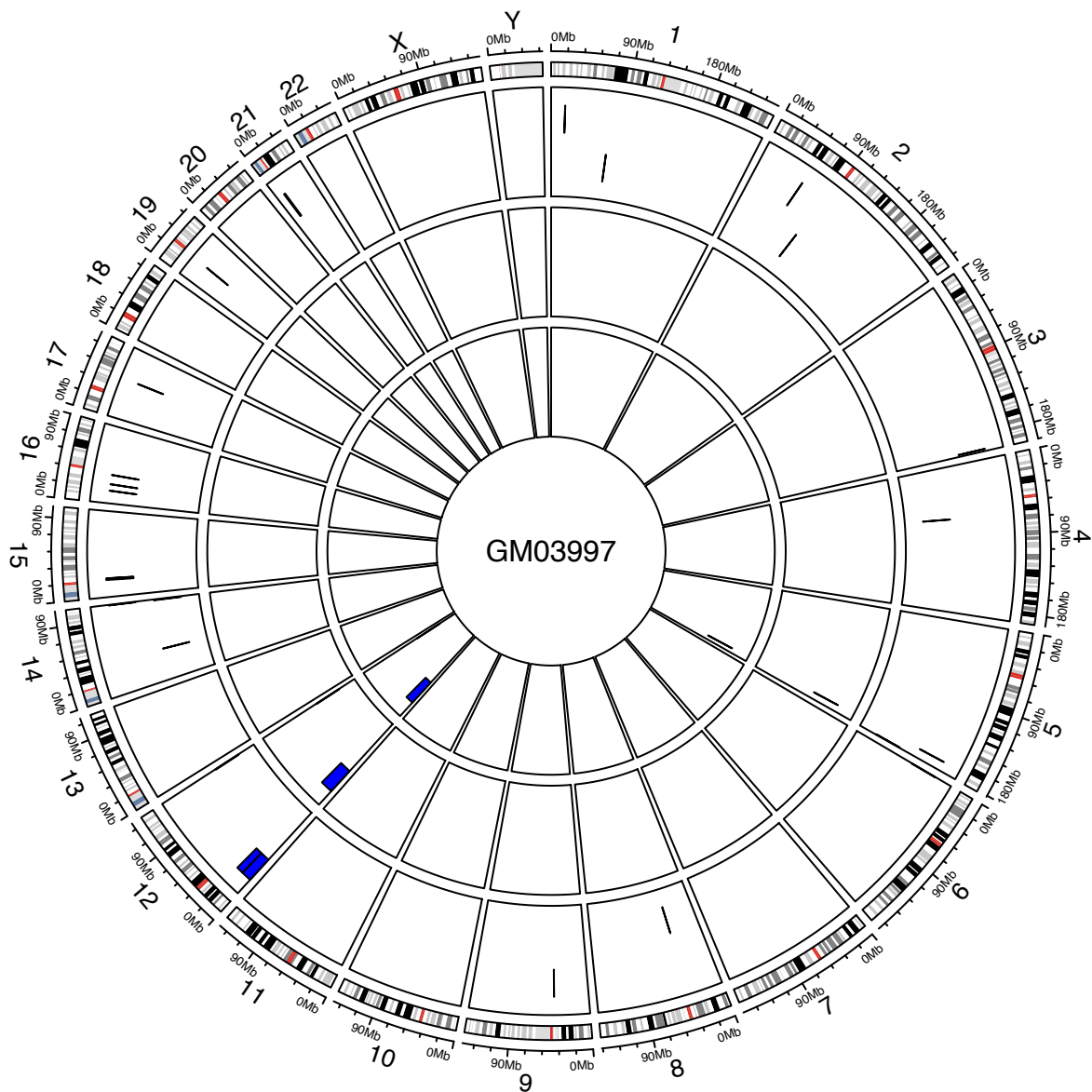

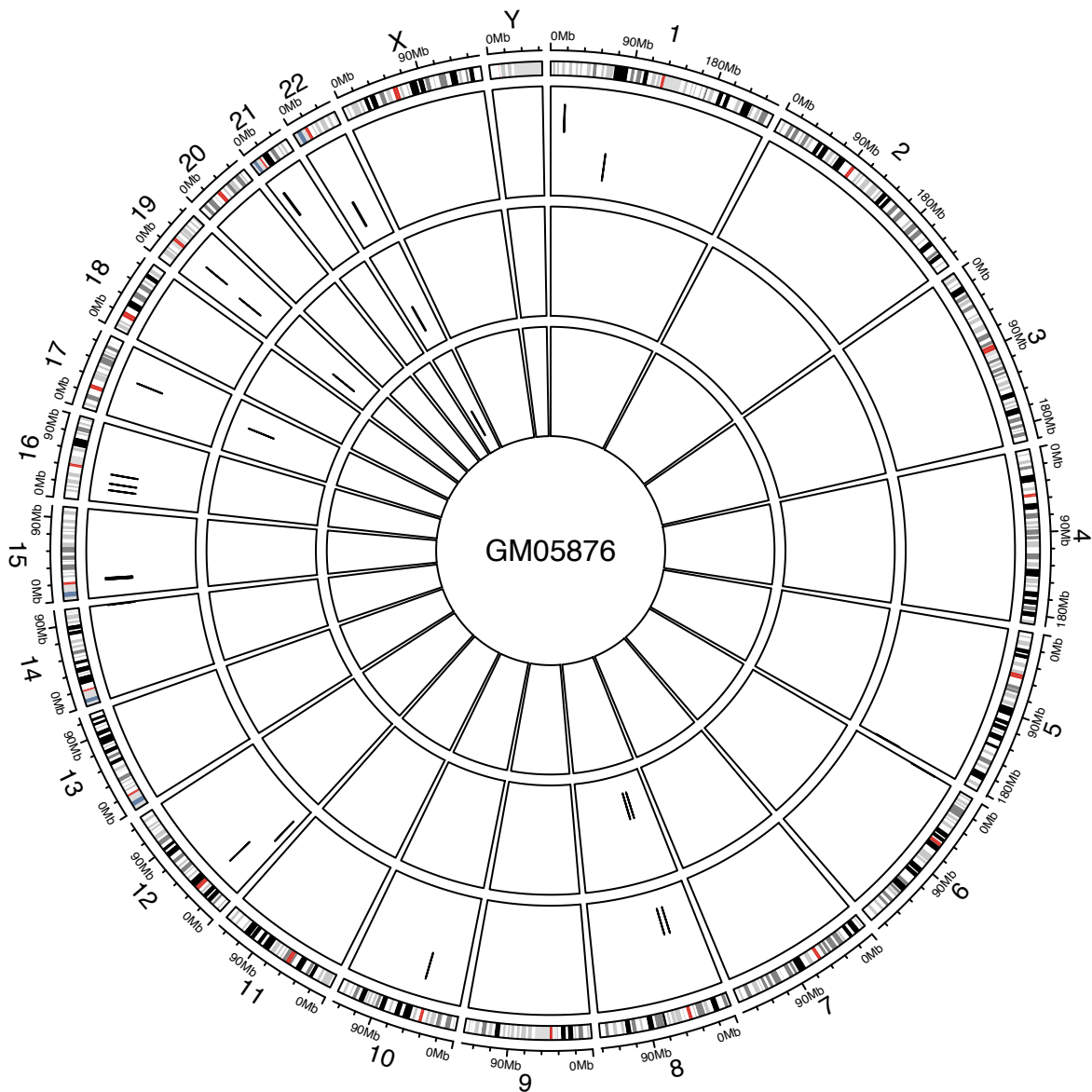

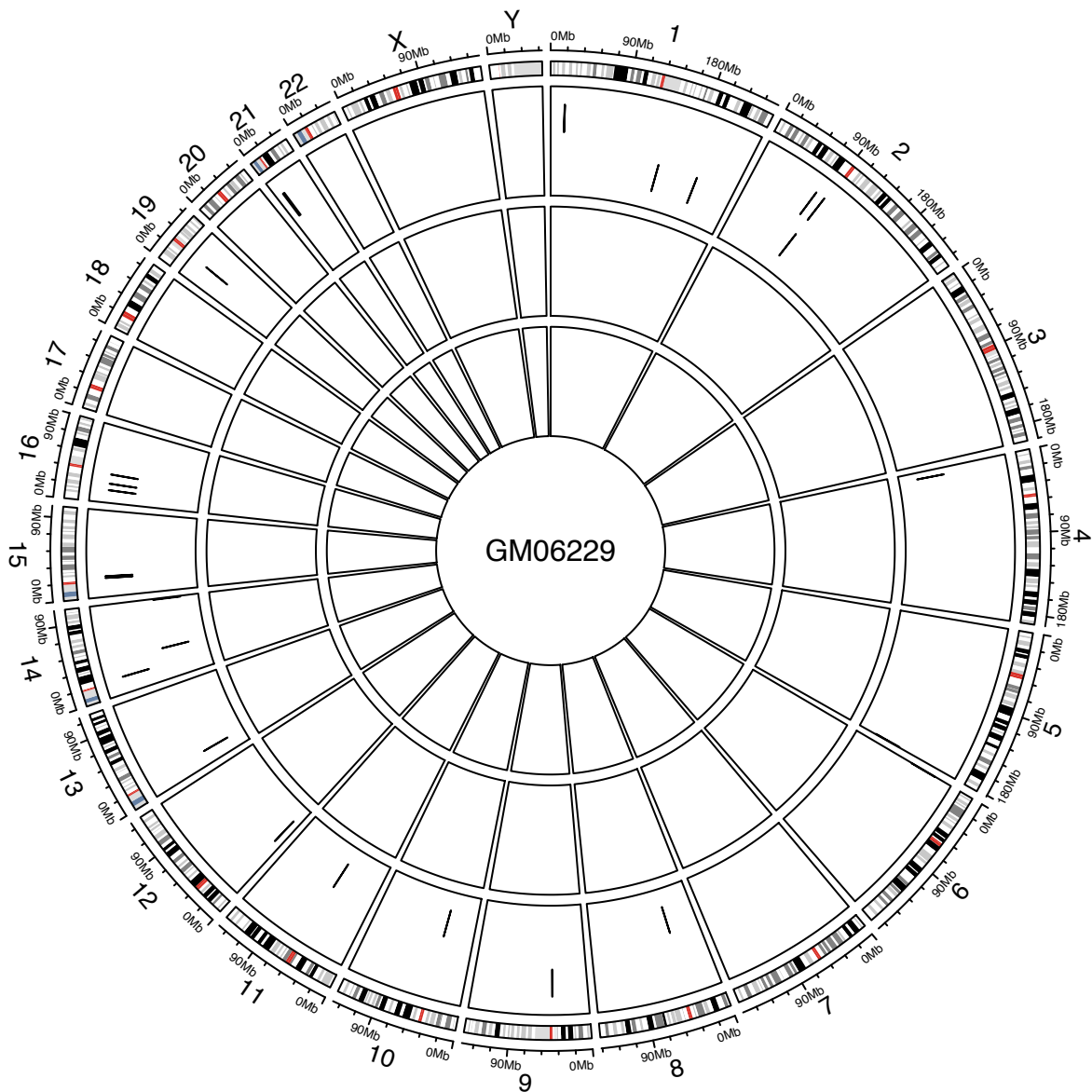

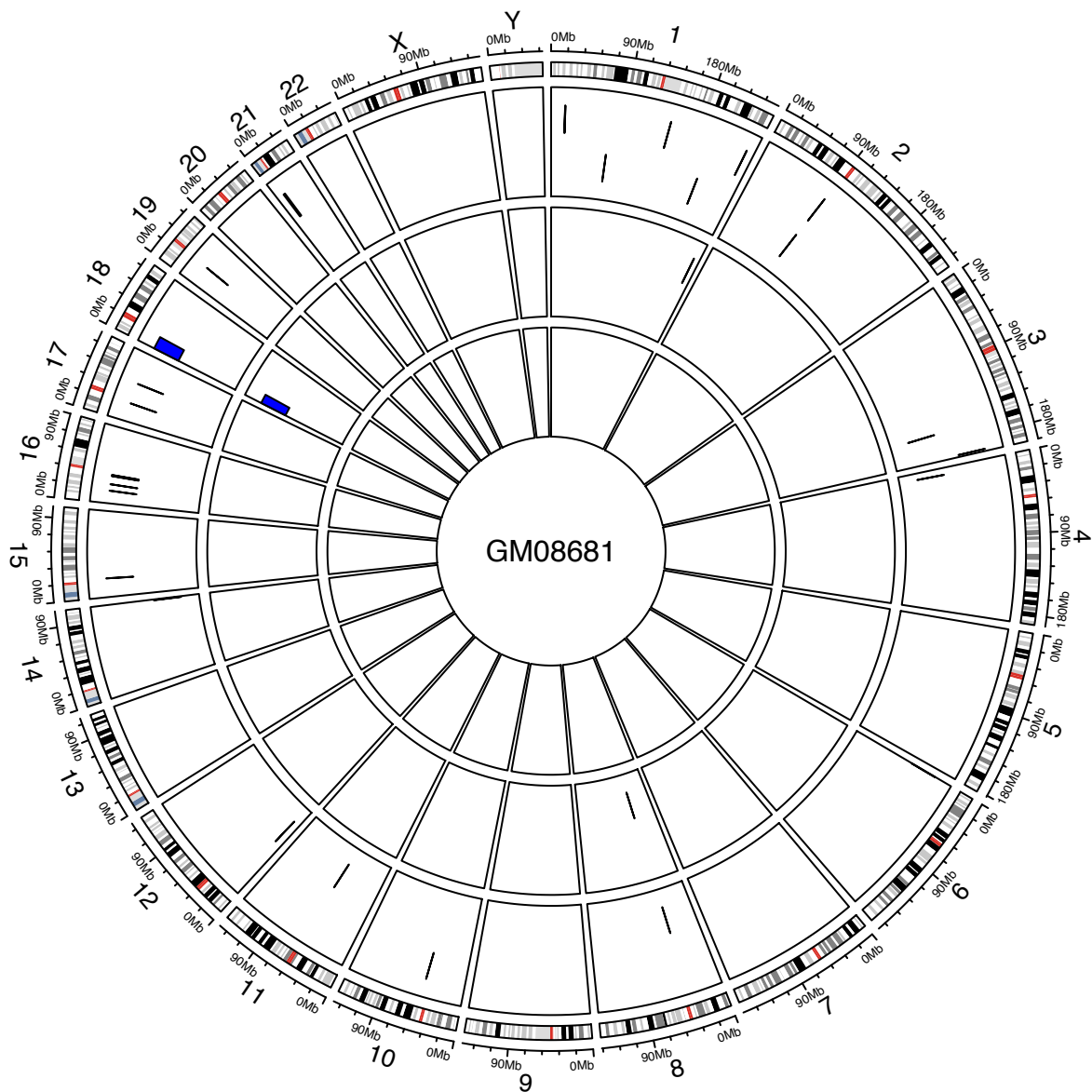

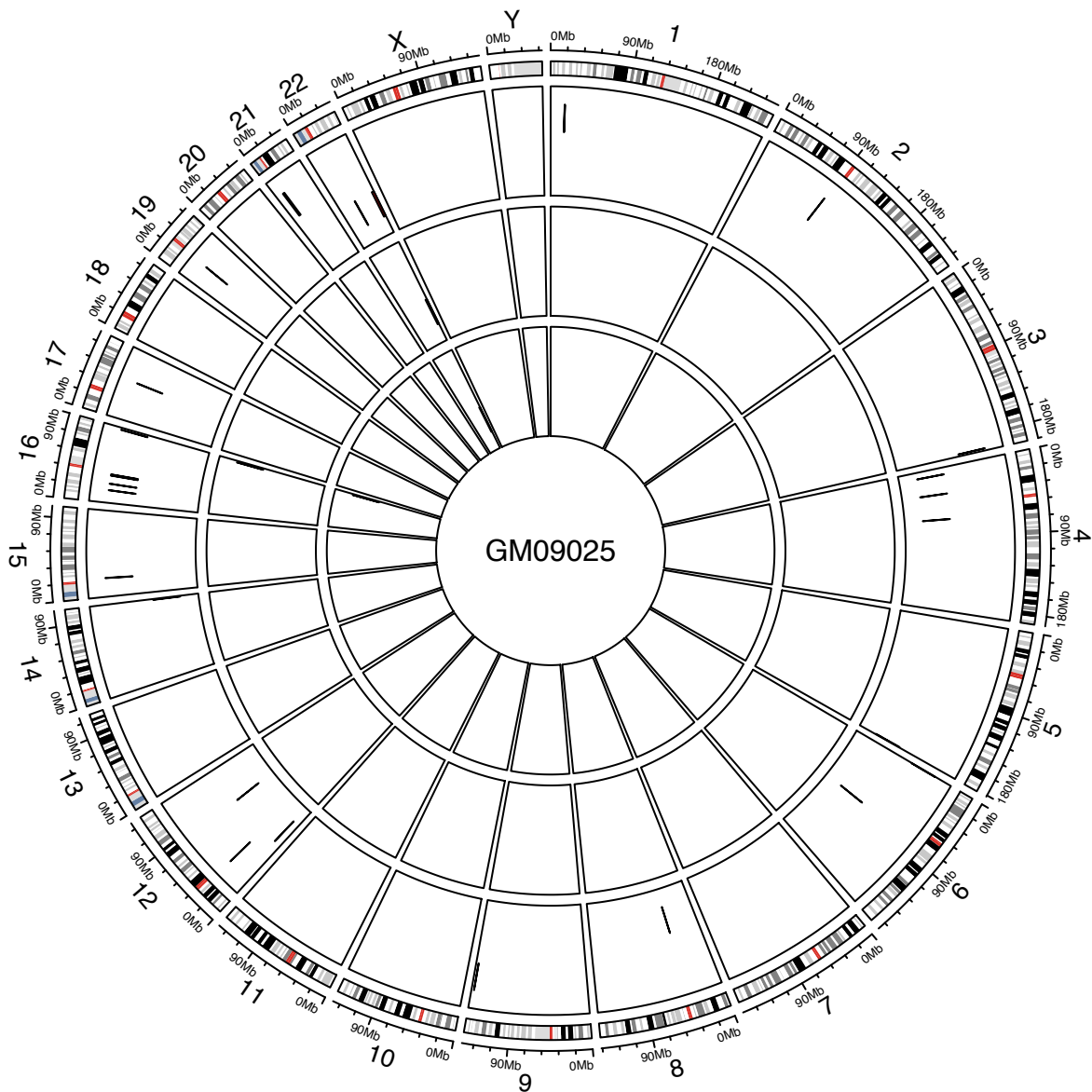

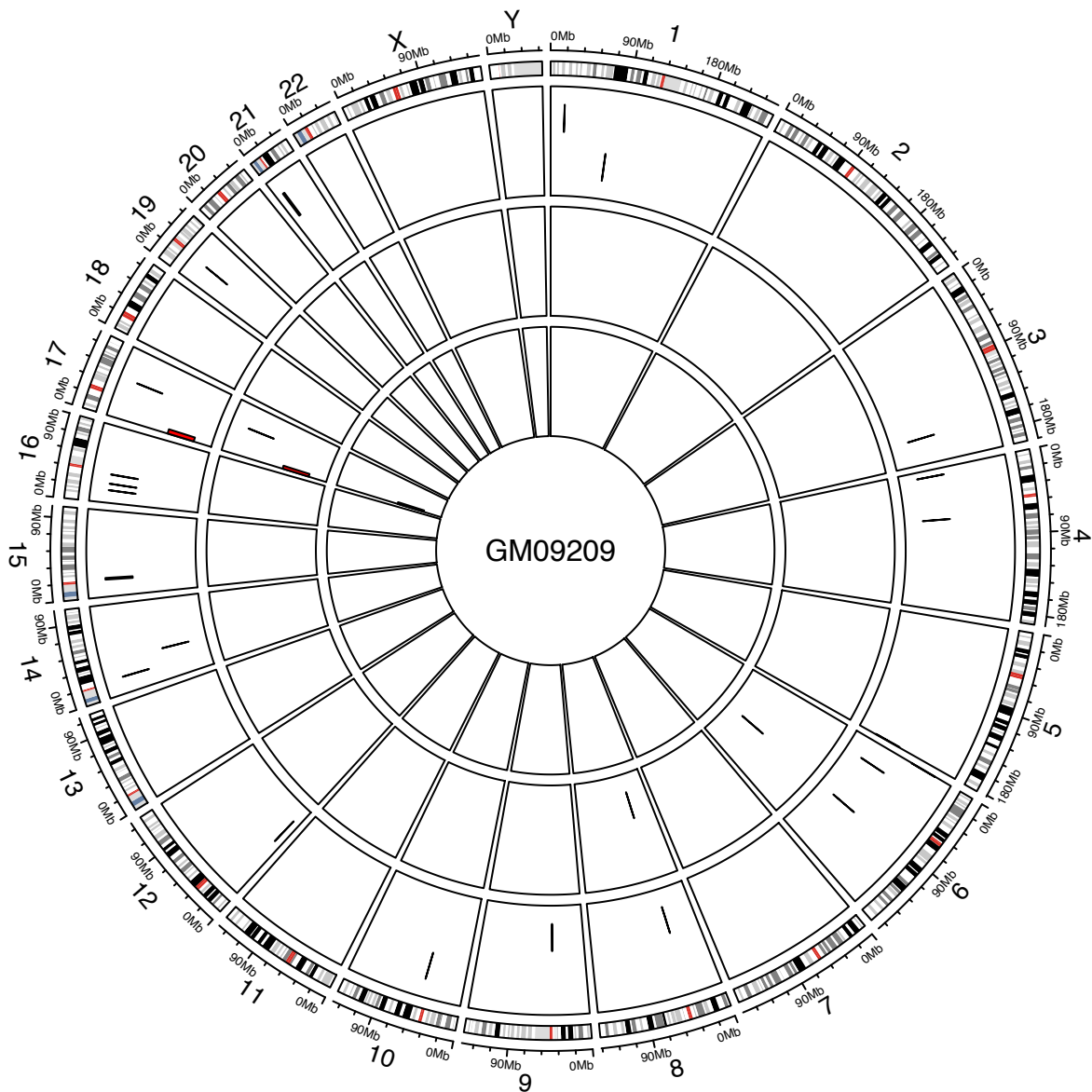

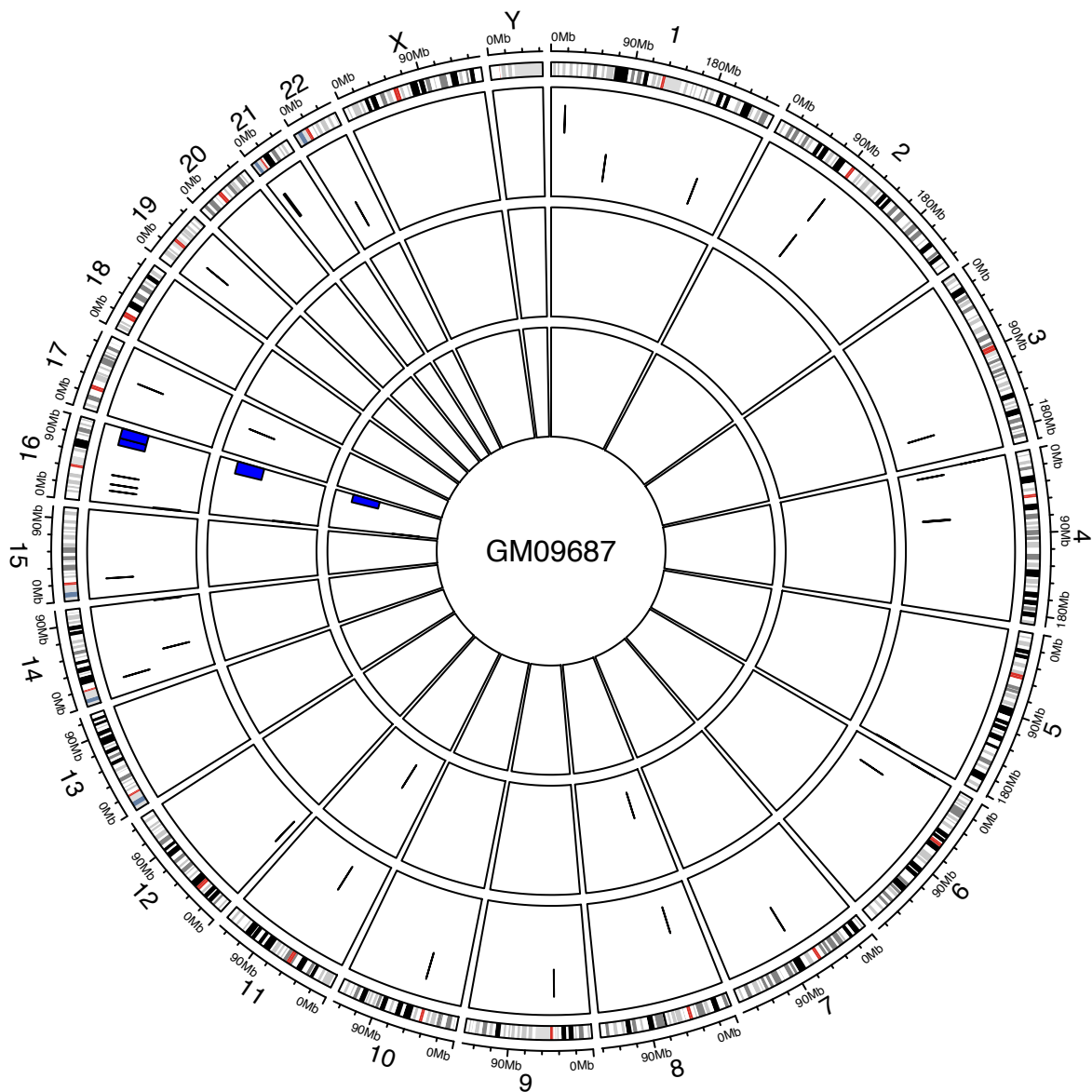

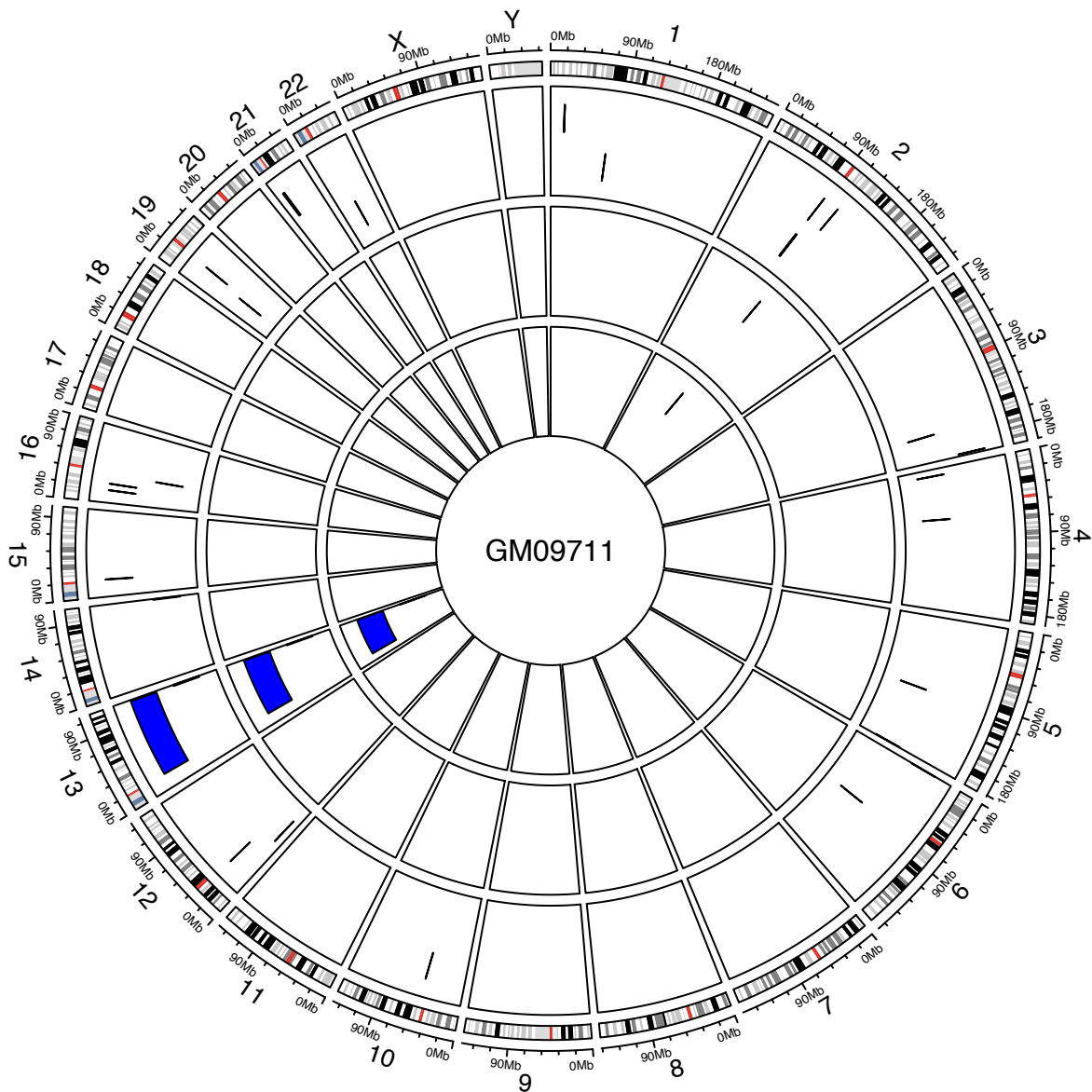

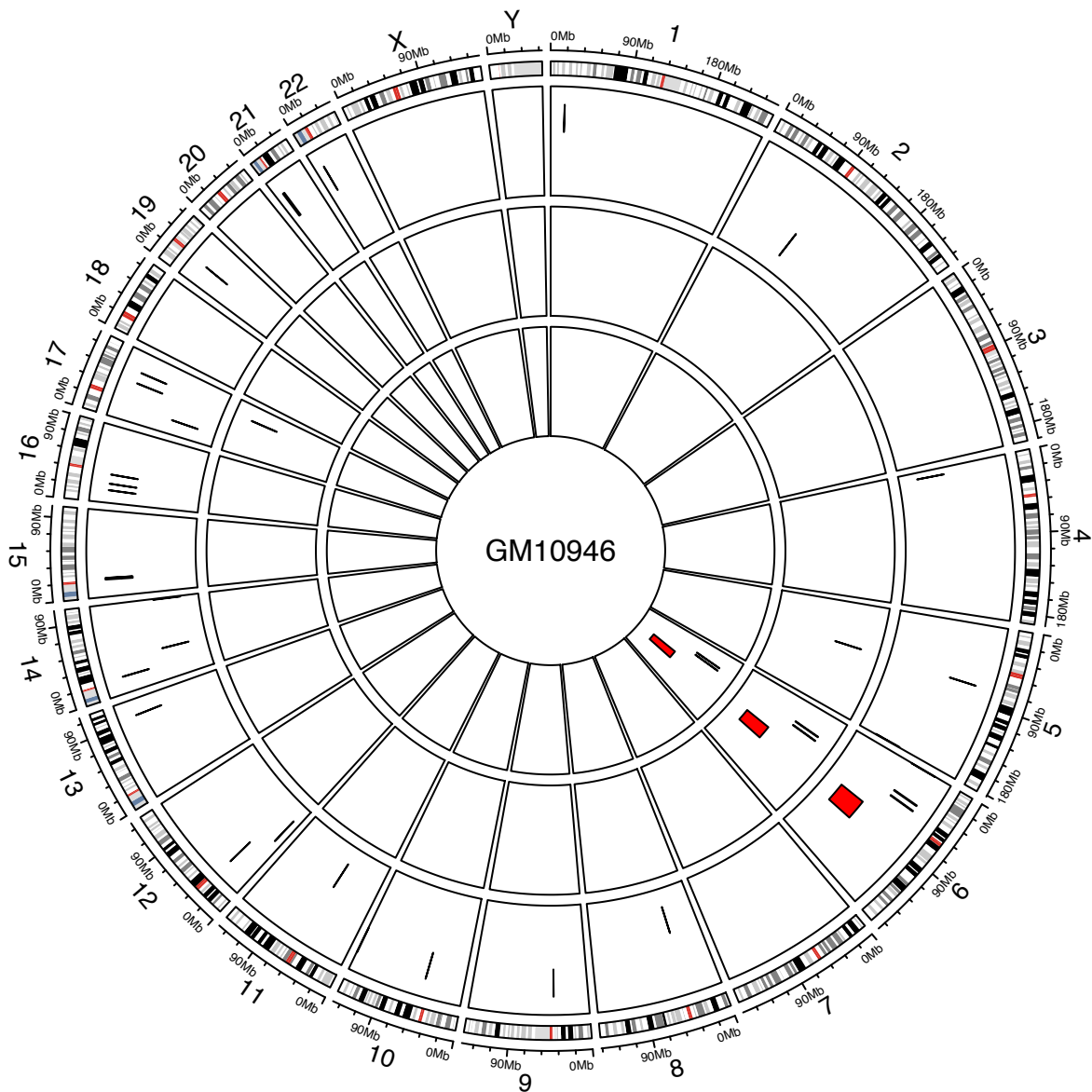

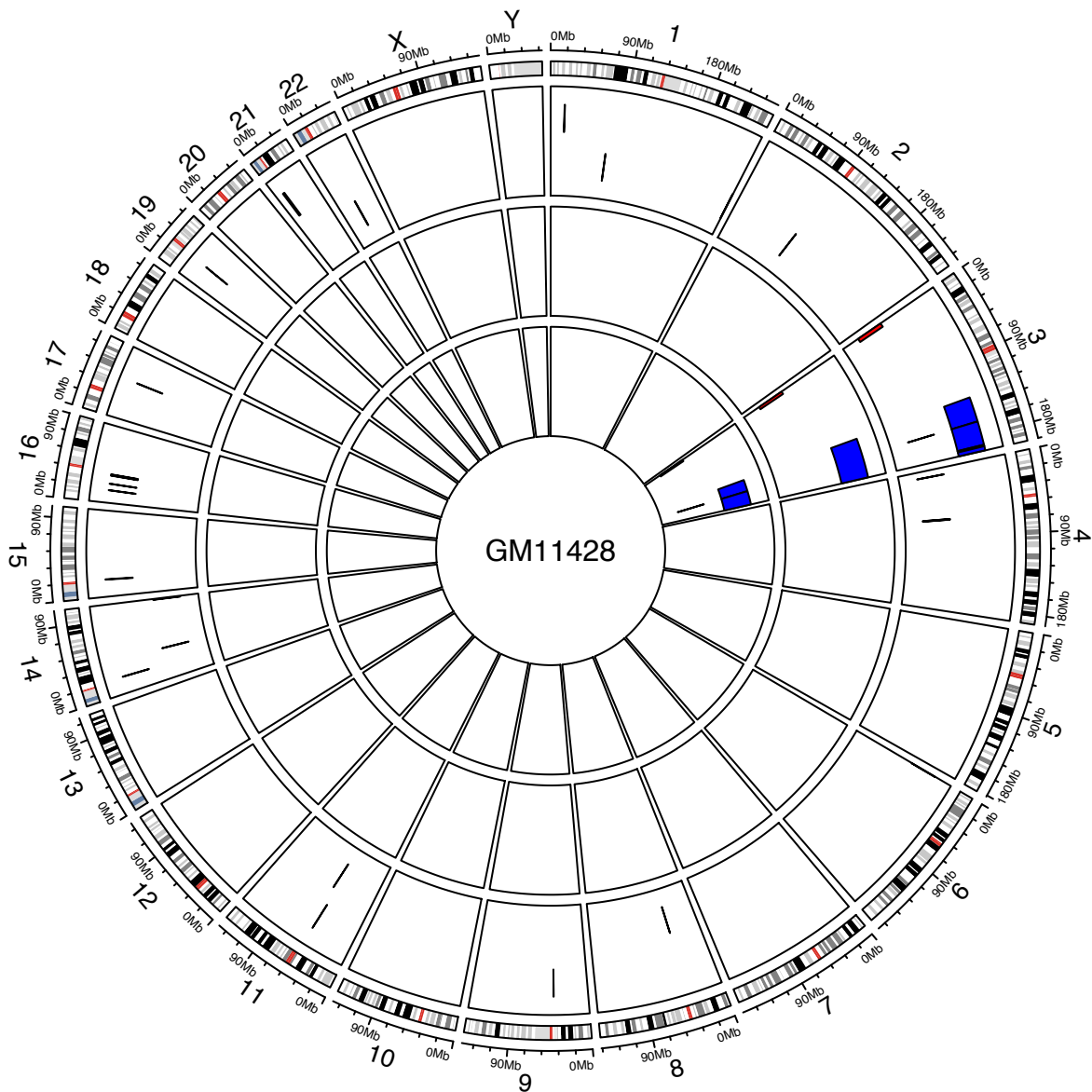

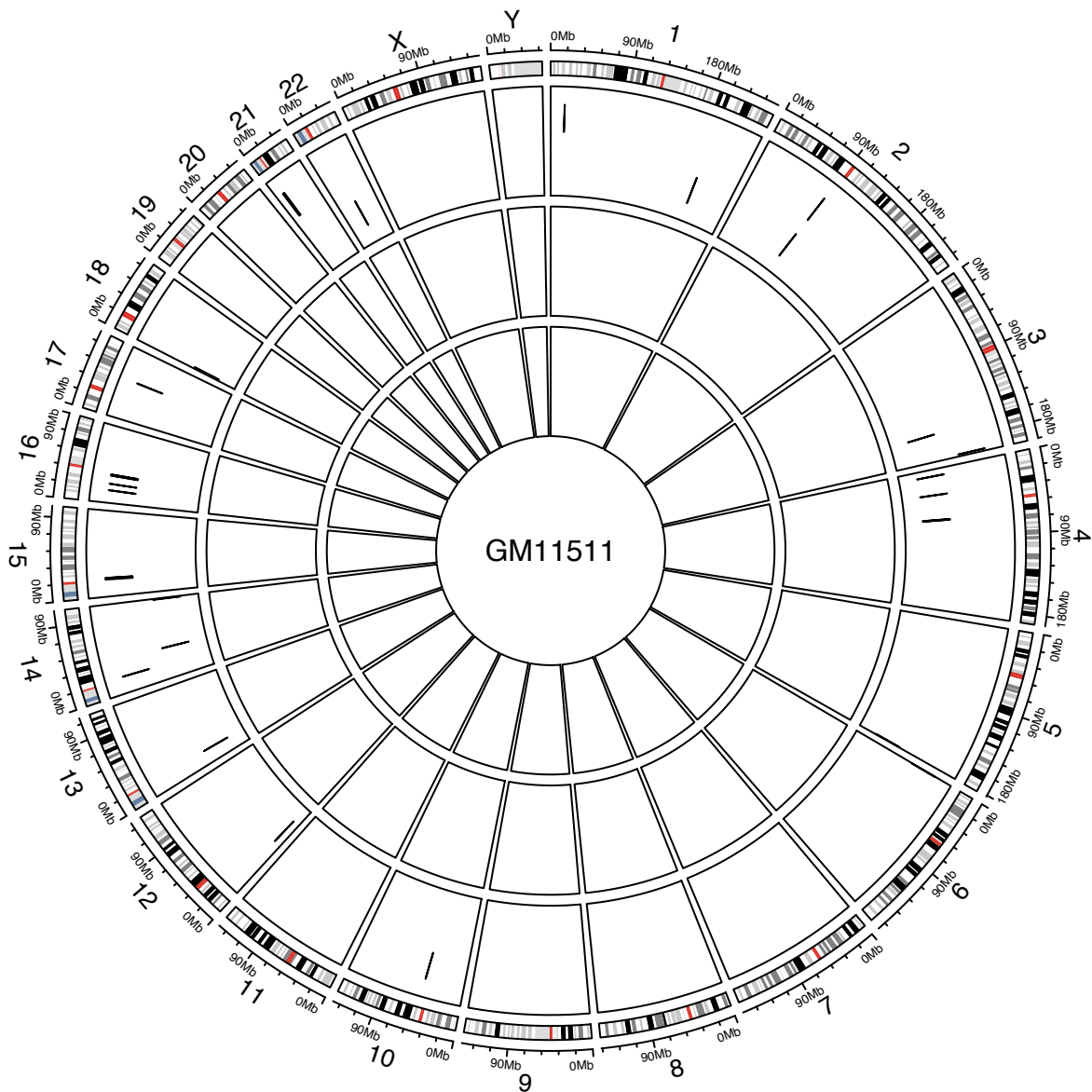

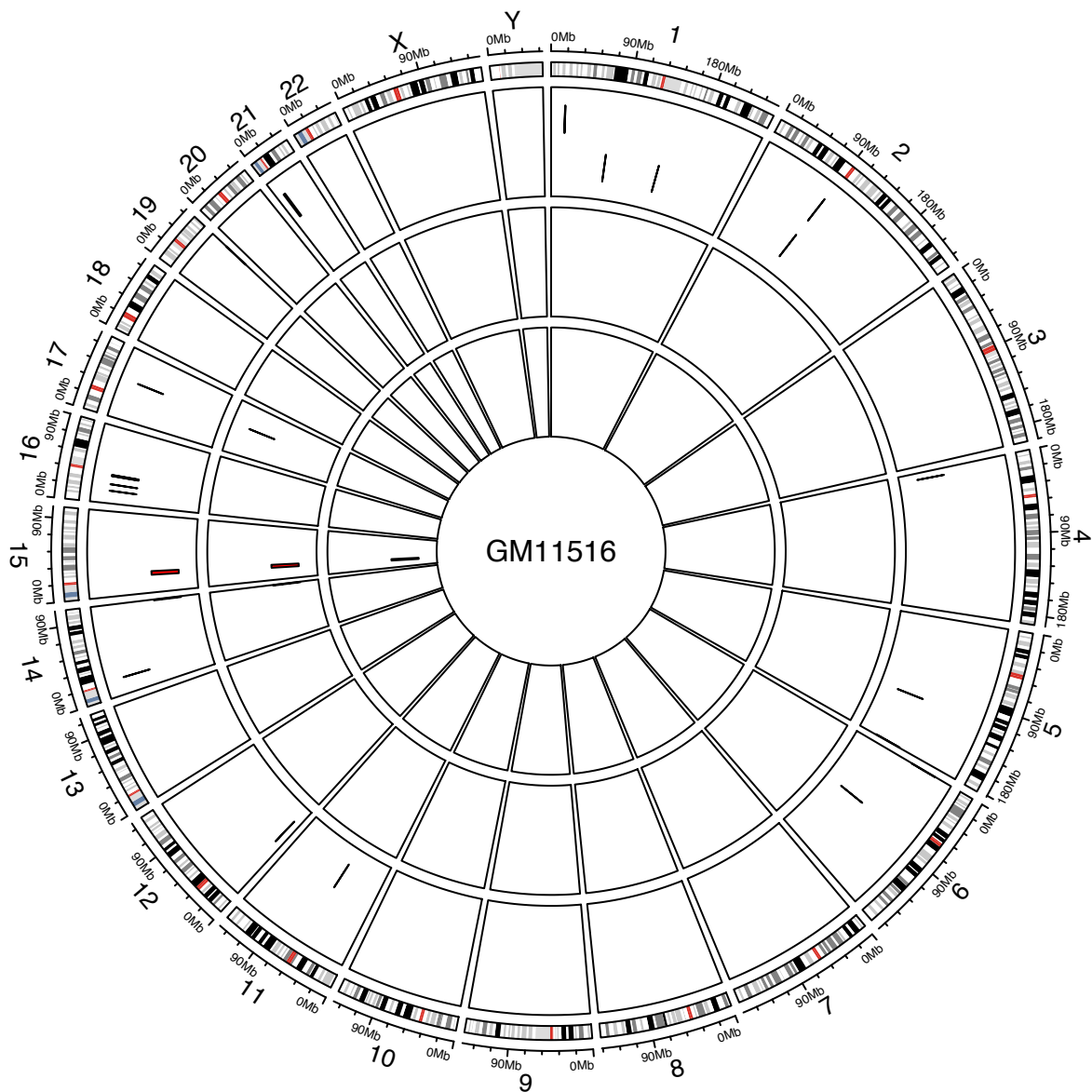

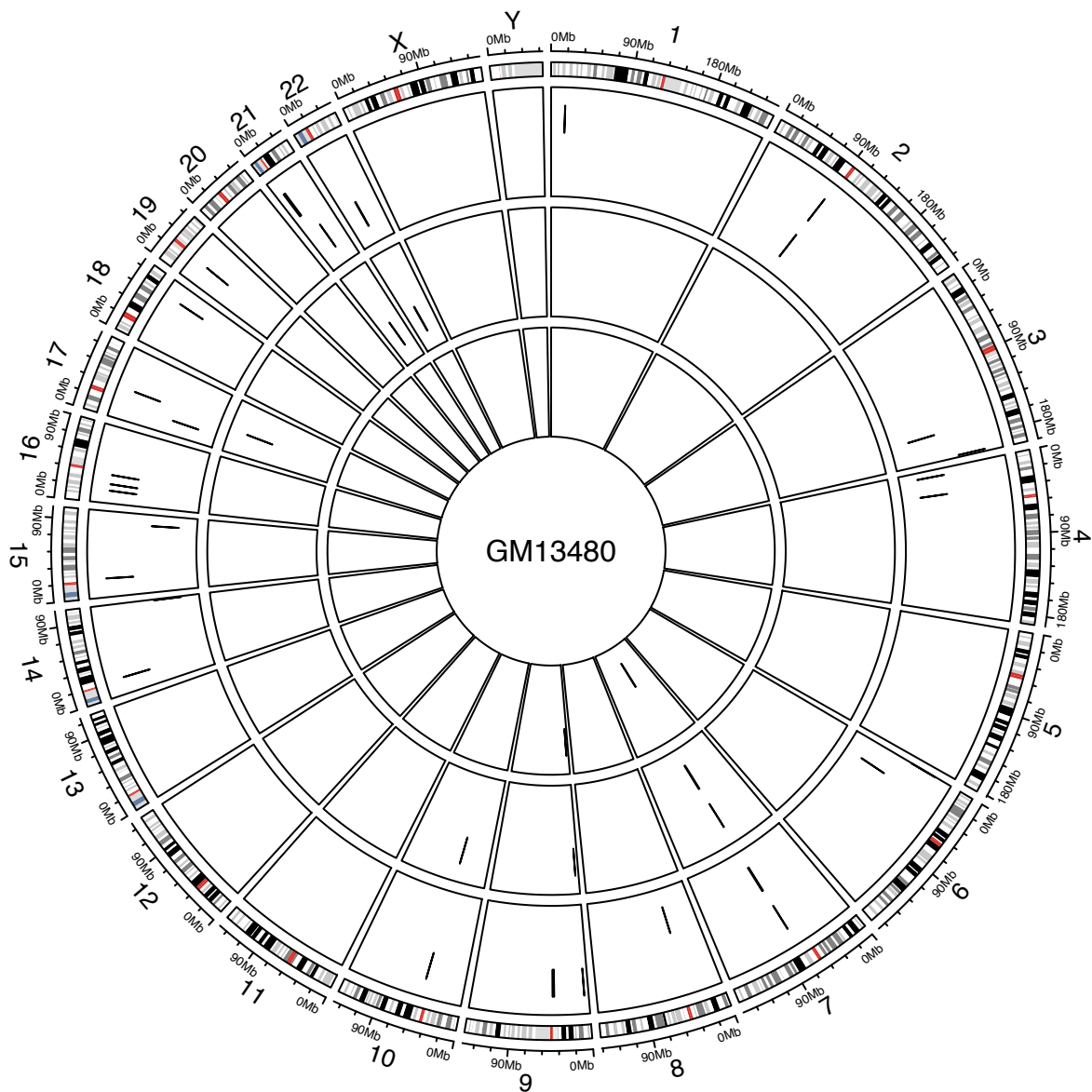

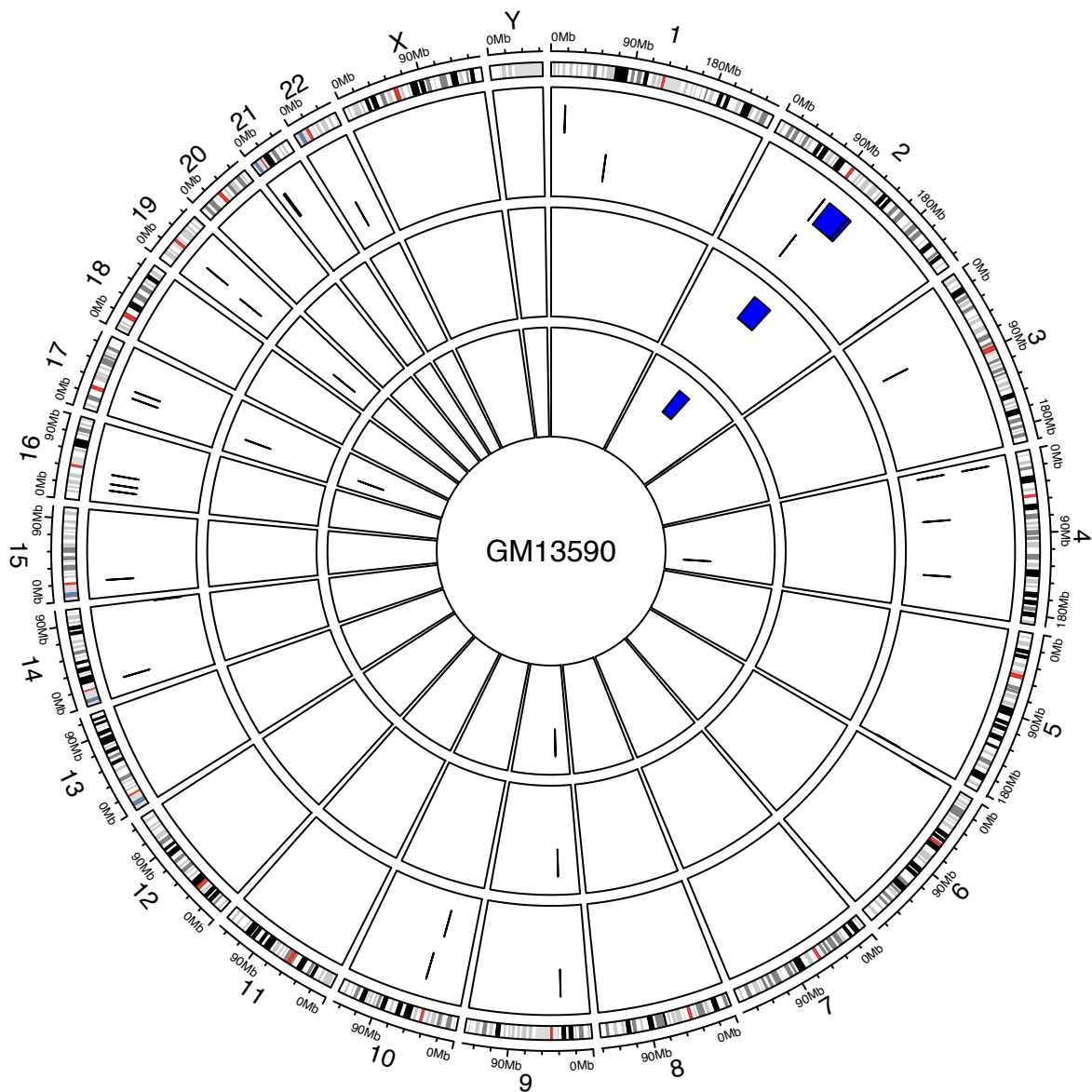

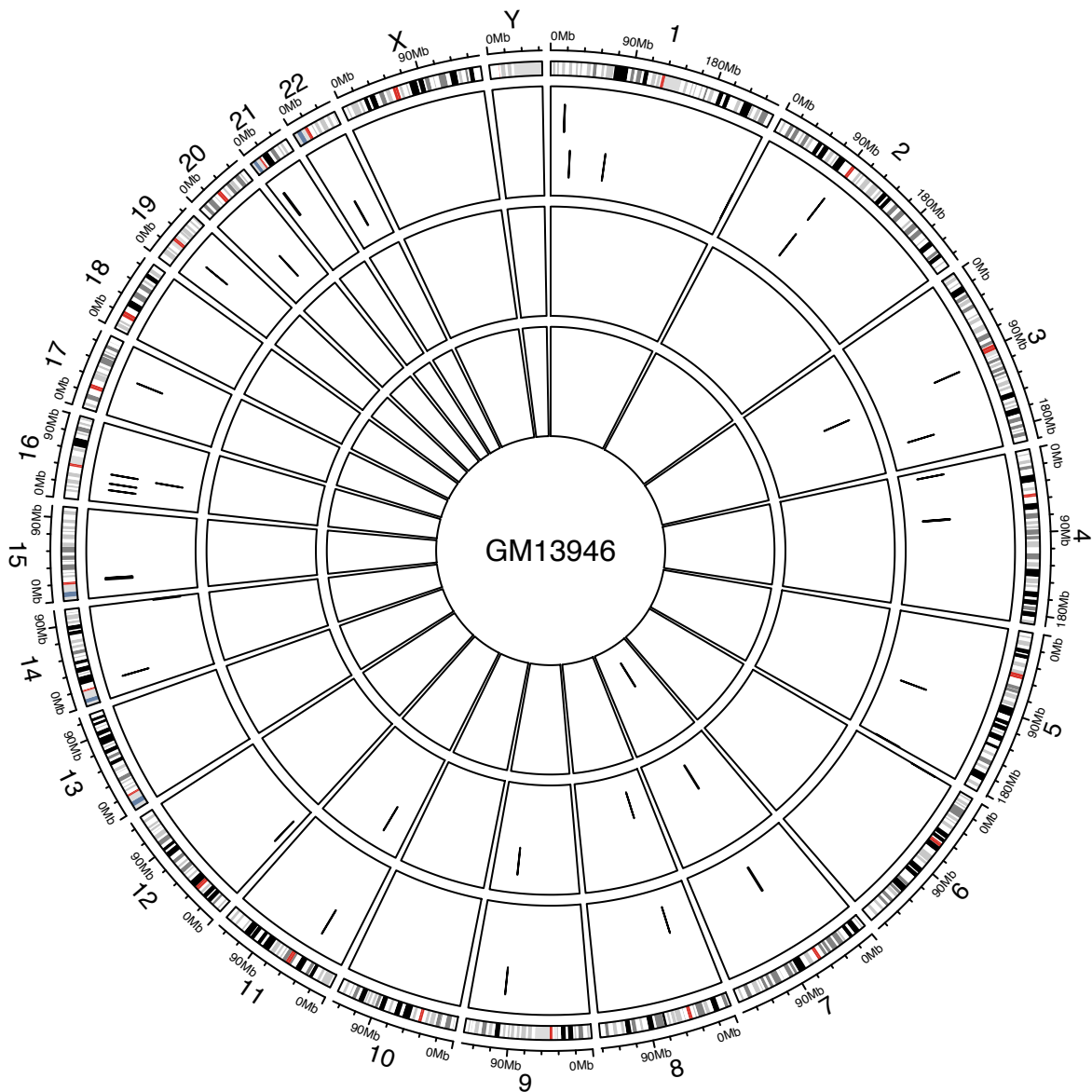

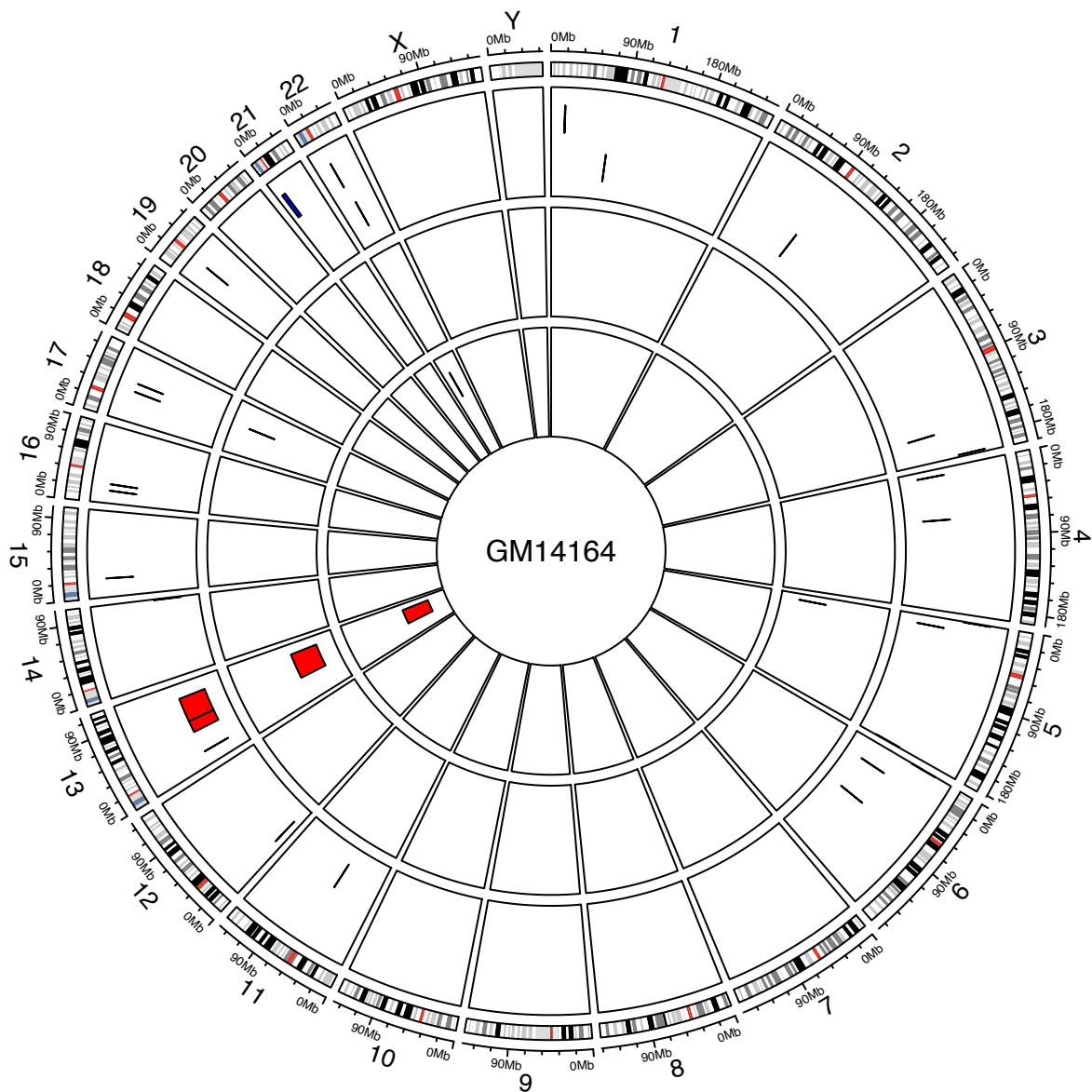

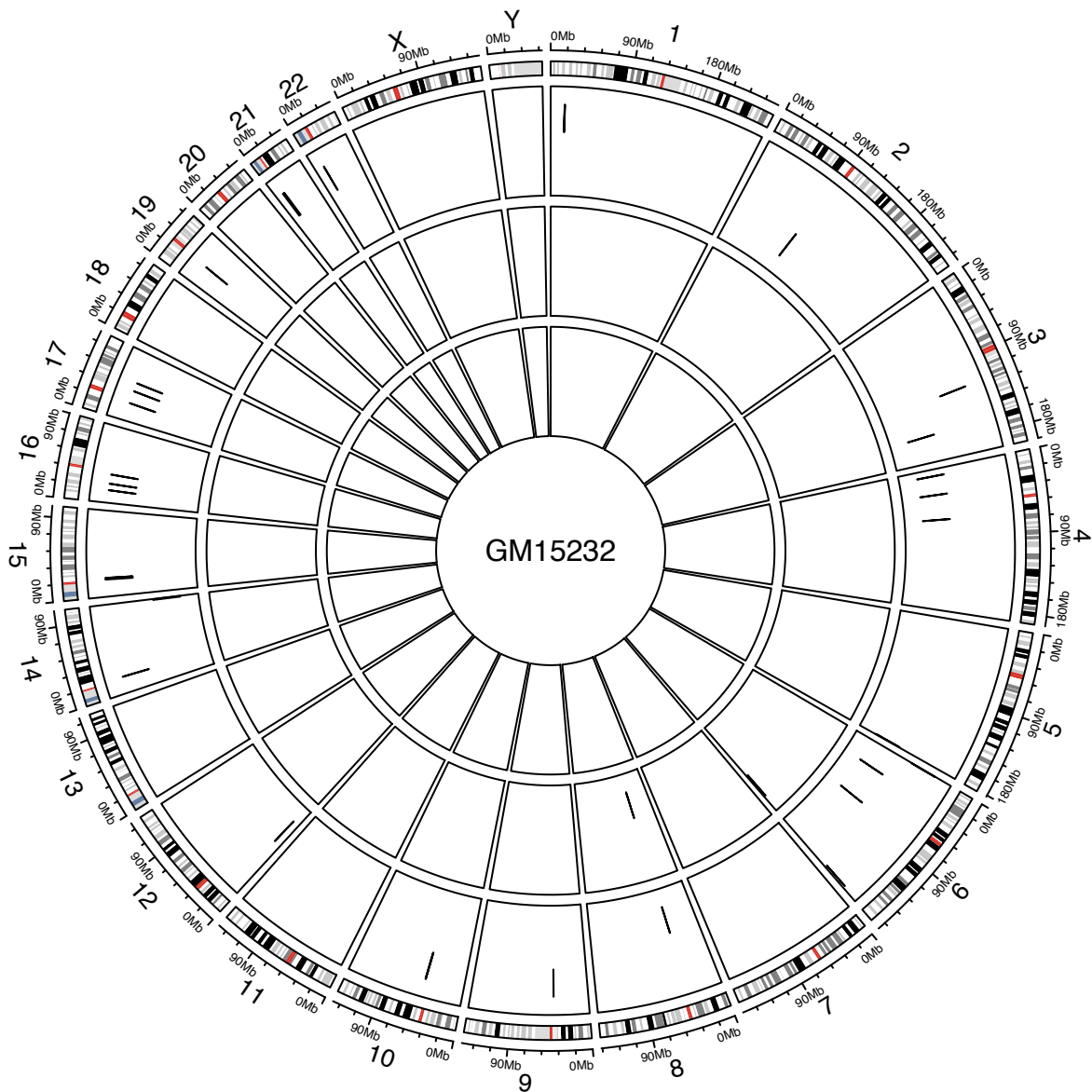

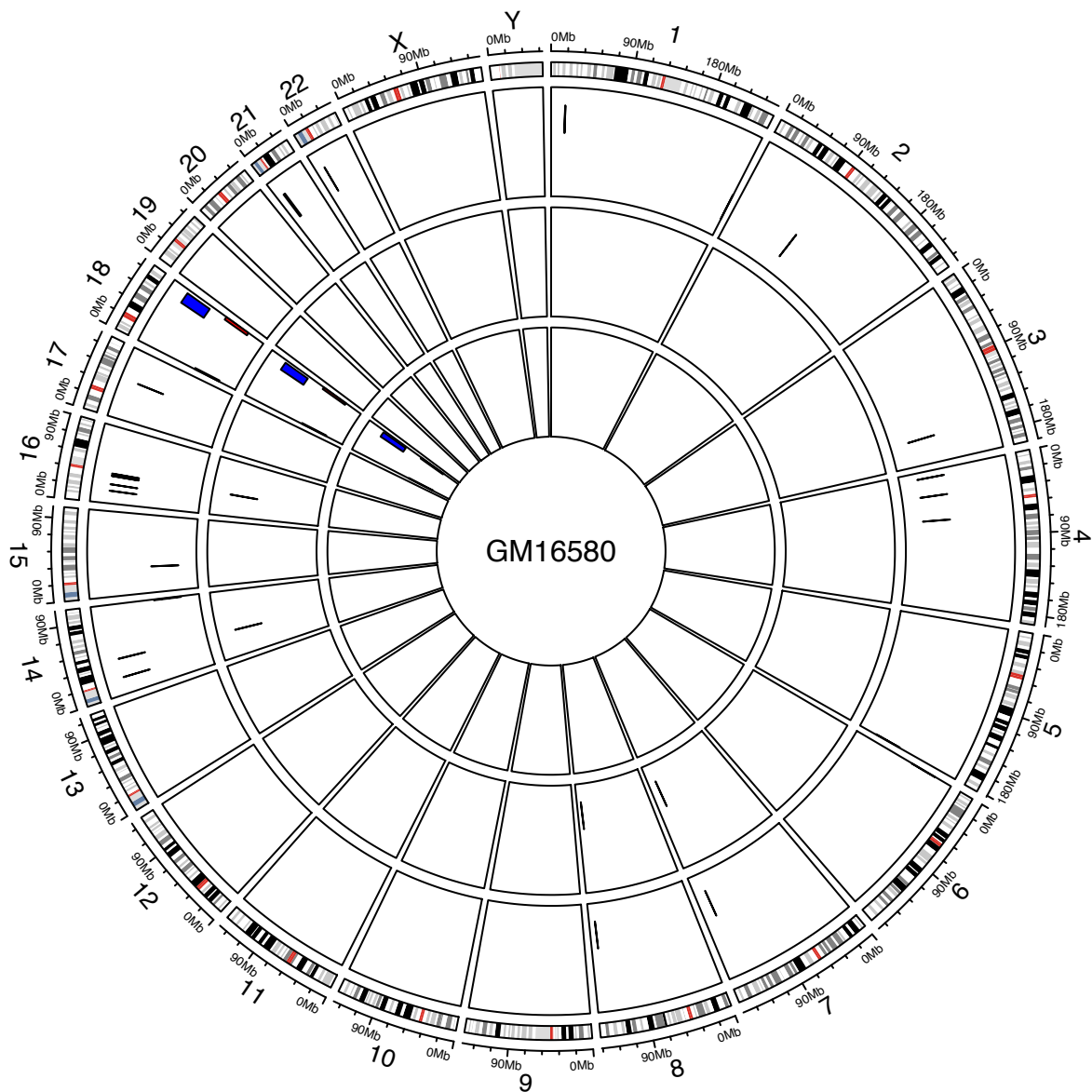

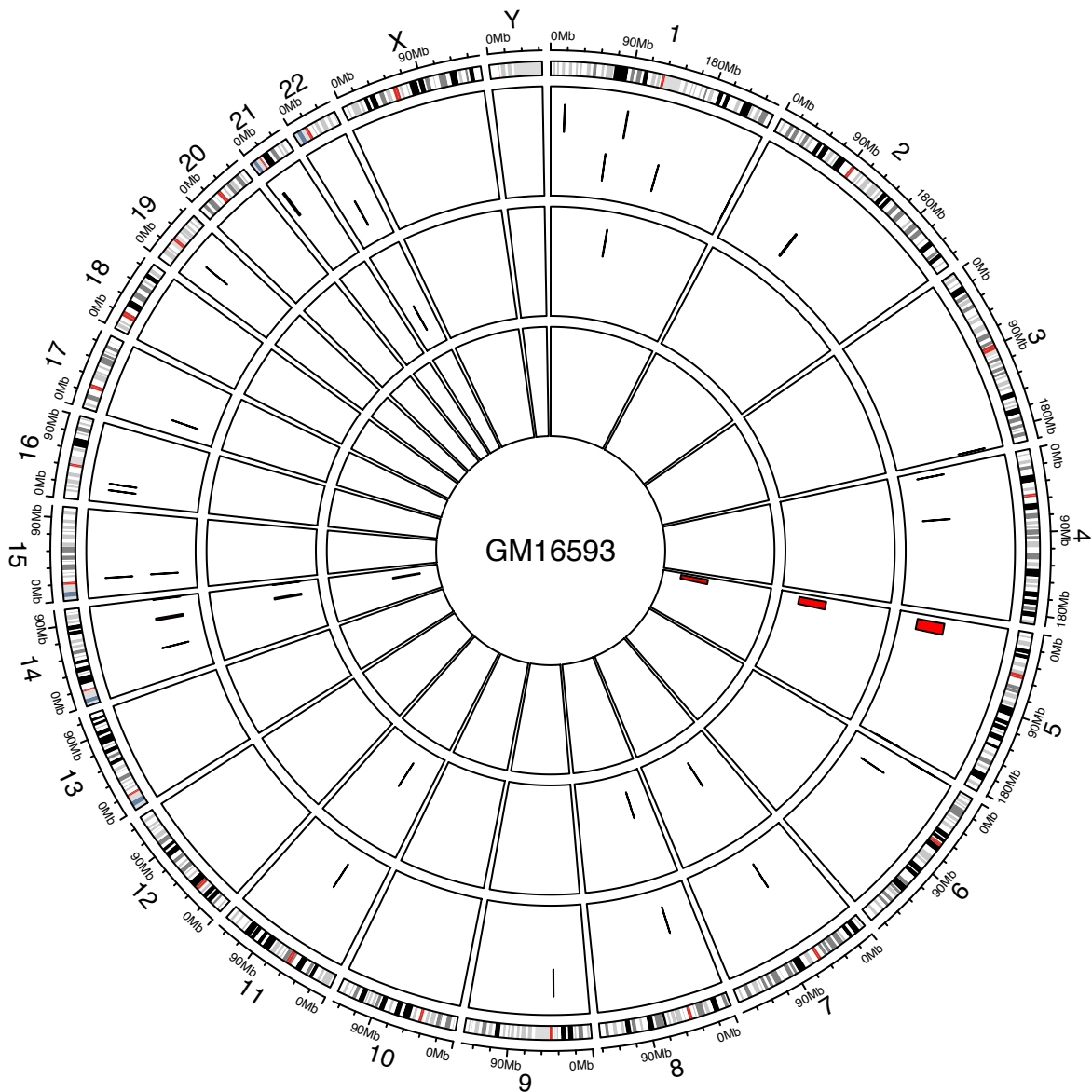

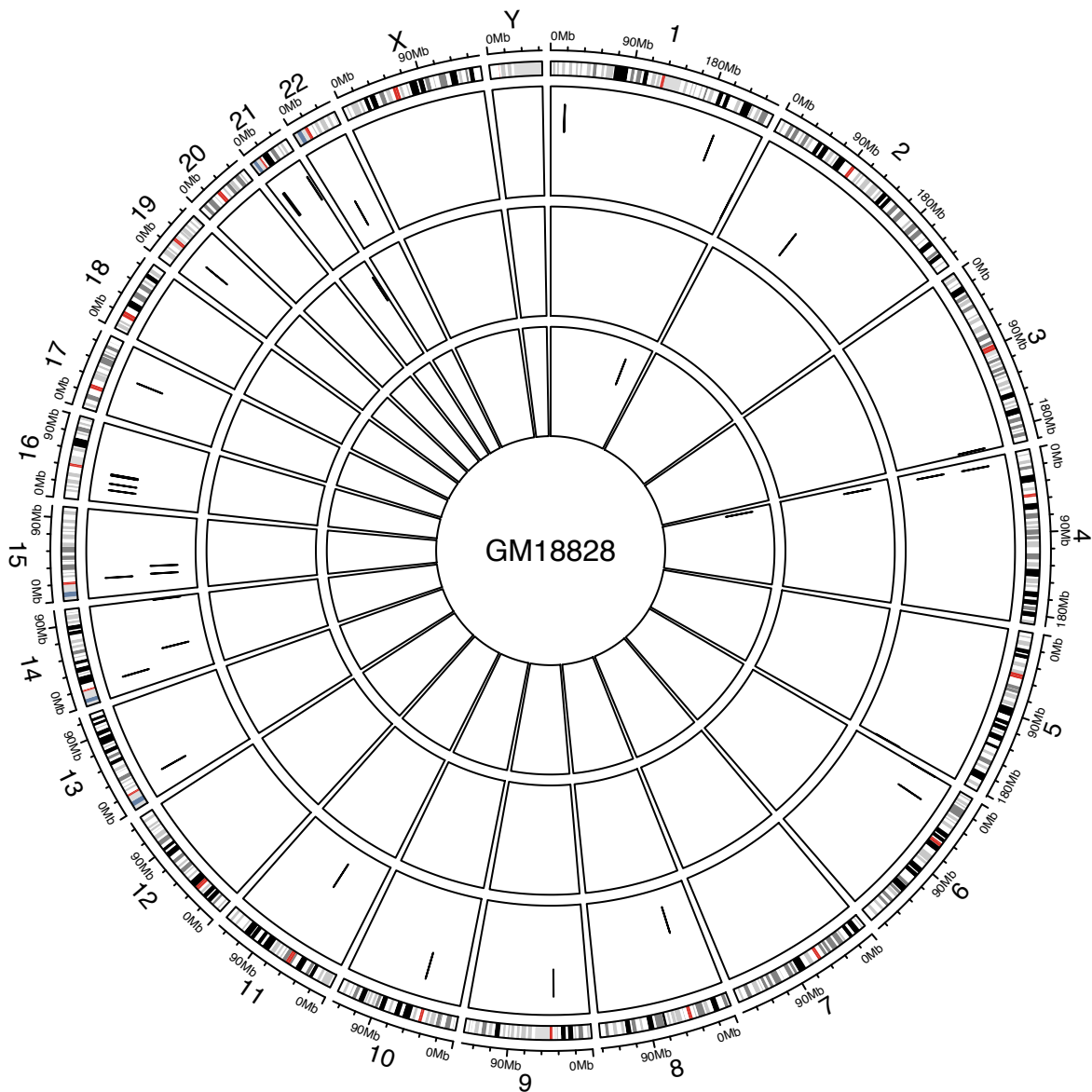

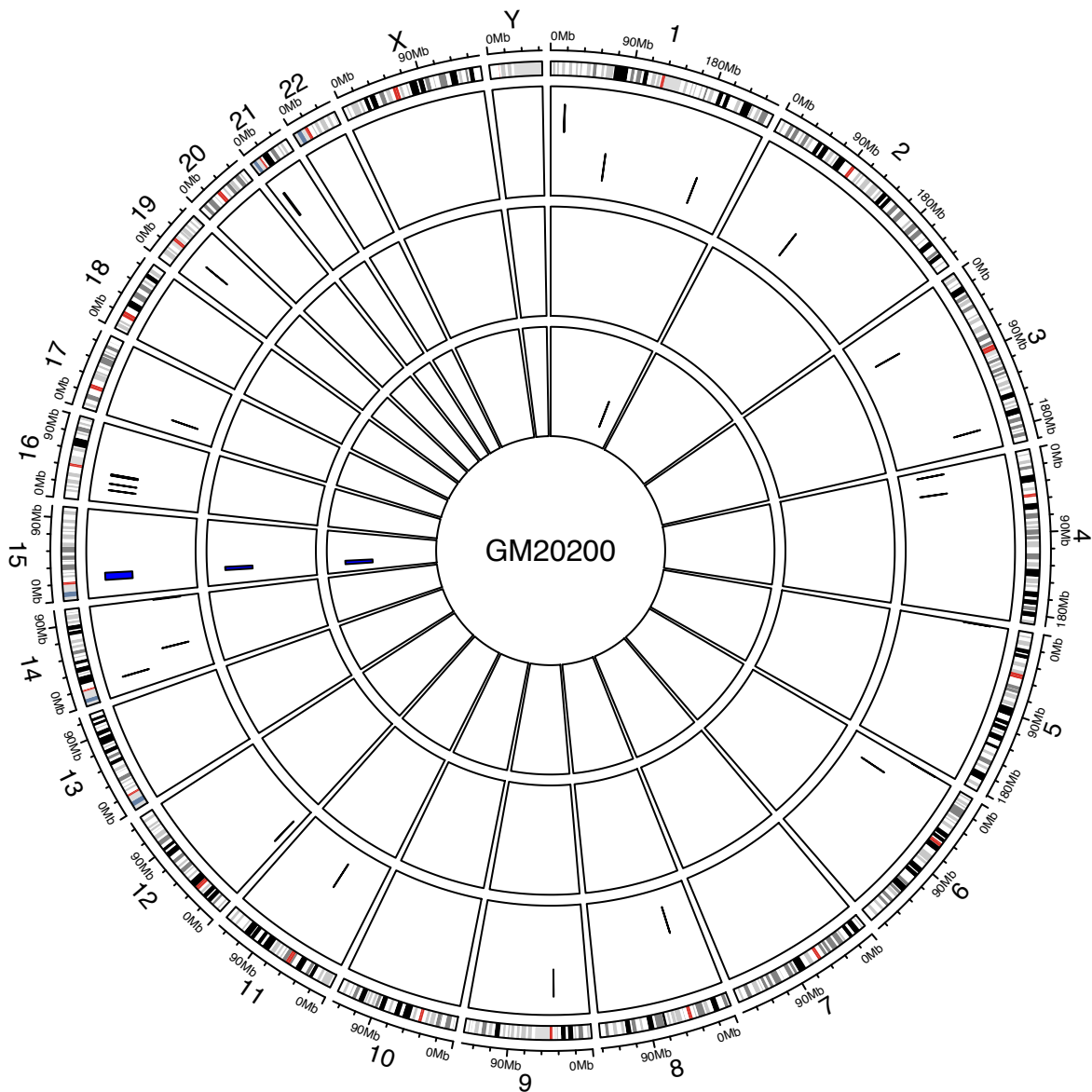

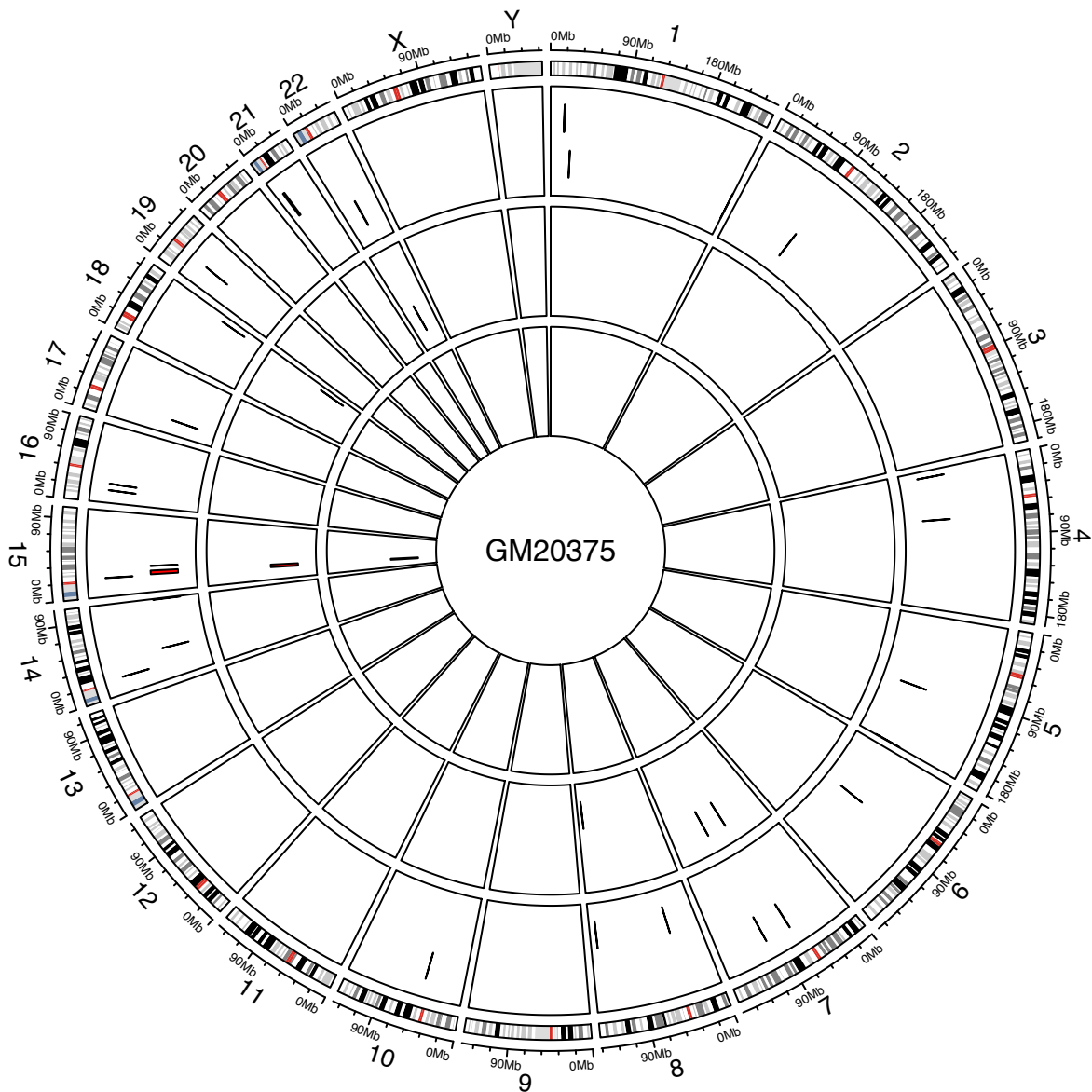

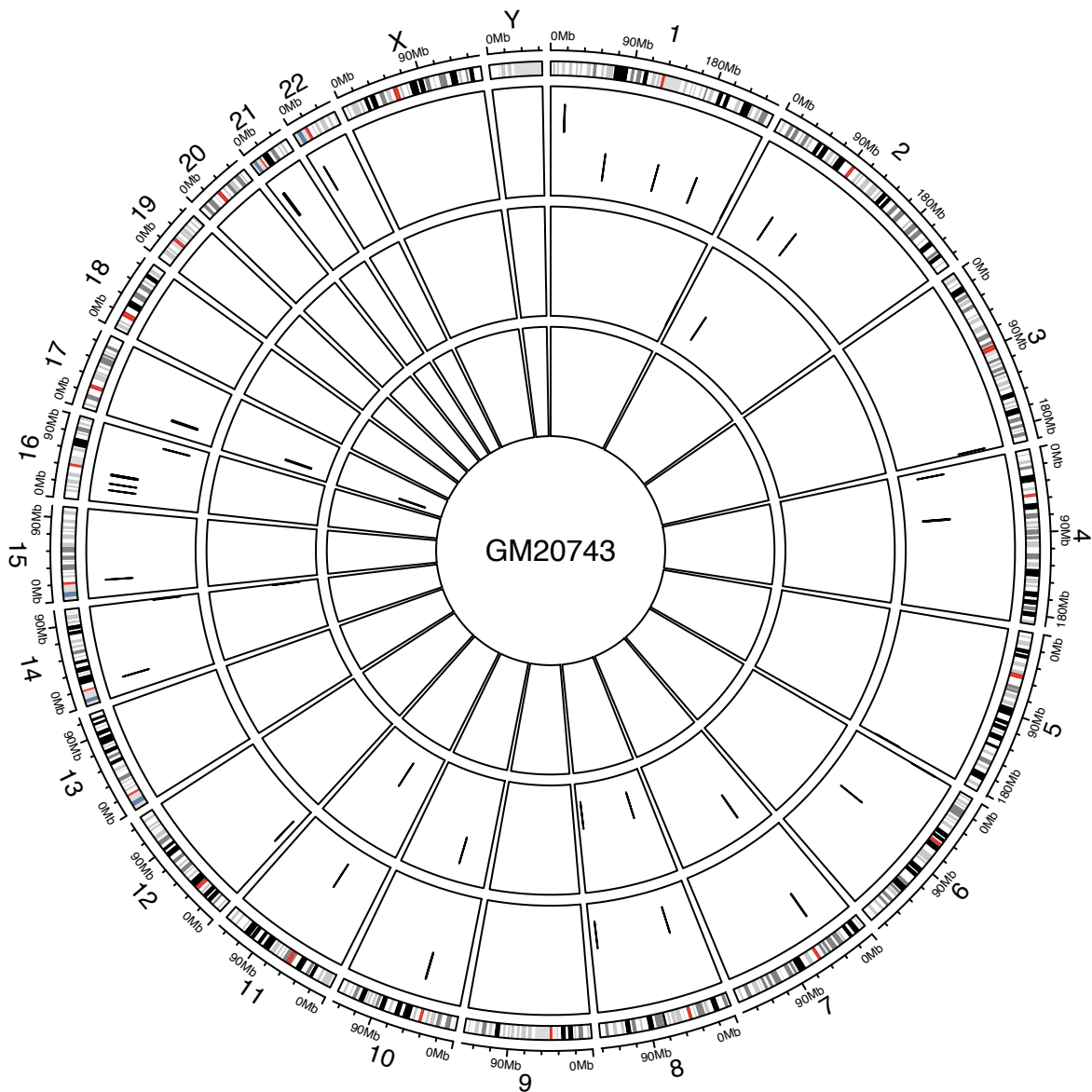

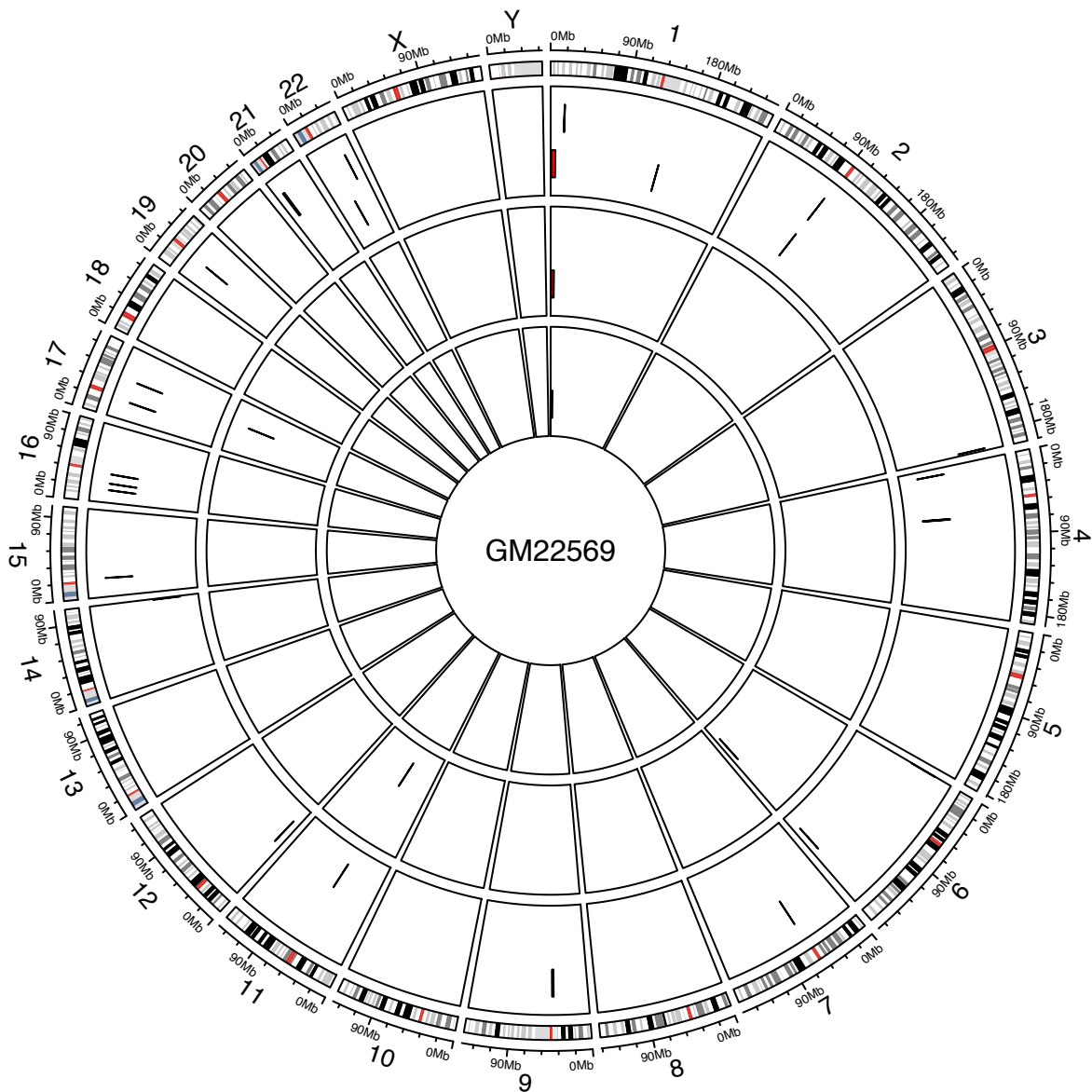

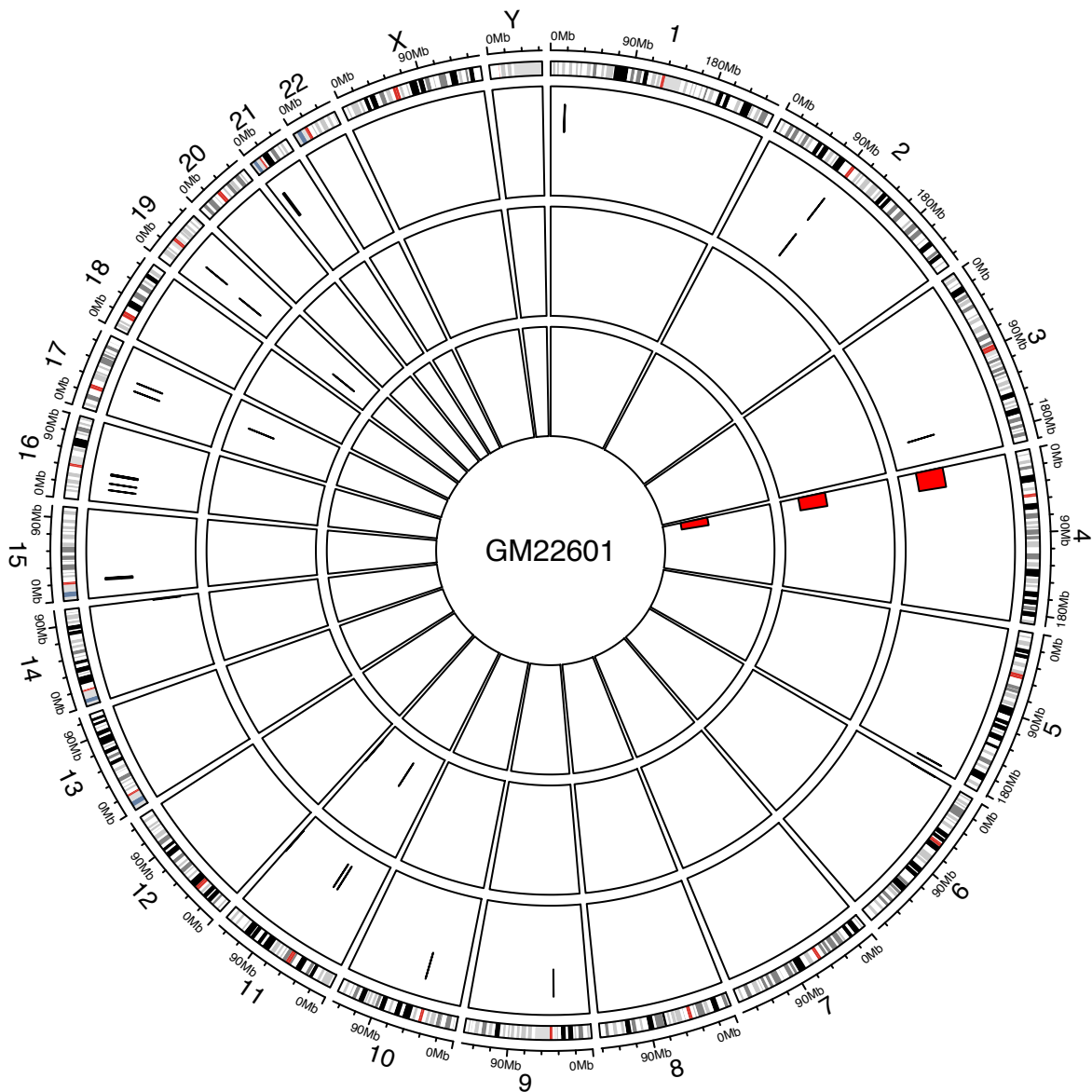

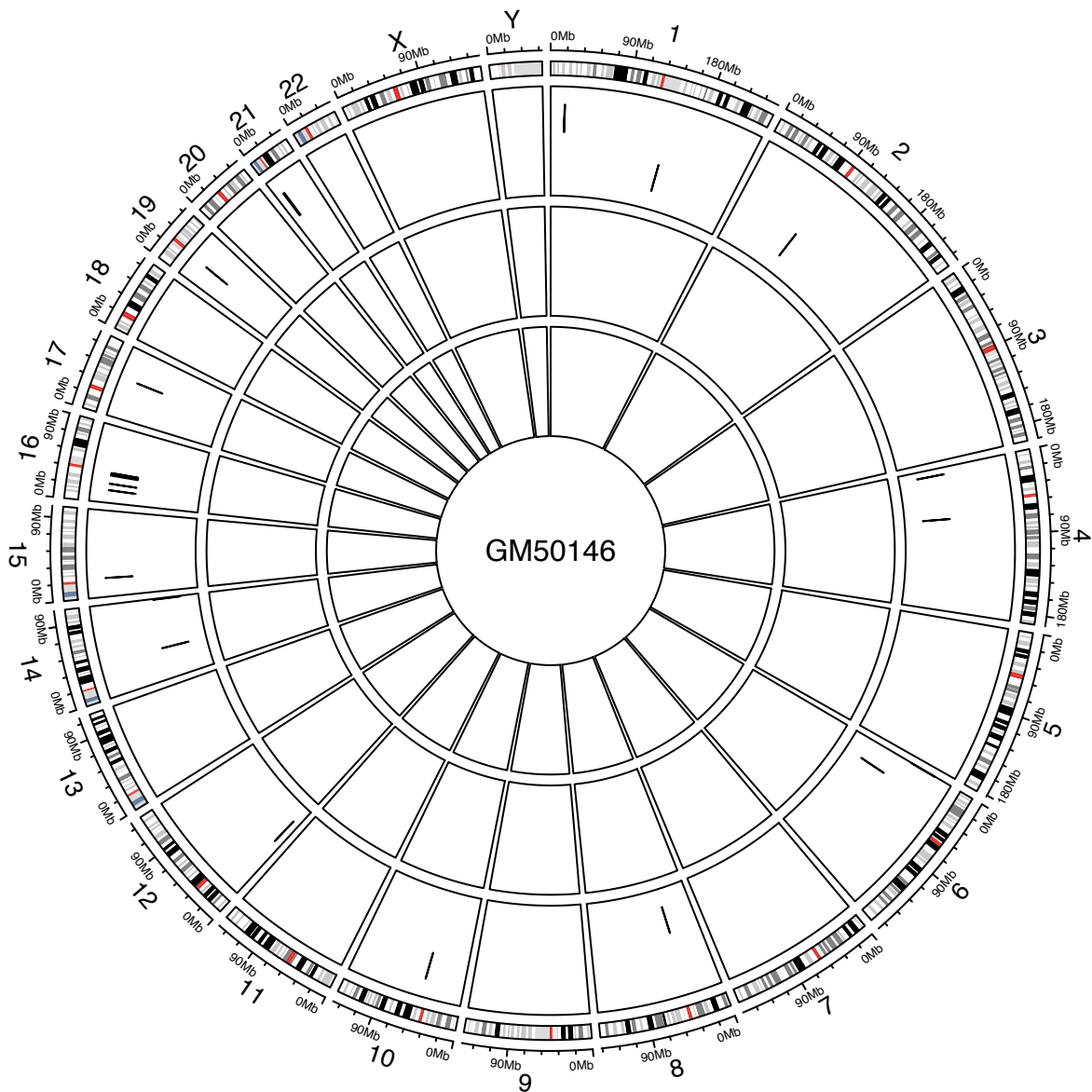

Supplement: Supplementary Figure S2 — Common and unique CNVs in the 31 testing samples detected by three different methods Each sub figure shows reported CNVs, from the inner circle to the outer circle, are from (1) Corriell Institute, (2) JAX-GM CMA, and (3) JAX-CNV on WGS. Deletions and duplications are colored red and blue, respectively. [file mmc3.pdf]

Figure S3.

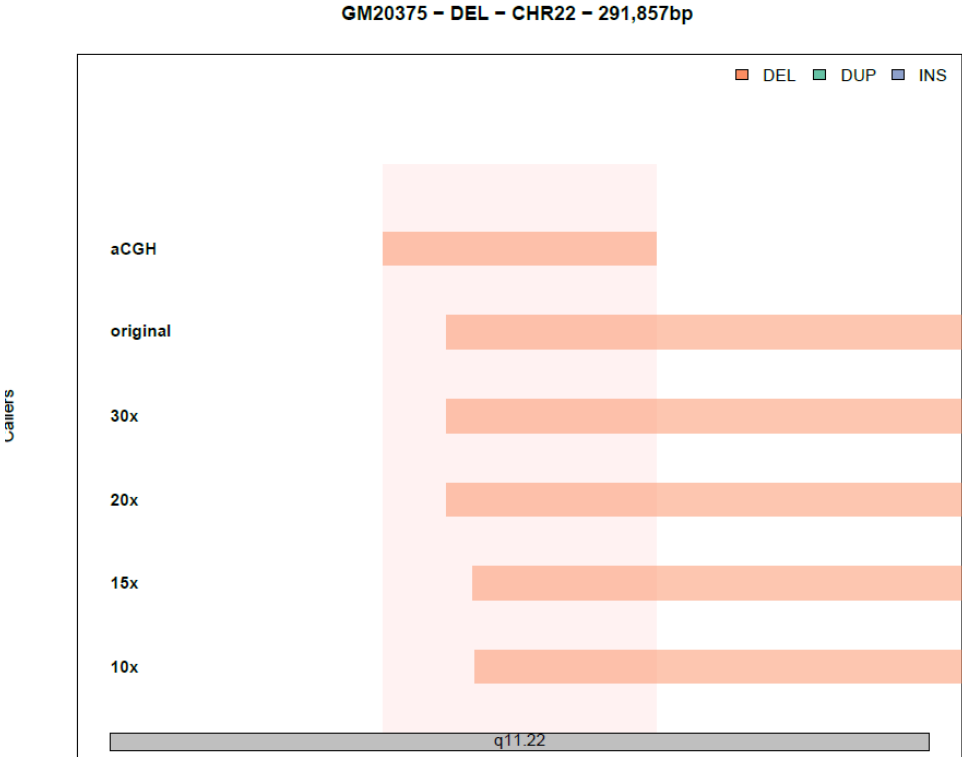

### GM20743 - DEL - CHR1 - 157,968bp

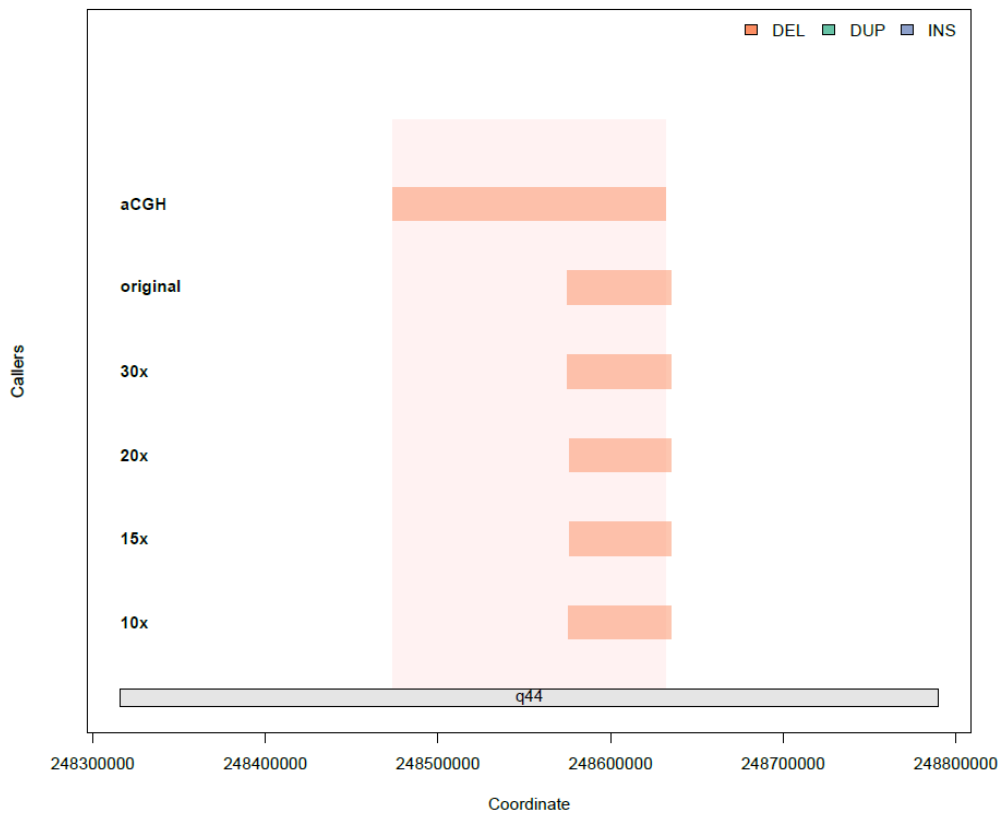

### GM20743 - DEL - CHR14 - 104,844bp

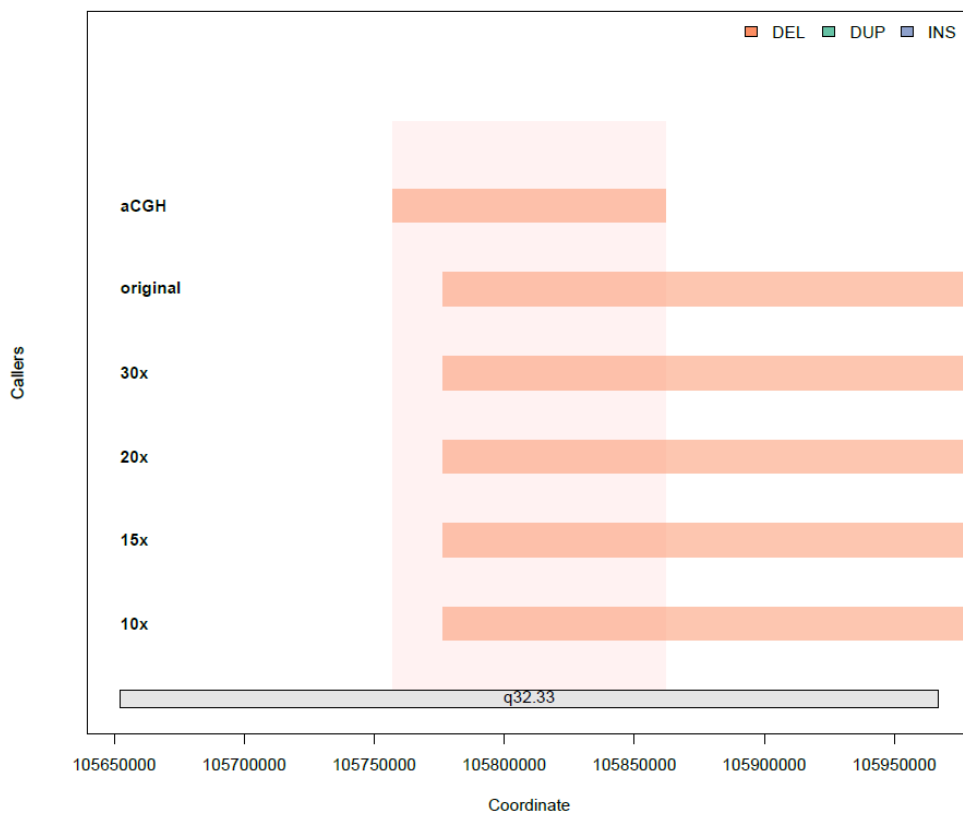

### GM09687 - DUP - CHR17 - 100,951bp

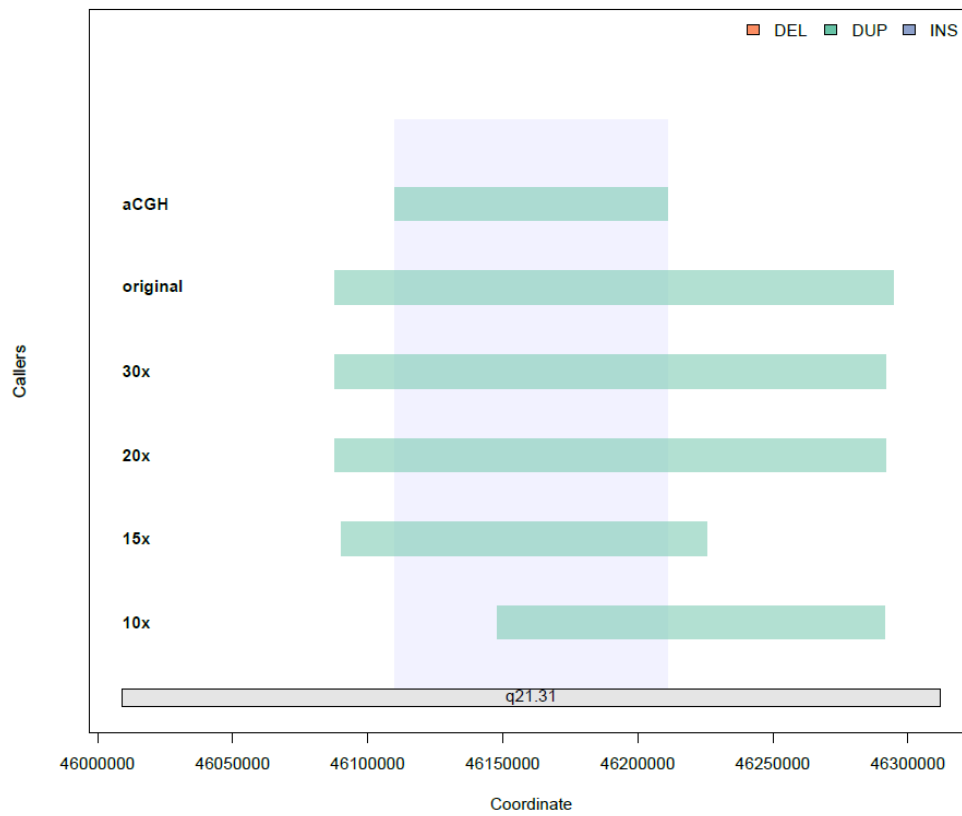

### GM13480 - DUP - CHR10 - 165,513bp

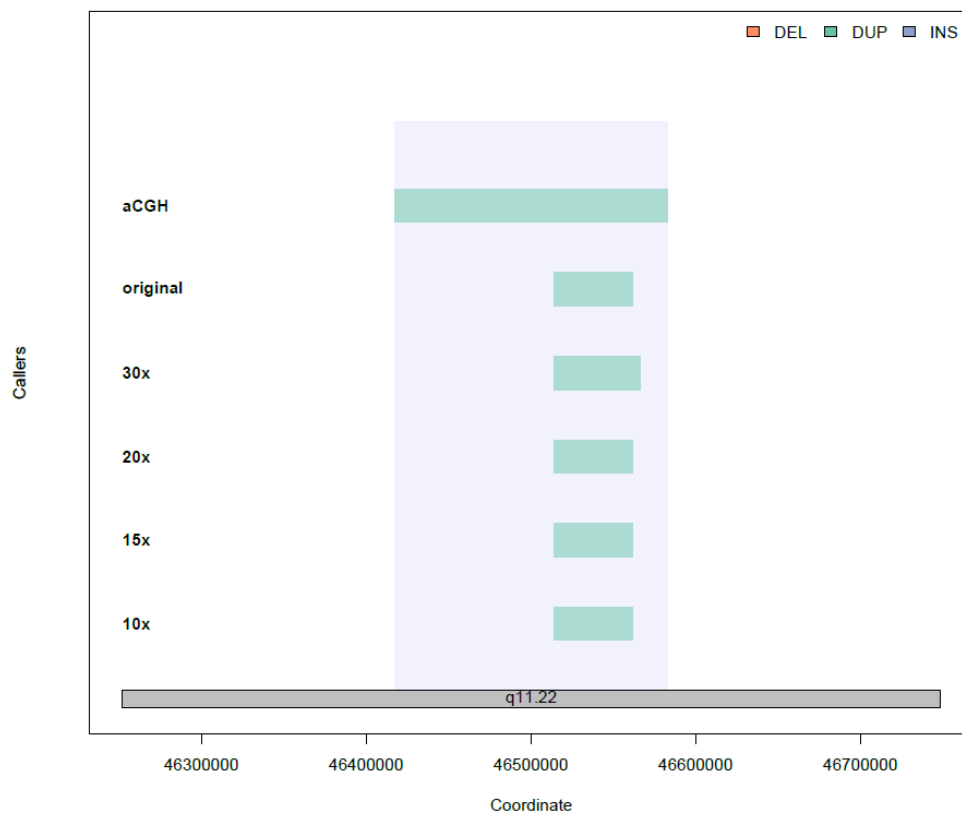

GM20743 - DUP - CHR10 - 182,732bp

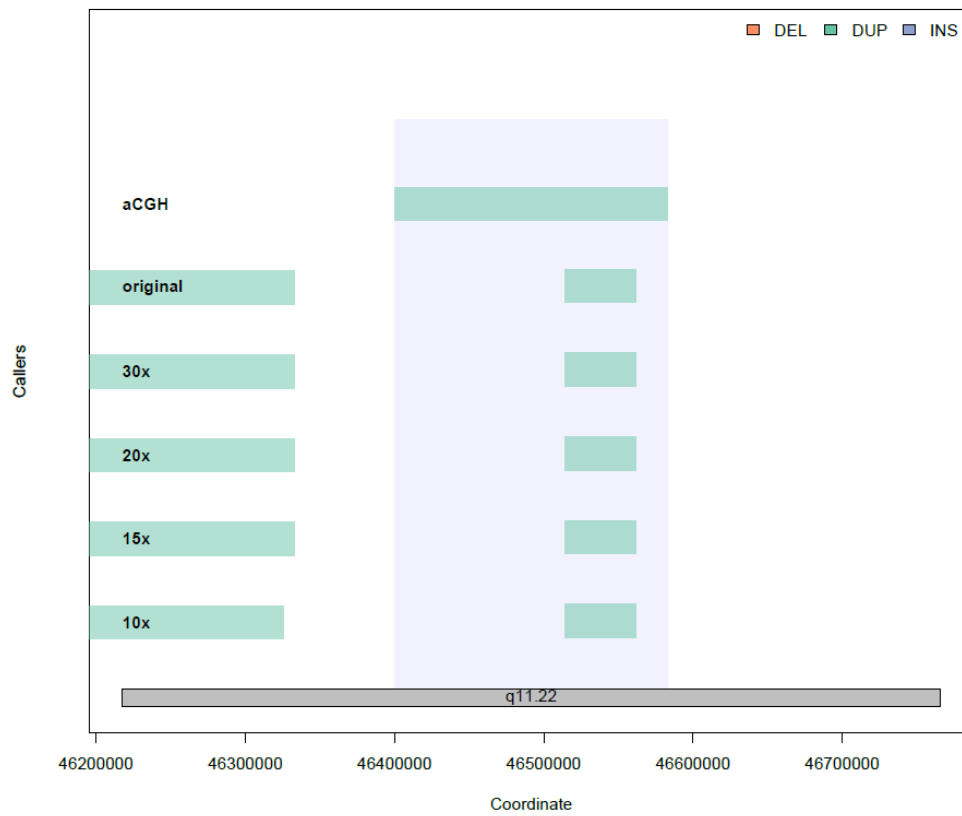

Supplement: Supplementary Figure S3 — JAX-CNV detected CNVs that were less than 50% reciprocal overlap with the ones detected by aCGH Three deletions and three duplications that did not meet the benchmark of 50% reciprocal overlap with the JAX-CNV calls, but they were still located in the same regions with either smaller and larger size ones. [file mmc4.pdf]

Figure S4.

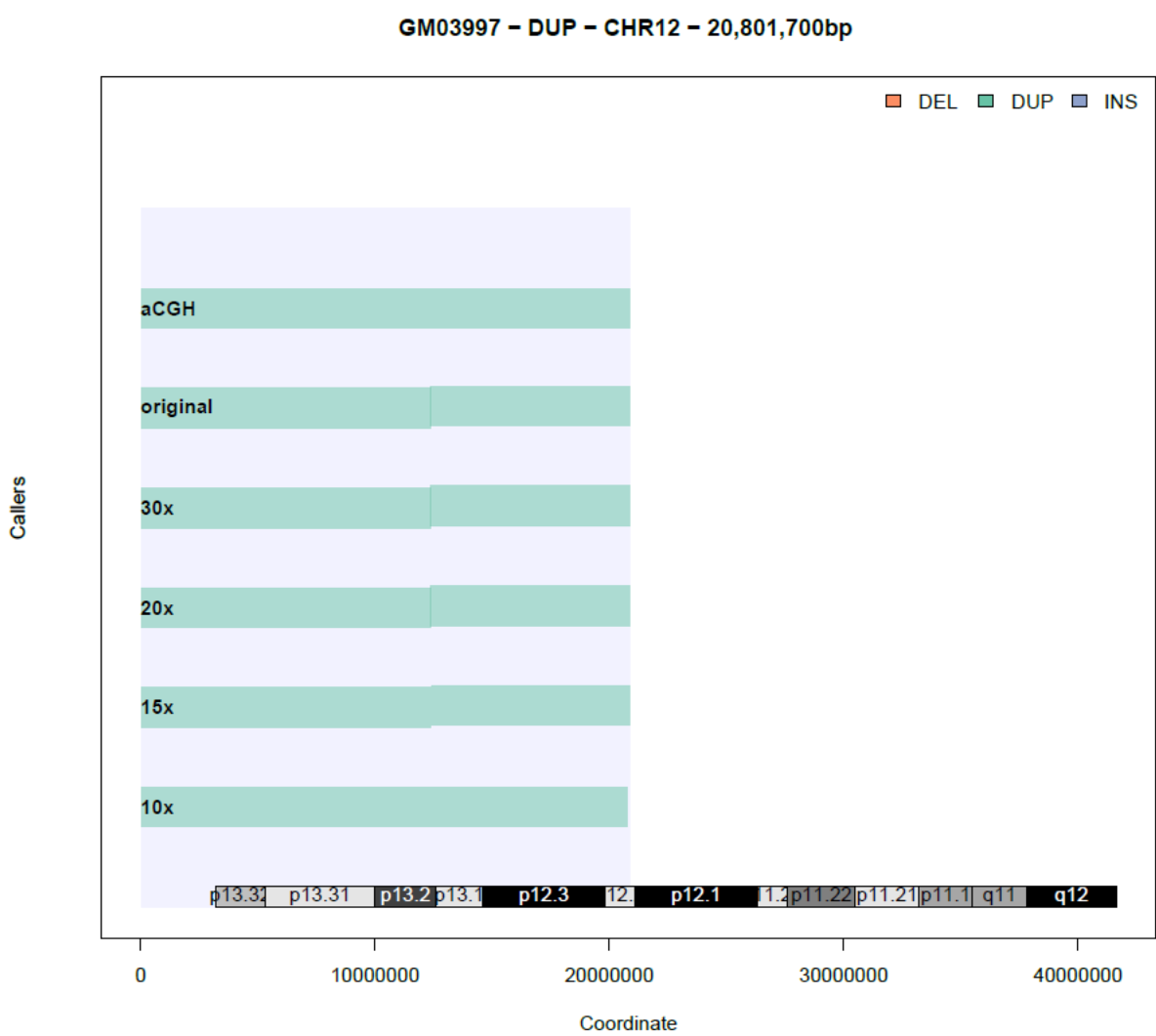

Supplement: Supplementary Figure S4 — JAX-CNV detected a huge duplication with an arm-level size (20.8 Mb) A 20.8 Mb duplication at the p arm of the chromosome 12 in the sample GM03997 was detected by JAX-CNV with two calls to cover it entirely. [file mmc5.pdf]

Figure S5.

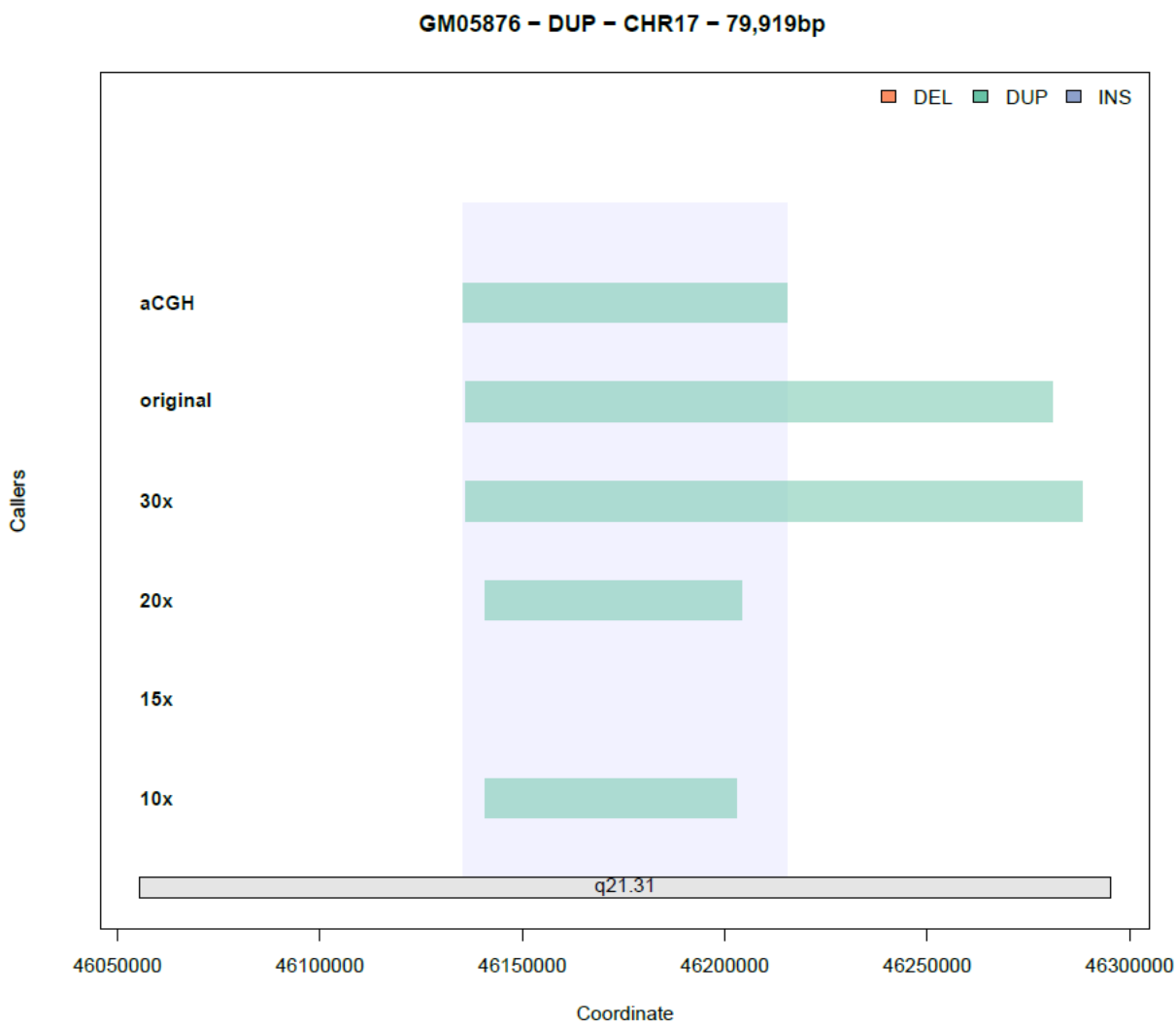

Supplement: Supplementary Figure S5 — A false negative result was observed with JAX-CNV at 15x WGS coverage One duplication of 79.919 kb located in q21.31 region was detected by aCGH and JAX-CNV at 30x, 20x and 10x WGS coverages, but was missed by JAX-CNVat 15x WGS coverage data, suggesting a false negative result. [file mmc6.pdf]

Figure S6.

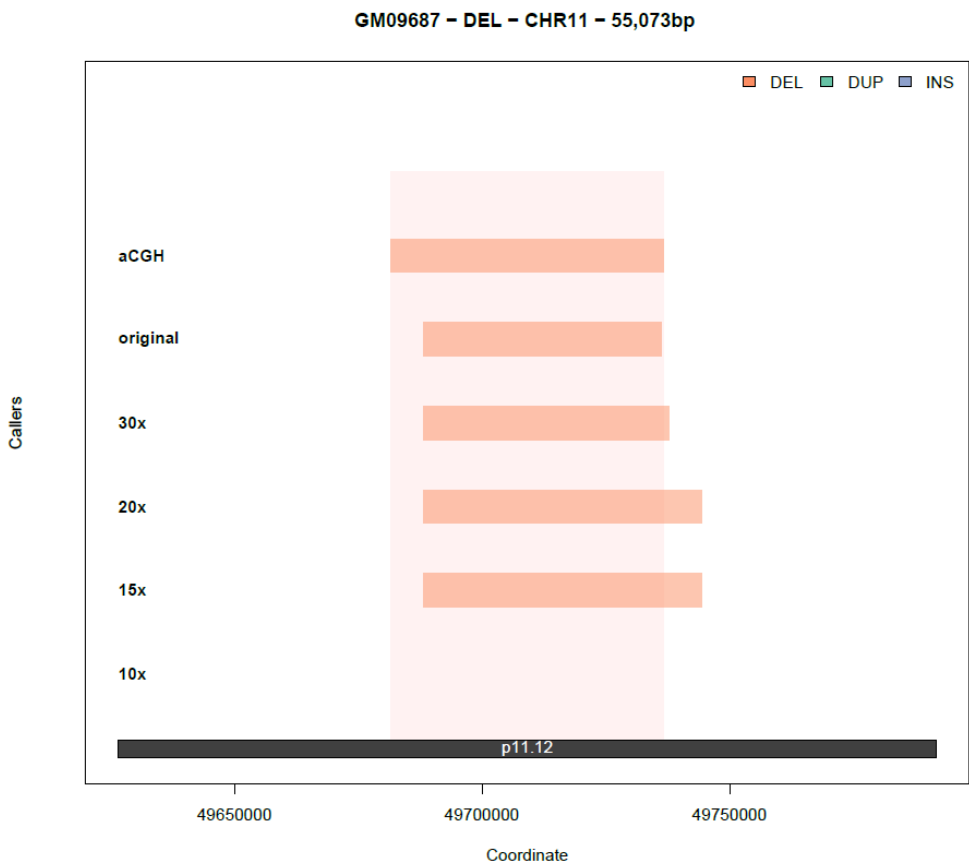

# GM08681 - DUP - CHR1 - 204,778bp

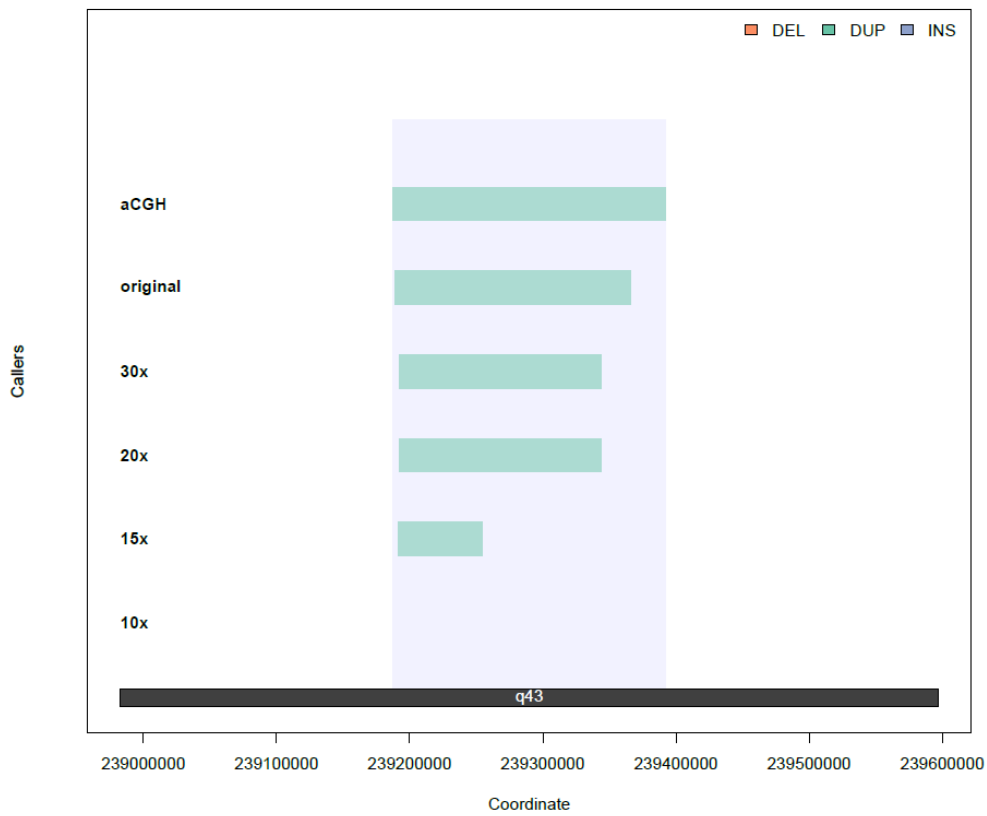

# GM11516 - DUP - CHR17 - 79,919bp

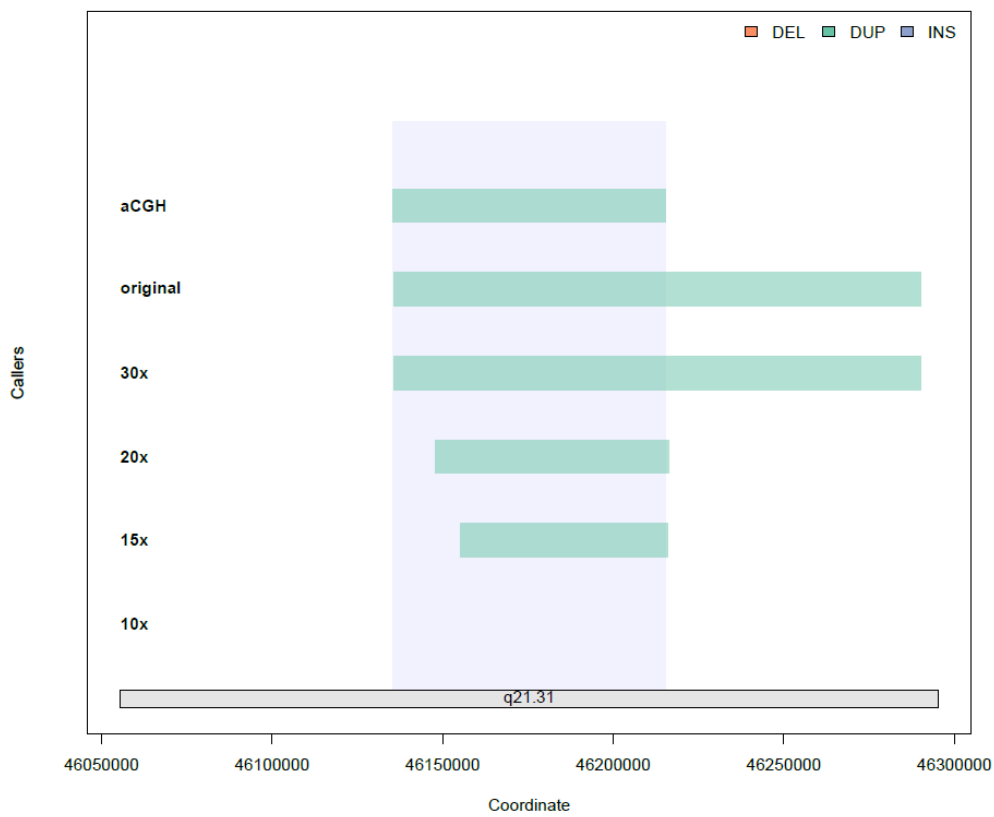

GM13480 - DUP - CHR17 - 52,130bp

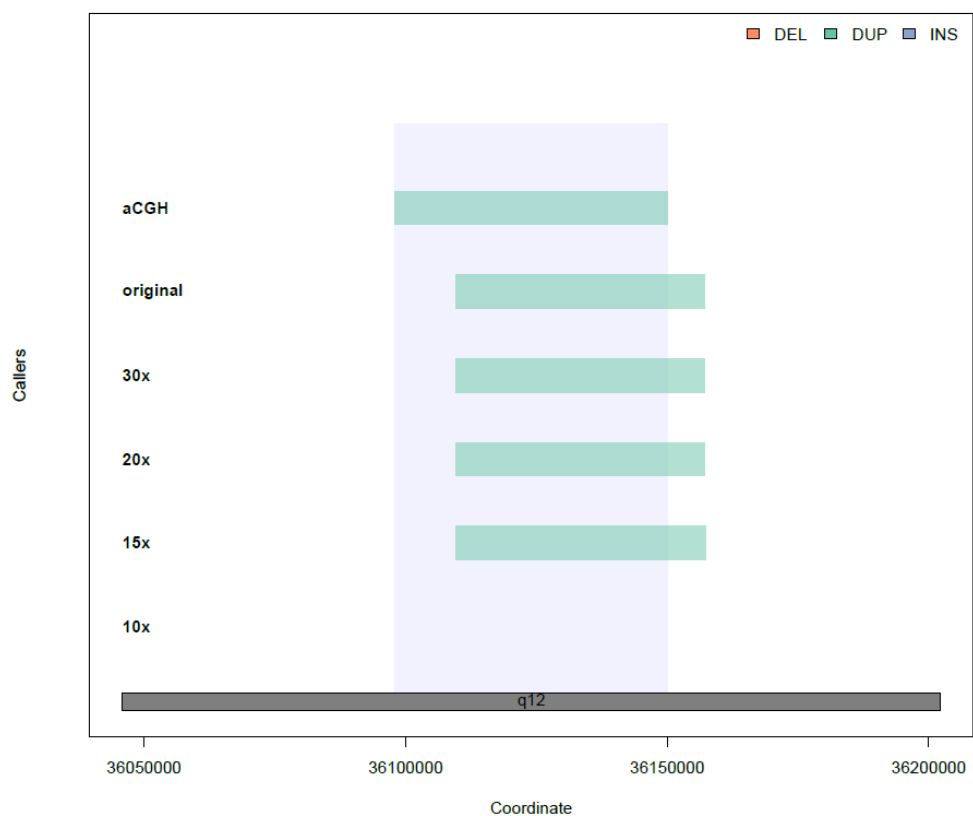

GM14164 - DUP - CHR17 - 79,919bp

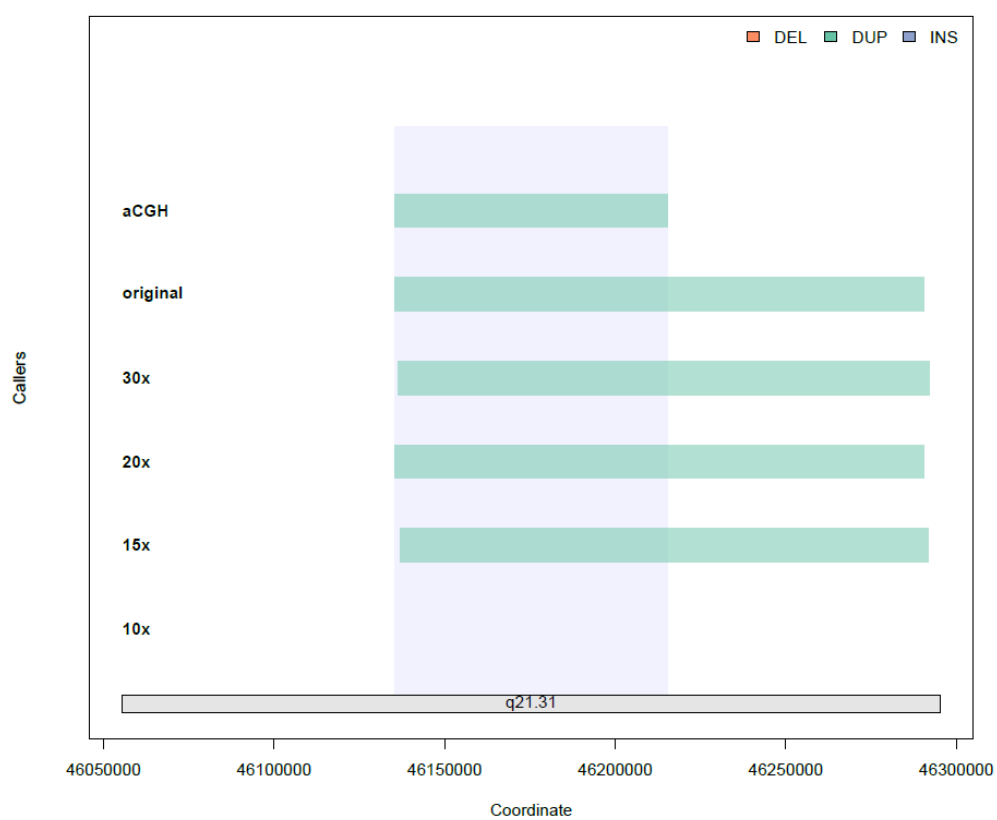

GM14164 - DUP - CHR22 - 148,797bp

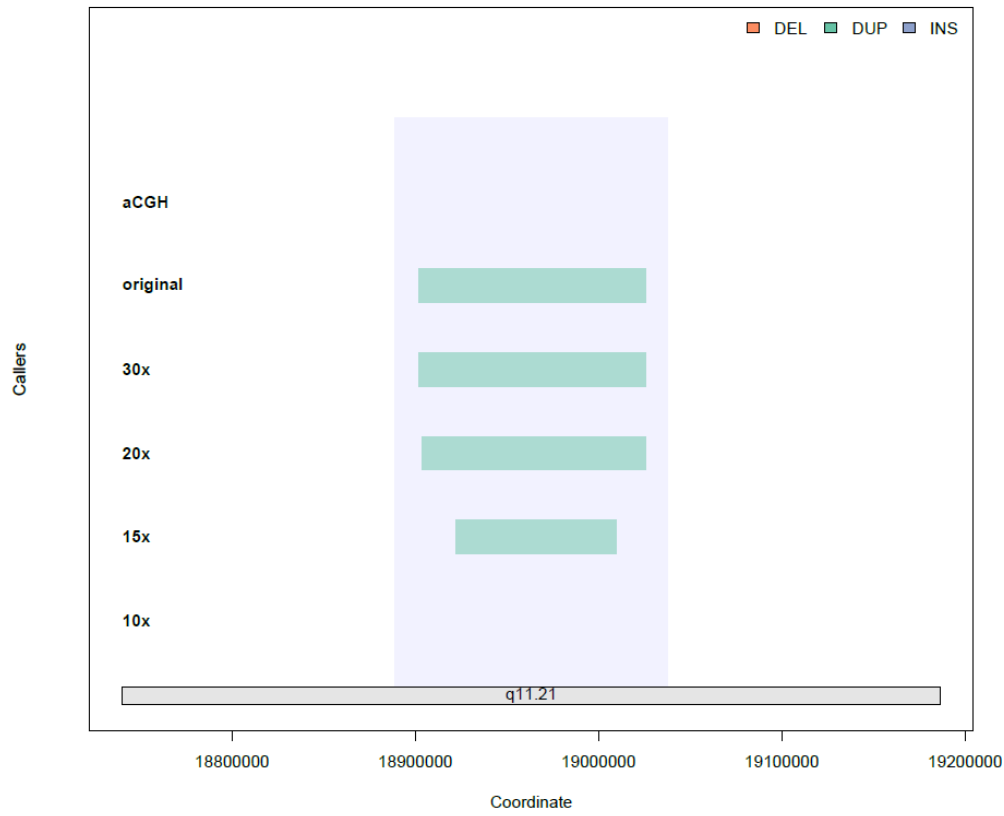

# GM18828 - DUP - CHR1 - 118,140bp

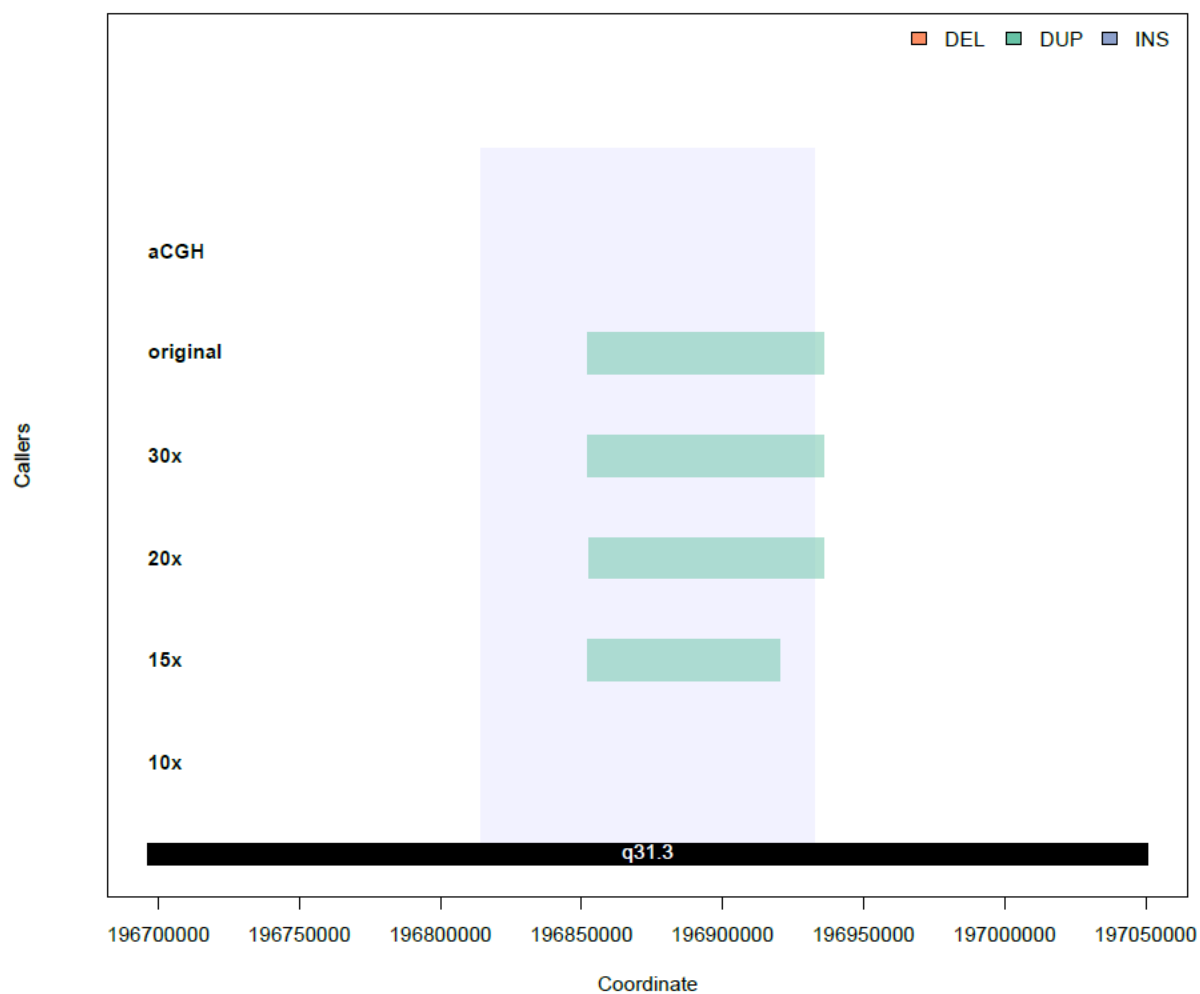

Supplement: Supplementary Figure S6 — Decreased sensitivity of JAX-CNV at 10x WGS coverage JAX-CNV was unable to detect one deletion and six duplications at 10x WGS coverage data. [file mmc7.pdf]

Figure S7.

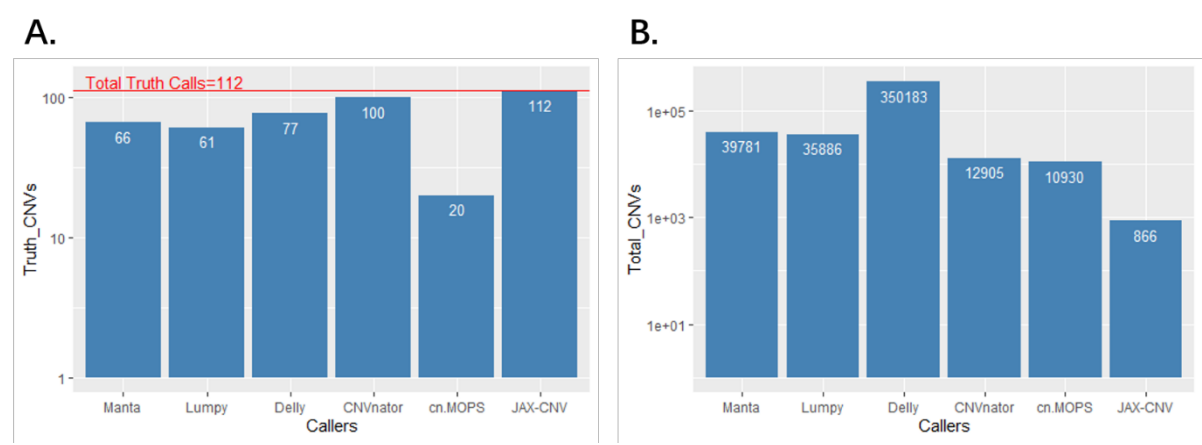

Supplement: Supplementary Figure S7 — The sensitivity and specificity of JAX-CNV are better than other calling algorithms A. The recalled CNVs in the truth set of each algorithm. B. Numbers of total calls reported by each algorithm. [file mmc8.pdf]
